# Electrochemically Driven Nickel‐Catalyzed Enantioselective Hydro‐Arylation/Alkenylation of Enones

**DOI:** 10.1002/advs.202405926

**Published:** 2024-09-12

**Authors:** Zenghui Ye, Weiyuan Ma, Xi Zhang, Huaqing Liu, Fengzhi Zhang

**Affiliations:** ^1^ School of Pharmacy Hangzhou Medical College Hangzhou Zhejiang 311399 China

**Keywords:** asymmetric synthesis, chiral nickel catalysts, electrochemical reductive conjugate arylation, electrochemistry

## Abstract

Herein, the study reports the first electrochemical nickel‐catalyzed enantioselective hydro‐arylation/alkenylation of enones in an undivided cell with low‐cost electrodes in the absence of external reductants and supporting electrolytes. Aryl bromides/iodides/triflates or alkenyl bromides are employed as electrophiles for the efficient preparation of more than 56 valuable β‐arylated/alkenylated ketones in a simple manner (up to 97% yield, 97% ee). With the advantages of electrochemistry, excellent functional group tolerance and late‐stage modification of complex natural products and pharmaceuticals made the established protocol greener and more economic. Mechanism investigation suggests that a Ni^I^/Ni^III^ cycle may be involved in this electro‐reductive reaction rather than metal reductant driven Ni^0^/Ni^II^ cycle. Overall, the efficient electrochemical activation and turnover of the nickel catalyst avoid the drawbacks posed by the employment of stoichiometric amount of sensitive metal powder reductants.

## Graphical abstract

Asymmetric electrochemical synthesis is recognized as a powerful and scalable methodology for organic synthesis. This study presents the first example of electrochemical enantioselective Ni‐catalyzed reductive conjugate hydro‐arylation/alkenylation of enones in an undivided cell, affording more than 56 valuable functionalized β‐arylated ketones in good to excellent enantioselectivity. Furthermore, various bio‐relevant compounds are successfully synthesized through this scalable protocol.

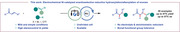

## 1 Introduction

**Figure 1:**
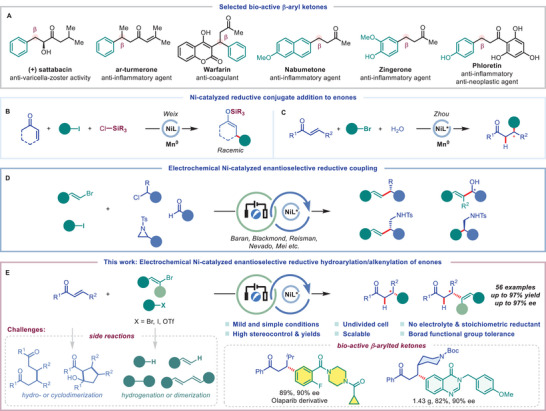
A) Selected bio‐active β‐aryl ketones. B,C) Previous reports of Ni‐catalyzed reductive conjugate addition to enones. D) Electrochemical enantioselective reductive cross‐couplings. E) This work and challenges.

Enantiopure β‐arylated ketone, a structure motif frequently found in natural products, materials, pharmaceuticals (**Figure** **1**A), or agrochemicals, can normally be prepared by rhodium catalyzed asymmetric conjugate addition of Michael acceptors with aryl nucleophiles such as organocuprate or boron reagents which ultimately come from the corresponding readily available aryl halides.^[^ ^1^, ^2^, ^3^, ^4^, ^5^ ^]^ Nickel catalysis has become a growing and empowering area of research over the past decade, providing new reactivity modes toward organic synthesis and have revolutionized synthetic strategies in pharmaceuticals and materials.^[^ ^6^, ^7^, ^8^, ^9^ ^]^ As early as the 1980s, Ronchi, Lebedev, Sustmann, and Condon have reported zinc‐ or electrochemical‐promoted nickel‐catalyzed reductive conjugate addition of activated olefins with organic halides, respectively.^[^ ^10^, ^11^, ^12^, ^13^ ^]^ However, only Michael acceptors without β‐substitution provide the corresponding products in high yields in those reports. In 2013, Weix and co‐workers revealed a nickel‐catalyzed reductive addition of aryl halides to enones via allylnickel species for the preparation of β‐arylated ketone, which required the use of stoichiometric amount of trialkylsilyl chlorides and manganese powder (Figure 1B).^[^ ^14^ ^]^ Recently, Zhou and co‐workers reported a metal reductant driven Ni‐catalyzed enantioselective reductive conjugate arylation of activated olefins and imines in the presence of super‐stoichiometric manganese powder (Figure 1C).^[^ ^15^, ^16^, ^17^, ^18^ ^]^ Despite the significant achievement of these preeminent work, there are still improving space as they rely on the use of super‐stoichiometric sensitive, flammable and hazardous metal reductant, glove box, and require more than one day to complete the transformation due to the slow turnover‐limiting reduction of the Ni catalyst by metal reductant,^[^ ^19^, ^20^ ^]^ which somehow limit their practical application.

Electrosynthesis, employing readily available electrical current as a sustainable and inherently safe redox reagent, achieving extreme oxidation or reduction capacity easily by varying the current or voltage, is recognized as a powerful and scalable methodology for organic synthesis.^[^ ^21^, ^22^, ^23^, ^24^, ^25^, ^26^, ^27^, ^28^, ^29^, ^30^, ^31^ ^]^ With the renaissance of electrosynthesis, the asymmetric electrocatalysis involving anodic oxidation has made significant progress in recent years.^[^ ^32^, ^33^, ^34^, ^35^, ^36^, ^37^, ^38^, ^39^, ^40^, ^41^, ^42^, ^43^, ^44^, ^45^ ^]^ However, there are few research focus on the asymmetric electrochemical reductive reactions (Figure 1D).^[^ ^46^, ^47^, ^48^, ^49^, ^50^, ^51^, ^52^, ^53^ ^]^ In 1997, Durandetti and coworkers described the first example of asymmetric electro‐reductive coupling (ERC) between α‐chloro esters and aryl halides by using chiral auxiliaries.^[^ ^46^ ^]^ In 2019, Reisman and coworkers reported a Ni/Box catalyzed enantioselective ERC of vinyl bromides and benzyl chlorides.^[^ ^47^ ^]^ In 2020, Mei and coworkers developed a Ni/Pyrox catalyzed ERC of aryl bromides for the synthesis of biaryl atropisomers.^[^ ^48^ ^]^ In 2021, Baran and coworkers described a Ni/Cr co‐catalyzed electro‐Nozaki‐Hiyama‐Kishi (*e*‐NHK) coupling reaction for the synthesis of chiral alcohols.^[^ ^49^ ^]^ In 2022, Cheng and coworkers developed a Pd‐catalyzed asymmetric allylic 4‐pyridinylation ERC reaction.^[^ ^50^ ^]^ Mei and coworkers also reported a paired electrolysis‐enabled nickel‐catalyzed enantioselective ERC of aryl bromides and α‐chloro esters.^[^ ^51^ ^]^ Recently, Nevado group and Mei group demonstrated the nickel catalyzed enantioselective ERC of aziridines with vinyl bromides and aryl iodides, respectively.^[^ ^52^, ^53^ ^]^ These excellent studies lay the foundation of electrochemical nickel catalyzed enantioselective reductive coupling.

With our continued interest in developing novel electrosynthetic methodologies,^[^ ^54^, ^55^, ^56^, ^57^, ^58^, ^59^, ^60^, ^61^ ^]^ we envision the possibility of using powerful and scalable electrosynthesis to achieve the enantioselective reductive addition of electrophiles to enones (Figure 1E). Notable features of this strategy include: a) using a simple undivided cell with readily available and low‐cost stainless steel electrodes; b) avoiding the use of electrolyte and external base; c) mild and efficient electro‐reductive conditions with good functional group compatibility and shorter reaction time compared to metal reductant; d) the adjustable reductive potential by replacing the stoichiometric reductant with electricity and enable the fast turnover of chiral nickel catalysts; e) scalable synthesis and late‐stage modification of bio‐relevant molecules. The successful conduction of this strategy relies on addressing the following significant challenges: First, the inhibition of side reactions such as the reductive hydro‐ or cyclodimerization of enones and the reductive hydrogenation or dimerization of aryl bromides during electrolysis.^[^ ^48^, ^62^, ^63^ ^]^ Second, the perfect match of nickel catalyst and chiral ligand to achieve the wide substrate scopes, excellent catalysis effect, and high enantioselectivities.^[^ ^13^, ^64^, ^65^, ^66^ ^]^

## 2 Results

### 2.1 Reaction Optimization

Initially, (*E*)‐chalcone (**1a**) and 4‐bromotoluene (**2a**) were selected as model substrates to identify the suitable reaction conditions. After extensive screening of conditions, electrolysis of a solution of **1a**, **2a**, NiBr_2_DME, chiral isoquinox ligand **L1** in an undivided cell equipped with 304 stainless‐steel electrodes as anode and cathode under nitrogen atmosphere, afforded **3a** in 93% yield and 92% ee with 74% faraday efficiency (**Table** **1**, entry 1). Other isoquinox ligand **L2‐L6** with different side‐arm group such as isopropyl, benzyl, phenyl, *sec*‐butyl, and indanyl led to moderate yields (43‐53%) and slightly lower stereoselectivity (68‐85% ee), which indicated the *tert*‐butyl group might be the best side‐arm group (Table 1, entry 2). Other 3‐methyl‐pyridine ligands **L7‐L9** gave slightly lower yield (54–66%) and enantioselectivities (60–82% ee) of **3a** (Table 1, entries 3–4).

**Table 1:** Optimization of the reaction conditions.^a^ ^)^

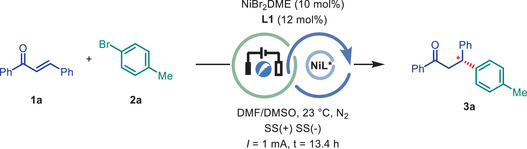			
Entry	Variation from standard conditions	Yield [%]^b^ ^)^	ee [%]^c^ ^)^
1	none	93	92
2	L2‐L6 instead of L1	43‐53	68–85
3	L7 instead of L1	66	82
4	L8‐L9 instead of L1	54‐55	60‐70
5	L10 instead of L1	72	94
6	*I* = 1.5 mA, 9 h	98	86
7	*I* = 0.5 mA, 26.8 h	90	92
8	DMF, DMAc, MeCN or DMSO as solvent	25–50	91‐92
9	Air	nr	–
10	Zn(+) Ni foam (‐)	trace	–
11	Zn(+) Ni foam (‐) with *p*‐I‐Tol, *I* = 10 mA, t = 2 h	75	92
12	Fe(+) Ni foam (‐)	92	92
13	0.1 mmol scale, t = 6.7 h	86	92
14	w/o electric current	nr	–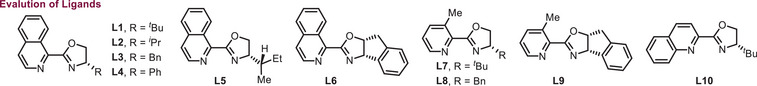			

^a)^ Reaction conditions. 1a (0.2 2 mmol), 2a (1.5 equiv.), NiBr2DME (10 mol.%), Ligand (12 mol.%), DMF/DMSO (1 1 mL/1 1 mL), N_2_, under 1 1 mA constant current in an undivided cell at 23°C for 13.4 h (Q = 2.5 F mol^−1^, Ecell = 1–2 V) with 304 stainless‐steel as electrodes;

^b)^ Isolated yield;

^c)^ Enantioselectivities were determined by chiral HPLC analysis. SS, stainless steel. nr, no reaction.

The use of quinolinox ligand **L10** improved the stereoselectivity of **3a** to 94% along with 72% yield which is much higher than the result (23% yield, 90% ee) reported by Zhou with Mn as reductant (Table 1, entry 5). It may be that manganese powder is difficult to promote the turnover of **L10** coordinated nickel catalysts, while electro‐protocol can easily promote this process by adjusting the reductive potential. Other chiral bisoxazoline and phenol‐oxazoline ligands are not effective (see Supplementary Information). A slight decrease in the stereoselectivity or yield was observed when the reaction was performed under 1.5 mA for 9 h (98%, 86% ee) or 0.5 mA 26.8 h (90%, 92% ee) (Table 1, entries 6–7). In addition, only 25–50% yield of **3a** was obtained with DMF, DMAc, MeCN or DMSO as single solvent (Table 1, entry 8). It's not surprising that this ERC reaction does not occur in the air atmosphere (Table 1, entry 9). Trace amount of product **3a** was obtained when Zn and Ni foam was used in place of SS anode and cathode, respectively (Table 1, entry 10). Interestingly, when 4‐iodotoluene was used to replace 4‐bromotoluene, the reaction equipped with Zn anode and Ni foam cathode under 10 mA afforded the desired product **3a** in 75% yield and 92% ee in 2 hours (Table 1, entry 11). While using iron electrode as anode, the reaction affords the similar yield and ee compared to SS anode (Table 1, entry 12). Considering the iron electrodes are prone to corrosion, the stainless‐steel electrodes are employed as anode in this protocol. To our delight, the desired product **3a** was efficiently synthesized (86%, 92% ee) after 6.7 hours of electrolysis when the reaction was carried out at 0.1 mmol scale (Table 1, entry 13). Those results further demonstrate the efficiency of electrochemical reduction compared to metal reductant (24 h). Control experiments indicated that the electrical current was necessary for this transformation (Table 1, entry 14).

### 2.2 Evaluation of Substrate Scopes

**Figure 2:**
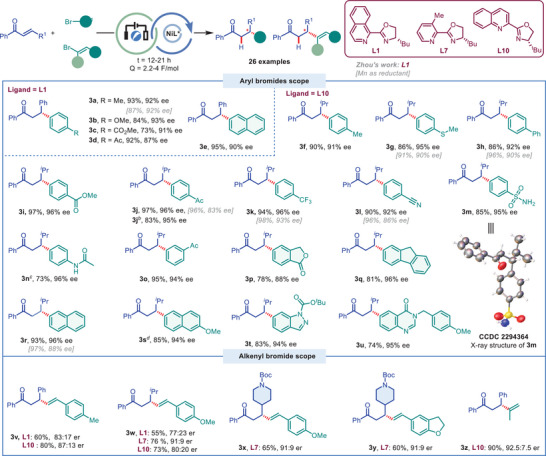
Electrophiles scope of electrochemical Ni‐catalyzed enantioselective hydro‐arylation/alkenylation of enones. a) Conditions: **1** (0.2 mmol), **2** (1.5 equiv), NiBr_2_DME (10 mol.%), Ligand (12 mol.%), DMF/DMSO (1 mL/1 mL), N_2_, 23 °C, SS(+)/SS(‐), *I* = 1 mA, *E*_cell_ = 1–2 V, Q = 2.2‐4 F mol^−1^, t = 12–21 h. b) Aryl triflate was used. c) Aryl iodide was used. d) LiBr (1 eq.) was added. All reported yields are isolated yields. Enantioselectivities were determined by chiral HPLC analysis.

Having established the optimized reaction conditions, we sought to examine the generality of this transformation (**Figure** **2**). First, the scope of aryl bromide was explored with (*E*)‐chalcone **1a** as Michael acceptor. The aryl bromides bearing both electron‐donating groups (EDGs, methyl, methoxyl) or electron‐withdrawing groups (EWGs, ester, acetyl) all gave the corresponding products **3a‐3d** in good to excellent yields and ees. 2‐Bromonaphthalene also afforded the desired product **3e** in 95% yield and 90% ee. The quinox ligand **L10** was chosen as chiral ligand when (*E*)‐enone (**1b**) was employed as Michael acceptor. Considerable improvement of product yields and ees were achieved by the use of quinox ligand **L10** under our electro‐conditions compared to Zhou's report. A wide range of aryl bromides bearing both EDGs (methyl **3f**, methylthio **3g**, phenyl **3h**, acetamide **3n**) and EWGs substituents (ester **3i**, acetyl **3j, 3o**, trifluoromethyl **3k**, cyno **3l** and sulfonamide **3m** etc.) reacted smoothly to afford the desired products in 73−97% yields and 91–96% ees. The absolute stereochemistry of compound **3m** was unambiguously confirmed by X‐ray diffraction analysis, and the configuration of all other products was assigned by analogy. Notably, aryl iodides, aryl triflates can be employed as electrophilic reagents and provided the desired products (**3n**, **3j**). The *meta*‐substituted aryl bromide also gave excellent yield and ee (**3o**). While *ortho*‐substituted hindered aryl bromides afforded the corresponding products in lower ees than *meta*‐ or *para*‐substituted aryl bromides (See Figure S8, Supporting Information). Aryl bromides with dihydroisobenzofuran, fluorenyl and naphthyl motif all gave the corresponding products excellent yields and ees (**3p**, **3q**, **3r**). The naphthyl bromide with methoxyl group also gave the corresponding product **3s** in 85% yield and 94% ee with the addition of lithium bromide. It is noteworthy that the reactions with nitrogen‐containing heterocyclic bromides as substrates all resulted in the satisfied yields and ees (**3t, 3u**). In addition, the yield and ees of β‐arylated ketones synthesized by employing **L10** as ligand were compared with those reported by Zhou's method. Generally, the electro‐reduction protocol gave higher ee (**3g**, **3j, 3k, 3l, 3r**) with similar yields compared to the protocol employed with metal reductant when the aryl bromides contain methylthio, acetyl, trifluoromethyl, cyan, and naphthyl group were used as substrates, respectively. Furthermore, we also demonstrated that alkenyl bromides, such as β‐aryl vinyl bromide and 2‐bromopropene, can be employed as electrophiles to give the desired products **3v‐3z** in moderate to good yields and ees with either isoquinox **L1**, 3‐methyl‐pyridine ligands **L7,** quinolinox **L10** as ligand, respectively.

**Figure 3:**
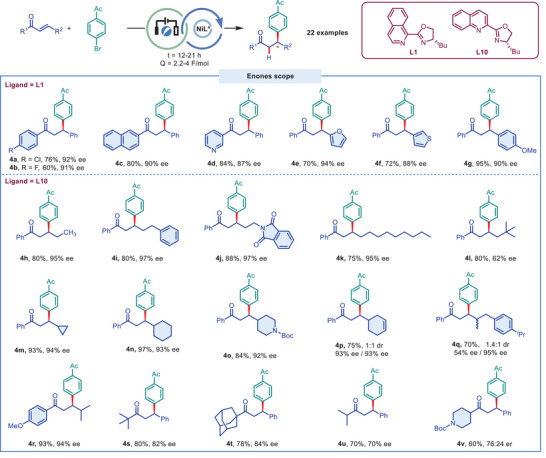
Enones scope of electrochemical Ni‐catalyzed enantioselective hydro‐arylation of enones. a) Conditions: **1** (0.2 mmol), **2f** (1.5 equiv), NiBr_2_DME (10 mol.%), Ligand (12 mol.%), DMF/DMSO (1 mL/1 mL), N_2_, 23 °C, SS(+)/SS(‐), *I* = 1 mA, *E*_cell_ = 1–2 V, Q = 2.2–4 F mol^−1^, t = 12–21 h. All reported yields are isolated yields. Enantioselectivities were determined by chiral HPLC analysis.

The scope of enones with different substituents on the carbonyl and alkene sides were then investigated (**Figure** **3**). The aryl ketones bearing chloro (**4a**), fluoro (**4b**) or methoxy group (**4r**), the naphthyl (**4c**) or pyridinyl (**4d**) ketones all reacted well and gave the corresponding products in good to excellent yields and ees. Compared to Zhou's relatively limited enone substrates with only Ph or *^i^* Pr on the alkene, we demonstrated that a wide range of enones with various aryl (**4e‐4g**) or alkyl substitutes (**4h‐4v**) reacted well under the optimum conditions. For example, ethyl (**4h**), phenylethyl (**4i**), protected amine (**4j**), nonyl (**4k**), cyclopropyl (**4m**), cyclohexyl (**4n**), and *N*‐Boc piperidyl (**4o**) were all tolerated and gave the corresponding products in excellent yields and ees. However, the enantioselectivity of product **4l** was decreased significantly when there is bulky group on the alkene side. A mixture of roughly 1:1 diastereoisomers (**4p** and **4q)** were obtained in good yields with good stereocontrol. In addition, the hindered alkyl ketone substrates, such as containing tert‐butyl and adamantly group, reacted well and afforded the desired products **4s** and **4t** in good yields and ees. However, the reaction provided the hydro‐arylated products **4u** and **4v** in moderate yields and inferior ees. Racemic products were produced in poor yield when the cyclic enones were employed as coupling partners (See Figure S8, Supporting Information).

### 2.3 Synthetic Applications

**Figure 4:**
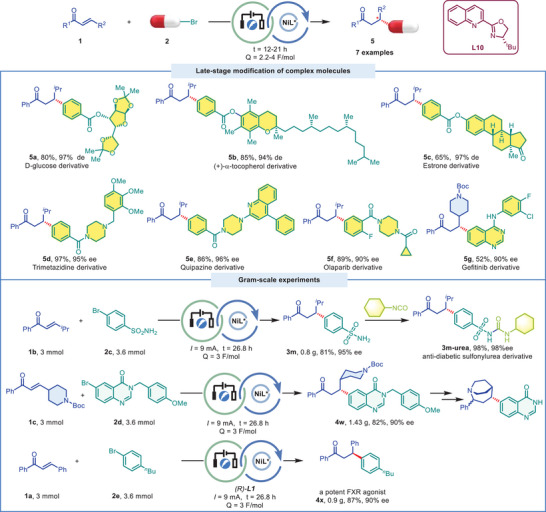
Synthetic Applications.

To demonstrate the synthetic utility of this asymmetric ERC reaction, we set out to apply this protocol to more structurally complex reaction partners featuring motifs commonly found in natural products and pharmaceutically active molecules (**Figure** **4**). Complex substrates bearing preexisted stereocenters were also compatible without loss of existing stereochemical property. Diacetone‐D‐glucose, tocopherol, and estrone derivatives were tolerated well, furnishing the corresponding chiral products in 80%, 85%, 65% yields with 97%, 94%, 97% de, respectively (**5a**‐**5c**). Trimetazidine and quipazine derived bromides reacted with enone **1b** to give the desired products in 97%, 86% yield with 95%, 96% ee, respectively (**5d**, **5e**). Olaparib and Gefitinib derivatives were also applied in the reaction delivering adducts **5f** and **5g** in 89%, 52% yield and 90%, 90% ee, respectively.

To verify the robustness of this electrochemistry, gram‐scale reactions were conducted and the corresponding highly functionalized products **3m** (sulfonamide) and **4w** (*N*‐Boc piperidine with quinazolinone) were readily obtained in good yields and excellent ees which could be further transformed into biologically active molecules (Figure 4).^[^ ^67^, ^68^, ^69^ ^]^ For example, the sulfonamide **3m** could be transformed into anti‐diabetic sulfonylurea derivative **3m‐urea** in 98% yield and 98% ee, which demonstrated the scalability of this powerful protocol for the synthesis of bioactive compounds. And compound **4x**, a potent FXR (farnesoid X receptor) agonist,^[^ ^70^ ^]^ can be prepared efficiently on gram‐scale from chalcone **1a** with 1‐bromo‐4‐butylbenzene **2e**.

### 2.4 Mechanism Investigation

**Figure 5:**
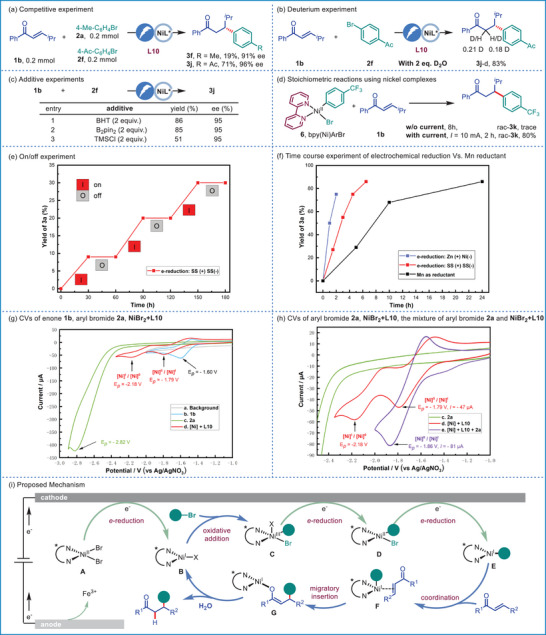
Mechanistic Investigation and Proposed Mechanism.

To elucidate the mechanism of this nickel‐catalyzed asymmetric ERC reaction, additional experiments were conducted, the results of which are summarized in **Figure** **5**. According to literature reports,^[^ ^71^, ^72^, ^73^ ^]^ the competitive experiments with different electronic 4‐substituted bromobenzenes were conducted (Figure 5a). The results show that electron‐deficient aryl bromide reacts much faster than the electron‐rich one. To identify the proton source the related control experiments were conducted (see Table S4, Supporting Information). The yield of product **3j** decreased significantly when the reaction was conducted in anhydrous solvents. In contrast, **3j** was obtained in good yields by adding 1.5‐3.0 equivalents of water into the reaction which suggests that the trace amount of water is beneficial to the reaction. Furthermore, the undeuterated product **3j** was obtained by ^1^H‐NMR when DMSO‐*d*_6_ was used as solvent which indicates the proton is not from the organic solvent (see Supplementary Information). However, the deuterated product **3j**‐*d* was obtained when 2 equivalent of D_2_O was added which indicates the proton is from the water. The result is consistent with Zhou's report (Figure 5b).

When two equivalents of a radical scavenger, such as butylated hydroxytoluene (BHT) or bis(pinacolato)diboron (B_2_Pin_2_) were added, the desired product **3j** was formed in good yield and ee, which indicated radical mechanism might not be involved in this reaction (Figure 5c). And a slight‐decreased yield was observed when two equivalents of chlorotrimethylsilane (TMSCl) was added, which suggests the reaction might proceed via elementary insertion of arylnickel species rather than allylnickel species (Figure 5c).^[^ ^14^ ^]^ To clarify whether the migration insertion mediated by [Ni]^II^ species in the reaction, we were prepared arylnickel^II^ complex **6** ^[^ ^74^ ^]^ and subjected it to stoichiometric reactions with 2 equiv of enone **1b** (Figure 5d). Not surprisingly, trace desired product rac‐**3k** was detected in the absence of electricity. In comparison, rac‐**3k** was produced in 80% yield with the current at 10 mA after 2 h, indicating that electro‐reduction was essential for aryl transfer.

In addition, the crucial role of electricity in this transformation has been proven through on/off experiments (Figure 5e). Finally, the reaction proceeded more efficiently under electrochemical conditions, including Zn (Table 1, entry 11) and SS anode, than manganese powder enabled nickel catalyzed protocol, which further demonstrates the relative efficacy of electrochemistry (Figure 5f).

To gain insights into the reaction mechanism, a series of cyclic voltammetric (CV) analyses were conducted (Figure 5g–h). The enone **1b** exhibits a reversible reductive peak at −1.60 V versus Ag/AgNO_3_ in DMSO (line b, blue line). And 4‐bromotoluene **2a** exhibits an unreversible reductive peak at −2.82 V versus Ag/AgNO_3_ (line c, green line). The mixture of NiBr_2_•DME and **L10** in a ratio of 1:1 exhibits two quasi‐reversible reductive peaks at −1.79 and −2.18 V versus Ag/AgNO_3_, which may be attributed to the reductive potential of [Ni]^II^/[Ni]^I^ and [Ni]^I^/[Ni]^0^, respectively (line d, red line). Those results were similar to Mei's report.^[^ ^48^ ^]^ Significant increase current in the reduction peak of [Ni]^II^/[Ni]^I^ was observed (E*_p_* = −1.86 V, −81 µA vs E*_p_* = −1.79 V, −47 µA) by the addition of **2a** into the mixture of NiBr_2_•DME and **L10** (line e, purple line) which indicates the oxidative addition of **2a** to [Ni]^I^ species by generating aryl‐[Ni]^III^ species. During the reaction process, the voltage of the reaction is maintained below 2 V. These results demonstrate that the [Ni]^I^/[Ni]^III^ cycle with fast activation of the electrophile by a [Ni]^I^ species was operated in this electro‐reductive reaction rather than metal reductant driven [Ni]^0^/[Ni]^II^ cycle,^[^ ^14^ ^]^ which is consistent with Reisman's report.^[^ ^73^ ^]^

Based on these studies and previous reports,^[^ ^15^, ^16^, ^17^, ^18^, ^71^, ^72^, ^73^ ^]^ a plausible mechanism for the Ni‐catalyzed ERC reaction is presented in Figure 5i. Upon cathodic reduction of the [Ni]^II^ precatalyst **A**, the resulting [Ni]^I^ species **B** rapidly reacts with aryl bromide to give [Ni]^III^ species **C**, which can be reduced to furnish resting state [Ni]^II^ species **D**. The active [Ni]^I^ species **E** could be formed after another cathodic reduction from species **D**, which coordinate with enones to give π‐complex **F**. After migratory insertion and the resulting nickel *O*‐enolate **G** could be hydrolyzed by water to release the final product and [Ni]^I^ species **B** to complete the catalytic process.

## 3 Conclusion

In summary, we have developed an electrochemical Ni‐catalyzed enantioselective reductive conjugate hydro‐arylation/alkenylation of enones with readily available and low‐cost stainless steel electrodes. A wide range of aryl bromides, iodides, triflates, or alkenyl bromides could be employed as electrophiles for the efficient synthesis of valuable β‐arylated or alkylated enones in good to excellent yields and enantioselectivities under mild conditions in a simple manner. This scalable protocol was further applied for the late‐stage modification of bio‐relevant compounds. The success of this reaction relies on the perfect match of electrochemistry with chiral nickel catalysts which avoid the drawbacks by employing the external stoichiometric amount of sensitive metal reductants. Mechanistic studies and CVs illustrated a possible Ni^I^/Ni^III^ cycle is involved in this transformation. Overall, we envisioned this established protocol with green and economic properties would be potentially applicable in organic synthesis and drug discovery.

## 4 Experimental Section

### General Information

All reagents were obtained from commercial suppliers and used without further purification. Yields for all compounds were determined by the column chromatography which was generally performed on silica gel (200‐300 mesh) using petroleum ether (PE)/EtOAc as eluent, and reactions were monitored by thin layer chromatography (TLC) on a glass pate coated with silica gel with fluorescent indicator (GF254) using UV light and iodine chromogenic method. The ^1^H and ^13^C nuclear magnetic resonance (NMR) spectra were recorded on a Bruker Advance 400 MHz (101 MHz) NMR spectrometers using CDCl_3_ as solvent with TMS as internal standard. Chemical shifts are given in ppm (δ) referenced to CDCl_3_ with 7.27 for ^1^H and 77.16 for ^13^C, and to DMSO‐*d*_6_ with 2.50 for ^1^H and 39.52 for ^13^C. Signals are abbreviated as follows: s, singlet; d, doublet; t, triplet; q, quartet; m, multiplet, and coupling constants are expressed in Hz. Chiral HPLC analysis was performed on an Agilent Infinity II 1260 instrument using Daicel Chiralcel columns at 25 °C and a mixture of HPLC‐grade hexanes and isopropanol (ethanol) as eluent. LC/MS analysis was conducted on an Agilent Infinity LC/MSD iQ (1260‐G6160) instrument.Cyclic voltammograms were obtained on a CHI 600E potentiostat. Electrolysis experiments were performed using DJS‐292B or HSPY‐600(30 V/100 mA) as DC power supply.

### General Procedure for the Electrochemical Ni‐Catalyzed Enantioselective Hydro‐Arylation/Alkenylation of Enones

A 10 mL Schlenk tube with a stir bar was charged with NiBr_2_(DME) (6.2 mg, 0.02 mmol, 10 mol.%), (*S*)−4‐(*tert*‐butyl)−2‐(isoquinolin‐1‐yl)−4,5‐dihydrooxazole **L1** (6.1 mg, 0.024 mmol, 12 mol.%), (*S*)−4‐(tert‐butyl)−2‐(3‐methylpyridin‐2‐yl)−4,5‐dihydrooxazole **L7** (5.2 mg, 0.024 mmol, 12 mol.%) or (*S*)−4‐(*tert*‐butyl)−2‐(quinolin‐2‐yl)−4,5‐dihydrooxazole **L10** (6.1 mg, 0.024 mmol, 12 mol.%), enone (0.2 mmol, if solid), electrophile (0.3 mmol, if solid), DMF (1 mL) and DMSO (1 mL). The tube was sealed with rubber septum which was equipped with 304 stainless steel electrodes (1.5 cm x 1 cm, ≈1 cm immersion depth in solution, *S* = 1 cm^2^) as anode and cathode and stirred for 10–20 min at room temperature. It was then evacuated and backfilled with nitrogen for three cycles. The enone or electrophile (if liquid) was added via a syringe. The reaction mixture was electrolyzed under a constant current of 1 mA (*J* = 1 mA cm^−2^, *E*_cell_ = 1–2 V) until the complete consumption of the starting material as judged by TLC or LC‐MS of an aliquot (12–21 h, 2.2–4 F mol^−1^). After the reaction, the electrodes were taken out and rinsed with EtOAc. Aqueous sat. EDTA was then added; the resulting mixture was extracted with EtOAc. The combined organic layer was dried over anhydrous Na_2_SO_4_ and concentrated in vacuo. The crude material was purified by column chromatography to furnish the desired products. The enantioselectivity of the purified product was determined by chiral HPLC analysis using Daicel Chiralcel columns. The racemic sample was prepared via a similar procedure using 10 mol.% NiBr_2_(bpy)_3_ as catalyst, LiBr as electrolyte, Zn anode, Ni foam cathode with current at 10 mA, 1.8 h.

Full experimental details and characterization of new compounds can be found in the Supplementary Information.

[CCDC 2294364 contains the supplementary crystallographic data for this paper. These data can be obtained free of charge from The Cambridge Crystallographic Data Centre via www.ccdc.cam.ac.uk/data_request/cif

## Conflict of Interest

The authors declare no conflict of interest.

## Author Contributions

Z.Y. and F.Z. conceived and designed the study and wrote the manuscript. Z.Y. performed the experiments, mechanistic studies, analyzed the data. W.M., X.Z., and H.L., synthesized some of the substrates and ligands.

## Supporting information



Supporting Information

## Data Availability

The data that support the findings of this study are available in the supplementary material of this article.

## References

[1] T. Hayashi , K. Yamasaki , Chem. Rev. 2003, 103, 2829. 12914482 10.1021/cr020022z

[2] H. J. Edwards , J. D. Hargrave , S. D. Penrose , C. G. Frost , Chem. Soc. Rev. 2010, 39, 2093. 20407730 10.1039/b919762c

[3] F. López , A. J. Minnaard , B. L. Feringa , Acc. Chem. Res. 2006, 40, 179. 10.1021/ar0501976 17370989

[4] A. Alexakis , J. E. Bäckvall , N. Krause , O. Pàmies , M. Diéguez , Chem. Rev. 2008, 108, 2796. 18671436 10.1021/cr0683515

[5] S. R. Harutyunyan , T. den Hartog , K. Geurts , A. J. Minnaard , B. L. Feringa , Chem. Rev. 2008, 108, 2824. 18698733 10.1021/cr068424k

[6] S. Z. Tasker , E. A. Standley , T. F. Jamison , Nature 2014, 509, 299. 24828188 10.1038/nature13274 PMC4344729

[7] L. Nattmann , R. Saeb , N. Nöthling , J. Cornella , Nature Catalysis 2020, 3, 6.

[8] C. A. Malapit , M. B. Prater , J. R. Cabrera‐Pardo , M. Li , T. D. Pham , T. P. McFadden , S. Blank , S. D. Minteer , Chem. Rev. 2022, 122, 3180. 34797053 10.1021/acs.chemrev.1c00614 PMC9714963

[9] T. B. Hamby , M. J. LaLama , C. S. Sevov , Science 2022, 376, 410. 35446658 10.1126/science.abo0039 PMC9260526

[10] G. P. Boldrini , D. Savoia , E. Tagliavini , C. Trombini , A. U. Ronchi , J. Organomet. Chem. 1986, 301, C62.

[11] S. A. Lebedev , V. S. Lopatina , E. S. Petrov , I. P. Beletskaya , J. Organomet. Chem. 1988, 344, 253.

[12] R. Sustmann , P. Hopp , P. Holl , Tetrahedron Lett 1989, 30, 689.

[13] S. Condon‐Gueugnot , E. Leonel , J.‐Y. Nedelec , J. Perichon , J. Org. Chem. 1995, 60, 7684.

[14] R. Shrestha , S. C. M. Dorn , D. J. Weix , J. Am. Chem. Soc. 2013, 135, 751. 23270480 10.1021/ja309176h PMC3547151

[15] L. Zhang , M. Zhao , M. Pu , Z. Ma , J. Zhou , C. Chen , Y.‐D. Wu , Y. Robin Chi , J. S. Zhou , J. Am. Chem. Soc. 2022, 144, 20249. 36315074 10.1021/jacs.2c05678

[16] L. Zhang , X. Wang , M. Pu , C. Chen , P. Yang , Y.‐D. Wu , Y. Robin Chi , J. S. Zhou , J. Am. Chem. Soc. 2023, 145, 8498. 10.1021/jacs.3c00548 37023358

[17] M. Zhao , L. Zhang , J. S. Zhou , ACS Catal 2024, 14, 6228.

[18] M. Zhao , W. Xu , Y.‐D. Wu , X. Yang , J. Wang , J. S. Zhou , J. Am. Chem. Soc. 2024, 146, 20477. 38982945 10.1021/jacs.4c06735

[19] Q. Lin , T. Diao , J. Am. Chem. Soc. 2019, 141, 17937. 31589820 10.1021/jacs.9b10026 PMC7058187

[20] Q. Lin , Y. Fu , P. Liu , T. Diao , J. Am. Chem. Soc. 2021, 143, 14196. 34432468 10.1021/jacs.1c05255 PMC8820414

[21] M. Yan , Y. Kawamata , P. S. Baran , Chem. Rev. 2017, 117, 13230. 28991454 10.1021/acs.chemrev.7b00397 PMC5786875

[22] Y. Yuan , A. Lei , Acc. Chem. Res. 2019, 52, 3309. 31774271 10.1021/acs.accounts.9b00512

[23] P. Xiong , H.‐C. Xu , Acc. Chem. Res. 2019, 52, 3339. 31774646 10.1021/acs.accounts.9b00472

[24] J. L. Röckl , D. Pollok , R. Franke , S. R. Waldvogel , Acc. Chem. Res. 2020, 53, 45. 31850730 10.1021/acs.accounts.9b00511

[25] J. C. Siu , N. Fu , S. Lin , Acc. Chem. Res. 2020, 53, 547. 32077681 10.1021/acs.accounts.9b00529 PMC7245362

[26] H. Wang , K. Liang , W. Xiong , S. Samanta , W. Li , A. Lei , Sci. Adv. 2020, 6, eaaz0590. 32440542 10.1126/sciadv.aaz0590 PMC7228760

[27] C. Xu , B. Ma , Z. Gao , X. Dong , C. Zhao , H. Liu , Sci. Adv. 2021, 7, eabk0100. 34767438 10.1126/sciadv.abk0100 PMC8589306

[28] B. Li , H. Ge , Sci. Adv. 2019, 5, eaaw2774. 31139749 10.1126/sciadv.aaw2774 PMC6534392

[29] Y. Yuan , Y. Chen , S. Tang , Z. Huang , A. Lei , Sci. Adv. 2018, 4, eaat5312. 30083610 10.1126/sciadv.aat5312 PMC6070360

[30] W. Zhang , L. Lu , W. Zhang , Y. Wang , S. D. Ware , J. Mondragon , J. Rein , N. Strotman , D. Lehnherr , K. A. See , S. Lin , Nature 2022, 604, 292. 35189623 10.1038/s41586-022-04540-4 PMC9016776

[31] C. Zhu , H. Yue , P. Nikolaienko , M. Rueping , CCS Chem. 2020, 2, 179.

[32] Q. Lin , L. Li , S. Luo , Chem. Eur. J. 2019, 25, 10033. 31026120 10.1002/chem.201901284

[33] K. Liang , Q. Zhang , C. Guo , Sci. Adv. 2022, 8, eadd7134. 36351023 10.1126/sciadv.add7134 PMC9645727

[34] X. Chang , Q. Zhang , C. Guo , Angew. Chem. Int. Ed. 2020, 59, 12612. 10.1002/anie.202000016 32057174

[35] N. Fu , L. Song , J. Liu , Y. Shen , J. C. Siu , S. Lin , J. Am. Chem. Soc. 2019, 141, 14480. 31498595 10.1021/jacs.9b03296 PMC7023682

[36] Q. Zhang , X. Chang , L. Peng , C. Guo , Angew. Chem. Int. Ed. 2019, 58, 6999. 10.1002/anie.201901801 30908778

[37] X. Huang , Q. Zhang , J. Lin , K. Harms , E. Meggers , Nat. Catal. 2018, 2, 34.

[38] T. von Münchow , S. Dana , Y. Xu , B. Yuan , L. Ackermann , Science 2023, 379, 1036. 36893225 10.1126/science.adg2866

[39] X. Chang , J. Zhang , Q. Zhang , C. Guo , Angew. Chem. Int. Ed. 2020, 59, 18500. 10.1002/anie.202006903 32652737

[40] L. Li , Y. Li , N. Fu , L. Zhang , S. Luo , Angew. Chem. Int. Ed. 2020, 59, 14347. 10.1002/anie.202006016 32506841

[41] Z.‐H. Wang , P.‐S. Gao , X. Wang , J.‐Q. Gao , X.‐T. Xu , Z. He , C. Ma , T.‐S. Mei , J. Am. Chem. Soc. 2021, 143, 15599. 34533943 10.1021/jacs.1c08671

[42] Q. Zhang , K. Liang , C. Guo , Angew. Chem. Int. Ed. 2022, 61, e202210632. 10.1002/anie.202210632 35912822

[43] P. Xiong , M. Hemming , S. I. Ivlev , E. Meggers , J. Am. Chem. Soc. 2022, 144, 6964. 35385651 10.1021/jacs.2c01686

[44] X. Tan , Q. Wang , J. Sun , Nat. Commun. 2023, 14, 357. 36690612 10.1038/s41467-023-36000-6 PMC9870882

[45] J. Rein , S. B. Zacate , K. Mao , S. Lin , Chem. Soc. Rev. 2023, 52, 8106. 37910160 10.1039/d3cs00511a PMC10842033

[46] M. Durandetti , J. Périchon , J.‐Y. Nédélec , J. Org. Chem. 1997, 62, 7914. 11671890 10.1021/jo971279d

[47] T. J. DeLano , S. E. Reisman , ACS Catal. 2019, 9, 6751. 32351776 10.1021/acscatal.9b01785 PMC7190267

[48] H. Qiu , B. Shuai , Y.‐Z. Wang , D. Liu , Y.‐G. Chen , P.‐S. Gao , H.‐X. Ma , S. Chen , T.‐S. Mei , J. Am. Chem. Soc. 2020, 142, 9872. 32392046 10.1021/jacs.9b13117

[49] Y. Gao , D. E. Hill , W. Hao , B. J. McNicholas , J. C. Vantourout , R. G. Hadt , S. E. Reisman , D. G. Blackmond , P. S. Baran , J. Am. Chem. Soc. 2021, 143, 9478. 34128671 10.1021/jacs.1c03007 PMC8720499

[50] W. Ding , M. Li , J. Fan , X. Cheng , Nat. Commun. 2022, 13, 5642. 36163325 10.1038/s41467-022-33452-0 PMC9512896

[51] D. Liu , Z.‐R. Liu , Z.‐H. Wang , C. Ma , S. Herbert , H. Schirok , T.‐S. Mei , Nat. Commun. 2022, 13, 7318. 36443306 10.1038/s41467-022-35073-z PMC9705544

[52] X. Hu , I. Cheng‐Sánchez , S. Cuesta‐Galisteo , C. Nevado , J. Am. Chem. Soc. 2023, 145, 6270. 36881734 10.1021/jacs.2c12869 PMC10037331

[53] Y.‐Z. Wang , Z.‐H. Wang , I. L. Eshel , B. Sun , D. Liu , Y.‐C. Gu , A. Milo , T.‐S. Mei , Nat. Commun. 2023, 14, 2322. 37087477 10.1038/s41467-023-37965-0 PMC10122672

[54] Z. Ye , Y. Wu , N. Chen , H. Zhang , K. Zhu , M. Ding , M. Liu , Y. Li , F. Zhang , Nat. Commun. 2020, 11, 3628. 32686668 10.1038/s41467-020-17389-w PMC7371640

[55] Z. Ye , M. Ding , Y. Wu , Y. Li , W. Hua , F. Zhang , Green Chem. 2018, 20, 1732.

[56] Z. Ye , F. Wang , Y. Li , F. Zhang , Green Chem 2018, 20, 5271.

[57] Y. Li , Z. Ye , N. Chen , Z. Chen , F. Zhang , Green Chem. 2019, 21, 4035.

[58] Z. Ye , R. Zhu , F. Wang , H. Jiang , F. Zhang , Org. Lett. 2021, 23, 8240. 34697944 10.1021/acs.orglett.1c02991

[59] H. Zhang , Z. Ye , N. Chen , Z. Chen , F. Zhang , Green Chem 2022, 24, 1463.

[60] N. Chen , Z. Ye , F. Zhang , Org. Biomol. Chem. 2021, 19, 5501. 34079974 10.1039/d1ob00420d

[61] Z. Ye , X. Zhang , W. Ma , F. Zhang , Green Chem. 2023, 25, 2524.

[62] J. Berthelot , C. Guette , F. Fournier , D. Davoust , Tetrahedron Lett. 1987, 28, 1881.

[63] A. Cabrera , R. L. Lagadec , P. Sharma , J. L. Arias , R. A. Toscano , L. Velasco , R. Gaviño , C. Alvarez , M. Salmón , J. Chem. Soc., Perkin Trans. 1 1998, 21, 3609.

[64] S. Condon , D. Dupré , I. Lachaise , J.‐Y. Nédélec , Synthesis 2002, 12, 1752.

[65] S. Condon , D. Dupré , G. Falgayrac , J.‐Y. Nédélec , Eur. J. Org. Chem. 2002, 1, 105.

[66] S. Condon , J.‐Y. Nédélec , Synthesis 2004, 18, 3070.

[67] X. Jiang , K. Wu , R. Bai , P. Zhang , Y. Zhang , Eur. J. Med. Chem. 2022, 229, 114085. 34998058 10.1016/j.ejmech.2021.114085

[68] H. Li , G. Fu , W. Zhong , Eur. J. Med. Chem. 2023, 245, 114915. 36375335 10.1016/j.ejmech.2022.114915

[69] N. Kerru , A. Singh‐Pillay , P. Awolade , P. Singh , Eur. J. Med. Chem. 2018, 152, 436. 29751237 10.1016/j.ejmech.2018.04.061

[70] D. Schuster , P. Markt , U. Grienke , J. Mihaly‐Bison , M. Binder , S. M. Noha , J. M. Rollinger , H. Stuppner , V. N. Bochkov , G. Wolber , Bioorg. Med. Chem. 2011, 19, 7168. 22018919 10.1016/j.bmc.2011.09.056 PMC3254253

[71] S. I. Ting , W. L. Williams , A. G. Doyle , J. Am. Chem. Soc. 2022, 144, 5575. 35298885 10.1021/jacs.2c00462

[72] Y.‐Z. Wang , B. Sun , X.‐Y. Zhu , Y.‐C. Gu , C. Ma , T.‐S. Mei , J. Am. Chem. Soc. 2023, 145, 23910. 37883710 10.1021/jacs.3c10109

[73] R. F. Turro , J. L. H. Wahlman , Z. J. Tong , X. Chen , M. Yang , E. P. Chen , X. Hong , R. G. Hadt , K. N. Houk , Y.‐F. Yang , S. E. Reisman , J. Am. Chem. Soc. 2023, 145, 14705. 37358565 10.1021/jacs.3c02649 PMC10347553

[74] S. Maiti , S. Roy , P. Ghosh , A. Kasera , D. Maiti , Angew. Chem. Int. Ed. 2022, 61, e202207472. 10.1002/anie.202207472 35929544

