# Supplementary material for: Electrochemically Driven Nickel‐Catalyzed Enantioselective Hydro‐Arylation/Alkenylation of Enones
Source: Adv Sci (Weinh). 2024 Sep 12;11(42):2405926. doi: 10.1002/advs.202405926 (PMC11558104; doi:10.1002/advs.202405926)
Supplement: Supplementary file 1 — Supporting Information [file ADVS-11-2405926-s001.docx]

Supporting Information

**Electrochemically driven Nickel-Catalyzed Enantioselective Hydro-Arylation/Alkenylation of Enones**

*Zenghui Ye, Weiyuan Ma, Xi Zhang, Huaqing Liu, Fengzhi Zhang**

**Affiliations:**

School of Pharmacy, Hangzhou Medical College, Hangzhou, Zhejiang, 311399, China. zhangfengzhi@hmc.edu.cn

**Table of Contents**

**1. General Information 1**

**2. Condition Optimization 2**

**3. General Procedure for the Electrochemical Ni-catalyzed Enantioselective Hydro-Arylation/Alkenylation of Enones 4**

**3.1 Procedure for 0.2 mmol Scale Synthesis 4**

**3.2 Gram-scale Experiments 5**

**4. Synthetic Applications 6**

**5. Mechanistic Investigation 7**

**5.1 Competitive Experiment 7**

**5.2 Water additive Experiments 8**

**5.3 Deuterium Experiment 8**

**5.4 Additive Experiments 10**

**5.5 Stoichiometric Reactions 10**

**5.6 On/off experiment 11**

**5.7 Time course experiment of electrochemical reduction Vs. Mn reductant 12**

**5.8 Weight variation of electrodes 12**

**5.9 Cyclic Voltammetry Studies 13**

**5.10 Comparison of electrochemical and nonelectrochemical conditions 14**

**6. Failed Examples 14**

**7. Characterization Data for the Products 15**

**8. X-Ray Crystallographic Data of 3m 41**

**9. Reference 42**

**10. NMR Spectra 43**

**11. HPLC Spectra 100**

**1. General Information**

All reagents were obtained from commercial suppliers and used without further purification. Yields for all compounds were determined by the column chromatography which was generally performed on silica gel (200-300 mesh) using petroleum ether (PE)/EtOAc as eluent, and reactions were monitored by thin layer chromatography (TLC) on a glass pate coated with silica gel with fluorescent indicator (GF254) using UV light and iodine chromogenic method. The ^1^H and ^13^C nuclear magnetic resonance (NMR) spectra were recorded on a Bruker Advance 400 MHz NMR spectrometers using CDCl_3_ as solvent with TMS as internal standard. Chemical shifts are given in ppm (δ) referenced to CDCl_3_ with 7.27 for ^1^H and 77.16 for ^13^C, and to DMSO-*d*_6_ with 2.50 for ^1^H and 39.52 for ^13^C. Signals are abbreviated as follows: s, singlet; d, doublet; t, triplet; q, quartet; m, multiplet, and coupling constants are expressed in Hz.

Chiral HPLC analysis was performed on an Agilent Infinity II 1260 instrument using Daicel Chiralcel columns at 25 °C and a mixture of HPLC-grade hexanes and isopropanol (ethanol) as eluent. LC/MS analysis was conducted on an Agilent Infinity LC/MSD iQ (1260-G6160) instrument.

Cyclic voltammograms were obtained on a CHI 600E potentiostat. Electrolysis experiments were performed using DJS-292B or HSPY-600(30 V/100 mA) as DC power supply.

Enones were prepared according to the previously reported procedure (Claisen-Schmidt condensation and Wittig reaction).^1^

**2. Condition Optimization**

**Table S1. Screening of Ligand.**

| **Entry*^a^*** | **Ligand** | **Yield%*^b^*** | **ee%*^c^*** |
| --- | --- | --- | --- |
| 1 | **L1** | 93 | 92 |
| 2 | **L2** | 50 | 78 |
| 3 | **L3** | 43 | 70 |
| 4 | **L4** | 46 | 68 |
| 5 | **L5** | 47 | 74 |
| 6 | **L6** | 53 | 85 |
| 7 | **L7** | 66 | 82 |
| 8 | **L8** | 54 | 60 |
| 9 | **L9** | 55 | 70 |
| 10 | **L10** | 72 | 94 |
| 11 | **L11** | 75 | 84 |
| 12 | **L12** | 56 | 66 |
| 13 | **L13** | 81 | 76 |
| 14 | **L14** | ND | -- |
| 15 | **L15** | ND | -- |
| [a] Reaction conditions (unless otherwise specified): **1a** (0.2 mmol), **2a** (0.3 mmol, 1.5 equiv), NiBr_2_DME (10 mmol%), Ligand (12 mmol%), stainless steel electrodes, DMF/DMSO (1 mL/1 mL), *I* = 1 mA, Q = 2.5 F/mol, t = 13.4 h. [b] Isolated yield. [c] Enantioselectivities were determined by chiral HPLC analysis. nd, not detected. | | | |

**Table S2. Screening of Electrical Current, Temperature and Solvent.**

| **Entry*^a^*** | **Solvent** | **Temp.(°C)** | ***I* (mA)** | **Q (F/mol)** | **t (h)** | **Yield%*^b^*** | **ee%*^c^*** |
| --- | --- | --- | --- | --- | --- | --- | --- |
| 1 | DMF/DMSO | 23 | 1.5 | 2.5 | 9 | 98 | 85 |
| 2 | DMF/DMSO | 23 | 0.5 | 2.5 | 26.8 | 90 | 92 |
| 3 | DMF/DMSO | 23 | 1 | 2.5 | 13.4 | 93 | 92 |
| 4 | DMF/DMSO | 0 | 1 | 2.5 | 13.4 | trace | -- |
| 5 | DMF | 23 | 1 | 2.5 | 13.4 | 50 | 92 |
| 6 | DMAc | 23 | 1 | 2.5 | 13.4 | 37 | 92 |
| 7 | MeCN | 23 | 1 | 2.5 | 13.4 | 25 | 91 |
| 8 | DMSO | 23 | 1 | 2.5 | 13.4 | 45 | 92 |
| 9*^d^* | DMF/DMSO | 23 | 1 | 2.5 | 13.4 | 93 | 86 |
| 10*^e^* | DMF/DMSO | 23 | 1 | 2.5 | 13.4 | nr | -- |
| 11*^f^* | DMF/DMSO | 23 | 1 | 2.5 | 6.7 | 86 | 92 |
| [a] Reaction conditions (unless otherwise specified): **1a** (0.2 mmol), **2a** (0.3 mmol, 1.5 equiv), NiBr_2_DME (10 mmol%), **L1** (12 mmol%), stainless steel electrodes, solvent (2 mL). [b] Isolated yield. [c] Enantioselectivities were determined by chiral HPLC analysis. [d] NiBr_2_DME (5 mmol%), **L1** (6 mmol%). [e] Air atmosphere. [f] 0.1 mmol scale. nr, no reaction. | | | | | | | |

**Table S3. Screening of electrodes with different electrophilic reagents.**

| **Entry*^a^*** | **Anode** | **Cathode** | **electrophilic reagent** | **Yield%*^b^*** | **ee%*^c^*** |
| --- | --- | --- | --- | --- | --- |
| 1 | Zn | Ni foam | **2a** | trace | -- |
| 2 | Zn | Ni foam | *p*-Tol-I instead of **2a** | 75 | 92 |
| 3 | Fe | Ni foam | **2a**, *I* = 1 mA, t = 13.4 h, Q = 2.5 F/mol | 92 | 92 |
| [a] Reaction conditions (unless otherwise specified): **1a** (0.2 mmol), electrophilic reagent (0.3 mmol, 1.5 equiv), NiBr_2_DME (10 mmol%), **L1** (12 mmol%), electrodes, DMF/DMSO (1 mL/1 mL). [b] Isolated yield. [c] Enantioselectivities were determined by chiral HPLC analysis. | | | | | |

**3. General Procedure for the Electrochemical Ni-catalyzed Enantioselective Hydro-Arylation/Alkenylation of Enones**

**3.1 Procedure for 0.2 mmol Scale Synthesis**

A 10 mL Schlenk tube with a stir bar was charged with NiBr_2_(DME) (6.2 mg, 0.02 mmol, 10 mol%), (*S*)-4-(*tert*-butyl)-2-(isoquinolin-1-yl)-4,5-dihydrooxazole **L1** (6.1 mg, 0.024 mmol, 12 mol%), (*S*)-4-(tert-butyl)-2-(3-methylpyridin-2-yl)-4,5-dihydrooxazole **L7** (5.2 mg, 0.024 mmol, 12 mol%) or (*S*)-4-(*tert*-butyl)-2-(quinolin-2-yl)-4,5-dihydrooxazole **L10** (6.1 mg, 0.024 mmol, 12 mol%), enone (0.2 mmol, if solid), electrophile (0.3 mmol, if solid), DMF (1 mL) and DMSO (1 mL). The tube was sealed with rubber septum which equipped with 304 stainless steel electrodes (1.5 cm x 1 cm, about 1 cm immersion depth in solution, *S* = 1 cm^2^) as anode and cathode and stirred for 10-20 min at room temperature. It was then evacuated, and backfilled with nitrogen for three cycles. The enone or electrophile (if liquid) was added via a syringe. The reaction mixture was electrolyzed under a constant current of 1 mA (*J* = 1 mA / cm^2^, *E*_cell_ = 1~2 V) until the complete consumption of the starting material as judged by TLC or LC-MS of an aliquot (12~21 h, 2.2~4 F/mol). After the reaction, the electrodes were taken out and rinsed with EtOAc. Aqueous sat. EDTA was then added; the resulting mixture was extracted with EtOAc. The combined organic layer was dried over anhydrous Na_2_SO_4_ and concentrated in vacuo. The crude material was purified by column chromatography to furnish the desired products. The enantioselectivity of the purified product was determined by chiral HPLC analysis using Daicel Chiralcel columns.

The racemic sample was prepared via a similar procedure using 10 mol% NiBr_2_(bpy)_3_ as catalyst, LiBr as electrolyte, Zn anode, Ni foam cathode with current at 10 mA, 1.8 h.

**3.2 Gram-scale Experiments**

**Procedure for 3 mmol scale synthesis**: A 25 mL Schlenk tube with a stir bar was charged with NiBr_2_(DME) (92.4 mg, 0.3 mmol, 10 mol%), (*S*)-4-(*tert*-butyl)-2-(quinolin-2-yl)-4,5-dihydrooxazole **L10** (91.5 mg, 0.36 mmol, 12 mol%), enone **1a**, **1b** or **1c** (3 mmol), aryl halide **2c,** **2d** or **2e** (3.6 mmol, 1.2 equiv.), DMF (7.5 mL) and DMSO (7.5 mL). The tube was sealed with rubber septum which equipped with stainless steel electrodes (1.5 cm x 6.5 cm, about 6 cm immersion depth in solution, *S* = 9 cm^2^) as anode and cathode and stirred for 20 min at room temperature. It was then evacuated, and backfilled with nitrogen for three cycles. The reaction mixture was electrolyzed under a constant current of 9 mA (*J* = 1 mA / cm^2^) until the complete consumption of the starting material as judged by TLC or LC-MS of an aliquot (~26.8 h, 3 F/mol). After the reaction, the electrodes were taken out and rinsed with EtOAc. Aqueous sat. EDTA was then added, the resulting mixture was extracted with EtOAc. The combined organic layer was dried over anhydrous Na_2_SO_4_ and concentrated in vacuo. The crude material was purified by column chromatography to furnish the desired products **3m**, **4w** or **4x**.

**Reduction of nickel catalyst loading:**

Lower the nickel catalyst loading to 1 mol%, the reaction affords the similar yield (80%) and lower ee (82%) in 3 mmol scale.


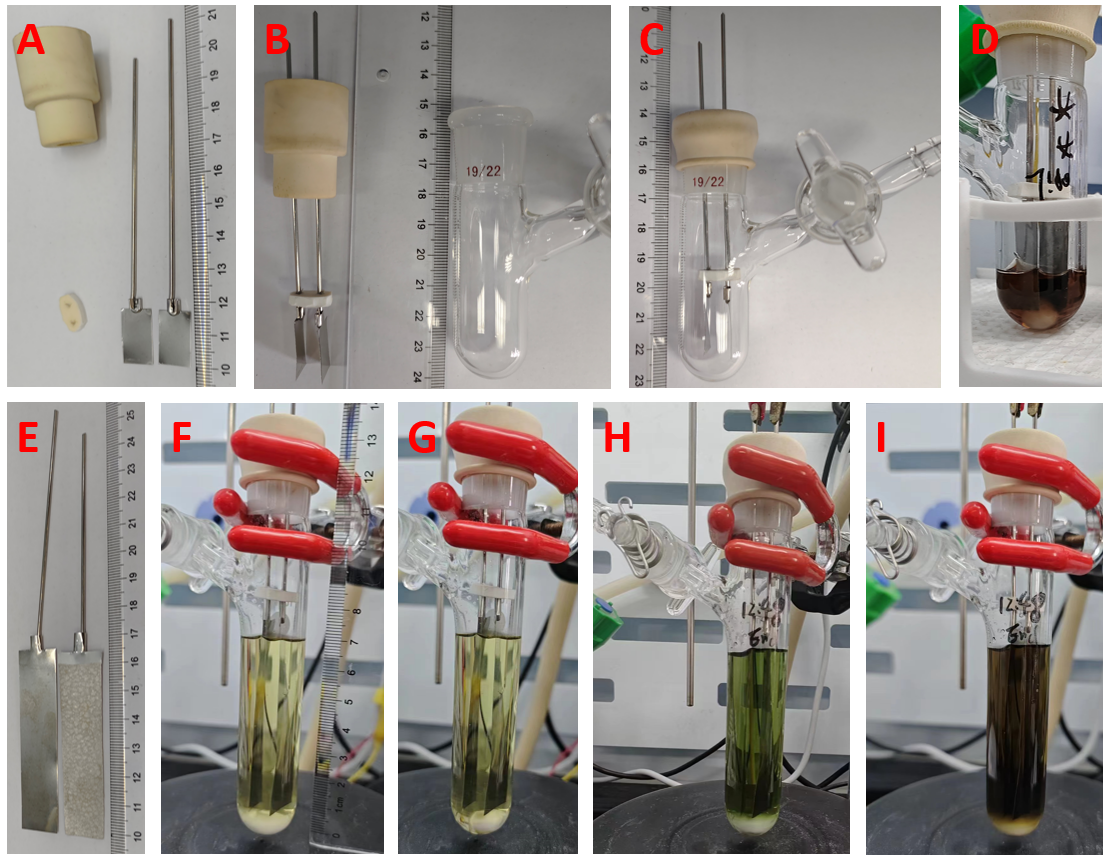
**Figure S1. Electrolysis setup**. **A-D**: 0.2 mmol scale reaction setup; **E-I**: Gram-scale setup: **F&G**: 0 min; **H**: after 10min; **I**: after 1 hour.

**4. Synthetic Applications**

The **3m** (0.5 mmol, 165 mg) was refluxed in a stirred solution of anhydrous potassium carbonate (1.5 mmol, 207 mg) in acetone (3 ml) for 1.5 h under dry conditions. After cool to room temperature, cyclohexylisocyanate (0.8 mmol, 100 mg) was added dropwise. The resultant mixture was refluxed for another 16 h. Acetone was removed under vacuum and water (10 ml) was added to the white residue. The mixture was acidified with concentrated hydrochloric acid to pH 1-2. White precipitate was filtered to provide the **3m-urea**.

Follow the general procedure for the electrochemical asymmetric arylation of enones, the complex natural products and pharmaceuticals derived bromides were used as electrophilic reagents. The desired products were obtained in good to excellent yields and ees with **L10** as ligand.

**5. Mechanistic Investigation**

**5.1 Competitive Experiment**

Follow the general procedure for the electrochemical asymmetric arylation of enones, the **2a** (0.2 mmol, 1 equiv.) and **2f** (0.2 mmol, 1 equiv.) were used as electrophilic reagents. The corresponding products **3f** and **3j** were obtained in 19%, 71% yield and 91%, 96% ee with **L10** as ligand, respectively. This result shown that electron deficient aryl bromide is more reactive than electron rich aryl bromide in this protocol.

**5.2 Water additive Experiments**

**Table S4. Water additive experiments**

| **Entry*^a^*** | **Solvent** | **Additive** | **Yield%*^b^*** | **ee%*^c^*** |
| --- | --- | --- | --- | --- |
| 1 | DMF & DMSO | -- | 97 | 96 |
| 2 | anhydrous DMF & DMSO | -- | 20 | 92 |
| 3 | anhydrous DMF & DMSO | H_2_O (1.5 eq.) | 62 | 94 |
| 4 | anhydrous DMF & DMSO | H_2_O (3.0 eq.) | 85 | 95 |
| [a] Reaction conditions (unless otherwise specified): **1b** (0.2 mmol), **2f** (0.3 mmol, 1.5 equiv), NiBr_2_DME (10 mmol%), **L10** (12 mmol%), SS(+)/SS(-) electrodes, DMF/DMSO (1 mL / 1 mL). [b] Isolated yield. [c] Enantioselectivities were determined by chiral HPLC analysis. | | | | |

Analytical DMF and DMSO were used as solvents in entry 1. Anhydrous DMF and DMSO were used as solvents in entries 2-4.

**5.3 Deuterium Experiment**

Follow the general procedure for the electrochemical asymmetric arylation of enones, the DMSO-*d*_6_ (1 mL) were used as solvent instead of DMSO (1 mL). The product 3j has been confirmed by ^1^H-NMR without deuterium (Figure S2).


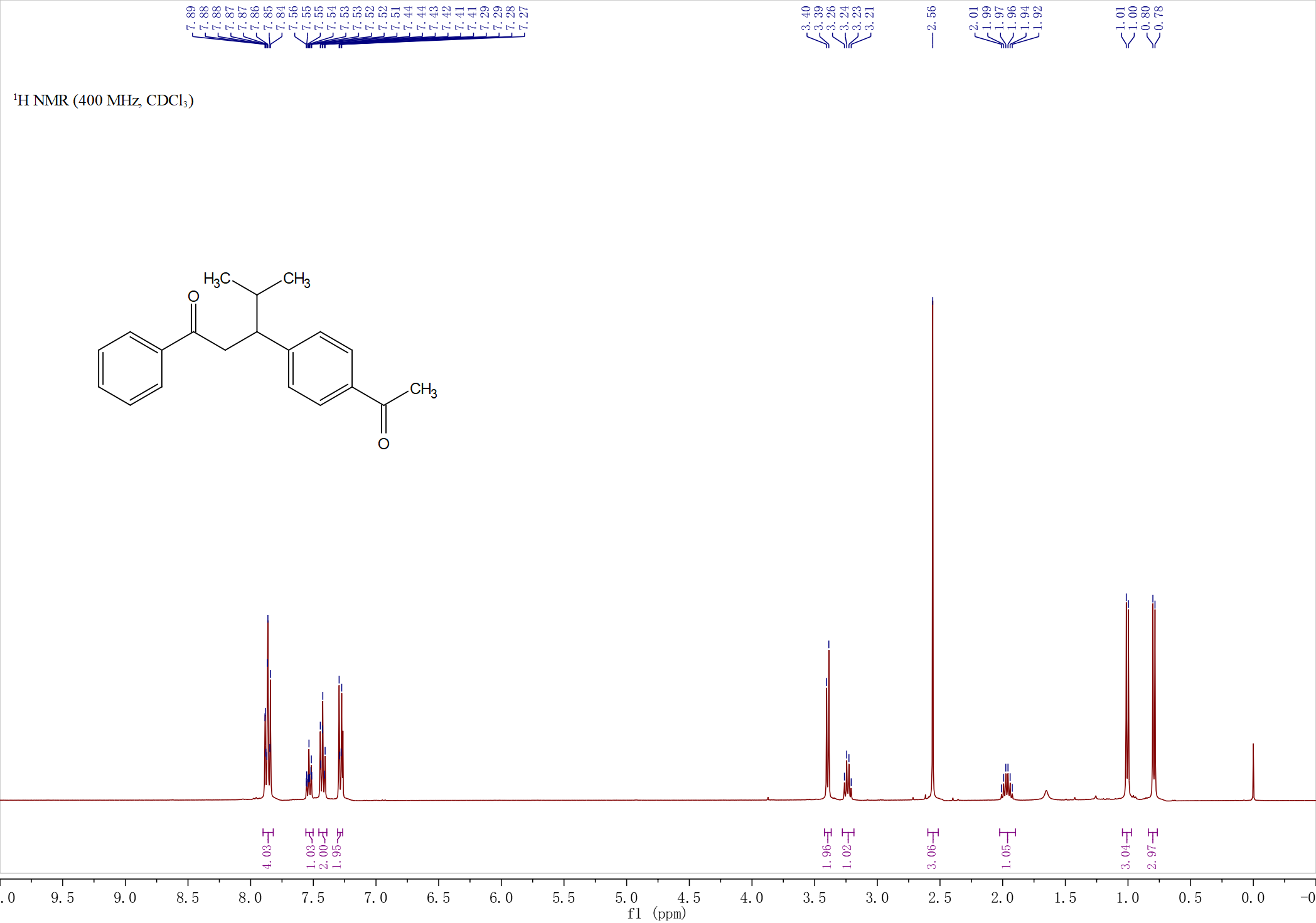


**Figure S2**. ^1^H-NMR of **3j**

Follow the general procedure for the electrochemical asymmetric arylation of enones, the D_2_O (2 equiv.) was used as additive. The product has been confirmed by ^1^H-NMR with deuterium at both α positions in nearly 1:1 ratio (Figure S3).


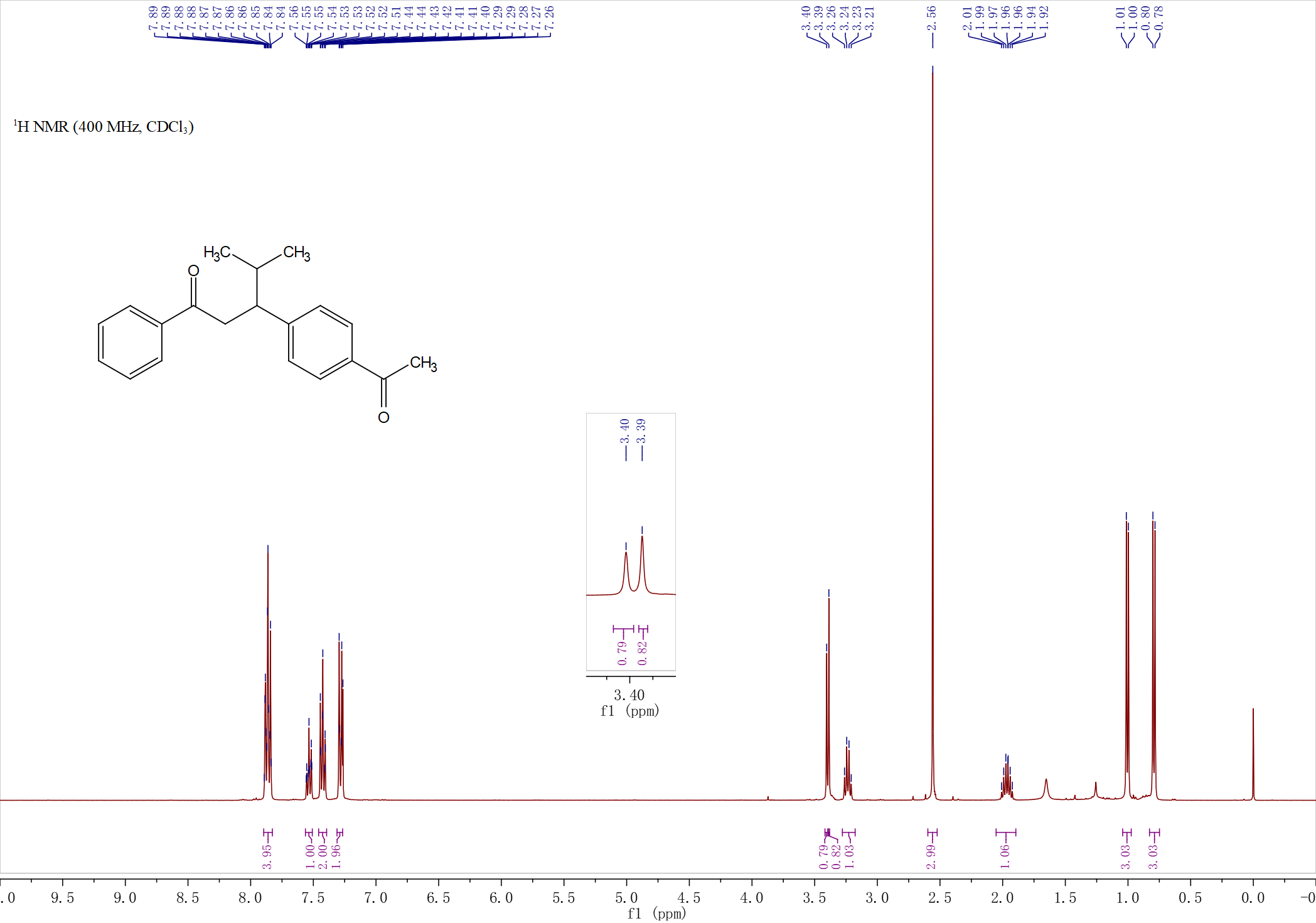


**Figure S3**. ^1^H-NMR of **3j-d**

**5.4 Additive Experiments**

1. Follow the general procedure for the electrochemical asymmetric arylation of enones, the butylated hydroxytoluene (BHT, 2 equiv.) was used as additive. The desired product was formed in 86% yield and 95% ee.
2. Follow the general procedure for the electrochemical asymmetric arylation of enones, the bis(pinacolato)diboron (B_2_Pin_2_, 2 equiv.) was used as additive. The desired product was formed in 85% yield and 95% ee.
3. Follow the general procedure for the electrochemical asymmetric arylation of enones, the chlorotrimethylsilane (TMSCl, 2 equiv.) was used as additive. The desired product was formed in 51% yield and 95% ee.

**5.5 Stoichiometric Reactions**

**Synthesis of Ni-complex 6**

The Ni-complex was prepared according to the reported procedure (*Angew. Chem. Int. Ed.* **2020**, *59*, 4370–4374).^2^ In a nitrogen filled glove box, a 50 mL round bottom flask containing a stirring bar was charged with Ni(COD)_2_ (276 mg, 1.0 mmol, 1.0 equiv), 2,2'-bipyridine (156 mg, 1.0 mmol, 1.0 equiv) and dry THF (10 mL) giving a dark purple mixture which was stirred overnight at 25 ºC. 1-bromo-4-(trifluoromethyl)benzene (1.4 mL, 10 mmol, 10.0 equiv) was added and stirred for additional 1 h. Dry pentane (30 mL) was added to the orange colored mixture and filtered. The resulting precipitate was washed with pentane (3 x 10 mL) and dried under vacumm to afford Ni-complex **6** (330 mg, 75% yield) as an orange solid. *The product was used immediately without further purification.*

**Stoichiometric experiments**

**Without current**: In a nitrogen filled glove box, a 10 mL round bottom flask containing a stirring bar was charged with Ni-complex **6** (87 mg, 0.2 mmol, 1.0 equiv), **1b** (70 mg, 0.4 mmol, 2.0 equiv), DMF (1 mL) and DMSO (1 mL). Then the reaction mixture was sealed, taken out from glove box and stirred at 23 ºC for 8 hours. TLC shows trace desired product rac-**3k** was formed.

**With current**: In a nitrogen filled glove box, a 10 mL Schlenk tube containing a stirring bar was charged with Ni-complex **6** (87 mg, 0.2 mmol, 1.0 equiv), **1b** (70 mg, 0.4 mmol, 2.0 equiv), DMF (1 mL) and DMSO (1 mL). The tube was sealed with rubber septum which equipped with stainless steel electrodes (1.5 cm x 1 cm, about 1 cm immersion depth in solution, *S* = 1 cm^2^) as anode and cathode. Then the reaction mixture was taken out from glove box and electrolyzed under a constant current of 10 mA for 2 hours. After the reaction, the electrodes were taken out and rinsed with EtOAc. Aqueous sat. EDTA was then added; the resulting mixture was extracted with EtOAc. The combined organic layer was dried over anhydrous Na_2_SO_4_ and concentrated in vacuo. The crude material was purified by column chromatography to furnish the desired product rac-**3k** as white solid: 51 mg, 80% yield.

**5.6 On/off experiment**

**Figure S4.** On/off experiment.

**5.7 Time course experiment of electrochemical reduction Vs. Mn reductant**

**Figure S5.** Time course experiment of electrochemical reduction Vs. Mn reductant

**5.8 Weight variation of electrodes**

**The amount of iron, chromium and nickel released during the electrolysis was obtained by weighing the stainless-steel rod anode before and after the electrolysis.**

0.2 mmol scale: After the reaction, the electrodes were taken out and rinsed with EtOAc, submersed in 1 N HCl and ultrasound for 30 S, wash with water and dry. Weighing the weight variation of electrodes.

| Entry | 1 | 2 | 3 | 4 | 5 |
| --- | --- | --- | --- | --- | --- |
| Weight loss of SS anode | 20.0 mg | 20.1 mg | 20.1 mg | 20.0 mg | 19.9 mg |
| Weight variation of SS cathode | 0 mg | 0 mg | 0 mg | 0 mg | 0 mg |

Fe = 55.85, Cr = 52, Ni = 58.69

SS = (55.85*72%+52*18%+58.69*10%) = 55.442

n *_SS_* = 20/55.442 = 0.36 mmol

n *_Fe_* = 0.36*72% = 0.26 mmol

n *_Cr_* = 0.36*18% = 0.065 mmol

n *_Ni_* = 0.36*10% = 0.036 mmol

**In all cases, 20 mg of the sacrificial anode were consumed. The weighting proportions of Fe/Cr/Ni were 72/18/10, corresponding to 0.26 mmol of iron (1.3 eq.), 0.065 mmol of chromium (32.4%) and 0.036 mmol of nickel (18%).**

**5.9 Cyclic Voltammetry Studies**

All the voltammetric experiments were recorded with a CHI600E potentiostat at room temperature in DMSO. *^n^*Bu_4_NPF_6_(0.1 M) was used as the supporting electrolyte, a glass carbon electrode (diameter, 3 mm) and a platinum wire were used as working and counter electrodes, respectively. The working electrode potentials were measured versus Ag/AgNO_3_ reference electrode (internal solution, 0.1 M AgNO_3_ in DMSO). The scan rate was 100 mV s^-1^.


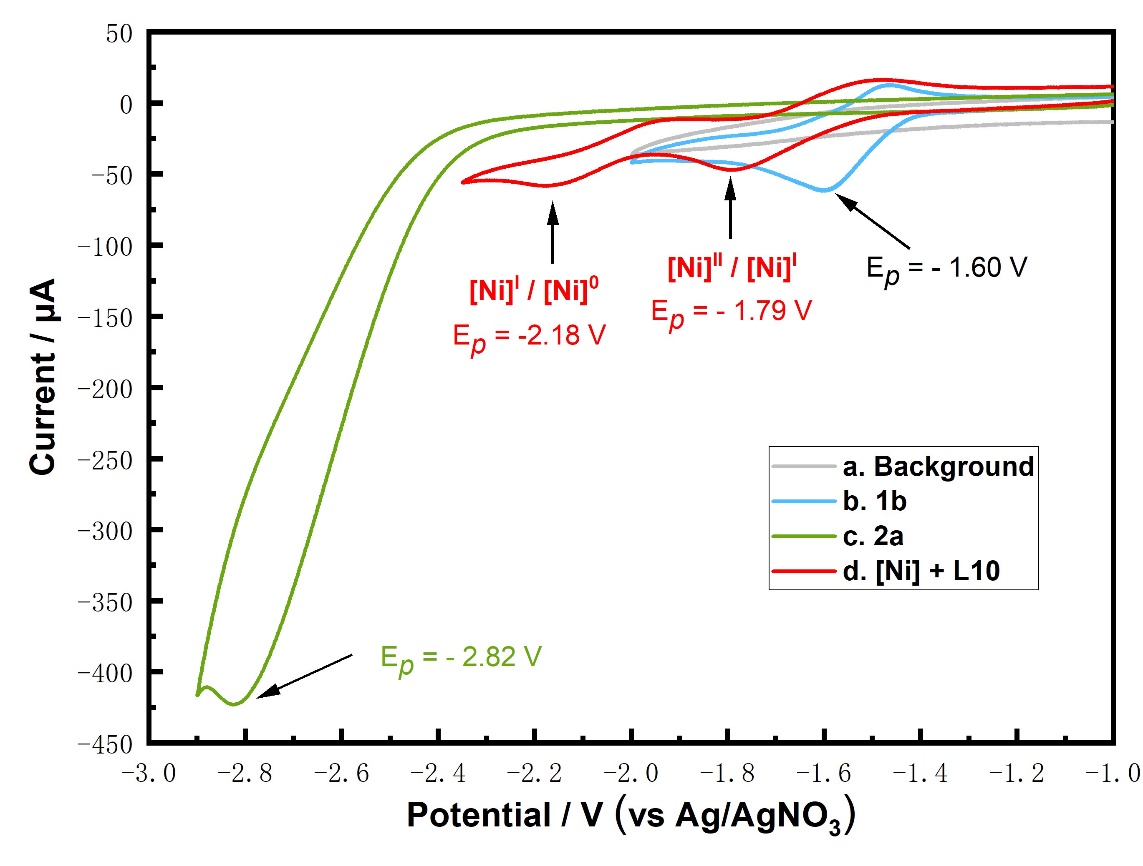


**Figure S6**. Cyclic voltammograms. a. background (0.1 M *^n^*Bu_4_NPF_6_ in DMSO) (black line); b. 10 mM **1b** (blue line); c. 10 mM **2a** (green line); d. 5 mM NiBr_2_•DME+ 5 mM **L10** (red line).


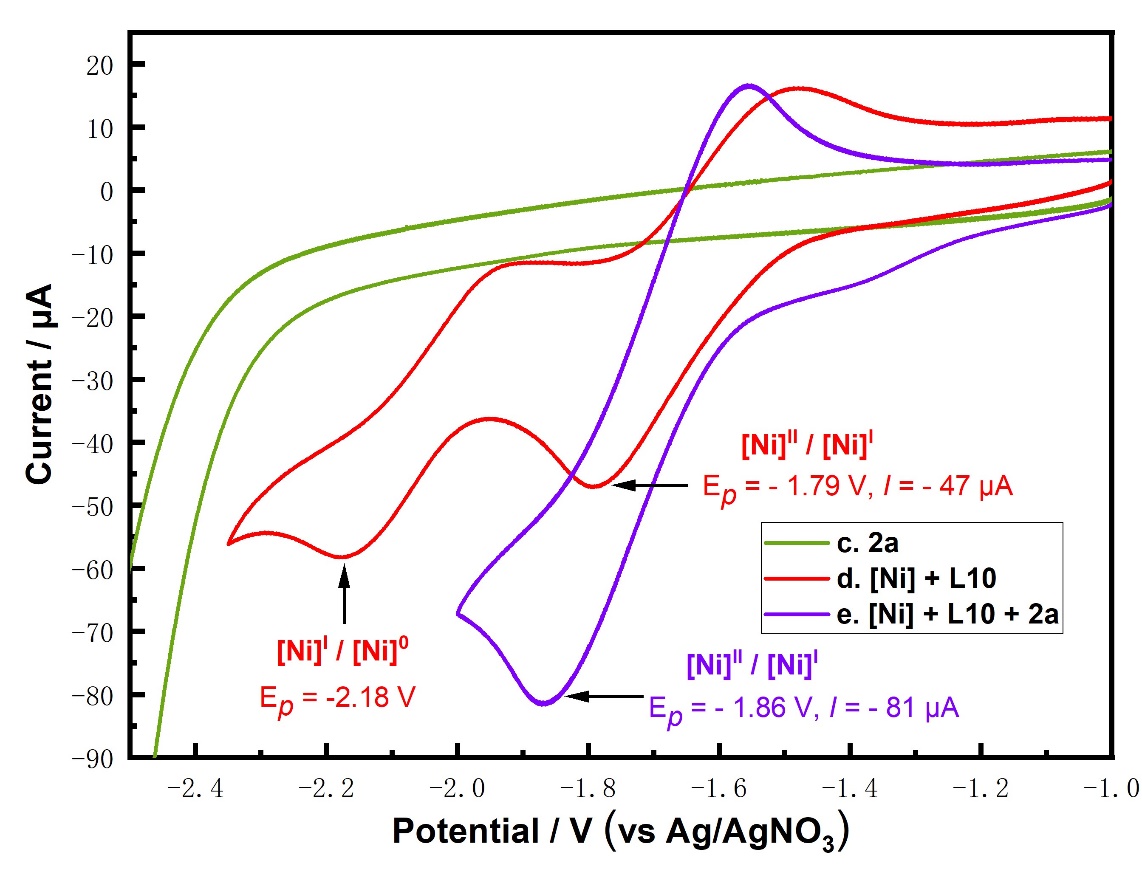


**Figure S7**. Cyclic voltammograms. c. 10 mM **2a** (green line); d. 5 mM NiBr_2_•DME+ 5 mM **L10** (red line); e. 5 mM NiBr_2_•DME+ 5 mM **L10 +** 10 mM **2a** (purple line).

**5.10** **Comparison of electrochemical and nonelectrochemical conditions**

The comparison of electrochemical and nonelectrochemical conditions using 4-bromoanisole as the substrate were conducted. And the results indicate the electro-protocol can promote this process.

**6. Failed Examples**

**Figure S8**. Failed examples including hindered aryl bromides, cyclic enones, unsatisfactory results, failed aryl bromides and failed enones.

**7. Characterization Data for the Products**

 **(*R*)-(1,3-diphenyl-3-(*p*-tolyl)propan-1-one (3a)**:

The product was isolated by flash chromatography (PE/EA = 30/1) as white solid. Ligand = **L1**, 55.5 mg, 93% yield, 92% ee. [α]^25^ D = -5.4° (c = 1.61, CHCl_3_).

**HPLC**: Daicel Chiralcel AD-H, *n*-hexane/isopropanol 95/5, flow rate = 1.0 mL/min, l = 254 nm, *t*_R_ = 9.8 min (major), 11.7 min (minor).

**^1^H NMR** (400 MHz, CDCl_3_) δ 8.00 – 7.94 (m, 2H), 7.61 – 7.55 (m, 1H), 7.47 (dd, *J* = 8.3, 7.0 Hz, 2H), 7.29 (d, *J* = 4.3 Hz, 3H), 7.22 – 7.17 (m, 3H), 7.11 (d, *J* = 8.0 Hz, 2H), 4.82 (t, *J* = 7.3 Hz, 1H), 3.75 (d, *J* = 7.4 Hz, 2H), 2.32 (s, 3H).

**^13^C NMR** (101 MHz, CDCl_3_) δ 198.1, 144.4, 141.2, 137.1, 135.9, 133.1, 129.3, 128.6, 128.6, 128.1, 127.8, 127.7, 126.3, 45.6, 44.8, 21.0.

**LC-MS** (ESI) *m/z* Calcd for C_22_H_21_O [M+H^+^]: 301.2; found: 301.1.

Spectroscopic data are in accordance with that reported in the literature.^3^

**(*R*)-3-(4-methoxyphenyl)-1,3-diphenylpropan-1-one (3b)**:

The product was isolated by flash chromatography (PE/EA = 20/1) as white solid. Ligand = **L1**, 53 mg, 84% yield, 93% ee. [α]^25^ D = -2.2° (c = 2.3, CHCl_3_).

**HPLC**: Daicel Chiralcel AD-H, *n*-hexane/isopropanol 95/5, flow rate = 1.0 mL/min, l = 254 nm, *t*_R_ = 19.7 min (major), 21.6 min (minor).

**^1^H NMR** (400 MHz, CDCl_3_) δ 7.99 – 7.93 (m, 2H), 7.61 – 7.54 (m, 1H), 7.47 (dd, *J* = 8.4, 7.0 Hz, 2H), 7.29 (d, *J* = 5.6 Hz, 4H), 7.21 (dq, *J* = 9.1, 3.5 Hz, 3H), 6.87 – 6.81 (m, 2H), 4.81 (t, *J* = 7.3 Hz, 1H), 3.78 (s, 3H), 3.74 (d, *J* = 7.4 Hz, 2H).

**^13^C NMR** (101 MHz, CDCl_3_) δ 198.2, 158.0, 144.5, 137.1, 136.3, 133.1, 128.8, 128.6, 128.6, 128.1, 127.8, 126.3, 113.9, 55.2, 45.2, 44.9.

**LC-MS** (ESI) *m/z* Calcd for C_22_H_21_O_2_ [M+H^+^]: 317.2; found: 317.2.

Spectroscopic data are in accordance with that reported in the literature.^3^

**Methyl (*R*)-4-(3-oxo-1,3-diphenylpropyl)benzoate (3c):**

The product was isolated by flash chromatography (PE/EA = 10/1) as white solid. Ligand = **L1**, 50 mg, 73% yield, 91% ee.

**HPLC**: Daicel Chiralcel IG, *n*-hexane/isopropanol 70/30, flow rate = 0.8 mL/min, l = 254 nm, *t*_R_ = 20.9 min (major), 24.4 min (minor).

**^1^H NMR** (400 MHz, CDCl_3_) δ 7.99 – 7.94 (m, 4H), 7.61 – 7.56 (m, 1H), 7.47 (dd, *J* = 8.4, 7.0 Hz, 2H), 7.39 – 7.35 (m, 2H), 7.34 – 7.25 (m, 4H), 7.25 – 7.19 (m, 1H), 4.91 (t, *J* = 7.3 Hz, 1H), 3.90 (s, 3H), 3.79 (dd, *J* = 7.3, 4.2 Hz, 2H).

**^13^C NMR** (101 MHz, CDCl_3_) δ 197.6, 167.0, 149.4, 143.4, 136.8, 133.3, 130.0, 128.8, 128.7, 128.3, 128.1, 127.9, 127.8, 126.7, 52.1, 45.9, 44.4.

**LC-MS** (ESI) *m/z* Calcd for C_23_H_21_O_3_ [M+H^+^]: 345.2; found: 345.1.

Spectroscopic data are in accordance with that reported in the literature.^4^

**(*R*)-3-(4-acetylphenyl)-1,3-diphenylpropan-1-one (3d):**

The product was isolated by flash chromatography (PE/EA = 10/1) as white solid. Ligand = **L1**, 60.5 mg, 92% yield, 87% ee.

**HPLC**: Daicel Chiralcel AD-H, *n*-hexane/isopropanol 80/20, flow rate = 1.0 mL/min, l = 254 nm, *t*_R_ = 16.2 min (major), 17.2 min (minor).

**^1^H NMR** (400 MHz, CDCl_3_) δ 7.98 – 7.91 (m, 2H), 7.87 (d, *J* = 8.4 Hz, 2H), 7.57 (t, *J* = 7.4 Hz, 1H), 7.45 (t, *J* = 7.7 Hz, 2H), 7.40 – 7.35 (m, 2H), 7.32 – 7.23 (m, 4H), 7.23 – 7.17 (m, 1H), 4.89 (t, *J* = 7.3 Hz, 1H), 3.86 – 3.68 (m, 2H), 2.55 (s, 3H).

**^13^C NMR** (101 MHz, CDCl_3_) δ 197.77, 197.55, 149.65, 143.31, 136.78, 135.39, 133.36, 128.80, 128.78, 128.72, 128.10, 128.07, 127.81, 126.77, 45.86, 44.31, 26.64.

**LC-MS** (ESI) *m/z* Calcd for C_23_H_21_O_2_ [M+H^+^]: 329.2; found: 329.2.

Spectroscopic data are in accordance with that reported in the literature.^5^

**(*R*)-3-(naphthalen-2-yl)-1,3-diphenylpropan-1-one (3e):**

The product was isolated by flash chromatography (PE/EA = 30/1) as white solid. Ligand = **L1**, 64 mg, 95% yield, 90% ee.

**HPLC**: Daicel Chiralcel AD-H, *n*-hexane/isopropanol 90/10, flow rate = 1.0 mL/min, l = 254 nm, *t*_R_ = 10.4 min (major), 11.3 min (minor).

**^1^H NMR** (400 MHz, CDCl_3_) δ 7.99 – 7.92 (m, 2H), 7.79 – 7.69 (m, 4H), 7.58 – 7.51 (m, 1H), 7.48 – 7.36 (m, 5H), 7.34 – 7.29 (m, 2H), 7.28 – 7.24 (m, 2H), 7.21 – 7.14 (m, 1H), 5.00 (t, *J* = 7.3 Hz, 1H), 3.92 – 3.77 (m, 2H).

**^13^C NMR** (101 MHz, CDCl_3_) δ 198.0, 144.0, 141.6, 137.0, 133.5, 133.2, 132.2, 128.7, 128.6, 128.3, 128.1, 128.0, 127.8, 127.6, 126.8, 126.5, 126.1, 125.8, 125.6, 46.0, 44.6.

**LC-MS** (ESI) *m/z* Calcd for C_25_H_21_O [M+H^+^]: 337.2; found: 337.2.

Spectroscopic data are in accordance with that reported in the literature.^4^

**(*R*)-4-methyl-1-phenyl-3-(*p*-tolyl)pentan-1-one (3f):**

The product was isolated by flash chromatography (PE/EA = 30/1) as colorless oil. Ligand = **L10**, 48 mg, 90% yield. 91% ee.

**HPLC**: Daicel Chiralcel AD-H, *n*-hexane/isopropanol 95/5, flow rate = 1.0 mL/min, l = 254 nm, *t*_R_ = 5.6 min (major), 7.6 min (minor).

**^1^H NMR** (400 MHz, CDCl_3_) δ 7.90 – 7.83 (m, 2H), 7.54 – 7.49 (m, 1H), 7.45 – 7.38 (m, 2H), 7.05 (s, 4H), 3.33 (dd, *J* = 7.0, 2.4 Hz, 2H), 3.13 (td, *J* = 7.4, 6.4 Hz, 1H), 2.28 (s, 3H), 1.91 (dq, *J* = 13.6, 6.8 Hz, 1H), 0.97 (d, *J* = 6.7 Hz, 3H), 0.79 (d, *J* = 6.7 Hz, 3H).

**^13^C NMR** (101 MHz, CDCl_3_) δ 199.6, 140.5, 137.4, 135.5, 132.8, 128.8, 128.5, 128.2, 128.1, 47.4, 42.6, 33.3, 21.0, 21.0, 20.3.

**LC-MS** (ESI) *m/z* Calcd for C_19_H_23_O [M+H^+^]: 267.2; found: 267.2.

Spectroscopic data are in accordance with that reported in the literature.^6^

**(*R*)-4-methyl-3-(4-(methylthio)phenyl)-1-phenylpentan -1-one (3g):**

The product was isolated by flash chromatography (PE/EA = 30/1) as white solid. Ligand = **L10**, 51 mg, 86% yield, 95% ee.

**HPLC**: Daicel Chiralcel AD-H, *n*-hexane/isopropanol 95/5, flow rate = 1.0 mL/min, l = 254 nm, *t*_R_ = 8.6 min (major), 12.2 min (minor).

**^1^H NMR** (400 MHz, CDCl_3_) δ 7.90 – 7.82 (m, 2H), 7.56 – 7.49 (m, 1H), 7.42 (dd, *J* = 8.2, 7.0 Hz, 2H), 7.18 – 7.07 (m, 4H), 3.38 – 3.30 (m, 2H), 3.12 (q, *J* = 7.2 Hz, 1H), 2.44 (s, 3H), 1.91 (dq, *J* = 13.6, 6.8 Hz, 1H), 0.97 (d, *J* = 6.7 Hz, 3H), 0.79 (d, *J* = 6.8 Hz, 3H).

**^13^C NMR** (101 MHz, CDCl_3_) δ 199.4, 140.7, 137.3, 135.6, 132.9, 128.9, 128.5, 128.0, 126.6, 47.3, 42.4, 33.2, 20.9, 20.3, 16.1.

**LC-MS** (ESI) *m/z* Calcd for C_19_H_23_OS [M+H^+^]: 299.1; found: 299.1.

Spectroscopic data are in accordance with that reported in the literature.^6^

**(*R*)-3-([1,1'-biphenyl]-4-yl)-4-methyl-1-phenylpentan-1-one (3h):**

The product was isolated by flash chromatography (PE/EA = 20/1) as white solid. Ligand = **L10**, 56.5 mg, 86% yield, 92% ee.

**HPLC**: Daicel Chiralcel AD-H, *n*-hexane/isopropanol 95/5, flow rate = 1.0 mL/min, l = 254 nm, *t*_R_ = 8.6 min (major), 12.5 min (minor).

**^1^H NMR** (400 MHz, CDCl_3_) δ 7.93 – 7.86 (m, 2H), 7.58 – 7.50 (m, 3H), 7.50 – 7.45 (m, 2H), 7.41 (td, *J* = 7.9, 6.6 Hz, 4H), 7.33 – 7.28 (m, 1H), 7.27 – 7.22 (m, 2H), 3.39 (d, *J* = 7.1 Hz, 2H), 3.22 (q, *J* = 7.1 Hz, 1H), 1.97 (h, *J* = 6.8 Hz, 1H), 1.01 (d, *J* = 6.7 Hz, 3H), 0.84 (d, *J* = 6.7 Hz, 3H).

**^13^C NMR** (101 MHz, CDCl_3_) δ 199.5, 142.8, 141.0, 138.9, 137.4, 132.9, 128.8, 128.7, 128.5, 128.1, 127.0, 127.0, 126.8, 47.4, 42.5, 33.3, 21.0, 20.4.

**LC-MS** (ESI) *m/z* Calcd for C_24_H_25_O [M+H^+^]: 329.2; found: 329.2.

Spectroscopic data are in accordance with that reported in the literature.^6^

**Methyl (*R*)-4-(4-methyl-1-oxo-1-phenylpentan-3-yl) benzoate (3i):**

The product was isolated by flash chromatography (PE/EA=10/1) as colorless oil. Ligand = **L10**, 60 mg, 97% yield, 96% ee.

**HPLC**: Daicel Chiralcel AD-H, *n*-hexane/isopropanol 85/15, flow rate = 1.0 mL/min, l = 254 nm, *t*_R_ = 9.2 min (major), 11.8 min (minor).

**^1^H NMR** (400 MHz, CDCl_3_) δ 7.95 – 7.90 (m, 2H), 7.89 – 7.84 (m, 2H), 7.56 – 7.50 (m, 1H), 7.47 – 7.39 (m, 2H), 7.26 (d, *J* = 8.4 Hz, 2H), 3.88 (s, 3H), 3.38 (d, *J* = 7.0 Hz, 2H), 3.23 (q, *J* = 7.2 Hz, 1H), 2.02 – 1.89 (m, 1H), 1.00 (d, *J* = 6.7 Hz, 3H), 0.78 (d, *J* = 6.7 Hz, 3H).

**^13^C NMR** (101 MHz, CDCl_3_) δ 199.0, 167.1, 149.3, 137.2, 133.0, 129.5, 128.6, 128.4, 128.1, 128.0, 52.0, 47.8, 42.2, 33.2, 20.9, 20.4.

**LC-MS** (ESI) *m/z* Calcd for C_20_H_23_O_3_ [M+H^+^]: 311.2; found: 311.2.

**(*R*)-3-(4-acetylphenyl)-4-methyl-1-phenylpentan-1-one(3j):**

The product was isolated by flash chromatography (PE/EA = 10/1) as white solid. When 4-Ac-Ph-Br was used as electrophile, Ligand = **L10**, the reaction mixture affords the product **3j** 57 mg, 97% yield, 96% ee. When 4-Ac-Ph-OTf was used as electrophile, Ligand = **L10**, the reaction mixture affords the product **3j** 49 mg, 83% yield, 95% ee.

**HPLC**: Daicel Chiralcel AD-H, *n*-hexane/isopropanol 90/10, flow rate = 1.0 mL/min, l = 254 nm, *t*_R_ = 15.5 min (major), 20.5 min (minor).

**^1^H NMR** (400 MHz, CDCl_3_) δ 7.90 – 7.82 (m, 4H), 7.56 – 7.50 (m, 1H), 7.42 (dd, *J* = 8.4, 7.0 Hz, 2H), 7.28 (d, *J* = 8.3 Hz, 2H), 3.39 (d, *J* = 7.0 Hz, 2H), 3.24 (q, *J* = 7.2 Hz, 1H), 2.55 (s, 3H), 1.97 (dq, *J* = 13.7, 6.8 Hz, 1H), 1.00 (d, *J* = 6.7 Hz, 3H), 0.79 (d, *J* = 6.7 Hz, 3H).

**^13^C NMR** (101 MHz, CDCl_3_) δ 198.9, 197.9, 149.7, 137.1, 135.3, 133.1, 128.6, 128.6, 128.3, 128.0, 47.8, 42.1, 33.2, 26.6, 20.9, 20.5.

**LC-MS** (ESI) *m/z* Calcd for C_20_H_23_O_2_ [M+H^+^]: 295.2; found: 295.2.

Spectroscopic data are in accordance with that reported in the literature.^6^

**(*R*)-4-methyl-1-phenyl-3-(4-(trifluoromethyl)phenyl) pentan-1-one (3k):**

The product was isolated by flash chromatography (PE/EA = 30/1) as white solid. Ligand = **L10**, 60 mg, 94% yield. 96% ee.

**HPLC**: Daicel Chiralcel AD-H, *n*-hexane/isopropanol 95/5, flow rate = 1.0 mL/min, l = 254 nm, *t*_R_ = 5.1 min (major), 6.6 min (minor).

**^1^H NMR** (400 MHz, CDCl_3_) δ 7.90 – 7.84 (m, 2H), 7.43 (dd, *J* = 8.3, 7.0 Hz, 2H), 7.30 (d, *J* = 8.0 Hz, 2H), 3.48 – 3.31 (m, 2H), 3.24 (q, *J* = 7.2 Hz, 1H), 1.96 (dq, *J* = 13.7, 6.8 Hz, 1H), 1.00 (d, *J* = 6.7 Hz, 3H), 0.79 (d, *J* = 6.7 Hz, 3H).

**^13^C NMR** (101 MHz, CDCl_3_) δ 198.8, 147.9, 137.1, 133.1, 132.9 (d, *J* *_C-F_* = 9.6 Hz), 128.6, 128.6, 128.3, 128.0, 125.1 (dd, *J* *_C-F_* = 3.8 Hz), 124.3 (dd, *J* *_C-F_* = 270.2 Hz), 47.6, 42.1, 33.2, 27.9, 25.8, 22.5, 20.8, 20.4.

**LC-MS** (ESI) *m/z* Calcd for C_19_H_20_F_3_O [M+H^+^]: 321.1; found: 321.1.

Spectroscopic data are in accordance with that reported in the literature.^6^

**(*R*)-4-(4-methyl-1-oxo-1-phenylpentan-3-yl)benzonitrile (3l):**

The product was isolated by flash chromatography (PE/EA = 10/1) as white solid. Ligand = **L10**, 50 mg, 90% yield. 92% ee.

**HPLC**: Daicel Chiralcel AD-H, *n*-hexane/isopropanol 85/15, flow rate = 1.0 mL/min, l = 254 nm, *t*_R_ = 9.4 min (major), 12.7 min (minor).

**^1^H NMR** (400 MHz, CDCl_3_) δ 7.90 – 7.83 (m, 2H), 7.58 – 7.51 (m, 3H), 7.43 (dd, *J* = 8.4, 7.0 Hz, 2H), 7.33 – 7.28 (m, 2H), 3.39 (dd, *J* = 7.0, 2.9 Hz, 2H), 3.22 (td, *J* = 7.8, 6.0 Hz, 1H), 2.01 – 1.89 (m, 1H), 1.00 (d, *J* = 6.7 Hz, 3H), 0.78 (d, *J* = 6.8 Hz, 3H).

**^13^C NMR** (101 MHz, CDCl_3_) δ 198.5, 149.6, 136.9, 133.2, 132.0, 129.2, 128.7, 127.9, 119.1, 110.0, 47.9, 41.9, 33.1, 20.8, 20.5.

**LC-MS** (ESI) *m/z* Calcd for C_19_H_20_NO [M+H^+^]: 278.1; found: 278.1.

Spectroscopic data are in accordance with that reported in the literature.^6^

**(*R*)-4-(4-methyl-1-oxo-1-phenylpentan-3-yl)benzene sulfonamide (3m):**

The product was isolated by flash chromatography (PE/EA = 2/1) as white solid. Ligand = **L10**, 56 mg, 85% yield. 95% ee.

**HPLC**: Daicel Chiralcel AD-H, *n*-hexane/isopropanol 70/30, flow rate = 0.7 mL/min, l = 254 nm, *t*_R_ = 13.5 min (major), 14.7 min (minor).

**^1^H NMR** (400 MHz, CDCl_3_) δ 7.91 – 7.84 (m, 2H), 7.84 – 7.76 (m, 2H), 7.59 – 7.50 (m, 1H), 7.43 (dd, *J* = 8.5, 7.1 Hz, 2H), 7.37 – 7.30 (m, 2H), 4.96 (d, *J* = 3.4 Hz, 2H), 3.43 – 3.37 (m, 2H), 3.24 (q, *J* = 7.2 Hz, 1H), 1.96 (dq, *J* = 13.7, 6.8 Hz, 1H), 1.00 (d, *J* = 6.7 Hz, 3H), 0.79 (d, *J* = 6.7 Hz, 3H).

**^13^C NMR** (101 MHz, CDCl_3_) δ 198.8, 149.5, 139.6, 136.9, 133.2, 129.1, 128.7, 128.0, 126.3, 47.6, 42.0, 33.2, 20.8, 20.5.

**LC-MS** (ESI) *m/z* Calcd for C_18_H_22_NO_3_S [M+H^+^]: 332.1; found: 332.1.

**(*R*)-*N*-(4-(4-methyl-1-oxo-1-phenylpentan-3-yl)phenyl) acetamide (3n):**

The product was isolated by flash chromatography (PE/EA = 2/1) as white solid. When 4-AcNH-Ph-I was used as electrophile, Ligand = **L10**, the reaction mixture affords the product **3n** 45 mg, 73% yield, 96% ee.

**HPLC**: Daicel Chiralcel AD-H, *n*-hexane/isopropanol 80/20, flow rate = 1.0 mL/min, l = 254 nm, *t*_R_ = 8.4 min (major), 12.2 min (minor).

**^1^H NMR** (400 MHz, CDCl_3_) δ 7.89 – 7.84 (m, 2H), 7.55 – 7.49 (m, 1H), 7.42 (dd, *J* = 8.2, 7.0 Hz, 2H), 7.38 – 7.34 (m, 2H), 7.11 (d, *J* = 8.5 Hz, 2H), 3.33 (dd, *J* = 7.0, 2.0 Hz, 2H), 3.12 (q, *J* = 7.2 Hz, 1H), 2.13 (s, 3H), 1.91 (h, *J* = 6.8 Hz, 1H), 0.97 (d, *J* = 6.6 Hz, 3H), 0.78 (d, *J* = 6.7 Hz, 3H).

**^13^C NMR** (101 MHz, CDCl_3_) δ 199.6, 168.2, 139.6, 137.3, 135.9, 132.9, 128.8, 128.5, 128.0, 119.6, 47.4, 42.5, 33.3, 24.6, 20.9, 20.3.

**LC-MS** (ESI) *m/z* Calcd for C_20_H_24_NO_2_ [M+H^+^]: 310.2; found: 310.2.

**(*R*)-3-(3-acetylphenyl)-4-methyl-1-phenylpentan-1-one (3o):**

The product was isolated by flash chromatography (PE/EA = 10/1) as colorless oil. Ligand = **L10**, 56 mg, 95% yield, 94% ee.

**HPLC**: Daicel Chiralcel AD-H, *n*-hexane/isopropanol 80/20, flow rate = 1.0 mL/min, l = 254 nm, *t*_R_ = 6.3 min (major), 8.3 min (minor).

**^1^H NMR** (400 MHz, CDCl_3_) δ 7.90 – 7.85 (m, 2H), 7.78 (d, *J* = 1.8 Hz, 1H), 7.74 (dt, *J* = 7.6, 1.5 Hz, 1H), 7.56 – 7.49 (m, 1H), 7.45 – 7.39 (m, 3H), 7.35 (t, *J* = 7.6 Hz, 1H), 3.40 (dd, *J* = 6.9, 1.2 Hz, 2H), 3.24 (q, *J* = 7.2 Hz, 1H), 2.57 (s, 3H), 2.04 – 1.90 (m, 1H), 1.00 (d, *J* = 6.7 Hz, 3H), 0.79 (d, *J* = 6.7 Hz, 3H).

**^13^C NMR** (101 MHz, CDCl_3_) δ 199.1, 198.4, 144.4, 137.2, 137.0, 133.6, 133.0, 128.6, 128.3, 128.0, 127.7, 126.5, 47.7, 42.3, 33.2, 26.7, 20.8, 20.5.

**LC-MS** (ESI) *m/z* Calcd for C_20_H_23_O_2_ [M+H^+^]: 295.2; found: 295.2.

**(*R*)-5-(4-methyl-1-oxo-1-phenylpentan-3-yl)isobenzo-furan-1(3*H*)-one (3p):**

The product was isolated by flash chromatography (PE/EA = 6/1) as white solid. Ligand = **L10**, 48 mg, 78% yield. 88% ee.

**HPLC**: Daicel Chiralcel AD-H, *n*-hexane/isopropanol 80/20, flow rate = 1.0 mL/min, l = 254 nm, *t*_R_ = 13.8 min (major), 18.1 min (minor).

**^1^H NMR** (400 MHz, CDCl_3_) δ 7.87 (dd, *J* = 8.2, 1.3 Hz, 2H), 7.80 (d, *J* = 7.9 Hz, 1H), 7.58 – 7.51 (m, 1H), 7.43 (t, *J* = 7.6 Hz, 2H), 7.37 (d, *J* = 8.0 Hz, 1H), 7.32 (s, 1H), 5.26 (s, 2H), 3.43 (d, *J* = 6.9 Hz, 2H), 3.29 (q, *J* = 7.2 Hz, 1H), 1.99 (dq, *J* = 13.7, 6.8 Hz, 1H), 1.03 (d, *J* = 6.7 Hz, 3H), 0.80 (d, *J* = 6.7 Hz, 3H).

**^13^C NMR** (101 MHz, CDCl_3_) δ 198.6, 171.1, 151.4, 146.8, 136.9, 133.2, 129.1, 128.7, 127.9, 125.5, 123.9, 122.1, 69.6, 48.2, 42.2, 33.3, 20.8, 20.7.

**LC-MS** (ESI) *m/z* Calcd for C_20_H_21_O_3_ [M+H^+^]: 309.1; found: 309.1.

**(*R*)-3-(9*H*-fluoren-2-yl)-4-methyl-1-phenylpentan-1-one (3q):**

The product was isolated by flash chromatography (PE/EA = 30/1) as white solid. Ligand = **L10**, 55 mg, 81% yield. 96% ee.

**HPLC**: Daicel Chiralcel AD-H, *n*-hexane/isopropanol 95/5, flow rate = 1.0 mL/min, l = 254 nm, *t*_R_ = 9.4 min (major), 13.8 min (minor).

**^1^H NMR** (400 MHz, CDCl_3_) δ 7.91 – 7.84 (m, 2H), 7.71 (dd, *J* = 7.4, 1.1 Hz, 1H), 7.65 (d, *J* = 7.9 Hz, 1H), 7.54 – 7.47 (m, 2H), 7.41 (dd, *J* = 8.3, 7.0 Hz, 2H), 7.36 – 7.30 (m, 2H), 7.28 – 7.22 (m, 1H), 7.18 (dd, *J* = 7.9, 1.6 Hz, 1H), 3.83 (s, 2H), 3.41 (d, *J* = 6.9 Hz, 2H), 3.23 (q, *J* = 7.2 Hz, 1H), 1.98 (h, *J* = 6.8 Hz, 1H), 1.02 (d, *J* = 6.7 Hz, 3H), 0.82 (d, *J* = 6.7 Hz, 3H).

**^13^C NMR** (101 MHz, CDCl_3_) δ 199.7, 143.2, 143.2, 142.5, 141.7, 139.8, 137.4, 132.8, 128.5, 128.1, 126.9, 126.6, 126.3, 125.1, 125.0, 119.6, 119.4, 48.1, 42.8, 36.9, 33.5, 21.1, 20.5.

**LC-MS** (ESI) *m/z* Calcd for C_25_H_25_O [M+H^+^]: 341.2; found: 341.2.

**(*R*)-4-methyl-3-(naphthalen-2-yl)-1-phenylpentan-1-one (3r):**

The product was isolated by flash chromatography (PE/EA = 30/1) as white solid. Ligand = **L10**, 56 mg, 93% yield. 96% ee.

**HPLC**: Daicel Chiralcel AD-H, *n*-hexane/isopropanol 80/20, flow rate = 1.0 mL/min, l = 254 nm, *t*_R_ = 5.3 min (major), 6.0 min (minor).

**^1^H NMR** (400 MHz, CDCl_3_) δ 7.92 – 7.85 (m, 2H), 7.80 – 7.71 (m, 3H), 7.59 (d, *J* = 1.8 Hz, 1H), 7.53 – 7.47 (m, 1H), 7.46 – 7.33 (m, 5H), 3.48 – 3.43 (m, 2H), 3.33 (td, *J* = 7.7, 5.9 Hz, 1H), 2.04 (dq, *J* = 13.7, 6.8 Hz, 1H), 1.03 (d, *J* = 6.7 Hz, 3H), 0.82 (d, *J* = 6.7 Hz, 3H).

**^13^C NMR** (101 MHz, CDCl_3_) δ 199.4, 141.3, 137.3, 133.3, 132.9, 132.2, 128.5, 128.0, 127.7, 127.7, 127.5, 126.9, 125.8, 125.2, 47.9, 42.6, 33.4, 21.0, 20.6.

**LC-MS** (ESI) *m/z* Calcd for C_22_H_23_O [M+H^+^]: 303.2; found: 303.2.

Spectroscopic data are in accordance with that reported in the literature.^6^

**(*R*)-3-(6-methoxynaphthalen-2-yl)-4-methyl-1-phenylpentan-1-one (3s):**

The product was isolated by flash chromatography (PE/EA = 10/1) as white solid. Ligand = **L10**, 36.5 mg, 55% yield. 94% ee. Under standard conditions with LiBr (1.0 eq), 56.5 mg, 85% yield and 94% ee.

**HPLC**: Daicel Chiralcel AD-H, *n*-hexane/isopropanol 80/20 flow rate = 1.0 mL/min, l = 254 nm, *t*_R_ = 6.4 min (major), 7.8 min (minor).

**^1^H NMR** (400 MHz, CDCl_3_) δ 7.91 – 7.84 (m, 2H), 7.64 (d, *J* = 8.5 Hz, 2H), 7.54 – 7.47 (m, 2H), 7.40 (dd, *J* = 8.3, 7.0 Hz, 2H), 7.32 (dd, *J* = 8.5, 1.8 Hz, 1H), 7.13 – 7.06 (m, 2H), 3.88 (s, 3H), 3.43 (d, *J* = 6.9 Hz, 2H), 3.29 (q, *J* = 7.2 Hz, 1H), 2.01 (h, *J* = 6.8 Hz, 1H), 1.02 (d, *J* = 6.6 Hz, 3H), 0.81 (d, *J* = 6.6 Hz, 3H).

**^13^C NMR** (101 MHz, CDCl_3_) δ 199.56, 157.20, 138.91, 137.34, 133.22, 132.86, 129.19, 128.82, 128.52, 128.05, 127.39, 126.71, 126.57, 118.59, 105.50, 55.32, 47.81, 42.69, 33.38, 21.07, 20.55.

**LC-MS** (ESI) *m/z* Calcd for C_23_H_25_O_2_ [M+H^+^]: 333.2; found: 333.2.

***tert*-butyl (*R*)-6-(4-methyl-1-oxo-1-phenylpentan-3-yl)-1*H*-indazole-1-carboxylate (3t):**

The product was isolated by flash chromatography (PE/EA=10/1) as colorless oil. Ligand = **L10**, 65 mg, 83% yield. 94% ee.

**HPLC**: Daicel Chiralcel AD-H, *n*-hexane/isopropanol 90/10, flow rate = 1.0 mL/min, l = 254 nm, *t*_R_ = 8.3 min (major), 9.6 min (minor).

**^1^H NMR** (400 MHz, CDCl_3_) δ 8.08 (d, *J* = 0.8 Hz, 1H), 8.04 (s, 1H), 7.90 – 7.84 (m, 2H), 7.60 (dd, *J* = 8.2, 0.7 Hz, 1H), 7.55 – 7.48 (m, 1H), 7.44 – 7.38 (m, 2H), 7.20 (dd, *J* = 8.3, 1.4 Hz, 1H), 3.45 (d, *J* = 6.9 Hz, 2H), 3.34 (q, *J* = 7.1 Hz, 1H), 2.09 – 1.97 (m, 1H), 1.71 (s, 9H), 1.03 (d, *J* = 6.7 Hz, 3H), 0.82 (d, *J* = 6.7 Hz, 3H).

**^13^C NMR** (101 MHz, CDCl_3_) δ 199.1, 149.4, 145.7, 140.1, 139.4, 137.2, 133.0, 128.5, 128.0, 125.1, 124.5, 120.5, 113.7, 84.6, 48.4, 42.7, 33.6, 28.2, 21.0, 20.7.

**LC-MS** (ESI) *m/z* Calcd for C_24_H_29_N_2_O_3_ [M+H^+^]: 393.2; found: 393.2.

**(*R*)-3-(4-methoxybenzyl)-6-(4-methyl-1-oxo-1-phenylpentan-3-yl)quinazolin-4(3*H*)-one (3u):**

The product was isolated by flash chromatography (PE/EA = 2/1) as white solid. Ligand = **L10**, 65 mg, 74% yield, 95% ee.

**HPLC**: Daicel Chiralcel AD-H, *n*-hexane/isopropanol 80/20, flow rate = 1.0 mL/min, l = 254 nm, *t*_R_ = 37.1 min (major), 39.9 min (minor).

**^1^H NMR** (400 MHz, CDCl_3_) δ 8.11 (d, *J* = 2.1 Hz, 1H), 8.05 (s, 1H), 7.88 (dt, *J* = 7.1, 1.4 Hz, 2H), 7.67 – 7.59 (m, 2H), 7.55 – 7.50 (m, 1H), 7.42 (dd, *J* = 8.3, 7.0 Hz, 2H), 7.34 – 7.28 (m, 2H), 6.90 – 6.83 (m, 2H), 5.10 (s, 2H), 3.78 (s, 3H), 3.45 (d, *J* = 6.9 Hz, 2H), 3.32 (q, *J* = 7.1 Hz, 1H), 2.00 (q, *J* = 6.8 Hz, 1H), 1.01 (d, *J* = 6.7 Hz, 3H), 0.79 (d, *J* = 6.7 Hz, 3H).

**^13^C NMR** (101 MHz, CDCl_3_) δ 198.8, 161.2, 159.6, 146.4, 145.7, 143.8, 137.0, 135.8, 133.0, 129.6, 128.6, 128.0, 127.9, 127.1, 125.0, 121.9, 114.4, 55.3, 49.2, 47.5, 42.4, 33.4, 20.9, 20.4.

**LC-MS** (ESI) *m/z* Calcd for C_28_H_29_N_2_O_3_ [M+H^+^]: 441.2; found: 441.2.

**(*S,E*)-1,3-diphenyl-5-(*p*-tolyl)pent-4-en-1-one (3v):**

The product was isolated by flash chromatography (PE/EA = 30/1) as white solid. Ligand = **L1**: 39 mg, 60% yield. 66% ee. Ligand = **L10**: 52 mg, 80% yield. 74% ee.

**HPLC**: Daicel Chiralcel OJ-H, *n*-hexane/isopropanol 80/20, flow rate = 1.0 mL/min, l = 254 nm, *t*_R_ = 17.2 min (minor), 27.8 min (major).

**^1^H NMR** (400 MHz, CDCl_3_) δ 7.96 – 7.92 (m, 2H), 7.47 – 7.40 (m, 3H), 7.34 – 7.28 (m, 4H), 7.24 – 7.16 (m, 3H), 7.06 (d, *J* = 7.9 Hz, 2H), 6.35 (d, *J* = 3.0 Hz, 2H), 4.29 (tt, *J* = 6.9, 2.9 Hz, 1H), 3.57 – 3.41 (m, 2H), 2.30 (s, 3H).

**^13^C NMR** (101 MHz, CDCl_3_) δ 198.3, 143.5, 137.2, 137.0, 134.4, 133.1, 131.6, 129.9, 129.2, 128.7, 128.6, 128.1, 127.8, 126.6, 126.2, 44.6, 44.0, 21.2.

**LC-MS** (ESI) *m/z* Calcd for C_24_H_23_O [M+H^+^]: 327.2; found: 327.2.

Spectroscopic data are in accordance with that reported in the literature.^7^

**(*R*,E)-3-isopropyl-5-(4-methoxyphenyl)-1-phenylpent-4-en-1-one (3w):**

The product was isolated by flash chromatography (PE/EA = 20/1) as white solid. Ligand = **L1**: 34 mg, 55% yield. 77:23 er. Ligand = **L7**: 47 mg, 76% yield. 94:6 er. Ligand = **L10**: 45 mg, 73% yield. 80:20 er.

**HPLC**: Daicel Chiralcel AD-H, *n*-hexane/isopropanol 90/10, flow rate = 0.8 mL/min, l = 254 nm, *t*_R_ = 8.7 min (major), 9.9 min (minor).

**^1^H NMR** (400 MHz, CDCl_3_) δ 7.92 (dd, *J* = 8.3, 1.4 Hz, 2H), 7.56 – 7.50 (m, 1H), 7.43 (dd, *J* = 8.3, 7.0 Hz, 2H), 7.24 – 7.18 (m, 2H), 6.83 – 6.76 (m, 2H), 6.27 (d, *J* = 15.8 Hz, 1H), 5.93 (dd, *J* = 15.8, 9.0 Hz, 1H), 3.77 (s, 3H), 3.16 – 2.98 (m, 2H), 2.76 (ddd, *J* = 14.1, 8.3, 5.9 Hz, 1H), 1.88 – 1.76 (m, 1H), 0.96 (dd, *J* = 11.2, 6.8 Hz, 6H).

**^13^C NMR** (101 MHz, CDCl_3_) δ 199.8, 158.8, 137.6, 132.8, 130.6, 130.4, 128.9, 128.6, 128.1, 127.2, 113.9, 55.3, 45.4, 41.7, 32.1, 20.8, 19.0.

**LC-MS** (ESI) *m/z* Calcd for C_21_H_25_O_2_ [M+H^+^]: 309.2; found: 309.2.

***tert*-butyl (R,E)-4-(1-(4-methoxyphenyl)-5-oxo-5-phenylpent-1-en-3-yl)piperidine-1-carboxylate (3x):**

The product was isolated by flash chromatography (PE/EA = 6/1) as colorless oil. Ligand = **L7**, 58 mg, 65% yield. 91:9 er.

**HPLC**: Daicel Chiralcel AD-H, *n*-hexane/isopropanol 80/20, flow rate = 0.8 mL/min, l = 254 nm, *t*_R_ = 12.5 min (minor), 14.9 min (major).

**^1^H NMR** (400 MHz, CDCl_3_) δ 7.92 (dd, *J* = 8.0, 1.4 Hz, 2H), 7.58 – 7.51 (m, 1H), 7.45 (t, *J* = 7.6 Hz, 2H), 7.24 – 7.18 (m, 2H), 6.80 (d, *J* = 8.7 Hz, 2H), 6.28 (d, *J* = 15.7 Hz, 1H), 5.90 (dd, *J* = 15.8, 9.3 Hz, 1H), 4.13 (d, *J* = 11.9 Hz, 2H), 3.78 (s, 3H), 3.12 (qd, *J* = 16.0, 6.6 Hz, 2H), 2.80 (p, *J* = 6.5 Hz, 1H), 2.65 (dtd, *J* = 15.9, 12.7, 2.6 Hz, 2H), 1.87 – 1.50 (m, 5H), 1.44 (s, 9H).

**^13^C NMR** (101 MHz, CDCl_3_) δ 199.4, 158.9, 154.8, 137.3, 133.0, 131.1, 130.0, 128.6, 128.4, 128.1, 127.5, 127.3, 113.9, 79.4, 55.3, 44.1, 44.0, 41.3, 40.3, 30.3, 29.7, 29.0, 28.5, 28.4.

**LC-MS** (ESI) *m/z* Calcd for C_28_H_36_NO_4_ [M+H^+^]: 450.3; found: 450.2.

 ***tert*-butyl (R,E)-4-(1-(2,3-dihydrobenzofuran-5-yl)-5-oxo-5-phenylpent-1-en-3-yl)piperidine-1-carboxylate (3y):**

The product was isolated by flash chromatography (PE/EA = 6/1) as light-yellow oil. Ligand = **L7**, 55 mg, 60% yield. 91:9 er.

**HPLC**: Daicel Chiralcel AD-H, *n*-hexane/isopropanol 80/20, flow rate = 0.8 mL/min, l = 254 nm, *t*_R_ = 12.9 min (minor), 17.6 min (major).

**^1^H NMR** (400 MHz, CDCl_3_) δ 7.95 – 7.89 (m, 2H), 7.58 – 7.50 (m, 1H), 7.45 (t, *J* = 7.7 Hz, 2H), 7.16 (d, *J* = 2.0 Hz, 1H), 7.00 (dd, *J* = 8.2, 1.9 Hz, 1H), 6.67 (d, *J* = 8.2 Hz, 1H), 6.25 (d, *J* = 15.7 Hz, 1H), 5.86 (dd, *J* = 15.7, 9.3 Hz, 1H), 4.53 (t, *J* = 8.7 Hz, 2H), 4.11 (q, *J* = 7.1 Hz, 2H), 3.19 – 3.12 (m, 3H), 3.06 (dd, *J* = 15.9, 7.7 Hz, 1H), 2.72 (m, 3H), 1.90 – 1.52 (m, 5H), 1.43 (s, 9H).

**^13^C NMR** (101 MHz, CDCl_3_) δ 199.4, 159.5, 154.8, 137.2, 133.0, 131.5, 130.0, 128.6, 128.1, 127.4, 126.5, 122.4, 109.1, 79.4, 71.4, 44.2, 41.4, 40.3, 29.6, 28.5.

**LC-MS** (ESI) *m/z* Calcd for C_29_H_36_NO_4_ [M+H^+^]: 462.3; found: 462.2.

 **(S)-4-methyl-1,3-diphenylpent-4-en-1-one (3z):**

The product was isolated by flash chromatography (PE/EA = 40/1) as colorless oil. Ligand = **L10**, 47.5 mg, 95% yield. 92.5:7.5 er.

**HPLC**: Daicel Chiralcel AD-H, *n*-hexane/isopropanol 97/3, flow rate = 1.0 mL/min, l = 254 nm, *t*_R_ = 6.15 min (minor), 6.8 min (major).

**^1^H NMR** (400 MHz, CDCl_3_) δ 7.96 – 7.89 (m, 2H), 7.57 – 7.51 (m, 1H), 7.48 – 7.40 (m, 2H), 7.31 – 7.24 (m, 4H), 7.20 (td, *J* = 6.0, 2.7 Hz, 1H), 4.91 – 4.83 (m, 2H), 4.05 (t, *J* = 7.3 Hz, 1H), 3.62 – 3.26 (m, 2H), 1.67 (d, *J* = 1.2 Hz, 3H).

**^13^C NMR** (101 MHz, CDCl_3_) δ 198.5, 147.1, 142.8, 137.1, 133.1, 128.6, 128.5, 128.1, 127.9, 126.6, 110.3, 47.5, 43.1, 22.2.

**LC-MS** (ESI) *m/z* Calcd for C_18_H_19_O [M+H^+^]: 251.1; found: 251.1.

 **(*R*)-3-(4-acetylphenyl)-1-(4-chlorophenyl)-3-phenyl propan-1-one (4a):**

The product was isolated by flash chromatography (PE/EA = 10/1) as white solid. Ligand = **L1**, 55 mg, 76% yield. 92% ee.

**HPLC**: Daicel Chiralcel AD-H, *n*-hexane/isopropanol 80/20, flow rate = 1.0 mL/min, l = 254 nm, *t*_R_ = 20.0 min (major), 23.8 min (minor).

**^1^H NMR** (400 MHz, CDCl_3_) δ 7.89 – 7.83 (m, 4H), 7.44 – 7.38 (m, 2H), 7.38 – 7.33 (m, 2H), 7.31 – 7.22 (m, 4H), 7.22 – 7.16 (m, 1H), 4.86 (t, *J* = 7.3 Hz, 1H), 3.82 – 3.64 (m, 2H), 2.54 (s, 3H).

**^13^C NMR** (101 MHz, CDCl_3_) δ 197.7, 196.4, 149.4, 143.1, 139.8, 135.5, 135.1, 129.5, 129.0, 128.8, 128.8, 128.1, 127.8, 126.8, 45.9, 44.3, 26.6.

**LC-MS** (ESI) *m/z* Calcd for C_23_H_20_ClO_2_ [M+H^+^]: 363.1; found: 363.1.

**(*R*)-3-(4-acetylphenyl)-1-(4-fluorophenyl)-3-phenylpropan-1-one (4b):**

The product was isolated by flash chromatography (PE/EA = 10/1) as white solid. Ligand = **L1**, 41.5 mg, 60% yield. 91% ee.

**HPLC**: Daicel Chiralcel AD-H, *n*-hexane/isopropanol 80/20, flow rate = 1.0 mL/min, l = 254 nm, *t*_R_ = 17.5 min (major), 19.7 min (minor).

**^1^H NMR** (400 MHz, CDCl_3_) δ 8.00 – 7.93 (m, 2H), 7.90 – 7.83 (m, 2H), 7.39 – 7.34 (m, 2H), 7.30 – 7.23 (m, 4H), 7.22 – 7.17 (m, 1H), 7.14 – 7.08 (m, 2H), 4.87 (t, *J* = 7.3 Hz, 1H), 3.82 – 3.65 (m, 2H), 2.55 (s, 3H).

**^13^C NMR** (101 MHz, CDCl_3_) δ 197.7, 196.0, 165.9 (d, *J* _C-F_ = 256.3 Hz), 149.5, 143.2, 135.5, 133.3 (d, *J* _C-F_ = 3.0 Hz), 130.7 (d, *J* _C-F_ = 9.3 Hz), 128.8, 128.8, 128.1, 127.8, 126.8, 115.8 (d, *J* _C-F_ = 21.8 Hz), 45.9, 44.2, 26.6.

**LC-MS** (ESI) *m/z* Calcd for C_23_H_20_FO_2_ [M+H^+^]: 347.1; found: 347.1.

**(*R*)-3-(4-acetylphenyl)-1-(naphthalen-2-yl)-3-phenyl propan-1-one (4c):**

The product was isolated by flash chromatography (PE/EA = 6/1) as white solid. Ligand = **L1**, 60 mg, 80% yield. 90% ee.

**HPLC**: Daicel Chiralcel AD-H, *n*-hexane/isopropanol 80/20, flow rate = 1.0 mL/min, l = 254 nm, *t*_R_ = 30.0 min (major), 31.5 min (minor).

**^1^H NMR** (400 MHz, CDCl_3_) δ 8.48 – 8.45 (m, 1H), 7.98 (dd, *J* = 8.6, 1.8 Hz, 1H), 7.94 (dd, *J* = 8.1, 1.4 Hz, 1H), 7.87 (ddd, *J* = 7.2, 3.9, 1.5 Hz, 4H), 7.60 (ddd, *J* = 8.2, 6.8, 1.4 Hz, 1H), 7.55 (ddd, *J* = 8.2, 6.9, 1.4 Hz, 1H), 7.43 – 7.38 (m, 2H), 7.30 (d, *J* = 5.1 Hz, 4H), 7.20 (ddt, *J* = 6.2, 4.6, 3.1 Hz, 1H), 4.95 (t, *J* = 7.3 Hz, 1H), 3.99 – 3.82 (m, 2H), 2.54 (s, 3H).

**^13^C NMR** (101 MHz, CDCl_3_) δ 197.8, 197.5, 149.7, 143.4, 135.7, 135.4, 134.2, 132.5, 129.8, 129.6, 128.8, 128.8, 128.7, 128.6, 128.2, 127.9, 127.8, 126.9, 126.8, 123.8, 46.0, 44.4, 26.6.

**LC-MS** (ESI) *m/z* Calcd for C_27_H_23_O_2_ [M+H^+^]:379.2; found: 379.2.

**(*R*)-3-(4-acetylphenyl)-3-phenyl-1-(pyridin-3-yl) propan-1-one (4d):**

The product was isolated by flash chromatography (PE/EA = 2/1) as white solid. Ligand = **L1**, 55 mg, 84% yield. 87% ee.

**HPLC**: Daicel Chiralcel OD-H, *n*-hexane/isopropanol 70/30, flow rate = 1.0 mL/min, l = 254 nm, *t*_R_ = 30.0 min (minor), 57.2 min (major).

**^1^H NMR** (400 MHz, CDCl_3_) δ 9.16 (d, *J* = 2.3 Hz, 1H), 8.77 (dd, *J* = 4.9, 1.7 Hz, 1H), 8.18 (dt, *J* = 8.0, 2.0 Hz, 1H), 7.91 – 7.85 (m, 2H), 7.44 – 7.39 (m, 1H), 7.39 – 7.35 (m, 2H), 7.33 – 7.28 (m, 2H), 7.26 (d, *J* = 6.8 Hz, 2H), 7.24 – 7.18 (m, 1H), 4.88 (t, *J* = 7.3 Hz, 1H), 3.86 – 3.70 (m, 2H), 2.55 (s, 3H).

**^13^C NMR** (101 MHz, CDCl_3_) δ 197.7, 196.5, 153.7, 149.5, 149.2, 142.8, 135.6, 135.5, 132.1, 128.9, 128.8, 128.0, 127.7, 127.0, 123.8, 45.8, 44.6, 26.6.

**LC-MS** (ESI) *m/z* Calcd for C_22_H_20_NO_2_ [M+H^+^]: 330.1; found: 330.1.

**(*S*)-3-(4-acetylphenyl)-3-(furan-2-yl)-1-phenylpropan-1-one (4e):**

The product was isolated by flash chromatography (PE/EA = 10/1) as white solid. Ligand = **L1**, 44 mg, 70% yield. 94% ee.

**HPLC**: Daicel Chiralcel IG, *n*-hexane/isopropanol 85/15, flow rate = 1.0 mL/min, l = 254 nm, *t*_R_ = 35.9 min (major), 38.1 min (minor).

**^1^H NMR** (400 MHz, CDCl_3_) δ 7.97 – 7.91 (m, 2H), 7.91 – 7.86 (m, 2H), 7.58 – 7.54 (m, 1H), 7.48 – 7.39 (m, 4H), 7.32 (dd, *J* = 1.9, 0.8 Hz, 1H), 6.28 (dd, *J* = 3.2, 1.9 Hz, 1H), 6.06 (dt, *J* = 3.2, 0.9 Hz, 1H), 4.90 (t, *J* = 7.2 Hz, 1H), 3.82 (dd, *J* = 17.4, 6.6 Hz, 1H), 3.61 (dd, *J* = 17.4, 7.7 Hz, 1H), 2.56 (s, 3H).

**^13^C NMR** (101 MHz, CDCl_3_) δ 197.8, 197.1, 155.8, 147.4, 141.9, 141.9, 136.6, 135.8, 133.4, 128.8, 128.7, 128.2, 128.1, 110.3, 106.2, 43.1, 40.1, 26.6.

**LC-MS** (ESI) *m/z* Calcd for C_21_H_19_O_3_ [M+H^+^]: 319.1; found: 319.1.

**(*S*)-3-(4-acetylphenyl)-1-phenyl-3-(thiophen-3-yl)propan-1-one (4f):**

The product was isolated by flash chromatography (PE/EA = 10/1) as white solid. Ligand = **L1**, 48 mg, 72% yield. 88% ee.

**HPLC**: Daicel Chiralcel AD-H, *n*-hexane/isopropanol 80/20, flow rate = 1.0 mL/min, l = 254 nm, *t*_R_ = 17.5 min (major), 19.7 min (minor).

**^1^H NMR** (400 MHz, CDCl_3_) δ 7.95 – 7.91 (m, 2H), 7.90 – 7.85 (m, 2H), 7.59 – 7.53 (m, 1H), 7.45 (dd, *J* = 8.4, 7.0 Hz, 2H), 7.40 – 7.35 (m, 2H), 7.26 – 7.23 (m, 1H), 6.99 (dt, *J* = 2.4, 1.0 Hz, 1H), 6.91 (dd, *J* = 5.0, 1.4 Hz, 1H), 4.93 (t, *J* = 7.2 Hz, 1H), 3.82 – 3.65 (m, 2H), 2.55 (s, 3H).

**^13^C NMR** (101 MHz, CDCl_3_) δ 197.8, 197.5, 149.4, 144.0, 136.8, 135.6, 133.4, 128.8, 128.7, 128.1, 128.1, 127.5, 126.2, 120.8, 44.8, 41.7, 26.6.

**LC-MS** (ESI) *m/z* Calcd for C_21_H_19_O_2_S [M+H^+^]: 335.1; found: 335.0.

**(*S*)-3-(4-acetylphenyl)-3-(4-methoxyphenyl)-1-phenyl propan-1-one (4g):**

The product was isolated by flash chromatography (PE/EA = 6/1) as white solid. Ligand = **L1**, 70 mg, 95% yield. 90% ee.

**HPLC**: Daicel Chiralcel AD-H, *n*-hexane/isopropanol 80/20, flow rate = 1.0 mL/min, l = 254 nm, *t*_R_ = 24.8 min (minor), 27.2 min (major).

**^1^H NMR** (400 MHz, CDCl_3_) δ 7.97 – 7.91 (m, 2H), 7.89 – 7.84 (m, 2H), 7.59 – 7.53 (m, 1H), 7.45 (dd, *J* = 8.4, 7.0 Hz, 2H), 7.38 – 7.34 (m, 2H), 7.20 – 7.13 (m, 2H), 6.85 – 6.79 (m, 2H), 4.83 (t, *J* = 7.3 Hz, 1H), 3.75 – 3.66 (m, 2H), 2.55 (s, 3H).

**^13^C NMR** (101 MHz, CDCl_3_) δ 197.8, 197.7, 158.3, 150.1, 144.4, 136.8, 136.5, 135.4, 135.3, 133.3, 128.7, 128.1, 128.0, 114.1, 55.3, 45.1, 44.5, 26.6.

**LC-MS** (ESI) *m/z* Calcd for C_24_H_23_O_3_ [M+H^+^]: 359.2; found: 359.2.

**(*S*)-3-(4-acetylphenyl)-1-phenylpentan-1-one (4h):**

The product was isolated by flash chromatography (PE/EA = 10/1) as colorless oil. Ligand = **L10**, 45 mg, 80% yield. 95% ee.

**HPLC**: Daicel Chiralcel AD-H, *n*-hexane/isopropanol 90/10, flow rate = 1.0 mL/min, l = 254 nm, *t*_R_ = 15.6 min (major), 19.9 min (minor).

**^1^H NMR** (400 MHz, CDCl_3_) δ 7.92 – 7.86 (m, 4H), 7.57 – 7.50 (m, 1H), 7.46 – 7.40 (m, 2H), 7.36 – 7.31 (m, 2H), 3.39 – 3.26 (m, 3H), 2.57 (s, 3H), 1.87 – 1.77 (m, 1H), 1.70 – 1.62 (m, 1H), 0.81 (t, *J* = 7.4 Hz, 3H).

**^13^C NMR** (101 MHz, CDCl_3_) δ 198.6, 197.9, 150.6, 137.0, 135.4, 133.1, 128.6, 128.0, 127.9, 45.1, 42.9, 29.2, 26.6, 12.1.

**LC-MS** (ESI) *m/z* Calcd for C_19_H_21_O_2_ [M+H^+^]: 281.1; found: 281.1.

 **(*S*)-3-(4-acetylphenyl)-1,5-diphenylpentan-1-one (4i):**

The product was isolated by flash chromatography (PE/EA = 10/1) as white solid. Ligand = **L10**, 57 mg, 80% yield. 97% ee.

**HPLC**: Daicel Chiralcel AD-H, *n*-hexane/isopropanol 90/10, flow rate = 1.0 mL/min, l = 254 nm, *t*_R_ = 23.3 min (major), 27.6 min (minor).

**^1^H NMR** (400 MHz, CDCl_3_) δ 7.96 – 7.90 (m, 2H), 7.90 – 7.83 (m, 2H), 7.58 – 7.50 (m, 1H), 7.46 – 7.34 (m, 4H), 7.28 – 7.22 (m, 2H), 7.20 – 7.14 (m, 1H), 7.09 (dd, *J* = 7.0, 1.7 Hz, 2H), 3.54 – 3.43 (m, 1H), 3.38 – 3.23 (m, 2H), 2.59 (s, 3H), 2.48 (dt, *J* = 9.3, 6.0 Hz, 2H), 2.12 (dddd, *J* = 14.4, 9.6, 7.2, 4.8 Hz, 1H), 1.99 (dtd, *J* = 13.4, 9.7, 5.6 Hz, 1H).

**^13^C NMR** (101 MHz, CDCl_3_) δ 198.3, 197.9, 150.2, 141.6, 136.9, 135.6, 133.2, 128.8, 128.6, 128.4, 128.3, 128.0, 125.9, 45.5, 40.9, 37.8, 33.7, 26.6.

**LC-MS** (ESI) *m/z* Calcd for C_25_H_25_O_2_ [M+H^+^]: 357.2; found: 357.2.

**(*S*)-2-(3-(4-acetylphenyl)-5-oxo-5-phenylpentyl) isoindoline -1,3-dione (4j):**

The product was isolated by flash chromatography (PE/EA = 3/1) as white solid. Ligand = **L10**, 75 mg, 88% yield. 97% ee.

**HPLC**: Daicel Chiralcel AD-H, *n*-hexane/isopropanol 70/30, flow rate = 0.8 mL/min, l = 254 nm, *t*_R_ = 42.9 min (minor), 47.6 min (major).

**^1^H NMR** (400 MHz, CDCl_3_) δ 7.89 – 7.83 (m, 2H), 7.81 – 7.76 (m, 2H), 7.74 (dd, *J* = 5.5, 3.1 Hz, 2H), 7.65 (dd, *J* = 5.5, 3.1 Hz, 2H), 7.56 – 7.49 (m, 1H), 7.41 (t, *J* = 7.7 Hz, 2H), 7.39 – 7.34 (m, 2H), 3.65 (t, *J* = 6.9 Hz, 2H), 3.57 – 3.47 (m, 1H), 3.30 (d, *J* = 6.9 Hz, 2H), 2.48 (s, 3H), 2.25 (ddt, *J* = 14.3, 10.1, 7.2 Hz, 1H), 2.14 (dtd, *J* = 13.6, 6.6, 4.5 Hz, 1H).

**^13^C NMR** (101 MHz, CDCl_3_) δ 197.8, 197.5, 168.2, 149.2, 136.8, 135.4, 133.8, 133.2, 131.9, 128.7, 128.6, 128.0, 127.8, 123.1, 45.5, 39.1, 36.5, 33.7, 26.5.

**LC-MS** (ESI) *m/z* Calcd for C_27_H_24_NO_4_ [M+H^+^]: 426.2; found: 426.2.

 **(*S*)-3-(4-acetylphenyl)-1-phenyldodecan-1-one (4k):**

The product was isolated by flash chromatography (PE/EA = 10/1) as white solid. Ligand = **L10**, 57 mg, 75% yield. 95% ee.

**HPLC**: Daicel Chiralcel AD-H, *n*-hexane/isopropanol 95/5, flow rate = 1.0 mL/min, l = 254 nm, *t*_R_ = 13.6 min (major), 17.7 min (minor).

**^1^H NMR** (400 MHz, CDCl_3_) δ 7.94 – 7.82 (m, 4H), 7.56 – 7.52 (m, 1H), 7.43 (dd, *J* = 8.4, 6.9 Hz, 2H), 7.36 – 7.29 (m, 2H), 3.47 – 3.36 (m, 1H), 3.29 (dd, *J* = 6.9, 3.3 Hz, 2H), 2.57 (s, 3H), 1.79 – 1.70 (m, 1H), 1.66 – 1.59 (m, 1H), 1.28 – 1.15 (m, 14H), 0.86 (t, *J* = 6.9 Hz, 3H).

**^13^C NMR** (101 MHz, CDCl_3_) δ 198.6, 197.9, 150.9, 137.0, 135.4, 133.1, 128.6, 128.6, 128.0, 127.9, 45.4, 41.2, 36.3, 31.9, 29.5, 29.5, 29.3, 27.5, 26.6, 22.7, 14.1.

**LC-MS** (ESI) *m/z* Calcd for C_26_H_35_O_2_ [M+H^+^]: 379.3; found: 379.2.

**(*S*)-3-(4-acetylphenyl)-5,5-dimethyl-1-phenylhexan-1-one (4l):**

The product was isolated by flash chromatography (PE/EA = 20/1) as white solid. Ligand = **L10**, 51.5 mg, 80% yield. 62% ee.

**HPLC**: Daicel Chiralcel AD-H, *n*-hexane/isopropanol 80/20, flow rate = 1.0 mL/min, l = 254 nm, *t*_R_ = 7.0 min (major), 9.6 min (minor).

**^1^H NMR** (400 MHz, CDCl_3_) δ 7.89 – 7.84 (m, 4H), 7.56 – 7.50 (m, 1H), 7.42 (dd, *J* = 8.3, 7.0 Hz, 2H), 7.40 – 7.36 (m, 2H), 3.59 (dp, *J* = 10.0, 3.5 Hz, 1H), 3.23 (dd, *J* = 7.0, 1.3 Hz, 2H), 2.57 (s, 3H), 1.82 (dd, *J* = 14.0, 9.4 Hz, 1H), 1.65 (dd, *J* = 14.0, 3.3 Hz, 1H), 0.79 (s, 9H).

**^13^C NMR** (101 MHz, CDCl_3_) δ 198.5, 197.9, 152.6, 137.0, 135.3, 133.1, 128.6, 128.6, 128.1, 128.0, 49.6, 48.0, 38.1, 31.5, 30.1, 26.6.

**LC-MS** (ESI) *m/z* Calcd for C_22_H_27_O_2_ [M+H^+^]: 323.2; found: 323.2

 **(*R*)-3-(4-acetylphenyl)-3-cyclopropyl-1-phenylpropan-1-one (4m):**

The product was isolated by flash chromatography (PE/EA = 10/1) as white solid. Ligand = **L10**, 54 mg, 93% yield. 94% ee.

**HPLC**: Daicel Chiralcel AD-H, *n*-hexane/isopropanol 80/20, flow rate = 1.0 mL/min, l = 254 nm, *t*_R_ = 11.0 min (major), 13.1min (minor).

**^1^H NMR** (400 MHz, CDCl_3_) δ 7.95 – 7.86 (m, 4H), 7.58 – 7.52 (m, 1H), 7.48 – 7.41 (m, 2H), 7.41 – 7.35 (m, 2H), 3.56 – 3.41 (m, 2H), 2.69 (ddd, *J* = 9.9, 8.0, 5.9 Hz, 1H), 2.57 (s, 3H), 1.10 (dtt, *J* = 9.8, 7.9, 4.9 Hz, 1H), 0.58 (dddd, *J* = 9.0, 7.9, 5.6, 4.3 Hz, 1H), 0.45 (dddd, *J* = 9.2, 7.9, 5.4, 4.4 Hz, 1H), 0.28 (dq, *J* = 9.7, 4.9 Hz, 1H), 0.22 – 0.14 (m, 1H).

**^13^C NMR** (101 MHz, CDCl_3_) δ 198.6, 197.9, 150.6, 137.1, 135.4, 133.1, 128.6, 128.1, 127.7, 46.3, 45.2, 26.6, 17.5, 5.6, 4.5.

**LC-MS** (ESI) *m/z* Calcd for C_20_H_21_O_2_ [M+H^+^]: 293.2; found: 293.2.

 **(*R*)-3-(4-acetylphenyl)-3-cyclohexyl-1-phenylpropan-1-one (4n):**

The product was isolated by flash chromatography (PE/EA = 10/1) as white solid. Ligand = **L10**, 65 mg, 97% yield. 93% ee.

**HPLC**: Daicel Chiralcel AD-H, *n*-hexane/isopropanol 80/20, flow rate = 1.0 mL/min, l = 254 nm, *t*_R_ = 9.5 min (major), 11.4 min (minor).

**^1^H NMR** (400 MHz, CDCl_3_) δ 7.86 (td, *J* = 8.2, 1.6 Hz, 4H), 7.57 – 7.49 (m, 1H), 7.47 – 7.38 (m, 2H), 7.30 – 7.25 (m, 2H), 3.50 – 3.30 (m, 2H), 3.26 (td, *J* = 8.2, 5.0 Hz, 1H), 2.56 (s, 3H), 1.94 – 1.72 (m, 2H), 1.68 – 1.53 (m, 3H), 1.45 (ddd, *J* = 12.2, 4.9, 2.5 Hz, 1H), 1.28 – 0.98 (m, 4H), 0.85 (qd, *J* = 12.2, 3.4 Hz, 1H).

**^13^C NMR** (101 MHz, CDCl_3_) δ 199.0, 197.9, 149.9, 137.1, 135.2, 133.0, 128.6, 128.3, 128.0, 47.0, 43.0, 42.0, 31.3, 30.9, 26.6, 26.5, 26.3, 26.3.

**LC-MS** (ESI) *m/z* Calcd for C_22_H_27_O_2_ [M+H^+^]: 335.2; found: 335.2.

***tert*-butyl (*R*)-4-(1-(4-acetylphenyl)-3-oxo-3-phenylpropyl) piperidine-1-carboxylate (4o):**

The product was isolated by flash chromatography (PE/EA = 4/1) as colorless oil. Ligand = **L10**, 73 mg, 84% yield. 92% ee.

**HPLC**: Daicel Chiralcel AD-H, *n*-hexane/isopropanol 80/20, flow rate = 1.0 mL/min, l = 254 nm, *t*_R_ = 18.3 min (major), 23.4 min (minor).

**^1^H NMR** (400 MHz, CDCl_3_) δ 7.90 – 7.83 (m, 4H), 7.57 – 7.51 (m, 1H), 7.43 (dd, *J* = 8.4, 7.0 Hz, 2H), 7.28 (d, *J* = 8.3 Hz, 2H), 4.09 (d, *J* = 40.4 Hz, 2H), 3.52 – 3.34 (m, 2H), 3.30 (td, *J* = 7.9, 5.5 Hz, 1H), 2.77 – 2.61 (m, 1H), 2.56 (s, 4H), 1.81 (dd, *J* = 23.5, 10.0 Hz, 2H), 1.73 (ddt, *J* = 11.5, 7.1, 3.4 Hz, 1H), 1.43 (s, 9H), 1.25 – 0.99 (m, 2H).

**^13^C NMR** (101 MHz, CDCl_3_) δ 198.4, 197.8, 154.7, 148.9, 136.9, 135.5, 133.2, 128.6, 128.5(3 overlapping signals), 128.0, 79.4, 46.3, 43.9, 41.7, 41.4, 30.3, 28.4, 26.6.

**LC-MS** (ESI) *m/z* Calcd for C_27_H_34_NO_4_ [M+H^+^]: 436.2; found: 436.2.

**(3*R*)-3-(4-acetylphenyl)-3-(cyclohex-3-en-1-yl)-1-phenyl propan-1-one (4p):**

The product was isolated by flash chromatography (PE/EA = 10/1) as white solid. Ligand = **L10**, 50 mg, 75% yield. 93% ee. 93% ee. 1:1 dr.

**HPLC**: Daicel Chiralcel AD-H, *n*-hexane/isopropanol 80/20, flow rate = 1.0 mL/min, l = 254 nm, *t*_R_ = 12.6 min (major), 13.9+15.3 min (minor).

**^1^H NMR** (400 MHz, CDCl_3_) δ 7.87 (ddt, *J* = 8.9, 8.0, 1.4 Hz, 4H), 7.54 (tt, *J* = 7.3, 1.3 Hz, 1H), 7.42 (td, *J* = 7.7, 1.7 Hz, 2H), 7.33 – 7.28 (m, 2H), 5.67 – 5.51 (m, 2H), 3.52 – 3.35 (m, 2H), 3.32 (ddd, *J* = 9.3, 4.6, 2.0 Hz, 1H), 2.56 (s, 3H), 2.14 – 1.86 (m, 5H), 1.68 (s, 2H).

**^13^C NMR** (101 MHz, CDCl_3_) δ 198.9, 197.9, 149.6, 137.0, 135.4, 133.1, 128.6, 128.6, 128.4, 128.0, 127.0, 126.0, 46.3, 42.2, 39.0, 30.0, 26.9, 26.6, 25.5.

**LC-MS** (ESI) *m/z* Calcd for C_23_H_25_O_2_ [M+H^+^]: 333.2; found: 333.2.

**(3*R*)-3-(4-acetylphenyl)-5-(4-isopropylphenyl)-4-methyl-1-phenylpentan-1-one (4q):**

The product was isolated by flash chromatography (PE/EA = 10/1) as colorless oil. Ligand = **L10**, 57.5 mg, 70% yield. 1.4:1 dr, 54% ee, 95% ee. Racemate: 1:2 dr according to ^1^H-NMR.

**HPLC**: Daicel Chiralcel AD-H, *n*-hexane/isopropanol 95/5, flow rate = 1.0 mL/min, l = 254 nm, *t*_R_ = 21.1 min (major), 26.7 min (minor); 25.3 min (major), 31.1 min (minor).

**^1^H NMR** (400 MHz, CDCl_3_) including two isomers δ 7.93 – 7.83 (m, 4H), 7.56 – 7.51 (m, 1H), 7.42 (td, *J* = 7.8, 2.0 Hz, 2H), 7.38 – 7.30 (m, 2H), 7.11 (dd, *J* = 9.3, 7.2 Hz, 2H), 7.06 – 6.95 (m, 2H), 3.63 – 3.35 (m, 3H), 2.93 – 2.60 (m, 2H), 2.57 (d, *J* = 3.7 Hz, 3H), 2.29 – 2.03 (m, 2H), 1.23 (dd, *J* = 6.9, 3.2 Hz, 6H), 0.83 (d, *J* = 6.3 Hz, 3H).

**^13^C NMR** (101 MHz, CDCl_3_) including two isomers: δ 198.8, 197.9, 152.3, 149.7, 148.7, 146.5, 146.5, 138.1, 137.9, 137.1, 137.0, 135.4, 135.4, 133.2, 133.1, 133.1, 129.0, 128.9, 128.9, 128.8, 128.7, 128.6, 128.6, 128.5, 128.3, 128.1, 128.0, 127.2, 126.4, 126.3, 46.4, 45.9, 42.2, 41.2, 40.9, 40.6, 40.4, 39.8, 35.5, 33.7, 26.6, 24.1, 17.0, 16.5.

**LC-MS** (ESI) *m/z* Calcd for C_29_H_33_O_2_ [M+H^+^]: 413.2; found: 413.2.

**(*R*)-3-(4-acetylphenyl)-1-(4-methoxyphenyl)-4-methylpentan-1-one (4r):**

The product was isolated by flash chromatography (PE/EA = 6/1) as white solid. Ligand = **L10**, 60 mg, 93% yield. 94% ee.

**HPLC**: Daicel Chiralcel AD-H, *n*-hexane/isopropanol 80/20, flow rate = 1.0 mL/min, l = 254 nm, *t*_R_ = 19.0 min (major), 32.6 min (minor).

**^1^H NMR** (400 MHz, CDCl_3_) δ 7.89 – 7.82 (m, 4H), 7.28 (dd, *J* = 8.0, 1.5 Hz, 2H), 6.92 – 6.86 (m, 2H), 3.85 (s, 3H), 3.36 – 3.31 (m, 2H), 3.22 (td, *J* = 7.6, 6.1 Hz, 1H), 2.55 (s, 3H), 2.02 – 1.90 (m, 1H), 1.00 (d, *J* = 6.7 Hz, 3H), 0.79 (d, *J* = 6.7 Hz, 3H).

**^13^C NMR** (101 MHz, CDCl_3_) δ 197.9, 197.4, 163.4, 149.8, 135.2, 130.3, 130.2, 128.6, 128.3, 113.7, 55.5, 48.0, 41.7, 33.2, 26.6, 20.9, 20.5.

**LC-MS** (ESI) *m/z* Calcd for C_21_H_25_O_3_ [M+H^+^]: 325.2; found: 325.2.

**(*R*)-1-(4-acetylphenyl)-4,4-dimethyl-1-phenylpentan-3-one (4s):**

The product was isolated by flash chromatography (PE/EA = 10/1) as white solid. Ligand = **L10**, 49 mg, 80% yield. 82% ee.

**HPLC**: Daicel Chiralcel AD-H, *n*-hexane/isopropanol 80/20, flow rate = 1.0 mL/min, l = 254 nm, *t*_R_ = 6.2 min (major), 7.0 min (minor).

**^1^H NMR** δ 7.90 – 7.83 (m, 2H), 7.36 – 7.31 (m, 2H), 7.31 – 7.26 (m, 2H), 7.23 – 7.17 (m, 3H), 4.71 (t, *J* = 7.3 Hz, 1H), 3.36 – 3.17 (m, 2H), 2.56 (s, 3H), 1.05 (s, 9H).

**^13^C NMR** (101 MHz, CDCl_3_) δ 213.0, 197.8, 149.9, 143.4, 135.3, 128.7 (overlapped two signals), 128.1, 127.8, 126.7, 45.6, 44.2, 42.6, 26.6, 26.1.

**LC-MS** (ESI) *m/z* Calcd for C_21_H_25_O_2_ [M+H^+^]: 309.2; found: 309.2.

 **(3R)-3-(4-acetylphenyl)-1-((1s,3S)-adamantan-1-yl)-3-phenylpropan-1-one (4t):**

The product was isolated by flash chromatography (PE/EA = 10/1) as white solid. Ligand = **L10**, 60 mg, 78% yield. 84% ee.

**HPLC**: Daicel Chiralcel AD-H, *n*-hexane/isopropanol 80/20, flow rate = 1.0 mL/min, l = 254 nm, *t*_R_ = 18.6 min (major), 20.1 min (minor).

**^1^H NMR** (400 MHz, CDCl_3_) δ 7.89 – 7.83 (m, 2H), 7.35 – 7.30 (m, 2H), 7.29 – 7.25 (m, 2H), 7.20 (dt, *J* = 8.3, 2.1 Hz, 3H), 4.71 (t, *J* = 7.3 Hz, 1H), 3.36 – 3.12 (m, 2H), 2.56 (s, 3H), 2.05 – 1.96 (m, 3H), 1.69 (m, 12H).

**^13^C NMR** (101 MHz, CDCl_3_) δ 212.7, 197.8, 150.0, 143.5, 135.3, 128.7, 128.1, 127.8, 126.6, 46.4, 45.3, 42.1, 38.0, 36.5, 27.8, 26.6.

**LC-MS** (ESI) *m/z* Calcd for C_27_H_31_O_2_ [M+H^+^]: 387.2; found: 387.2.

 **(*R*)-1-(4-acetylphenyl)-4-methyl-1-phenylpentan-3-one (4u):**

The product was isolated by flash chromatography (PE/EA = 20/1) as colorless oil. Ligand = **L10**, 41 mg, 70% yield. 70% ee.

**HPLC**: Daicel Chiralcel AD-H, *n*-hexane/isopropanol 80/20, flow rate = 1.0 mL/min, l = 254 nm, *t*_R_ = 18.6 min (major), 20.1 min (minor).

**^1^H NMR** (400 MHz, CDCl_3_) δ 7.89 – 7.84 (m, 2H), 7.35 – 7.31 (m, 2H), 7.31 – 7.26 (m, 2H), 7.20 (dt, *J* = 7.8, 1.4 Hz, 3H), 4.69 (t, *J* = 7.4 Hz, 1H), 3.23 (dd, *J* = 7.4, 4.1 Hz, 2H), 2.56 (s, 3H), 2.55 – 2.48 (m, 1H), 1.00 (dd, *J* = 6.9, 0.7 Hz, 6H).

**^13^C NMR** (101 MHz, CDCl_3_) δ 212.1, 197.9, 149.7, 143.2, 135.4, 128.7, 128.0, 127.7, 126.7, 46.1, 45.7, 41.4, 26.6, 17.9.

**LC-MS** (ESI) *m/z* Calcd for C_20_H_23_O_2_ [M+H^+^]: 295.2; found: 295.2.

 ***tert*-butyl (*R*)-4-(3-(4-acetylphenyl)-3-phenylpropanoyl)piperidine-1-carboxylate (4v):**

The product was isolated by flash chromatography (PE/EA = 4/1) as colorless oil. Ligand = **L10**, 52 mg, 60% yield. 76: 24 er.

**HPLC**: Daicel Chiralcel IA-H, *n*-hexane/isopropanol 90/10, flow rate = 0.8 mL/min, l = 254 nm, *t*_R_ = 28.2 min (major), 29.4 min (minor).

**^1^H NMR** (400 MHz, CDCl_3_) δ 7.80 (d, *J* = 8.0 Hz, 2H), 7.24 (d, *J* = 8.4 Hz, 2H), 7.21 – 7.18 (m, 2H), 7.15 – 7.10 (m, 3H), 4.61 (t, *J* = 7.4 Hz, 1H), 3.96 (s, 2H), 3.17 (d, *J* = 7.4 Hz, 2H), 2.64 (t, *J* = 12.1 Hz, 2H), 2.49 (s, 3H), 2.31 (s, 1H), 1.80 (d, *J* = 8.5 Hz, 2H), 1.62 (d, *J* = 13.0 Hz, 2H), 1.36 (s, 9H).

**^13^C NMR** (101 MHz, CDCl_3_) δ 209.6, 197.8, 154.6, 149.5, 143.0, 135.4, 128.8, 128.8, 128.0, 127.7, 127.1, 126.8, 79.7, 49.1, 46.4, 45.5, 28.4, 27.1, 26.6.

**LC-MS** (ESI) *m/z* Calcd for C_27_H_33_NNaO_4_ [M+Na^+^]: 458.2; found: 458.2.

**(3a*R*,5*R*,6*S*,6a*R*)-5-((*S*)-2,2-dimethyl-1,3-dioxolan-4-yl)-2,2-dimethyltetrahydrofuro[2,3-*d*][1,3]dioxol-6-yl 4-((*R*)-4-methyl-1-oxo-1-phenylpentan-3-yl)benzoate (5a):**

The product was isolated by flash chromatography (PE/EA = 6/1) as sticky colorless oil. Ligand = **L10**, 86 mg, 80% yield. 97% de.

**HPLC**: Daicel Chiralcel AD-H, *n*-hexane/isopropanol 90/10, flow rate = 1.0 mL/min, l = 254 nm, *t*_R_ = 25.8 min (minor), 27.8 min (major).

**^1^H NMR** (400 MHz, CDCl_3_) δ 7.95 – 7.84 (m, 4H), 7.57 – 7.51 (m, 1H), 7.43 (dd, *J* = 8.4, 7.0 Hz, 2H), 7.28 (d, *J* = 8.5 Hz, 2H), 5.92 (d, *J* = 3.6 Hz, 1H), 5.46 (d, *J* = 2.6 Hz, 1H), 4.59 (d, *J* = 3.7 Hz, 1H), 4.39 – 4.28 (m, 2H), 4.14 – 4.04 (m, 2H), 3.39 (dd, *J* = 7.0, 1.9 Hz, 2H), 3.23 (q, *J* = 7.3 Hz, 1H), 2.01 – 1.90 (m, 1H), 1.55 (s, 3H), 1.41 (s, 3H), 1.31 (s, 3H), 1.27 (s, 3H), 1.01 (d, *J* = 6.7 Hz, 3H), 0.79 (d, *J* = 6.6 Hz, 3H).

**^13^C NMR** (101 MHz, CDCl_3_) δ 198.8, 165.2, 150.1, 137.0, 133.1, 129.6, 128.6, 128.6, 128.0, 127.4, 112.3, 109.4, 105.1, 83.4, 79.9, 76.5, 72.6, 67.2, 47.9, 42.1, 33.2, 26.9, 26.7, 26.2, 25.2, 20.8, 20.5.

**LC-MS** (ESI) *m/z* Calcd for C_31_H_39_O_8_ [M+H^+^]: 539.3; found: 539.3.

**(*R*)-2,5,7,8-tetramethyl-2-((4*R*,8*R*)-4,8,12-trimethyltridecyl)chroman-6-yl 4-((*R*)-4-methyl-1-oxo-1-phenylpentan-3-yl)benzoate (5b):**

The product was isolated by flash chromatography (PE/EA = 20/1) as colorless oil. Ligand = **L10**, 120 mg, 85% yield. 94% de.

**HPLC**: Daicel Chiralcel AD-H, *n*-hexane/isopropanol 98/2, flow rate = 1.0 mL/min, l = 254 nm, *t*_R_ = 11.3 min (major), 12.9 min (minor).

**^1^H NMR** (400 MHz, CDCl_3_) δ 8.15 – 8.08 (m, 2H), 7.93 – 7.86 (m, 2H), 7.56 – 7.50 (m, 1H), 7.44 (dd, *J* = 8.4, 6.9 Hz, 2H), 7.37 – 7.31 (m, 2H), 3.50 – 3.35 (m, 2H), 3.28 (td, *J* = 7.9, 5.7 Hz, 1H), 2.61 (t, *J* = 6.8 Hz, 2H), 2.11 (s, 3H), 2.04 (s, 3H), 2.00 (s, 3H), 1.80 (td, *J* = 12.3, 11.0, 6.6 Hz, 2H), 1.61 – 1.46 (m, 4H), 1.39 (dt, *J* = 10.9, 5.0 Hz, 3H), 1.32 – 1.20 (m, 12H), 1.18 – 1.05 (m, 6H), 1.03 (d, *J* = 6.7 Hz, 3H), 0.85 (dd, *J* = 11.7, 6.7 Hz, 15H).

**^13^C NMR** (101 MHz, CDCl_3_) δ 198.9, 165.2, 150.0, 149.4, 140.6, 137.1, 133.1, 130.1, 128.6, 128.6, 128.0, 127.6, 127.0, 125.2, 123.1, 117.5, 75.1, 47.8, 42.1, 39.4, 37.5, 37.3, 33.3, 32.8, 28.0, 24.8, 24.5, 22.8, 22.7, 21.1, 20.9, 20.7, 20.5, 19.8, 19.7, 13.1, 12.3, 11.9.

**LC-MS** (ESI) *m/z* Calcd for C_48_H_69_O_4_ [M+H^+^]: 709.5; found: 709.5.

**(8*R*,9*S*,13*S*,14*S*)-13-methyl-17-oxo-7,8,9,11,12,13,14,15,16,17-decahydro-6*H*-cyclopenta[*a*]phenanthren-3-yl 4-((*R*)-4-methyl-1-oxo-1-phenylpentan-3-yl)benzoate (5c):**

The product was isolated by flash chromatography (PE/EA = 6/1) as colorless oil. Ligand = **L10**, 71 mg, 65% yield. 97% ee.

**HPLC**: Daicel Chiralcel OJ-H, *n*-hexane/EtOH 60/40, flow rate = 0.8 mL/min, l = 240 & 250 nm, *t*_R_ = 48.7 min (minor), 89.4 min (major).

**^1^H NMR** (400 MHz, CDCl_3_) δ 8.12 – 8.04 (m, 2H), 7.92 – 7.85 (m, 2H), 7.59 – 7.50 (m, 1H), 7.43 (dd, *J* = 8.4, 7.0 Hz, 2H), 7.36 – 7.28 (m, 3H), 6.95 (dd, *J* = 8.4, 2.6 Hz, 1H), 6.91 (d, *J* = 2.5 Hz, 1H), 3.42 (d, *J* = 7.0 Hz, 2H), 3.27 (q, *J* = 7.2 Hz, 1H), 2.92 (dd, *J* = 8.3, 3.6 Hz, 2H), 2.51 (dd, *J* = 18.9, 8.6 Hz, 1H), 2.46 – 2.37 (m, 1H), 2.31 (tt, *J* = 10.8, 5.1 Hz, 1H), 2.22 – 2.11 (m, 1H), 2.09 (dd, *J* = 11.2, 5.8 Hz, 2H), 2.05 – 1.92 (m, 4H), 1.61 (ddd, *J* = 12.0, 7.6, 2.2 Hz, 3H), 1.58 – 1.43 (m, 4H), 1.02 (d, *J* = 6.7 Hz, 3H), 0.92 (s, 3H), 0.81 (d, *J* = 6.7 Hz, 3H).

**^13^C NMR** (101 MHz, CDCl_3_) δ 197.9, 164.4, 149.1, 147.8, 137.0, 136.3, 136.0, 132.0, 129.0, 127.6, 127.5, 127.0, 126.5, 125.4, 120.7, 117.9, 49.4, 46.9, 46.9, 43.1, 41.1, 37.0, 34.9, 32.2, 30.5, 28.4, 25.3, 24.7, 20.6, 19.8, 19.4, 12.8, 12.8.

**LC-MS** (ESI) *m/z* Calcd for C_37_H_41_O_4_ [M+H^+^]: 549.3; found: 549.3.

**(*R*)-4-methyl-1-phenyl-3-(4-(4-(2,3,4-trimethoxybenzyl)piperazine-1-carbonyl)phenyl)pentan-1-one (5d):**

The product was isolated by flash chromatography (PE/EA = 1/1) as white solid. Ligand = **L10**, 106 mg, 97% yield. 95% ee.

**HPLC**: Daicel Chiralcel AD-H, *n*-hexane/isopropanol 70/30, flow rate = 0.8 mL/min, l = 254 nm, *t*_R_ = 16.8 min (major), 25.4 min (minor).

**^1^H NMR** (400 MHz, CDCl_3_) δ 7.91 – 7.79 (m, 2H), 7.55 – 7.48 (m, 1H), 7.44 – 7.39 (m, 2H), 7.28 (dd, *J* = 6.8, 1.5 Hz, 2H), 7.21 (d, *J* = 8.3 Hz, 2H), 6.97 (d, *J* = 8.6 Hz, 1H), 6.64 (d, *J* = 8.6 Hz, 1H), 3.88 (s, 3H), 3.87 (s, 3H), 3.85 (s, 3H), 3.82 – 3.60 (m, 2H), 3.50 (s, 2H), 3.46 – 3.25 (m, 4H), 3.19 (td, *J* = 8.1, 5.4 Hz, 1H), 2.45 (d, *J* = 56.6 Hz, 4H), 1.97 – 1.90 (m, 1H), 0.98 (d, *J* = 6.7 Hz, 3H), 0.78 (d, *J* = 6.7 Hz, 3H).

**^13^C NMR** (101 MHz, CDCl_3_) δ 199.2, 170.4, 153.1, 152.6, 145.6, 142.3, 137.2, 133.5, 133.0, 128.5, 128.4, 128.0, 127.1, 127.0, 125.2, 107.0, 61.3, 60.8, 56.4, 56.0, 47.7, 42.2, 33.2, 20.9, 20.4.

**LC-MS** (ESI) *m/z* Calcd for C_33_H_41_N_2_O_5_ [M+H^+^]: 545.3; found: 545.3.

**(*R*)-4-methyl-1-phenyl-3-(4-(4-(4-phenylquinolin-2-yl)piperazine-1-carbonyl)phenyl)pentan-1-one (5e):**

The product was isolated by flash chromatography (PE/EA = 4/1) as white solid. Ligand = **L10**, 98 mg, 86% yield. 96% ee.

**HPLC**: Daicel Chiralcel AD-H, *n*-hexane/isopropanol 70/30, flow rate = 0.8 mL/min, l = 254 nm, *t*_R_ = 15.7 min (major), 25.3 min (minor).

**^1^H NMR** (400 MHz, CDCl_3_) δ 7.89 (d, *J* = 7.3 Hz, 2H), 7.77 (d, *J* = 8.3 Hz, 1H), 7.65 (dd, *J* = 8.3, 1.3 Hz, 1H), 7.58 – 7.41 (m, 9H), 7.34 (d, *J* = 7.8 Hz, 2H), 7.28 – 7.24 (m, 2H), 7.23 – 7.17 (m, 1H), 6.90 (s, 1H), 3.84 (d, *J* = 43.1 Hz, 6H), 3.56 (s, 2H), 3.46 – 3.29 (m, 2H), 3.22 (td, *J* = 8.1, 5.3 Hz, 1H), 1.96 (h, *J* = 6.9 Hz, 1H), 1.00 (d, *J* = 6.7 Hz, 3H), 0.81 (d, *J* = 6.7 Hz, 3H).

**^13^C NMR** (101 MHz, CDCl_3_) δ 199.1, 170.7, 156.7, 150.2, 148.2, 146.0, 138.7, 137.2, 133.3, 133.0, 129.7, 129.4, 128.6, 128.5, 128.5, 128.3, 128.0, 127.2, 127.1, 125.7, 122.9, 122.3, 109.8, 109.8, 47.7, 45.5, 42.2, 33.2, 20.9, 20.5.

**LC-MS** (ESI) *m/z* Calcd for C_38_H_38_N_3_O_2_ [M+H^+^]: 568.3; found: 568.3.

**(*R*)-3-(3-(4-(cyclopropanecarbonyl)piperazine-1-carbonyl)-4-fluorophenyl)-4-methyl-1-phenylpentan-1-one (5f):**

The product was isolated by flash chromatography (PE/EA = 1/1) as white solid. Ligand = **L10**, 80 mg, 89% yield. 90% ee.

**HPLC**: Daicel Chiralcel AD-H, *n*-hexane/isopropanol 80/20, flow rate = 1.0 mL/min, l = 254 nm, *t*_R_ = 12.3 min (major), 18.9 min (minor).

**^1^H NMR** (400 MHz, CDCl_3_) δ 7.85 (d, *J* = 7.6 Hz, 2H), 7.56 – 7.51 (m, 1H), 7.43 (t, *J* = 7.6 Hz, 2H), 7.26 – 7.19 (m, 1H), 7.19 – 7.13 (m, 1H), 7.00 (t, *J* = 8.9 Hz, 1H), 3.87 – 3.49 (m, 7H), 3.38 – 3.12 (m, 5H), 1.95 – 1.89 (m, 1H), 1.01 (td, *J* = 6.7, 4.2 Hz, 5H), 0.80 (d, *J* = 6.7 Hz, 5H).

**^13^C NMR** (101 MHz, CDCl_3_) δ 199.0, 172.3, 165.6 (d, *J* _C-F_= 16.7 Hz), 156.7 (d, *J* _C-F_= 246.0 Hz), 141.6, 140.3, 137.1, 133.1, 128.6, 128.0, 122.0 (d, *J* _C-F_= 182.4 Hz), 115.5 (d, *J* _C-F_= 18.6 Hz), 47.3, 42.4, 42.3, 33.1, 20.8, 20.5, 11.1, 7.7.

**LC-MS** (ESI) *m/z* Calcd for C_27_H_32_FN_2_O_3_ [M+H^+^]: 451.2; found: 451.2.

***tert*-butyl (*R*)-4-(1-(4-((3-chloro-4-fluorophenyl)amino)quinazolin-6-yl)-3-oxo-3-phenylpropyl)piperidine-1-carboxylate (5g):**

The product was isolated by flash chromatography (PE/EA = 2/1) as white solid. Ligand = **L10**, 61 mg, 52% yield. 90% ee.

**HPLC**: Daicel Chiralcel AD-H, *n*-hexane/isopropanol 70/30, flow rate = 0.8 mL/min, l = 254 nm, *t*_R_ = 21.3 min (major), 32.7 min (minor).

**^1^H NMR** (400 MHz, CDCl_3_) δ 8.68 (s, 1H), 7.90 – 7.83 (m, 4H), 7.81 (d, *J* = 8.6 Hz, 1H), 7.61 (dd, *J* = 8.7, 1.8 Hz, 1H), 7.58 – 7.51 (m, 2H), 7.41 (t, *J* = 7.8 Hz, 2H), 7.12 (t, *J* = 8.8 Hz, 1H), 4.08 (d, *J* = 58.8 Hz, 2H), 3.48 (d, *J* = 6.8 Hz, 2H), 3.35 (q, *J* = 7.3 Hz, 1H), 2.67 (s, 1H), 2.54 (s, 1H), 2.28 (s, 3H), 1.80 (tdd, *J* = 15.4, 12.8, 11.7, 5.6 Hz, 2H), 1.42 (s, 9H).

**^13^C NMR** (101 MHz, CDCl_3_) δ 199.0, 157.4, 156.0, 154.8, 154.3, 153.6, 148.7, 141.8, 136.7, 135.1, 133.5, 132.3, 128.9, 128.8, 128.0, 124.1, 121.8, 121.8, 121.1, 121.0, 120.8, 116.6, 116.4, 114.9, 79.7, 46.7, 41.9, 41.2, 30.4, 28.4.

**LC-MS** (ESI) *m/z* Calcd for C_33_H_35_ClFN_4_O_3_ [M+H^+^]: 589.2; found: 589.2.

**(*R*)-*N*-(cyclohexylcarbamoyl)-4-(4-methyl-1-oxo-1-phenylpentan-3-yl)benzenesulfonamide (3m-urea):**

The product is filtered to obtain a white solid. 0.5 mmol, 223.5 mg, 98% yield. 98% ee.

**HPLC**: Daicel Chiralcel AD-H, *n*-hexane/isopropanol 70/30, flow rate = 0.8 mL/min, l = 254 nm, *t*_R_ = 11.9 min (major), 16.1 min (minor).

**^1^H NMR** (400 MHz, CDCl_3_) δ 7.87 (d, *J* = 7.4 Hz, 2H), 7.80 (d, *J* = 8.3 Hz, 2H), 7.54 (t, *J* = 7.3 Hz, 1H), 7.43 (t, *J* = 7.6 Hz, 2H), 7.36 (d, *J* = 8.2 Hz, 2H), 6.37 (d, *J* = 8.0 Hz, 1H), 3.57 (dtd, *J* = 10.3, 6.8, 4.0 Hz, 1H), 3.39 (dd, *J* = 6.8, 3.7 Hz, 2H), 3.27 (q, *J* = 7.1 Hz, 1H), 2.03 – 1.95 (m, 1H), 1.38 – 1.11 (m, 10H), 0.99 (d, *J* = 6.7 Hz, 3H), 0.78 (d, *J* = 6.7 Hz, 3H).

**^13^C NMR** (101 MHz, CDCl_3_) δ 198.5, 150.7, 150.6, 137.3, 136.9, 133.2, 129.2, 128.6, 128.0, 127.0, 49.3, 47.5, 42.0, 33.9, 33.1, 25.6, 24.9, 20.8, 20.4.

**LC-MS** (ESI) *m/z* Calcd for C_25_H_33_N_2_O_4_S [M+H^+^]: 457.2; found: 457.2.

***tert*-butyl (*R*)-4-(1-(3-(4-methoxybenzyl)-4-oxo-3,4-dihydroquinazolin-6-yl)-3-oxo-3-phenylpropyl)piperidine-1-carboxylate (4w):**

The product was isolated by flash chromatography (PE/EA = 2/1) as white solid. Ligand = **L10**, **0.2 mmol scale**: 98.5 mg, 85% yield. 90% ee. Ligand = **L10**, **3 mmol scale**: 1.43 g, 82%, 90% ee.

**HPLC**: Daicel Chiralcel AD-H, *n*-hexane/isopropanol 70/30, flow rate = 0.8 mL/min, l = 254 nm, *t*_R_ = 37.5 min (major), 43.8 min (minor).

**^1^H NMR** (400 MHz, CDCl_3_) δ 8.10 (t, *J* = 1.4 Hz, 1H), 8.06 (s, 1H), 7.91 – 7.85 (m, 2H), 7.62 (d, *J* = 1.4 Hz, 2H), 7.56 – 7.50 (m, 1H), 7.42 (dd, *J* = 8.4, 7.1 Hz, 2H), 7.33 – 7.28 (m, 2H), 6.90 – 6.84 (m, 2H), 5.11 (s, 2H), 4.14 (s, 1H), 4.02 (s, 1H), 3.78 (s, 3H), 3.50 – 3.40 (m, 2H), 2.92 (d, *J* = 29.1 Hz, 1H), 2.66 (s, 1H), 2.55 (s, 1H), 1.79 (d, *J* = 11.4 Hz, 4H), 1.42 (s, 9H).

**^13^C NMR** (101 MHz, CDCl_3_) δ 198.3, 161.1, 159.6, 154.7, 146.7, 145.9, 143.0, 136.8, 135.7, 133.2, 129.6, 128.6, 128.0, 127.8, 127.4, 125.0, 122.1, 114.4, 79.4, 55.3, 49.3, 46.0, 42.0, 41.6, 28.4.

**LC-MS** (ESI) *m/z* Calcd for C_35_H_40_N_3_O_5_ [M+H^+^]: 582.3; found: 582.3.

**(*S*)-3-(4-butylphenyl)-1,3-diphenylpropan-1-one (4x):**

The product was isolated by flash chromatography (PE/EA = 20/1) as white solid. Ligand = (*R*)-**L1**, **3 mmol scale**: 0.9 g, 87%, 90% ee.

**HPLC**: Daicel Chiralcel IG-3, *n*-hexane/isopropanol 95/5, flow rate = 1.0 mL/min, l = 254 nm, *t*_R_ = 10.1 min (minor), 10.8 min (major).

**^1^H NMR** (600 MHz, CDCl_3_) δ 7.91 (d, *J* = 7.0 Hz, 2H), 7.53 (t, *J* = 7.4 Hz, 1H), 7.43 (t, *J* = 7.7 Hz, 2H), 7.26-7.25 (m, 4H), 7.16 (d, *J* = 8.2 Hz, 3H), 7.06 (d, *J* = 7.9 Hz, 2H), 4.78 (t, *J* = 7.3 Hz, 1H), 3.72-3.71 (m, 2H), 2.53 (t, *J* = 7.9 Hz, 2H), 1.57-1.48 (m, 2H), 1.37-1.30 (m, 2H), 0.89 (t, *J* = 7.3 Hz, 3H).

**^13^C NMR** (151 MHz, CDCl_3_) 198.1, 144.4, 141.3, 140.9, 137.1, 133.0, 128.6, 128.5, 128.0, 127.8, 127.6, 126.3, 45.6, 44.8, 35.2, 33.5, 22.4, 13.9.

**LC-MS** (ESI) *m/z* Calcd for C_25_H_26_NaO [M+Na^+^]: 365.2; found: 365.2.

Spectroscopic data are in accordance with that reported in the literature.^8^

**8.** **X-Ray Crystallographic Data of 3m**


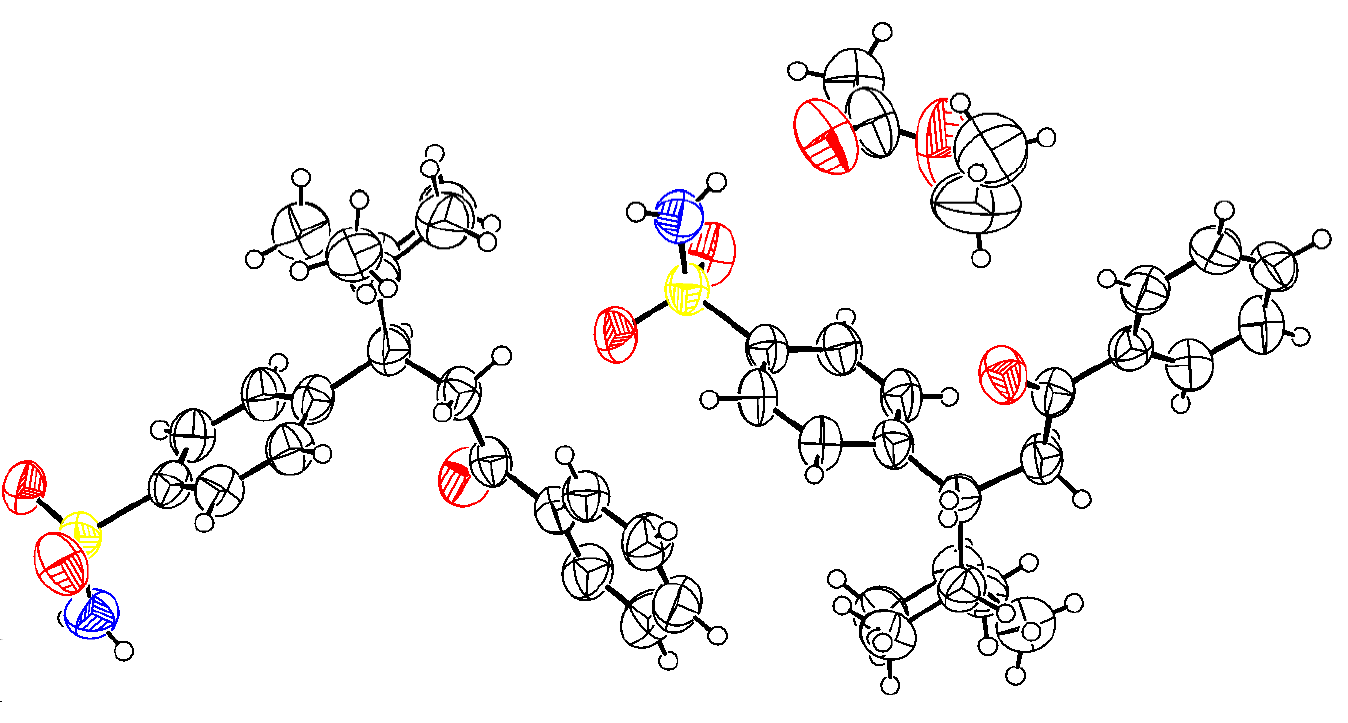


CH_3_COOCH_2_CH_3_

**Table S5**. Crystal data and structure refinement for **3m** **(CCDC 2294364)**

| Empirical Formula | C_40_H_49_N_2_O_8_S_2_ |
| --- | --- |
| Formula weight | 749.93 |
| Temperature/K | 303.51(10) |
| Crystal system | triclinic |
| Space group | P1 |
| *a*/Å | 5.40970(10) |
| *b*/Å | 12.6455(2) |
| *c*/Å | 14.7997(2) |
| *α*/° | 87.2670(10) |
| *β*/° | 81.6170(10) |
| *γ*/° | 80.0840(10) |
| *V*/Å^3^ | 986.38(3) |
| *Z* | 1 |
| *D*_calcd_ /g⋅cm^-3^ | 1.262 |
| *μ*/mm^-1^ | 1.657 |
| F(000) | 399.0 |
| Crystal size/mm^3^ | 0.09 × 0.07 × 0.05 |
| Radiation | Cu Kα (λ = 1.54184) |
| *θ* range/° | 9.216 to 151.714 |
| Index ranges | -6 ≤ h ≤ 6, -15 ≤ k ≤ 15, -18 ≤ l ≤ 18 |
| Reflections collected | 27570 |
| Independent reflections | 7402 [R_int_ = 0.0259, R_sigma_ = 0.0243] |
| Data/restraints/parameters | 7402/114/533 |
| Goodness-of-fit on F^2^ | 1.071 |
| Final R indexes [I>=2σ (I)] | R_1_ = 0.0461, wR_2_ = 0.1339 |
| Final R indexes [all data] | R_1_ = 0.0486, wR_2_ = 0.1371 |
| Largest diff. peak/hole / e Å^-3^ | 0.38/-0.34 |
| Flack parameter | 0.014(5) |

**9.** **Reference**

[1] (a) M.-Y. Chang, Y.-C. Chen, C.-K. Chan, *Tetrahedron*, **2014**, *70*, 2257-2263; (b) D.-N. Liu, S.-K. Tian, *Chem. - Eur. J.*, **2009**, *15*, 4538-4542.

[2] S.-Z. Sun, Y. Duan, R. S. Mega, R. J. Somerville, R. Martin, *Angew. Chem. Int. Ed.* **2020**, *59*, 4370 –4374.

[3] P. He, Y. Lu, C.-G. Dong, Q.-S. Hu, *Org. Lett*., **2007**, *9*, 343-346.

[4] G. Casotti, G. Ciancaleoni, F. Lipparini, C. Nieri A. Iuliano, *Chem. Sci.*, **2020**, *11*, 257-263.

[5] M. Shimizu, T. Yamamoto, *Tetrahedron Lett.*, 2020, 61, 152257.

[6] L. Zhang, M. Zhao, M. Pu, Z. Ma, J. Zhou, C. Chen, Y.-D. Wu, Y. R. Chi, J. S. Zhou, *J. Am. Chem. Soc.* **2022**, *144*, 20249–20257.

[7] B. Brooks, N. Hiller, J. A. May., *Tetrahedron Lett.*, **2021**, *83*, 153412*.*

[8] L-L Zhang, Y.-Z. Gao, S.-H. Cai, H. Yu, S.-J. Shen, Q. Ping, Z.-P. Yang, *Nat. Commun.* **2024**, *15*, 2733

**10.** **NMR Spectra**

**1,3-diphenyl-3-(*p*-tolyl)propan-1-one (3a)**


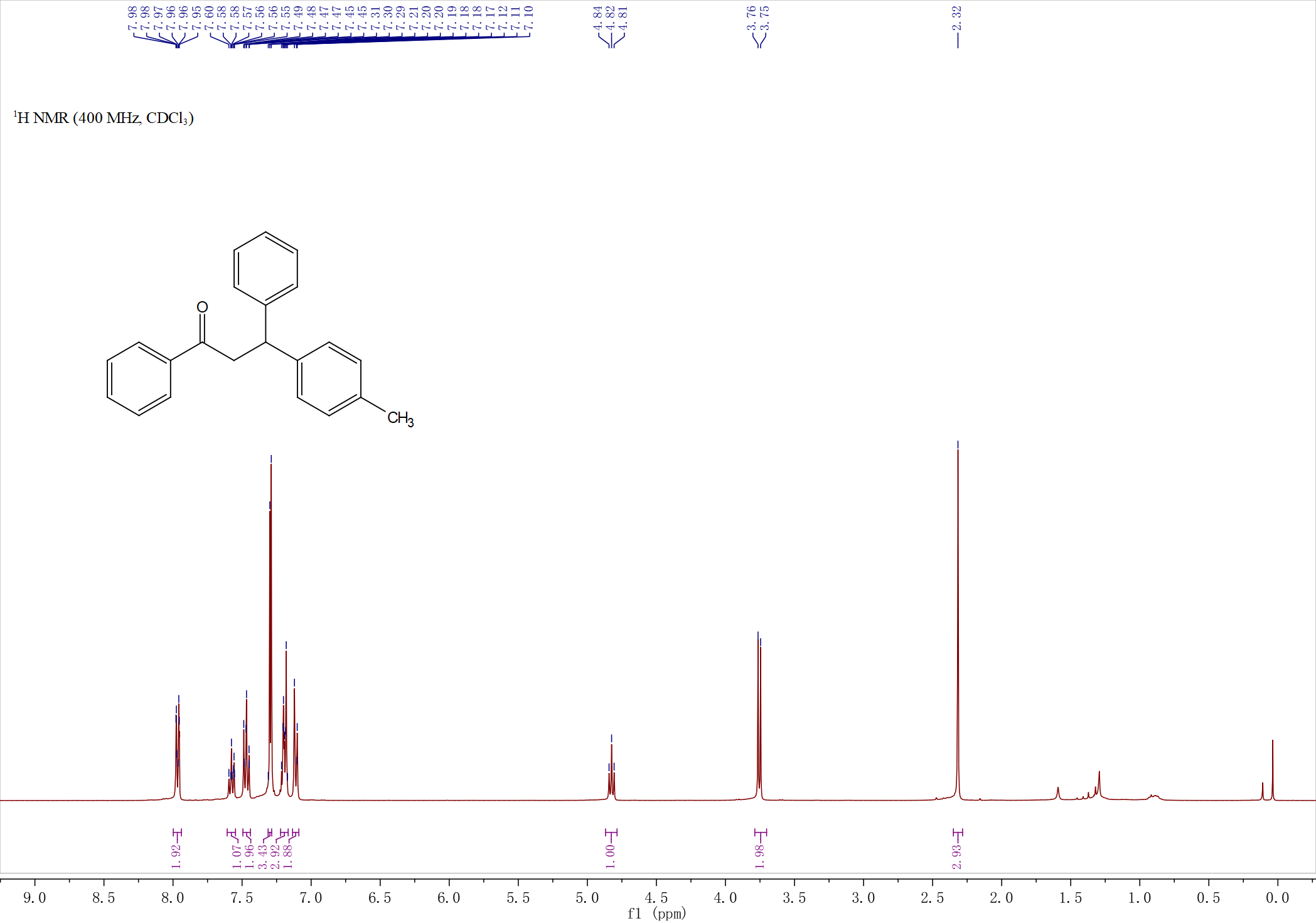


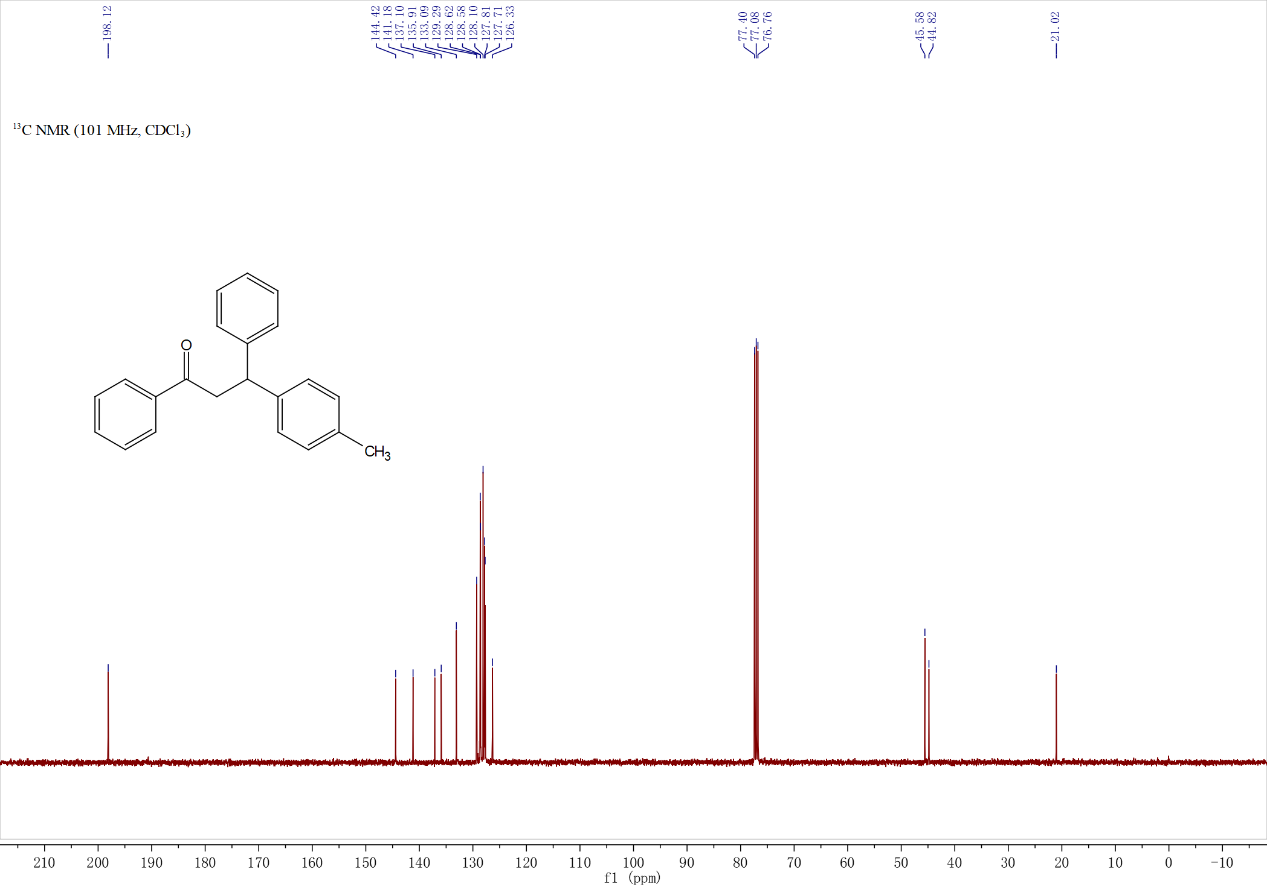


**3-(4-methoxyphenyl)-1,3-diphenylpropan-1-one (3b)**


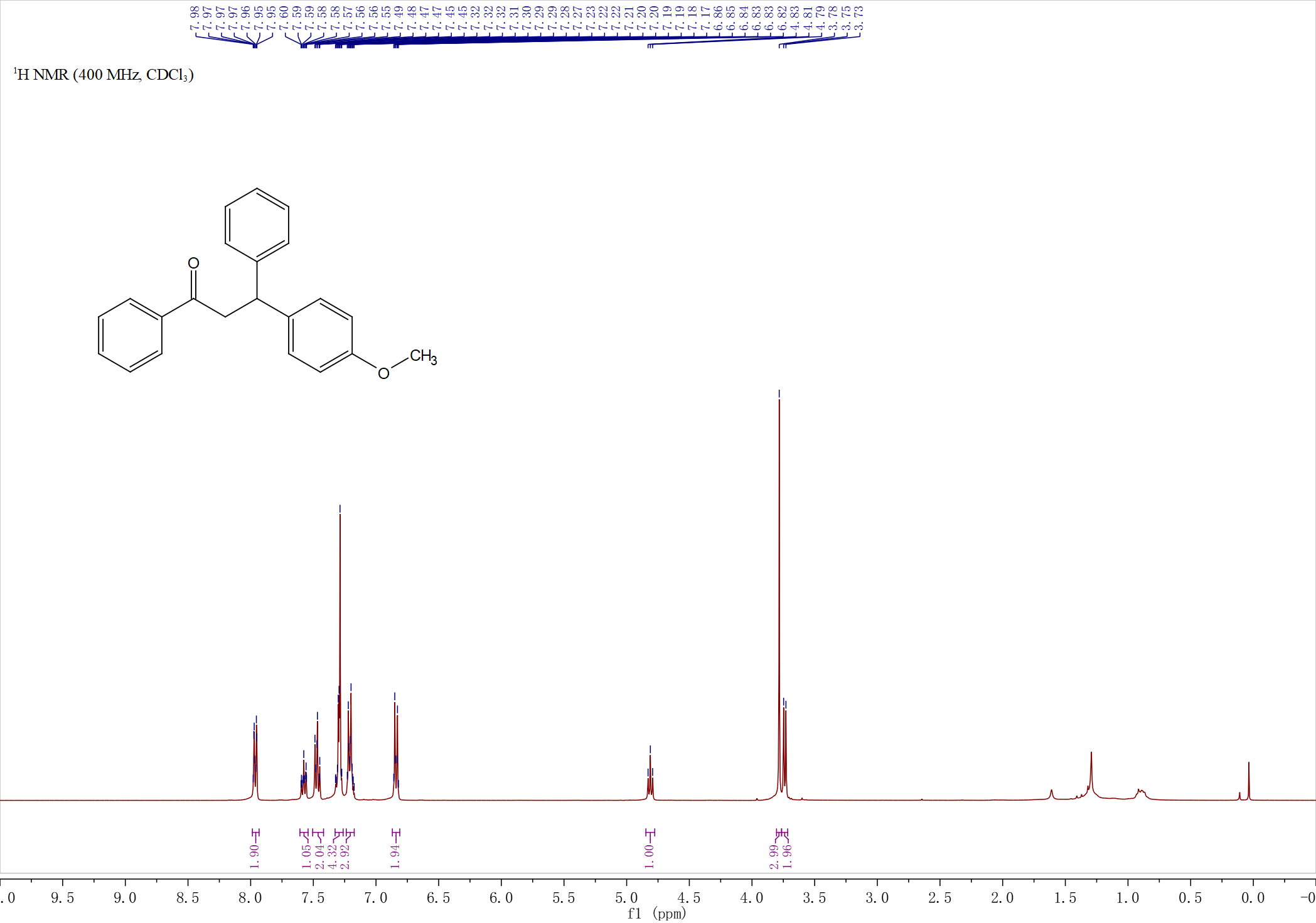


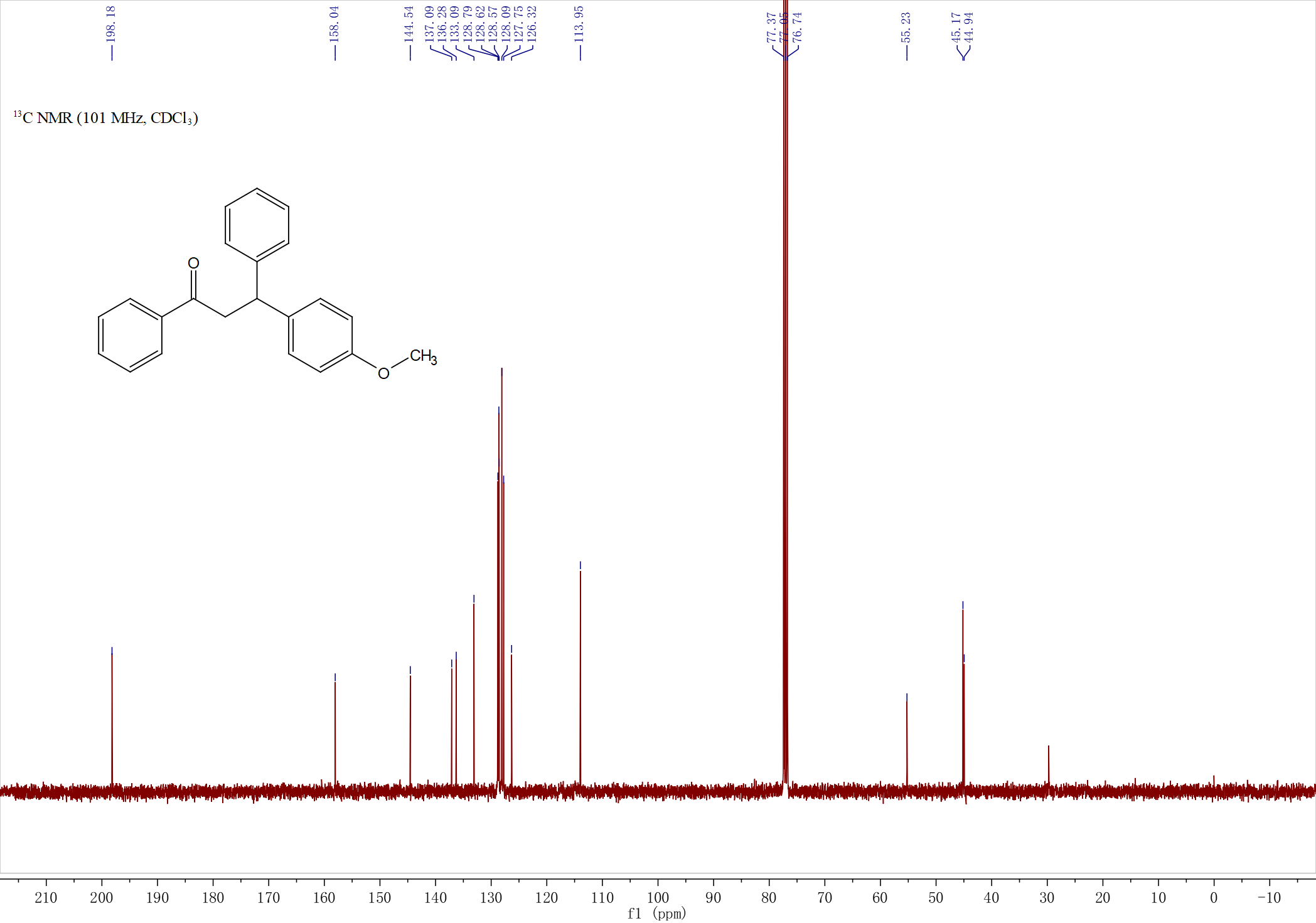


**Methyl 4-(3-oxo-1,3-diphenylpropyl)benzoate (3c)**


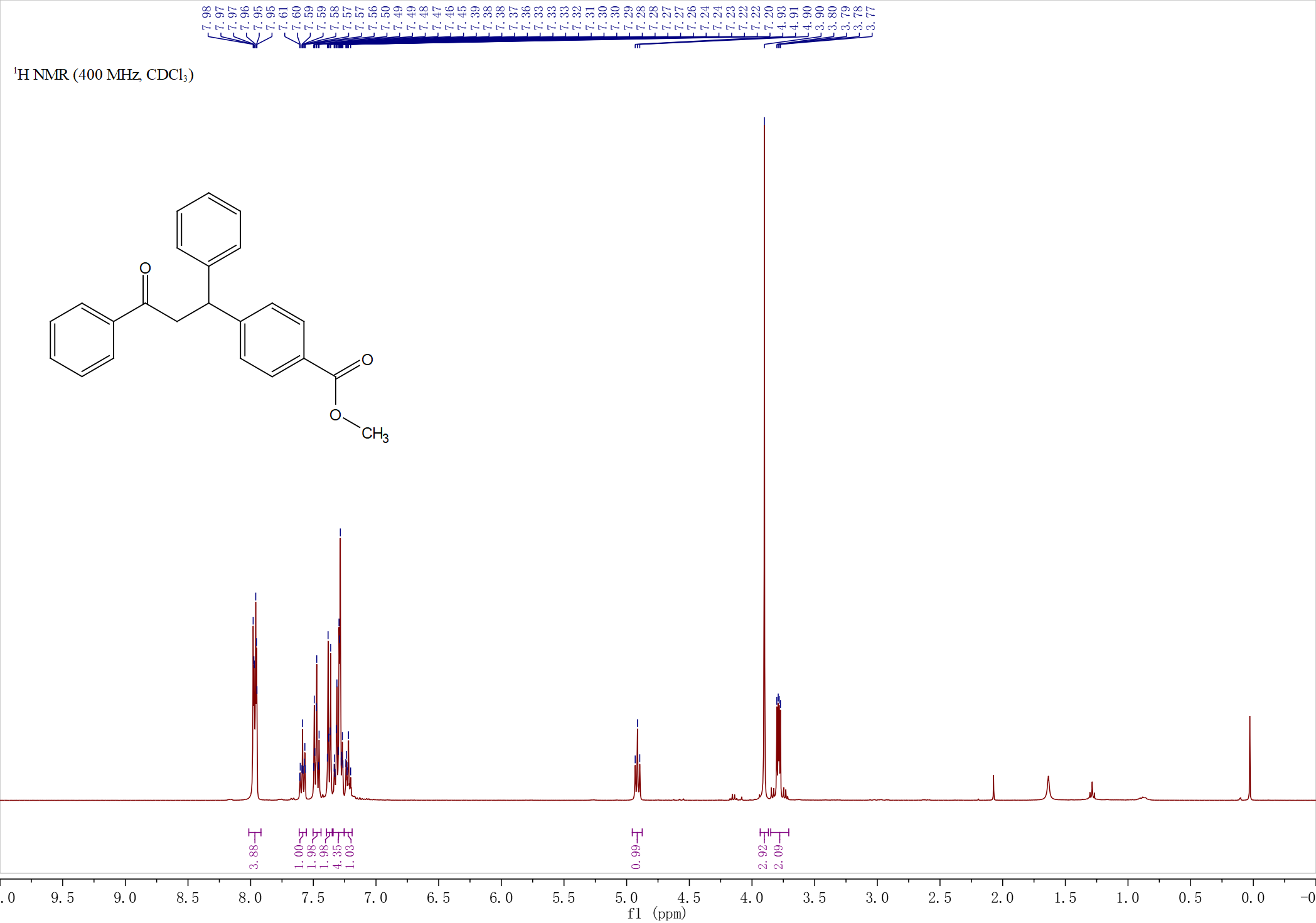


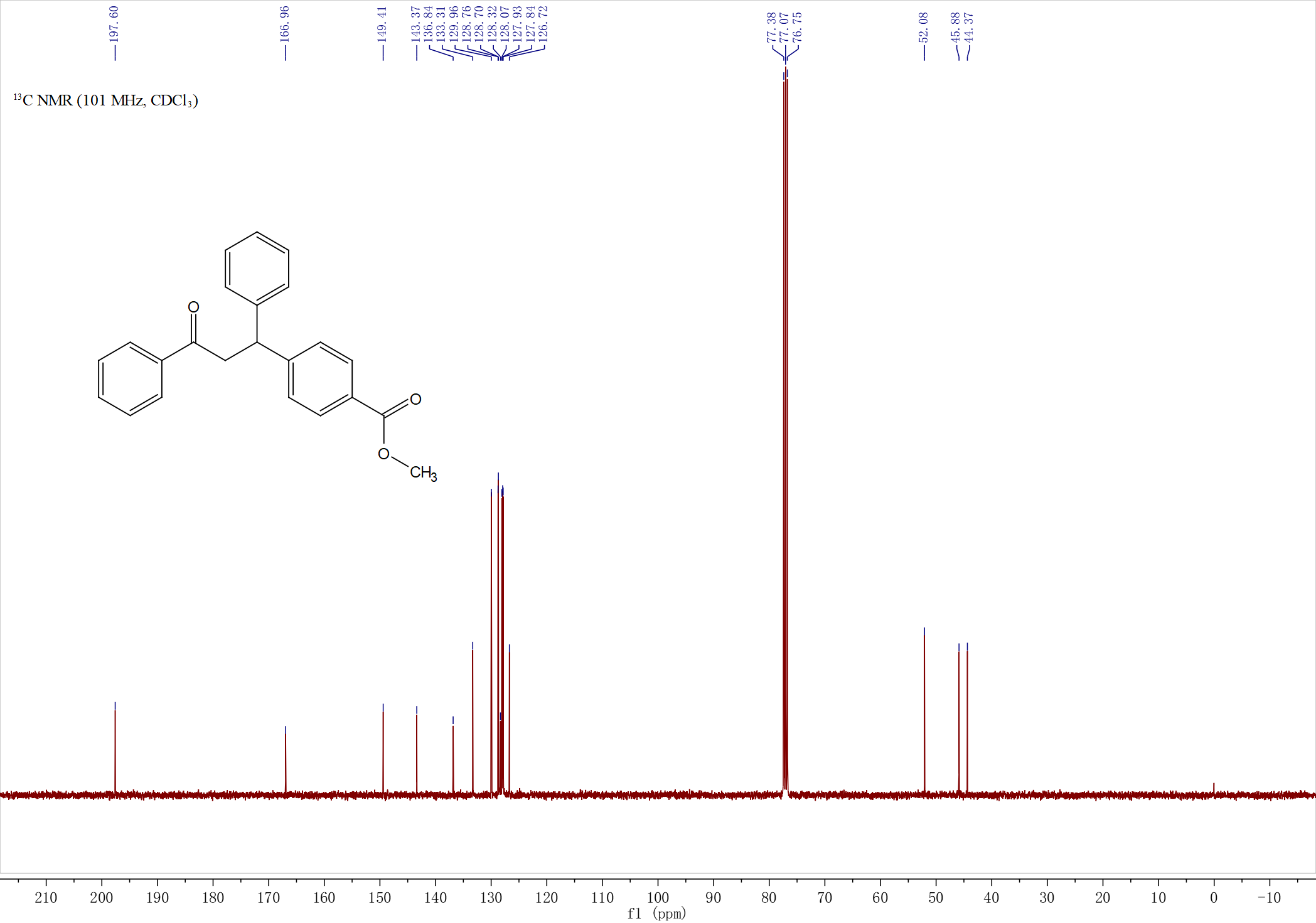


**3-(4-acetylphenyl)-1,3-diphenylpropan-1-one (3d)**


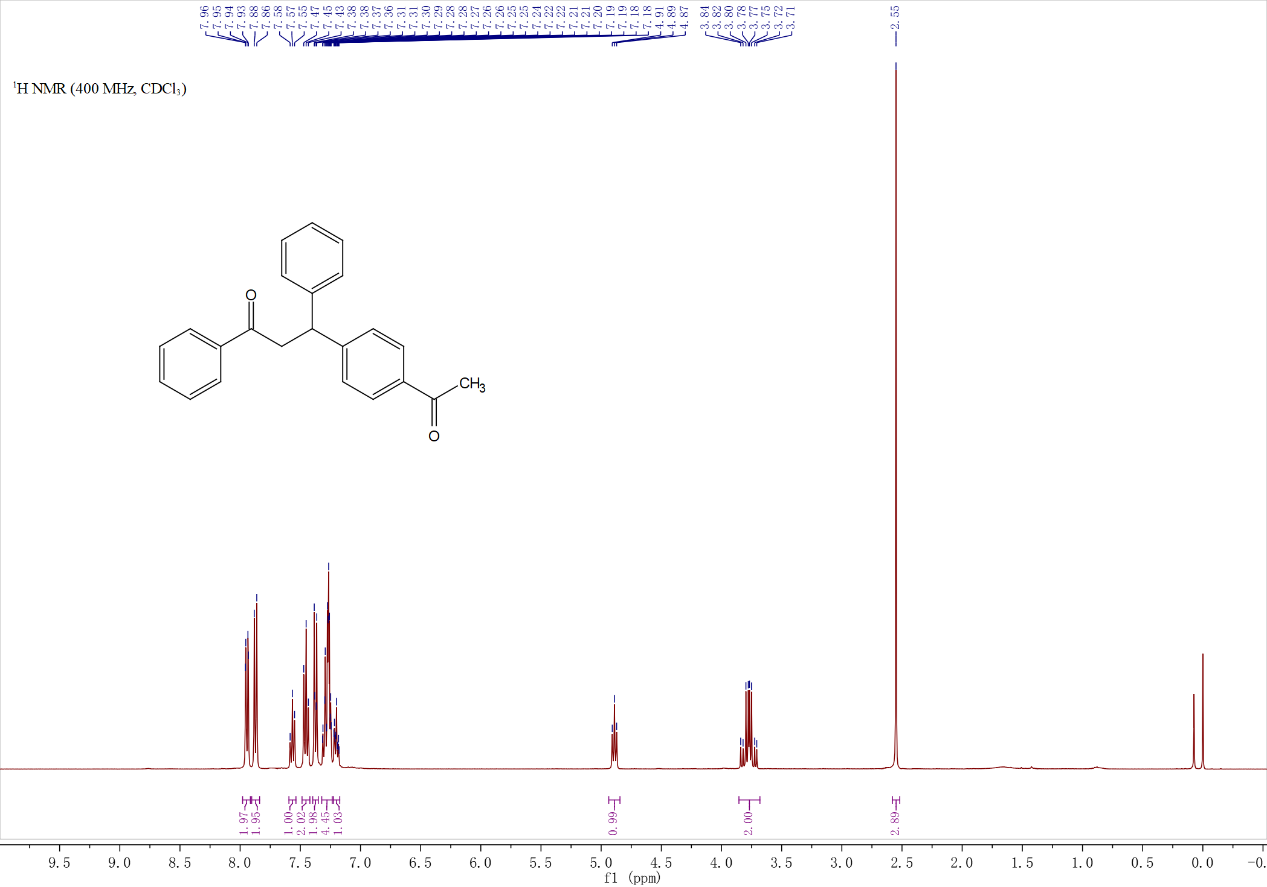


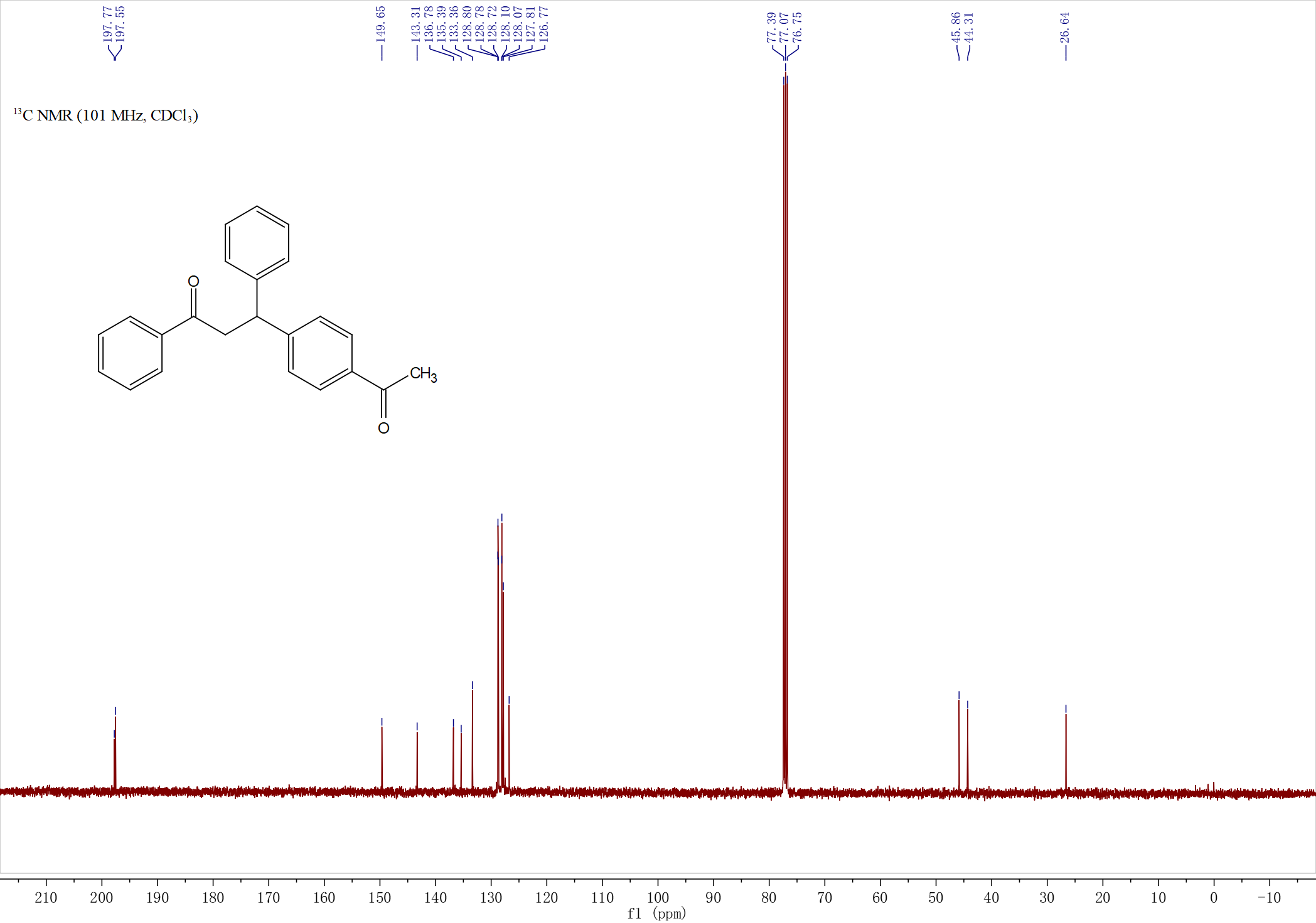


**3-(naphthalen-2-yl)-1,3-diphenylpropan-1-one (3e)**


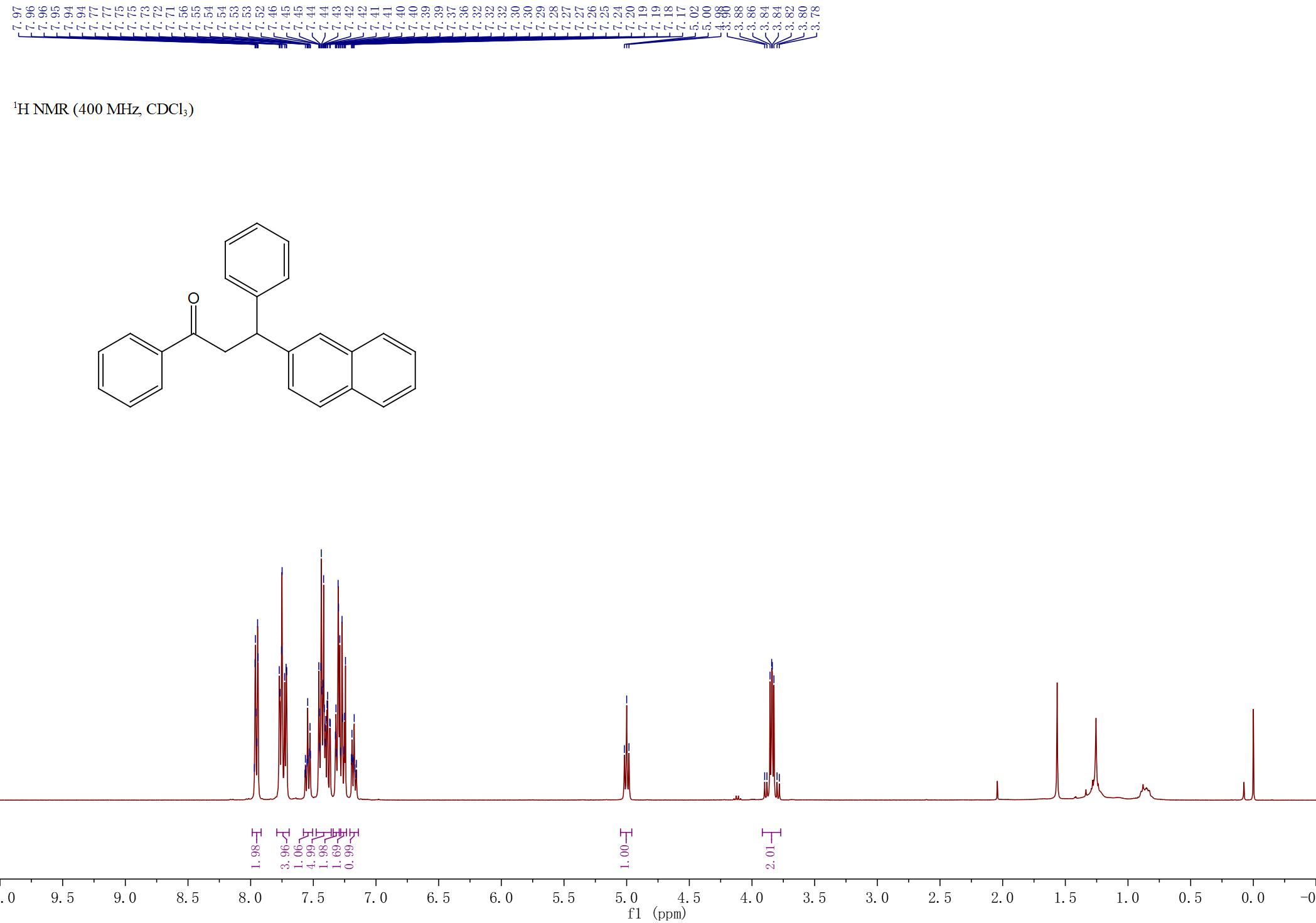


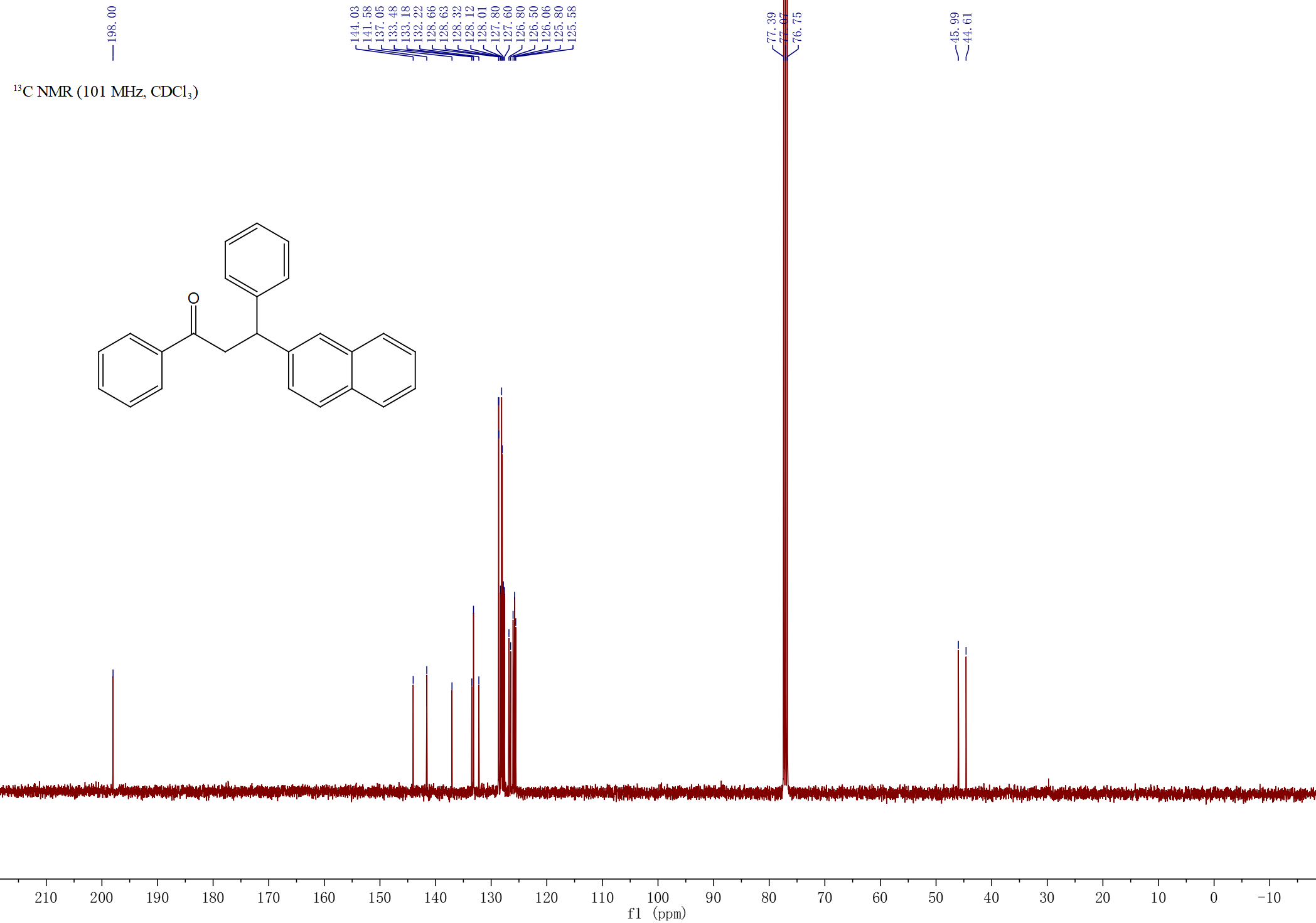


**4-methyl-1-phenyl-3-(*p*-tolyl)pentan-1-one (3f)**


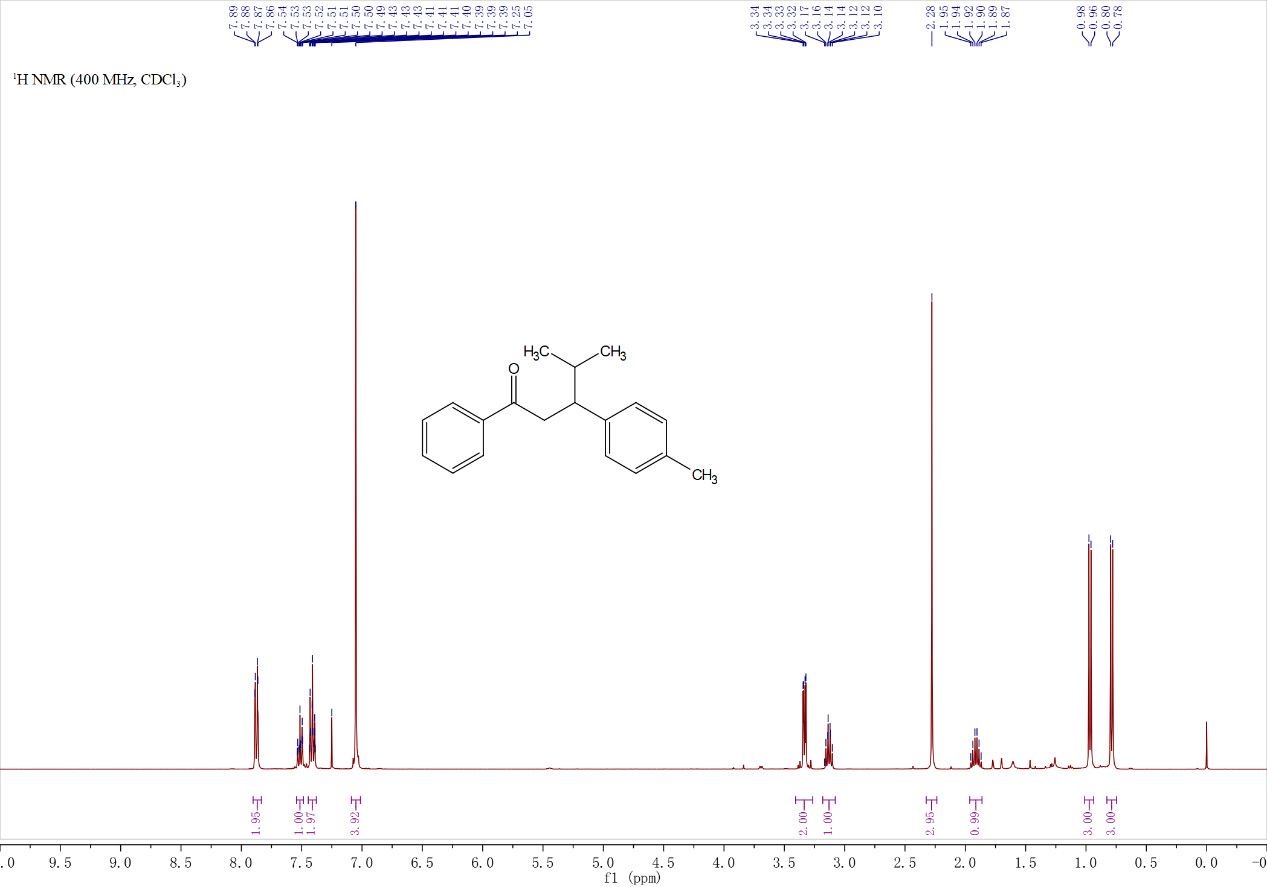


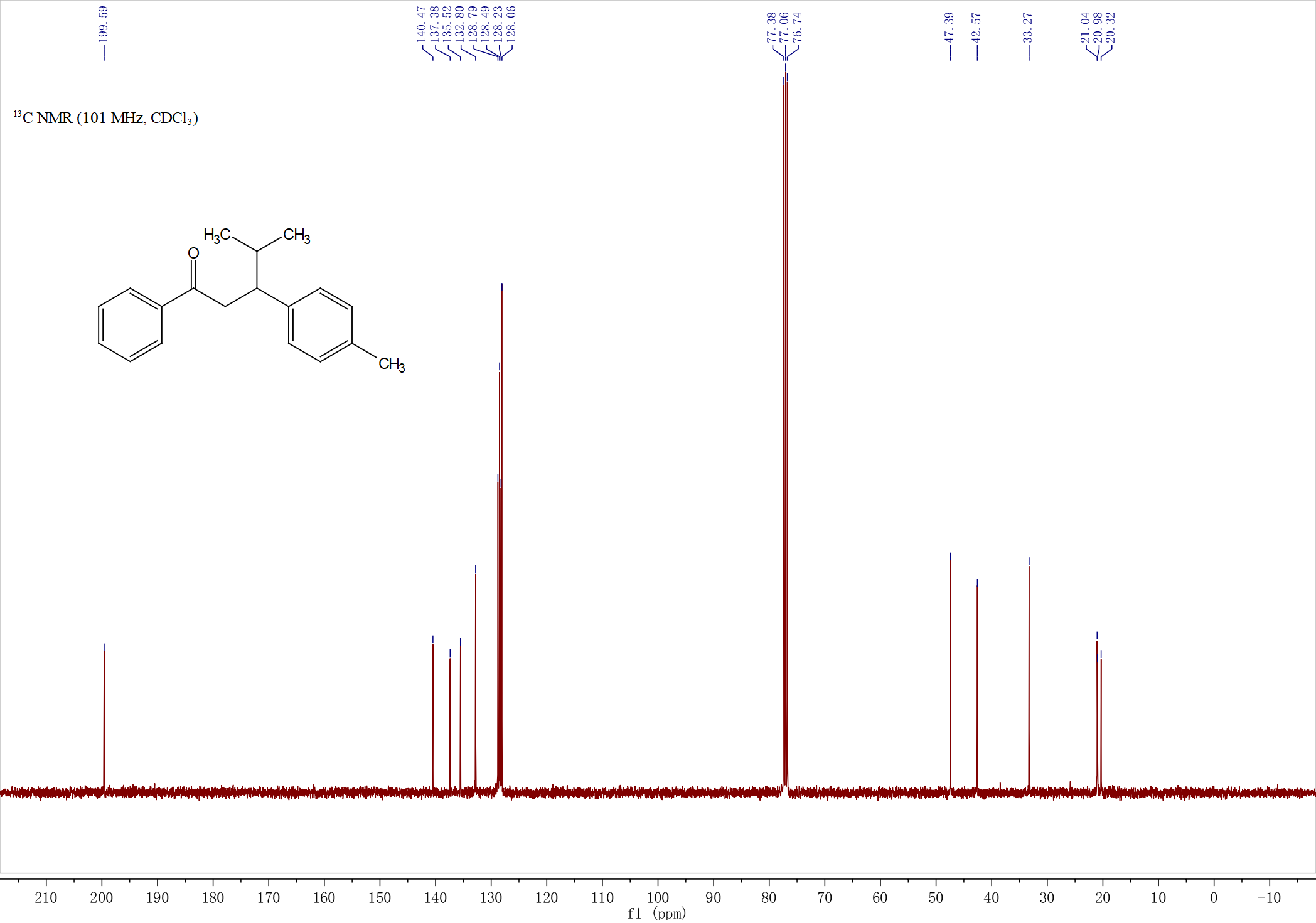


**4-methyl-3-(4-(methylthio)phenyl)-1-phenylpentan-1-one (3g)**


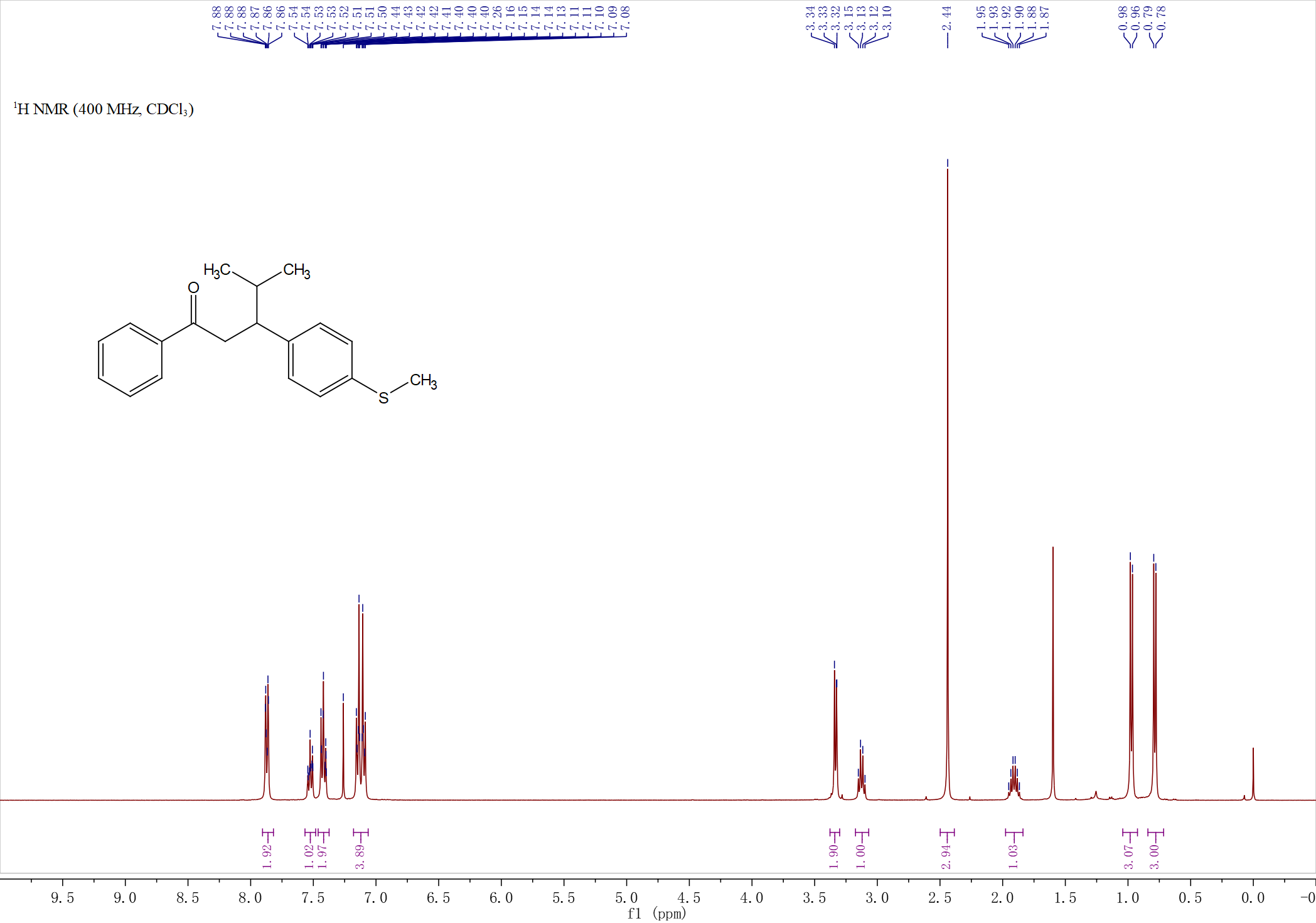


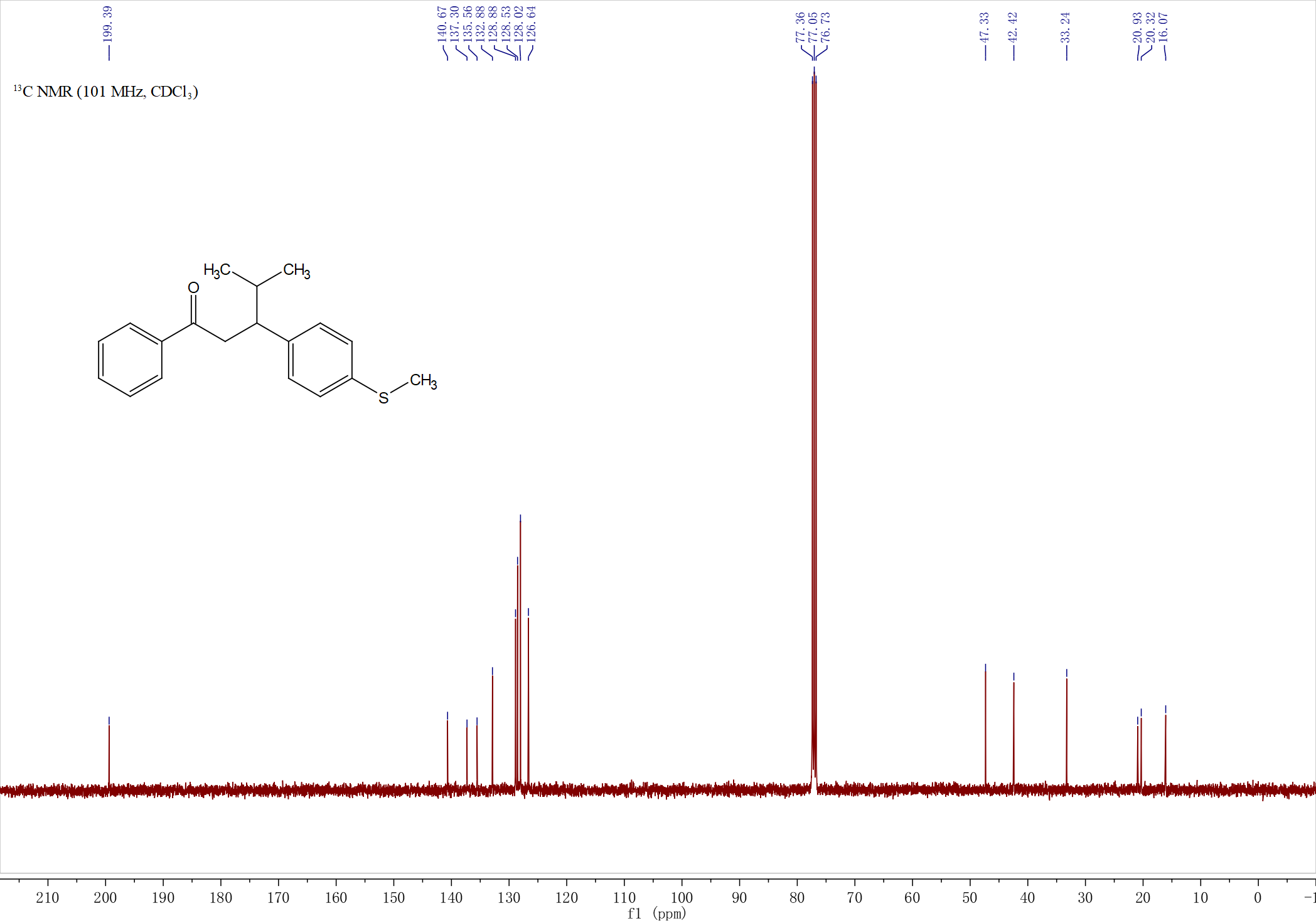


**3-([1,1'-biphenyl]-4-yl)-4-methyl-1-phenylpentan-1-one (3h)**


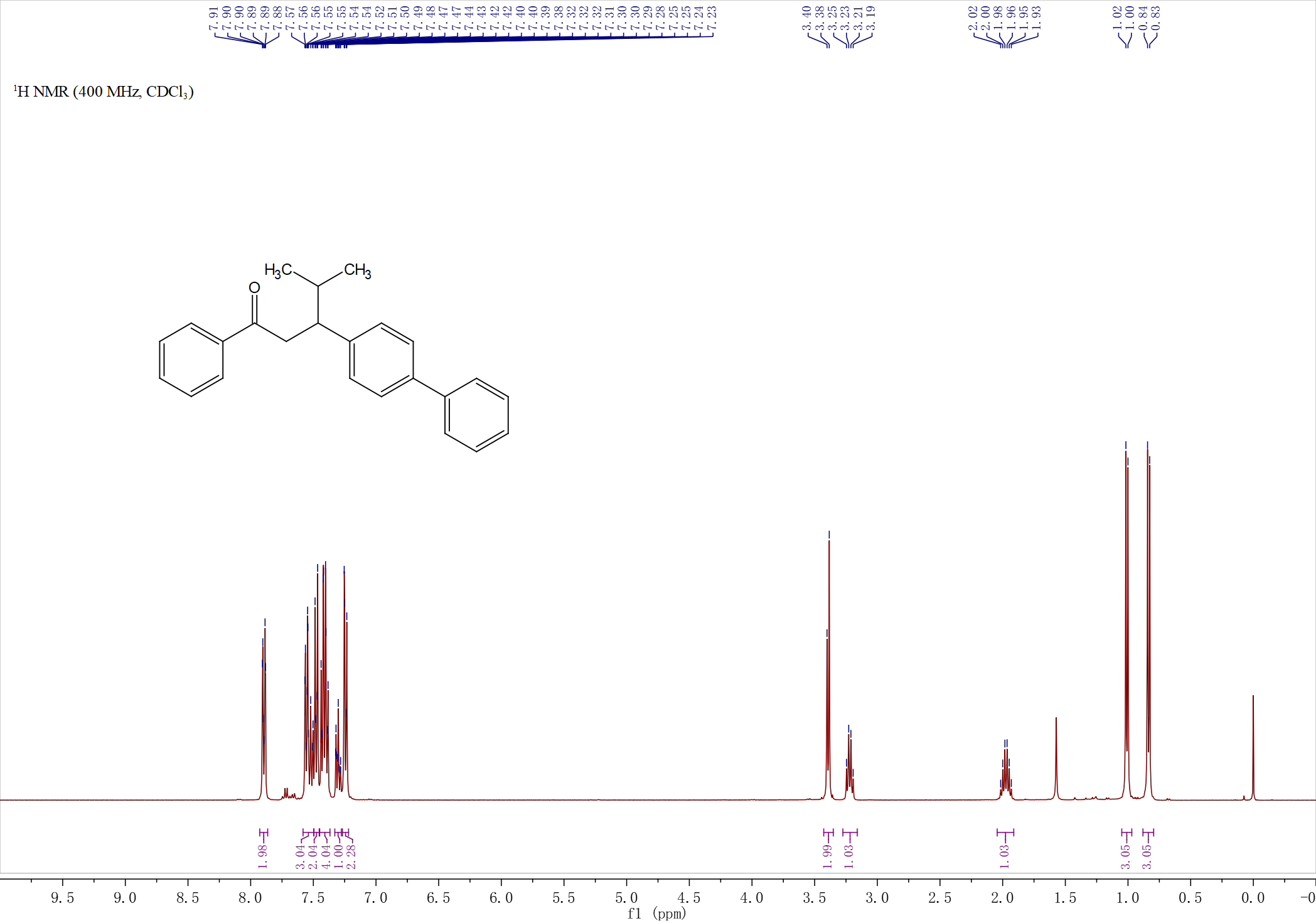


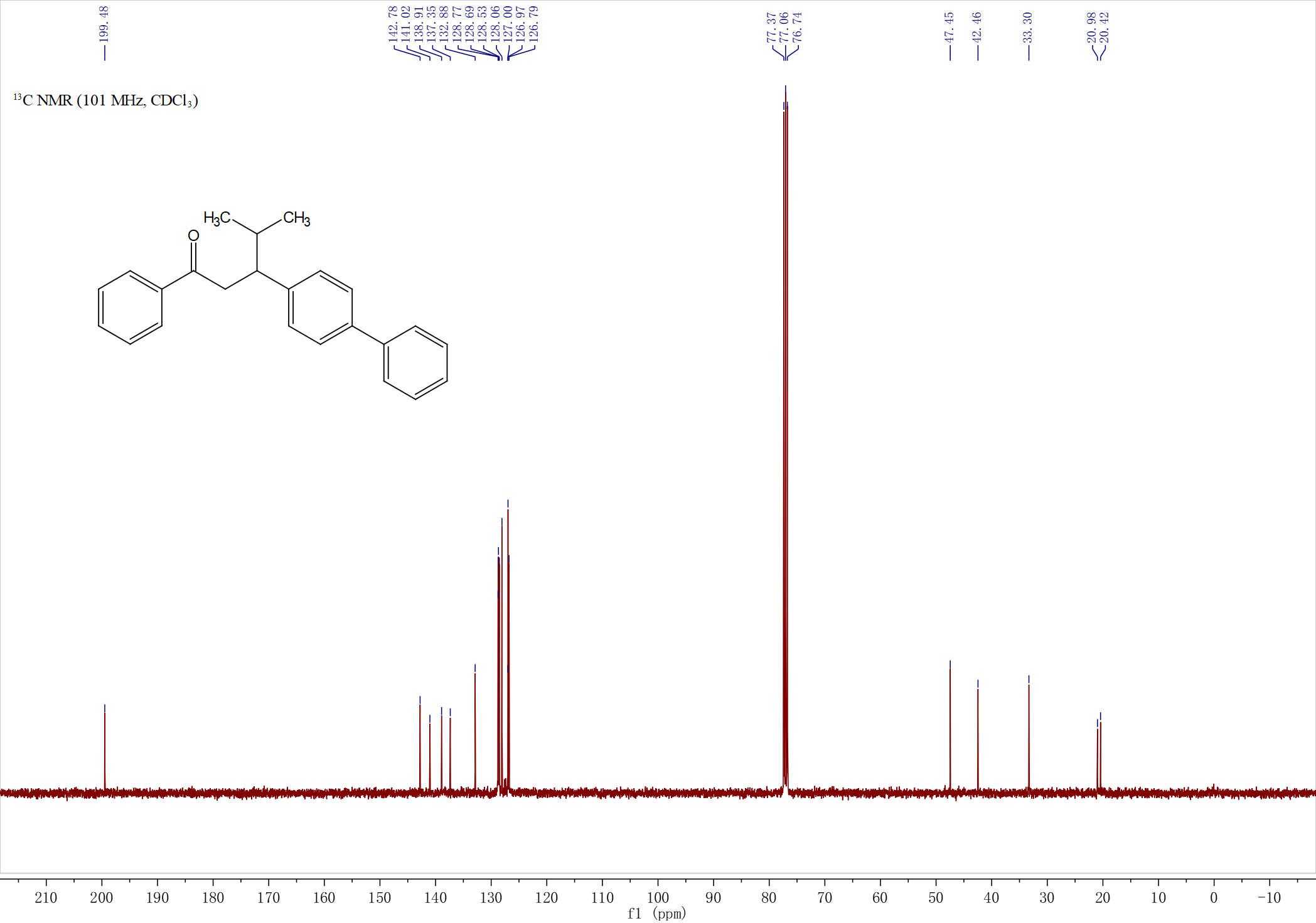


**Methyl 4-(4-methyl-1-oxo-1-phenylpentan-3-yl)benzoate (3i)**


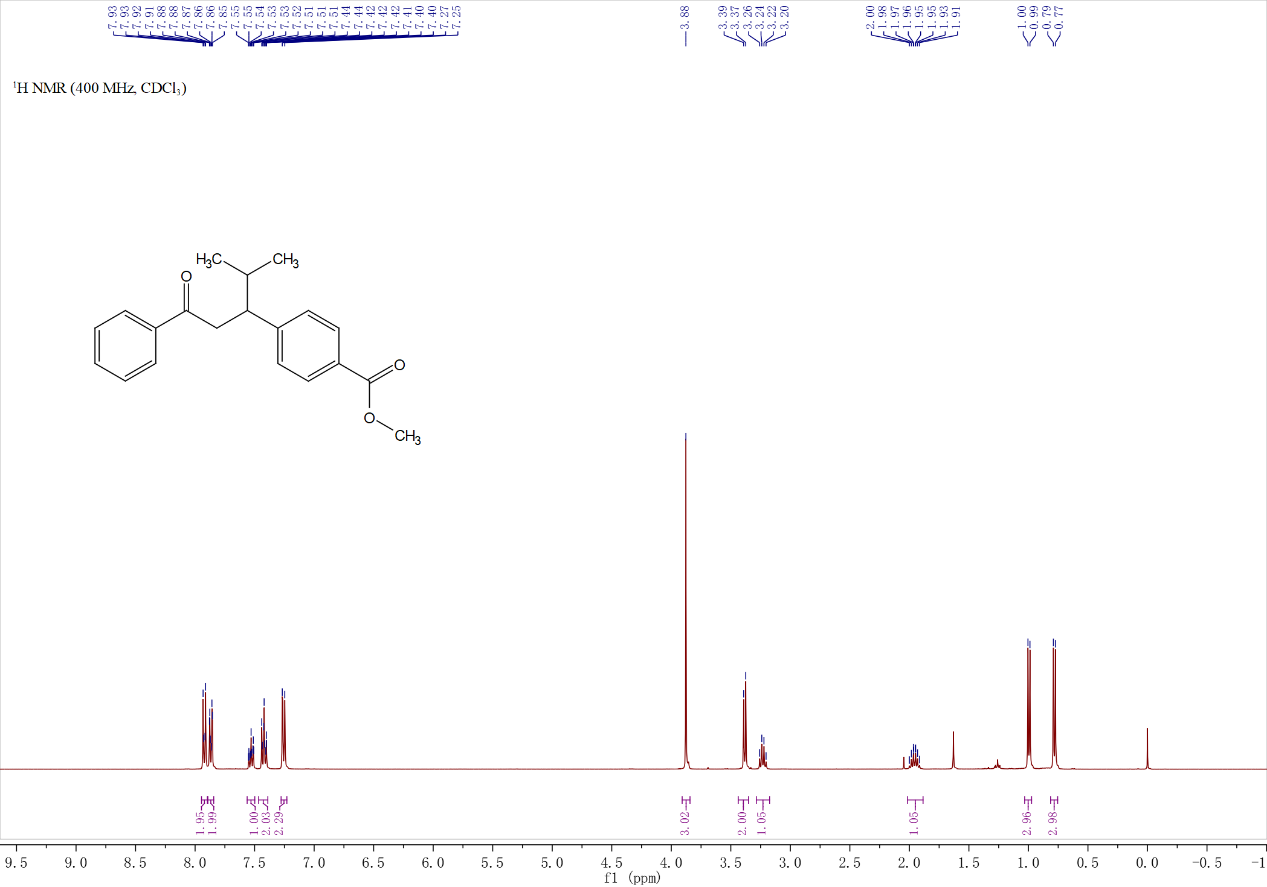


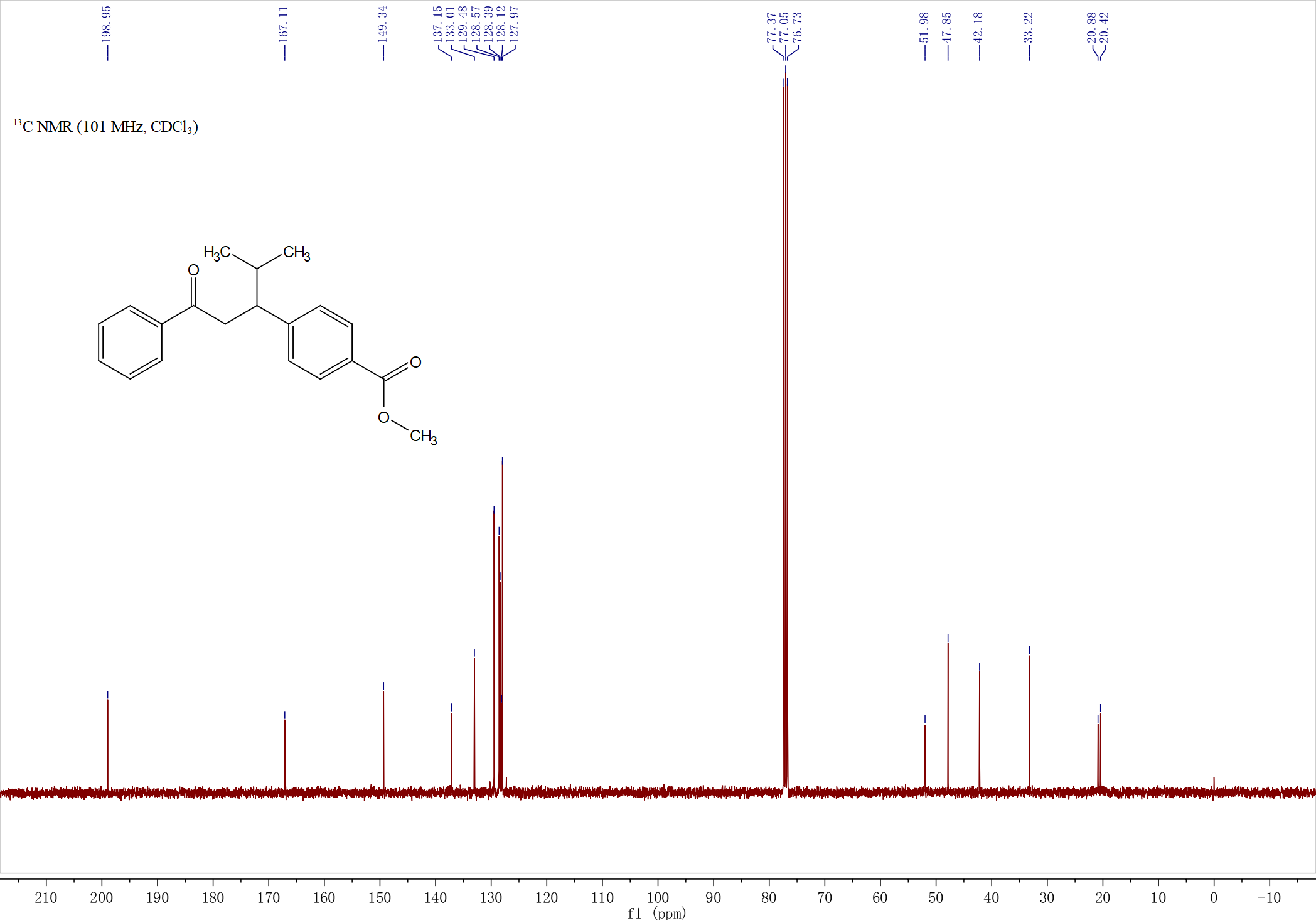


**3-(4-acetylphenyl)-4-methyl-1-phenylpentan-1-one(3j)**


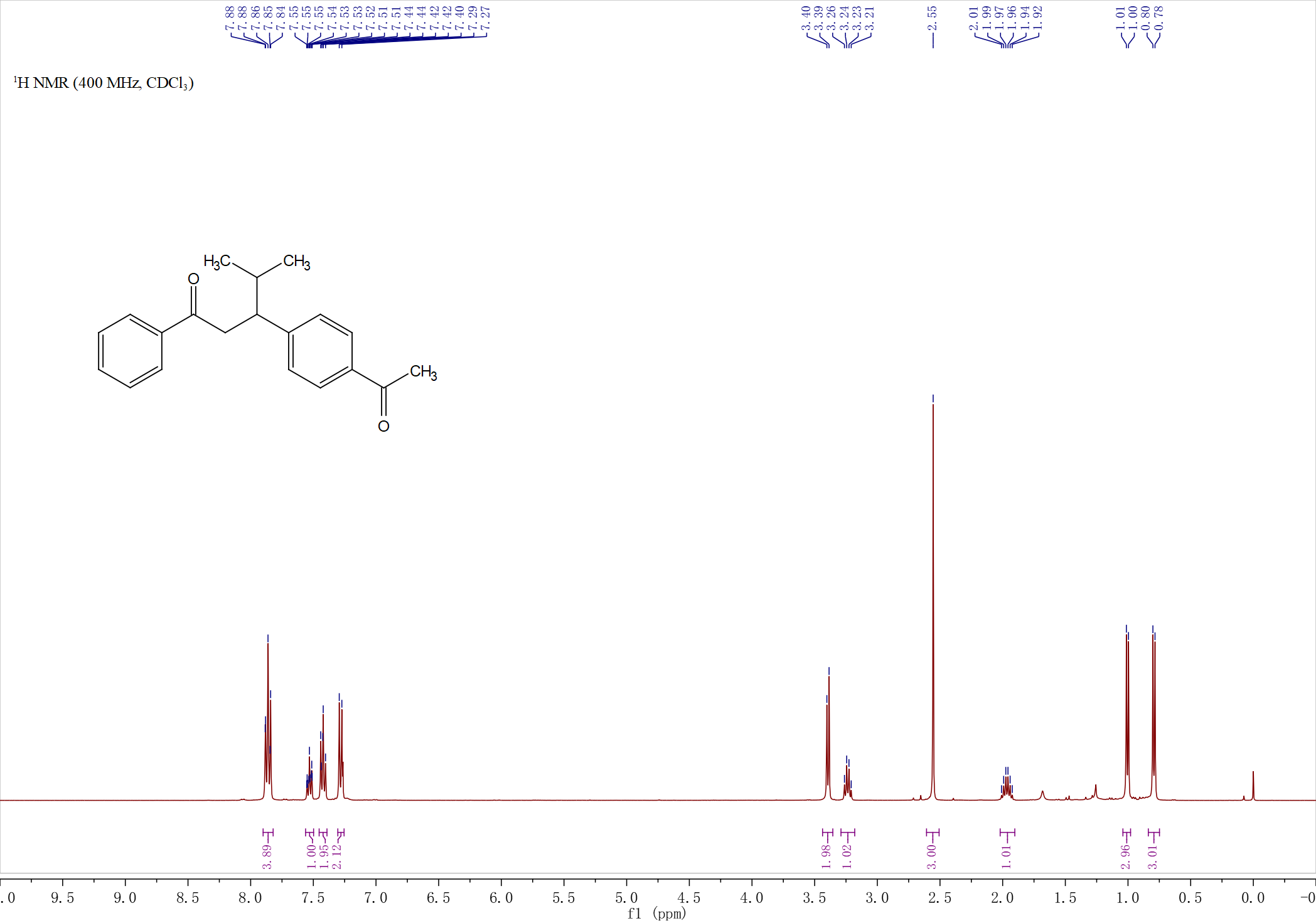


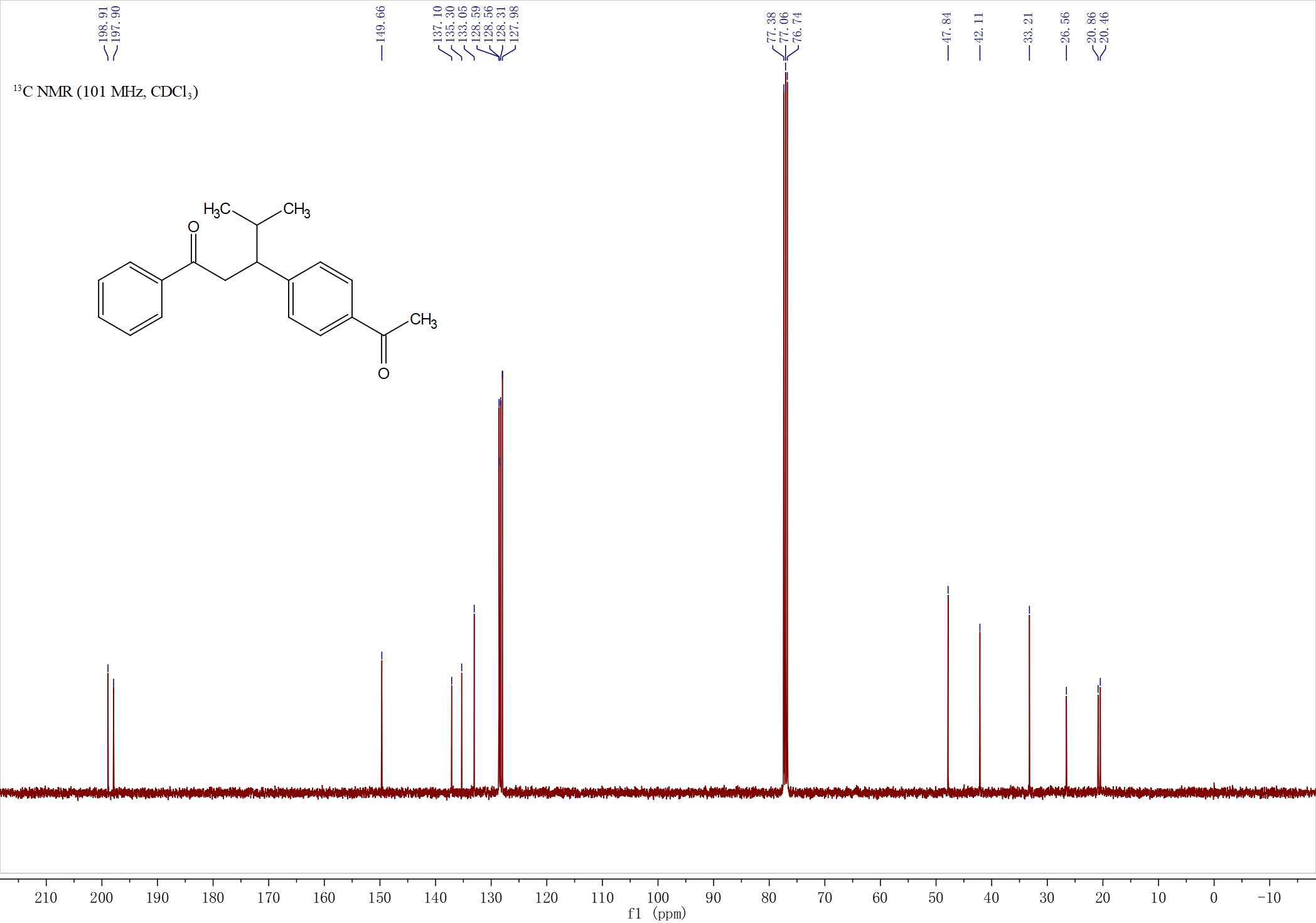


**4-methyl-1-phenyl-3-(4-(trifluoromethyl)phenyl) pentan-1-one (3k)**


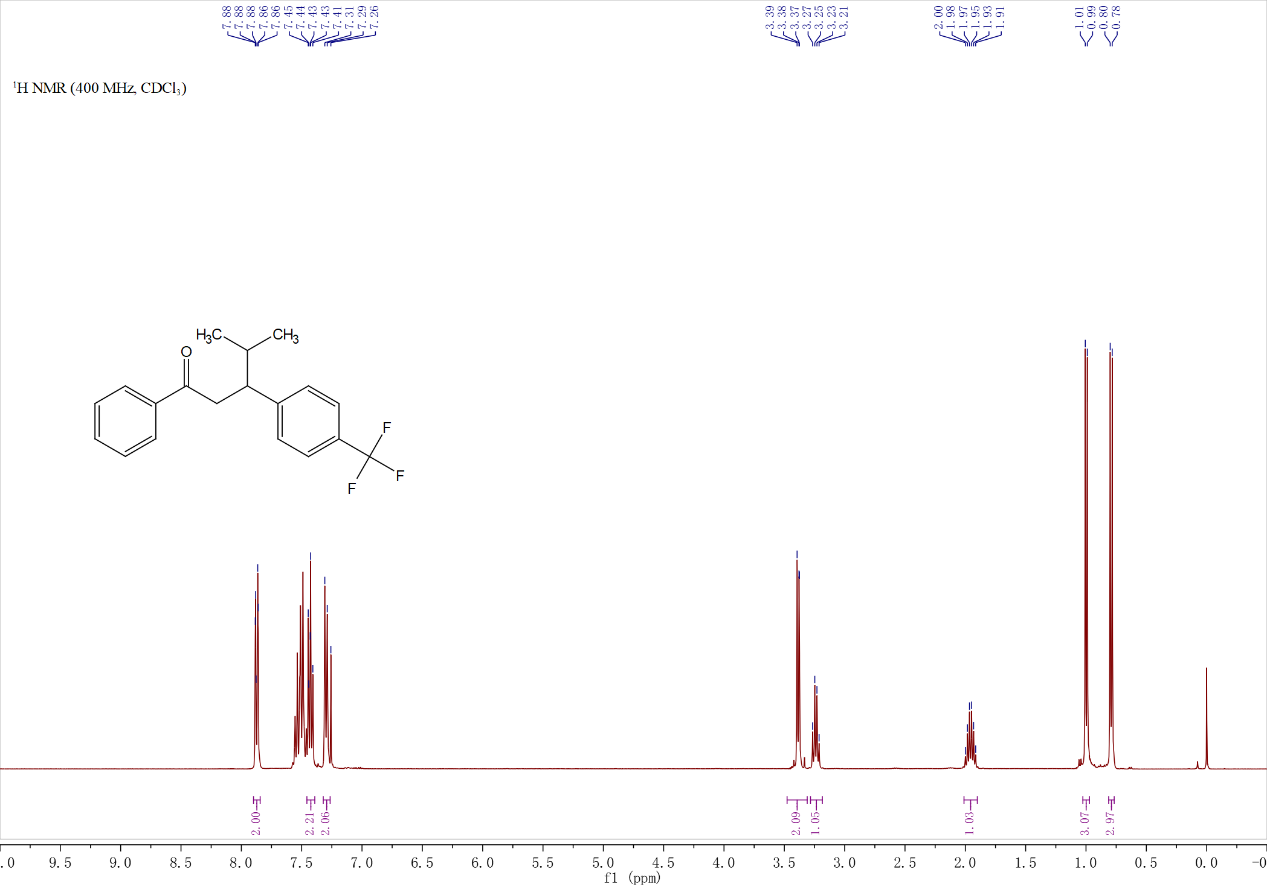


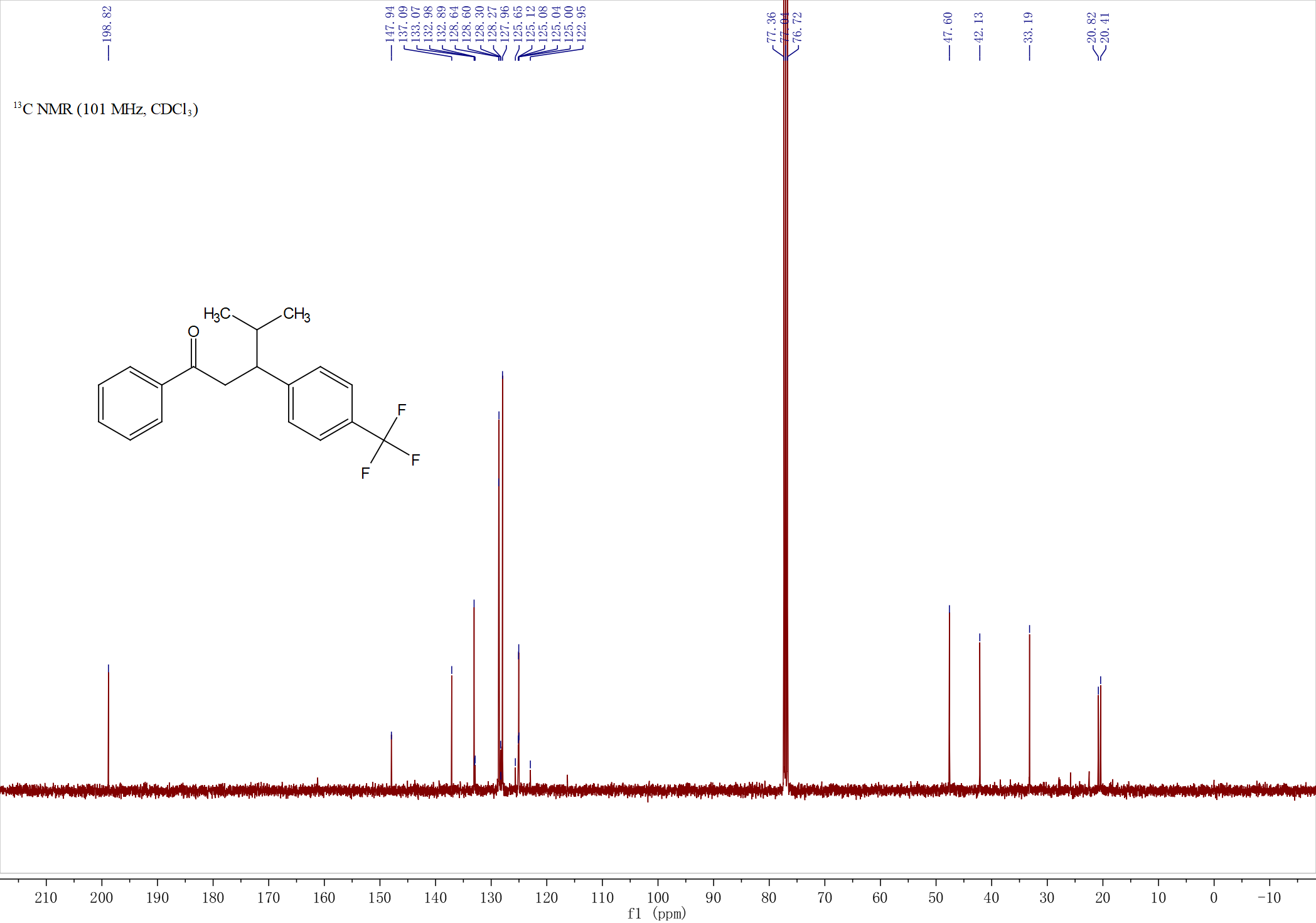


**4-(4-methyl-1-oxo-1-phenylpentan-3-yl)benzonitrile (3l)**


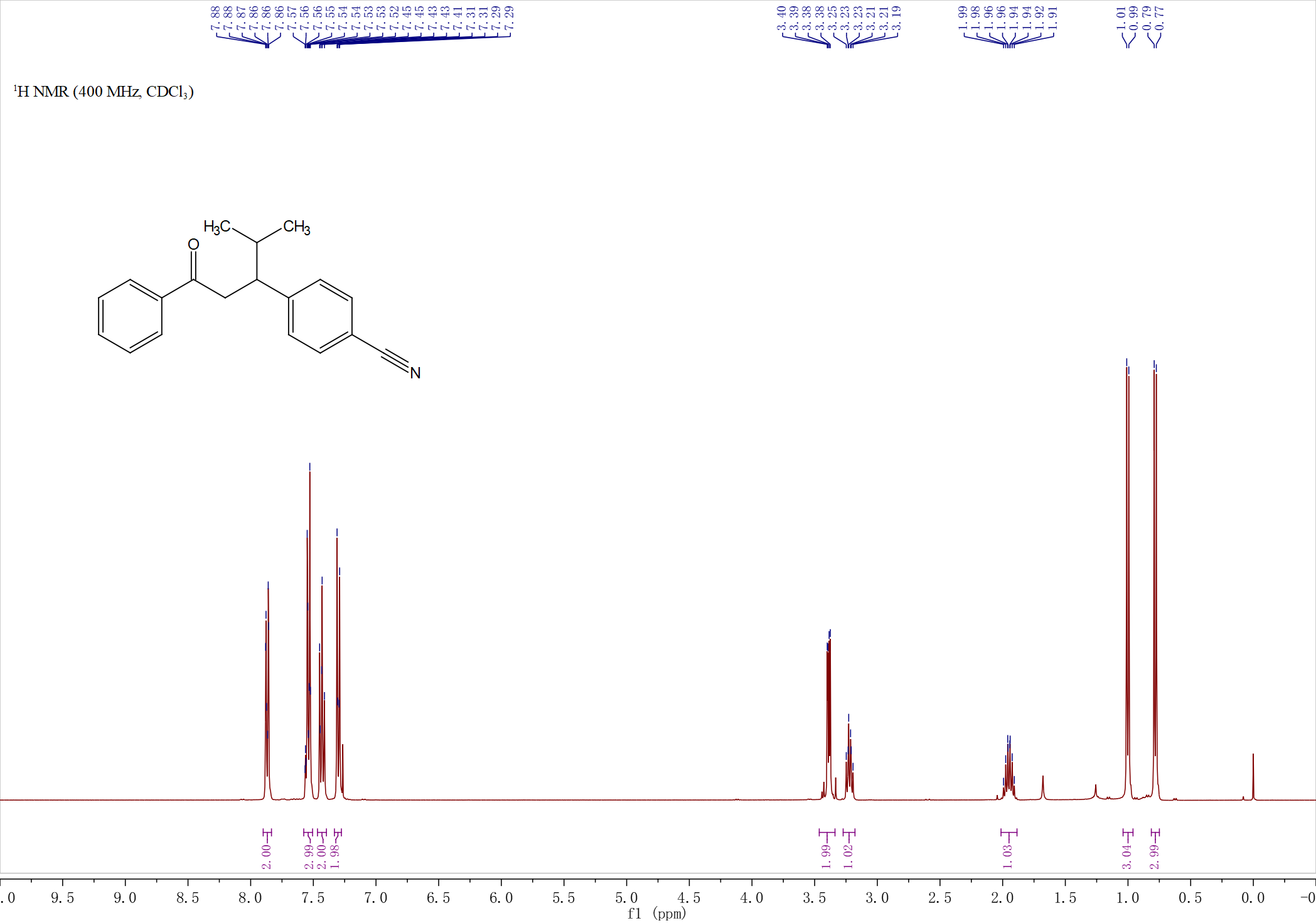


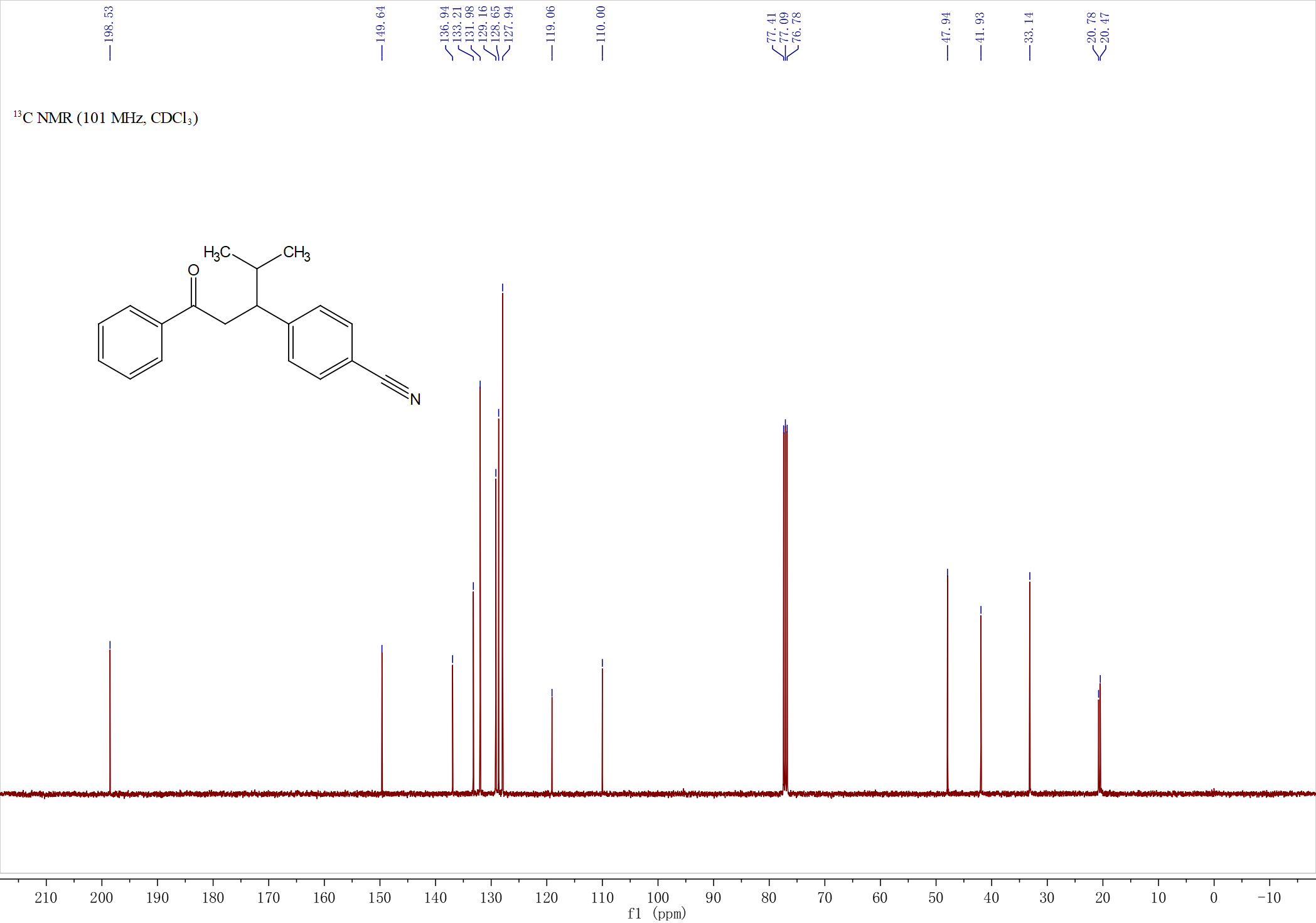


**4-(4-methyl-1-oxo-1-phenylpentan-3-yl)benzene sulfonamide (3m)**


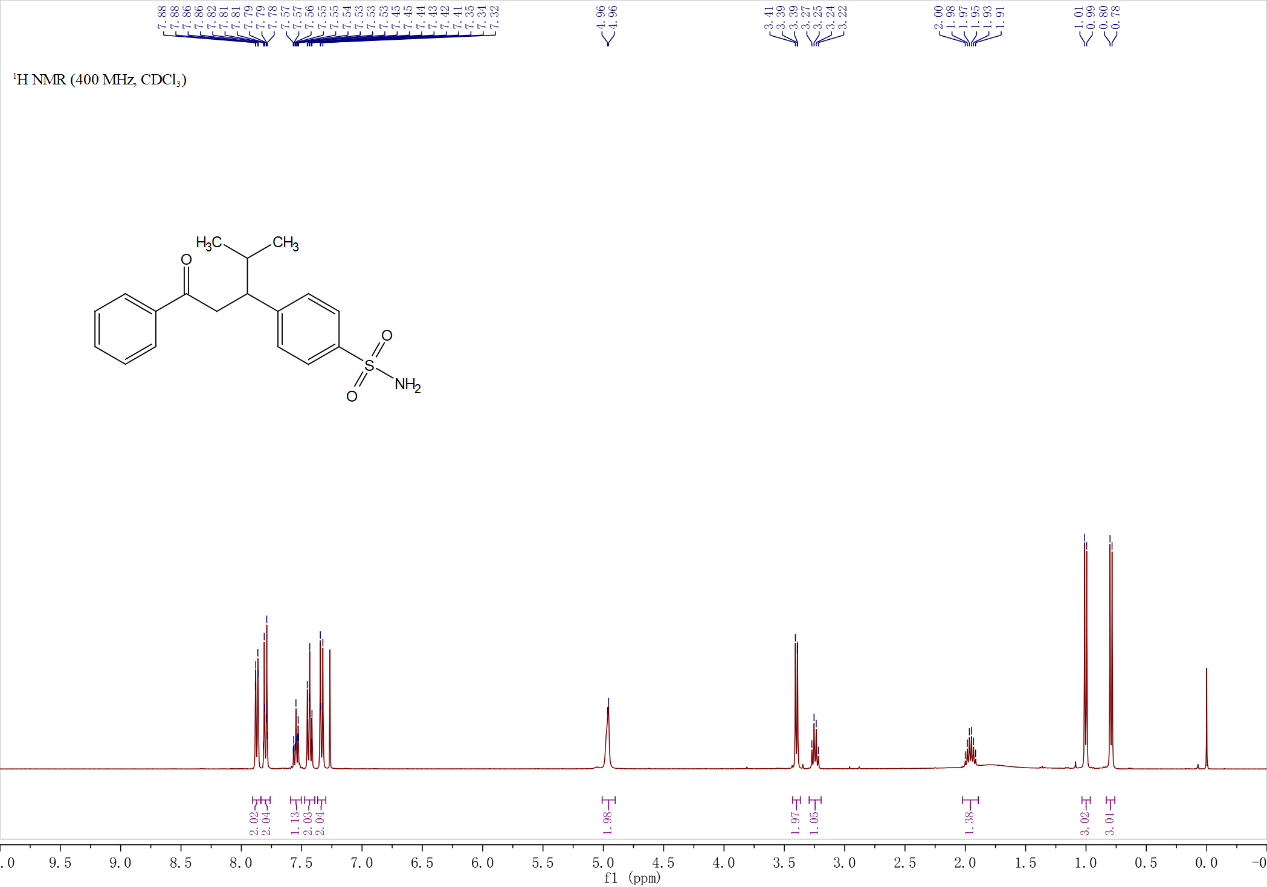


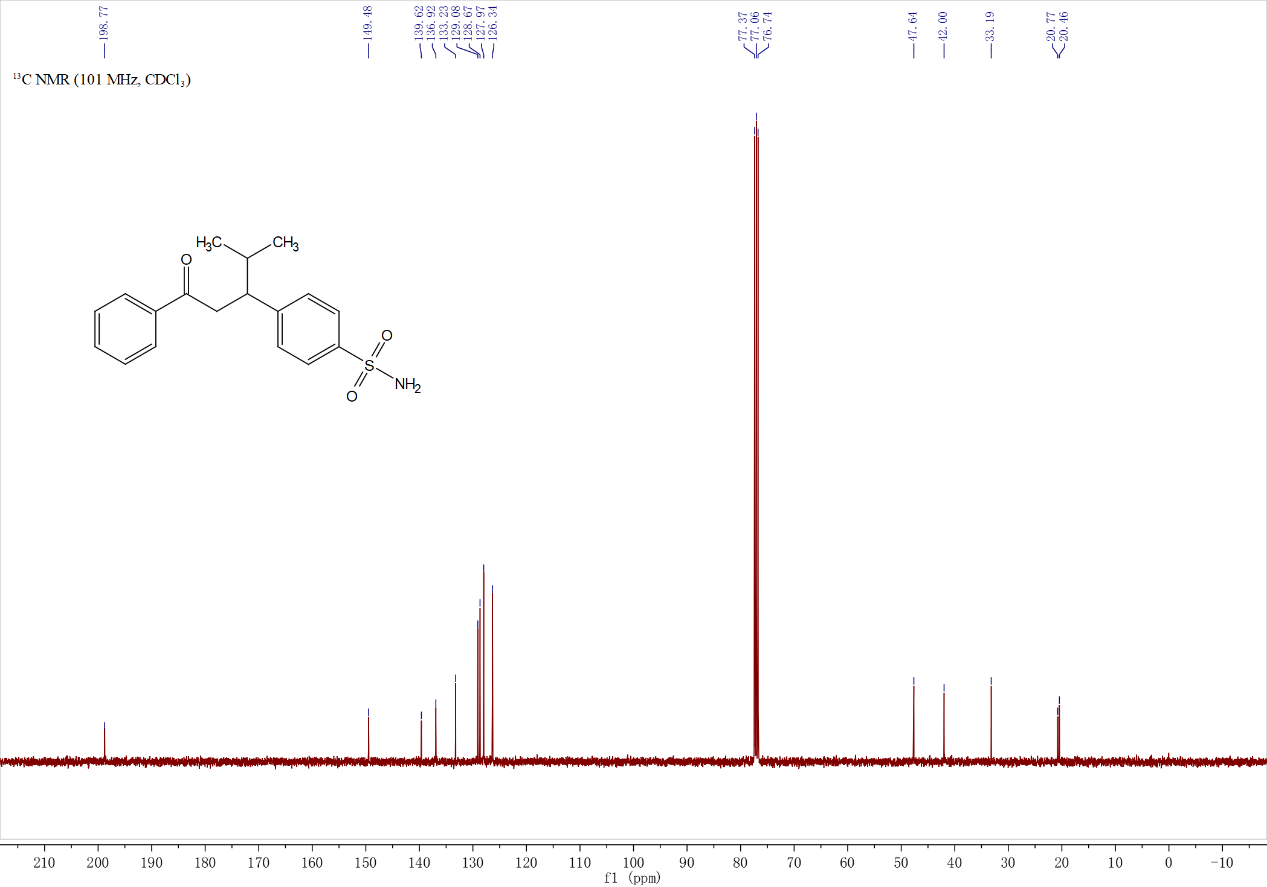


***N*-(4-(4-methyl-1-oxo-1-phenylpentan-3-yl)phenyl) acetamide (3n)**


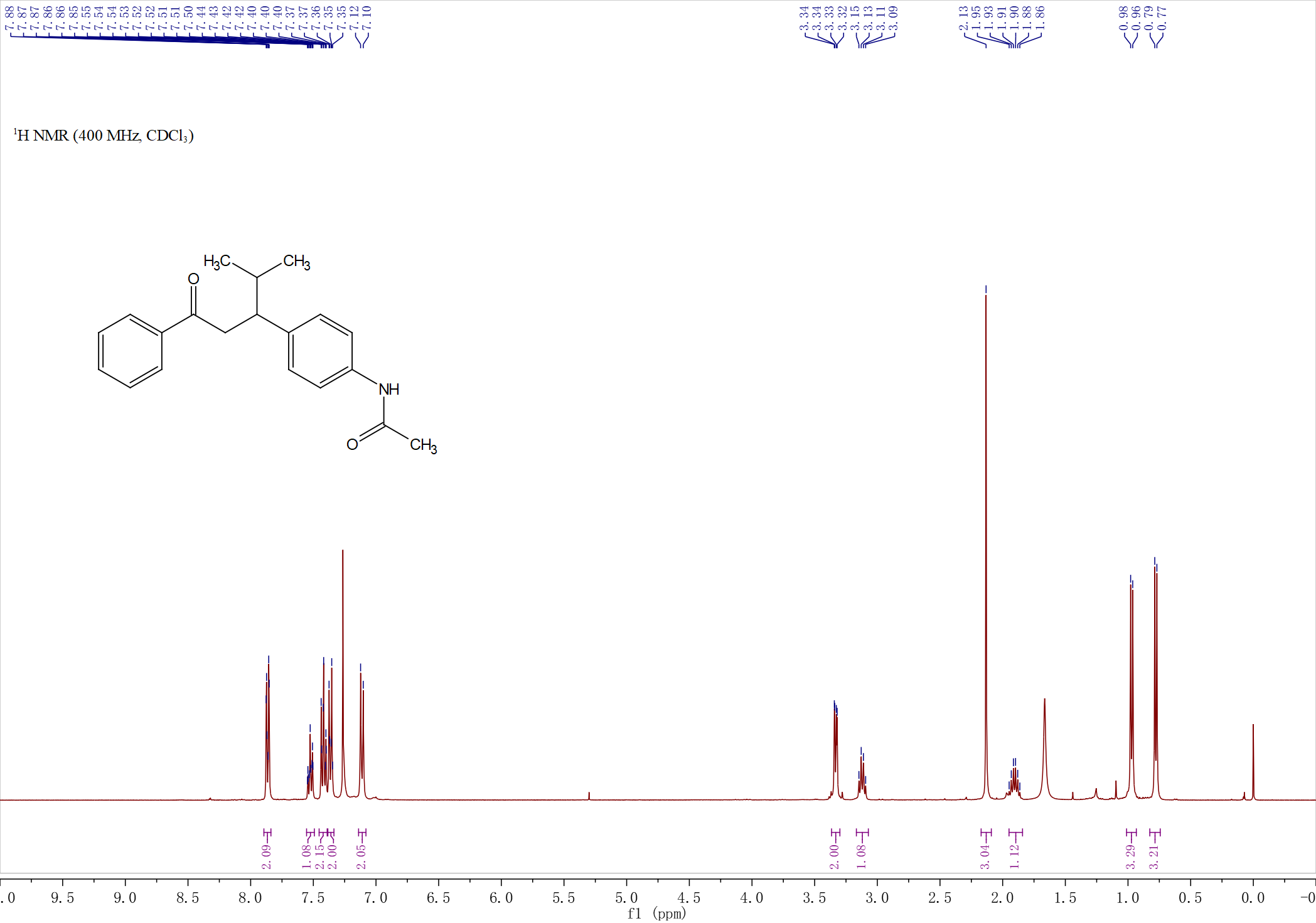


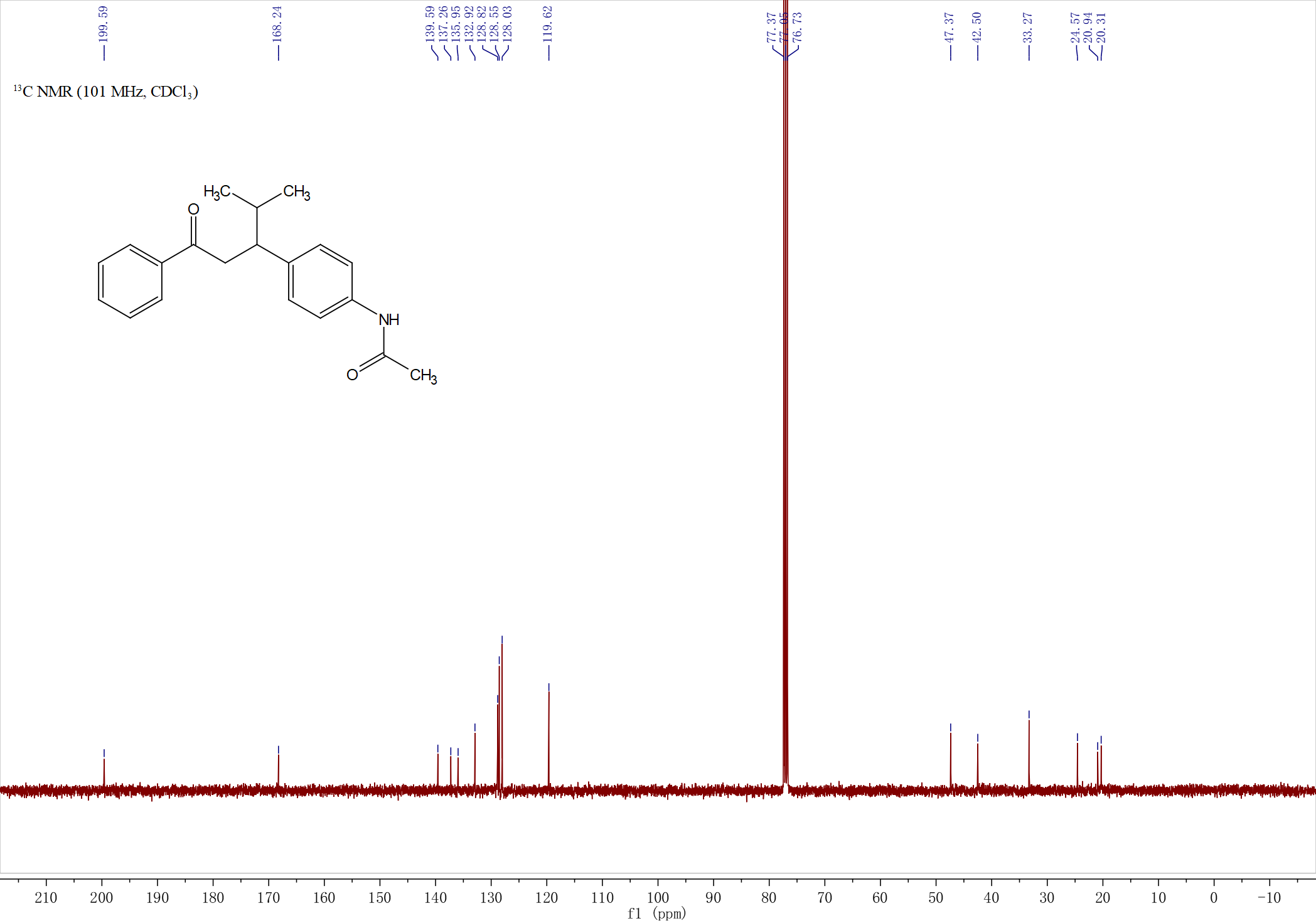


**3-(3-acetylphenyl)-4-methyl-1-phenylpentan-1-one (3o)**


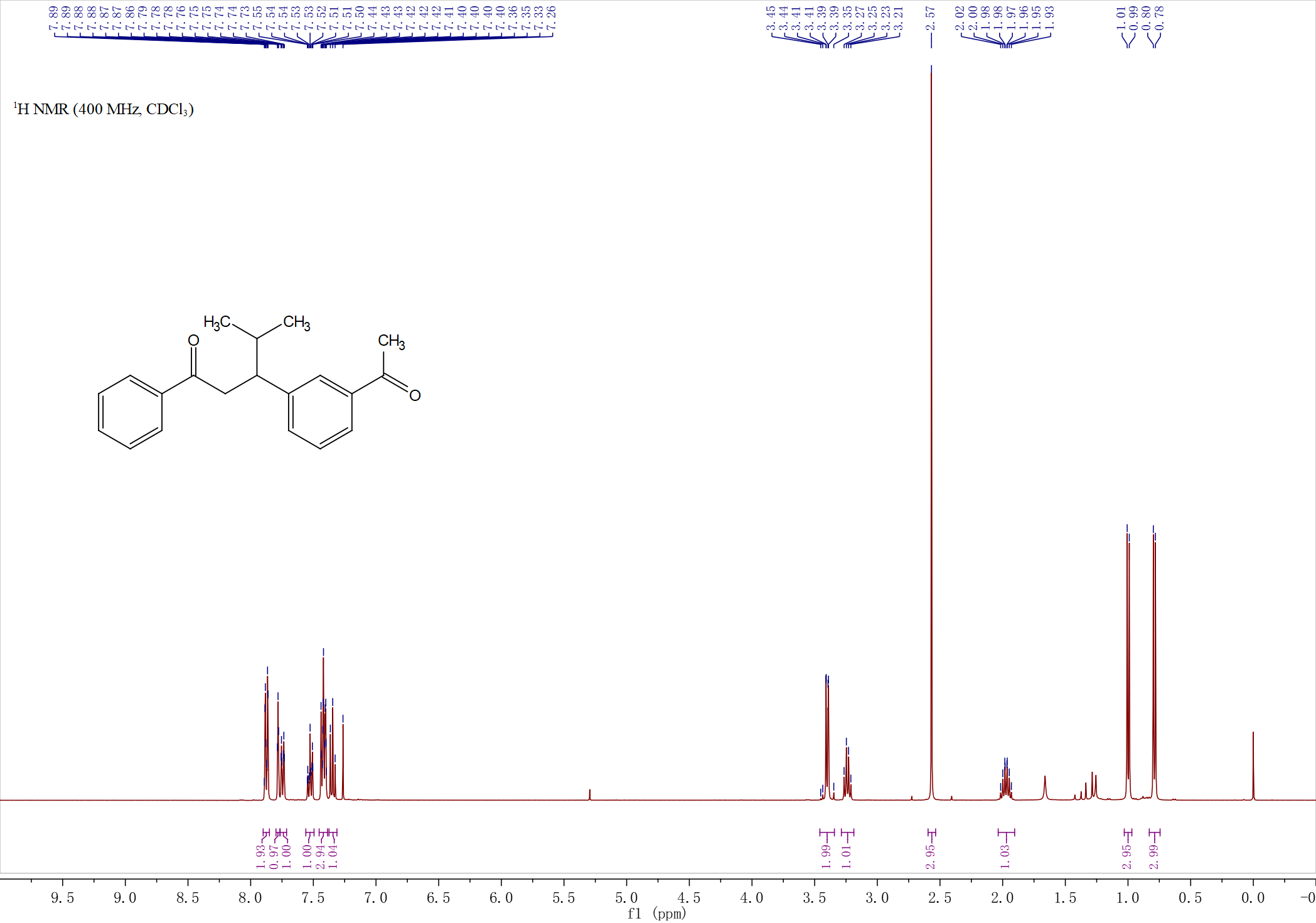


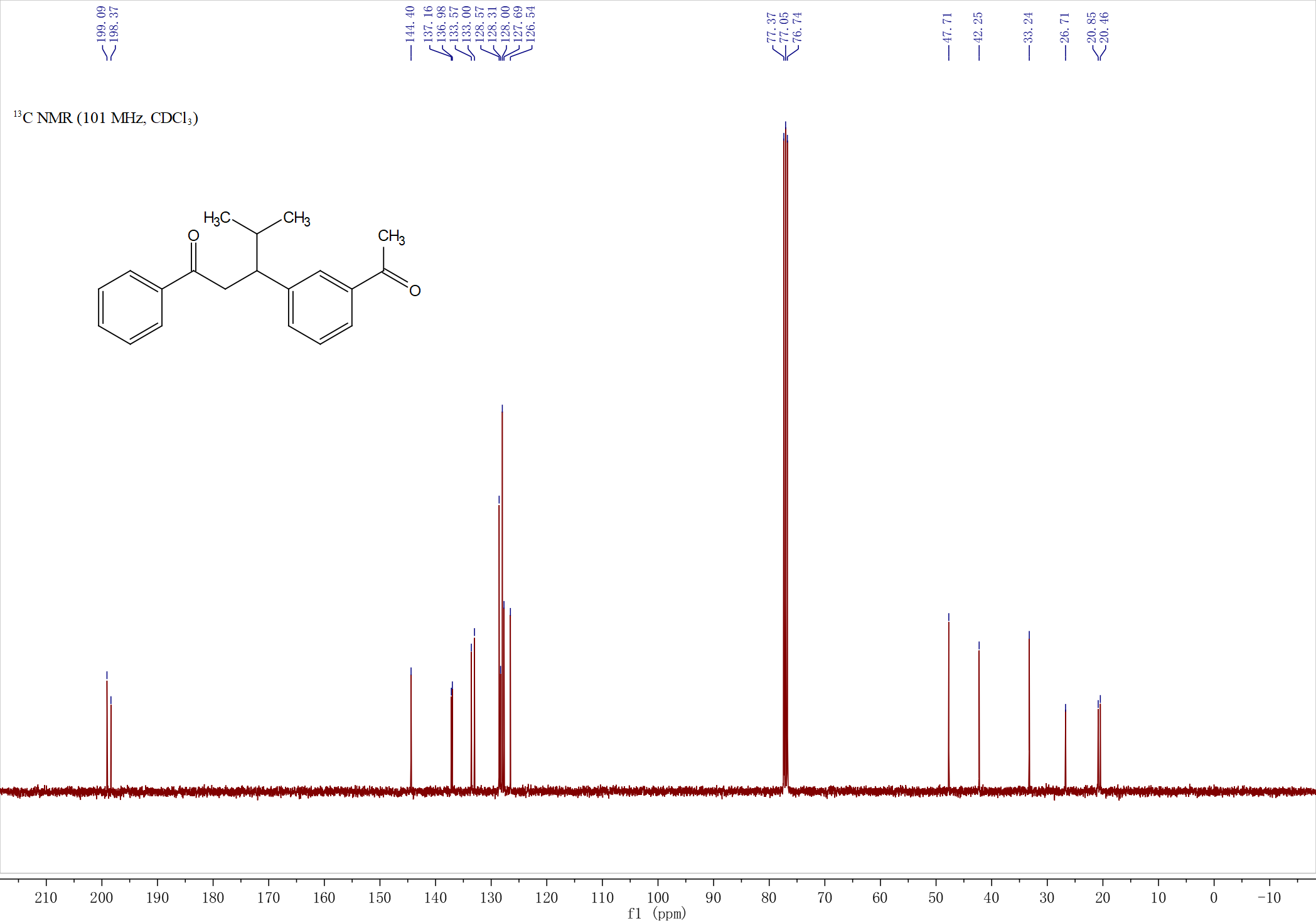


**5-(4-methyl-1-oxo-1-phenylpentan-3-yl)isobenzofuran-1(3*H*)-one (3p)**


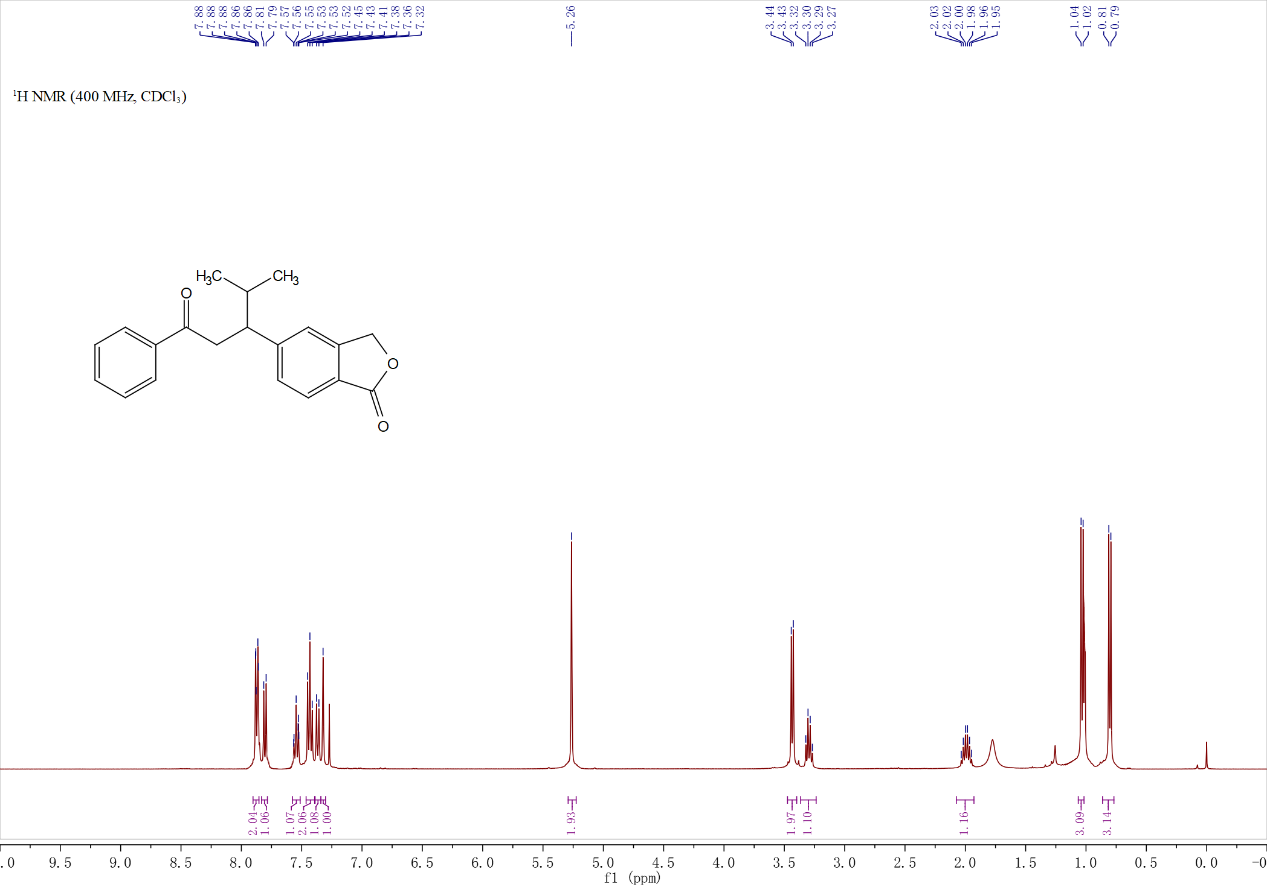


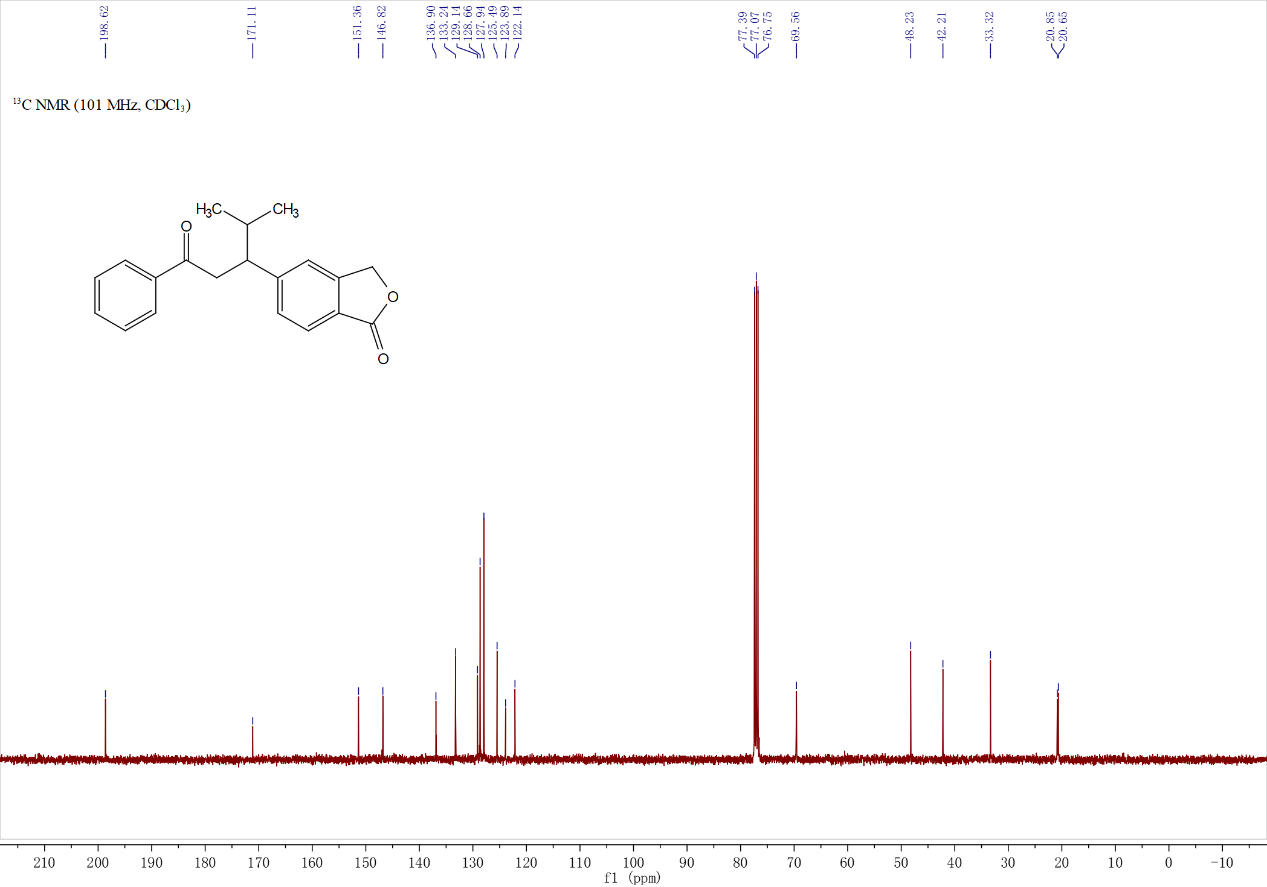


**3-(9*H*-fluoren-2-yl)-4-methyl-1-phenylpentan-1-one (3q)**


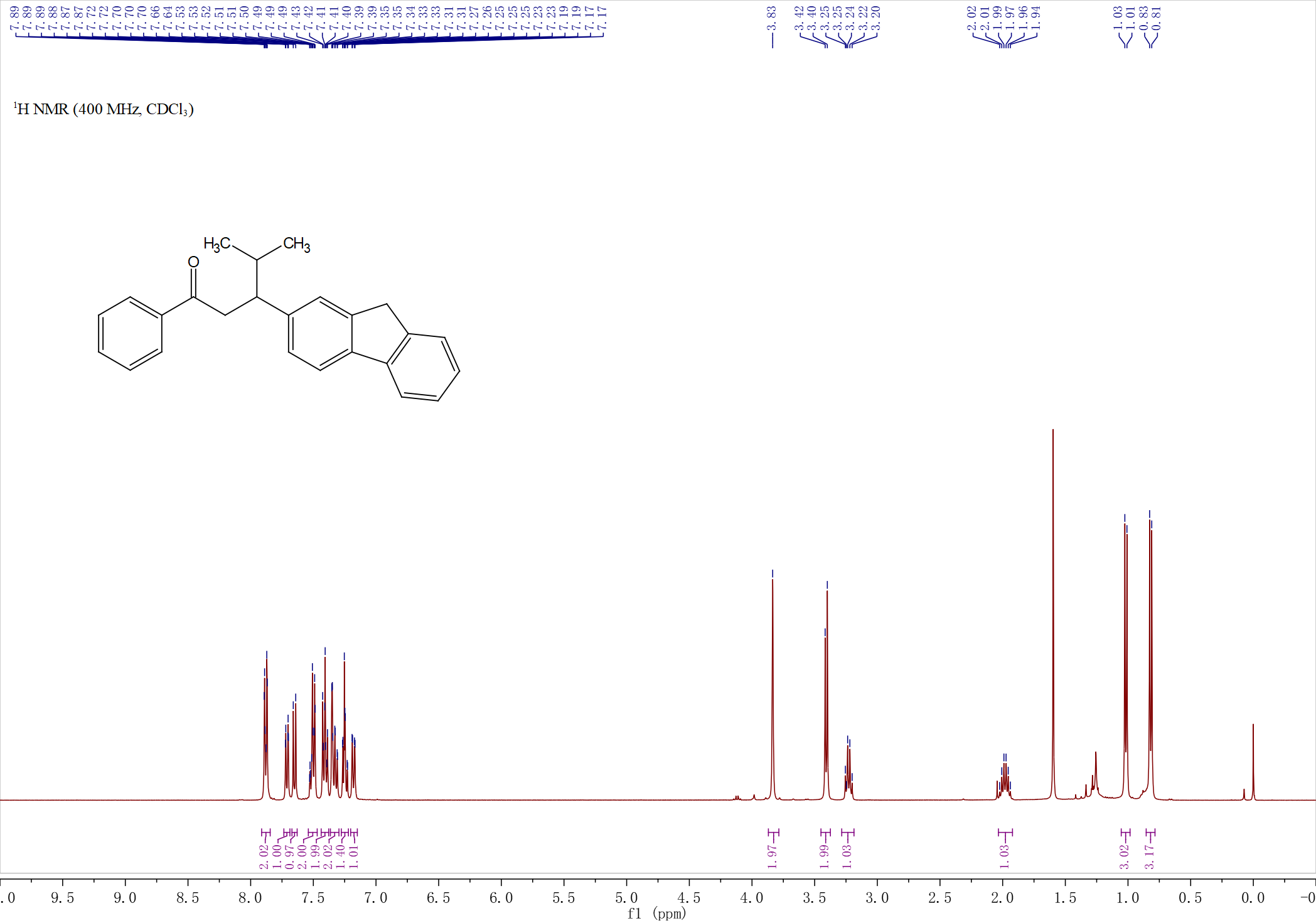


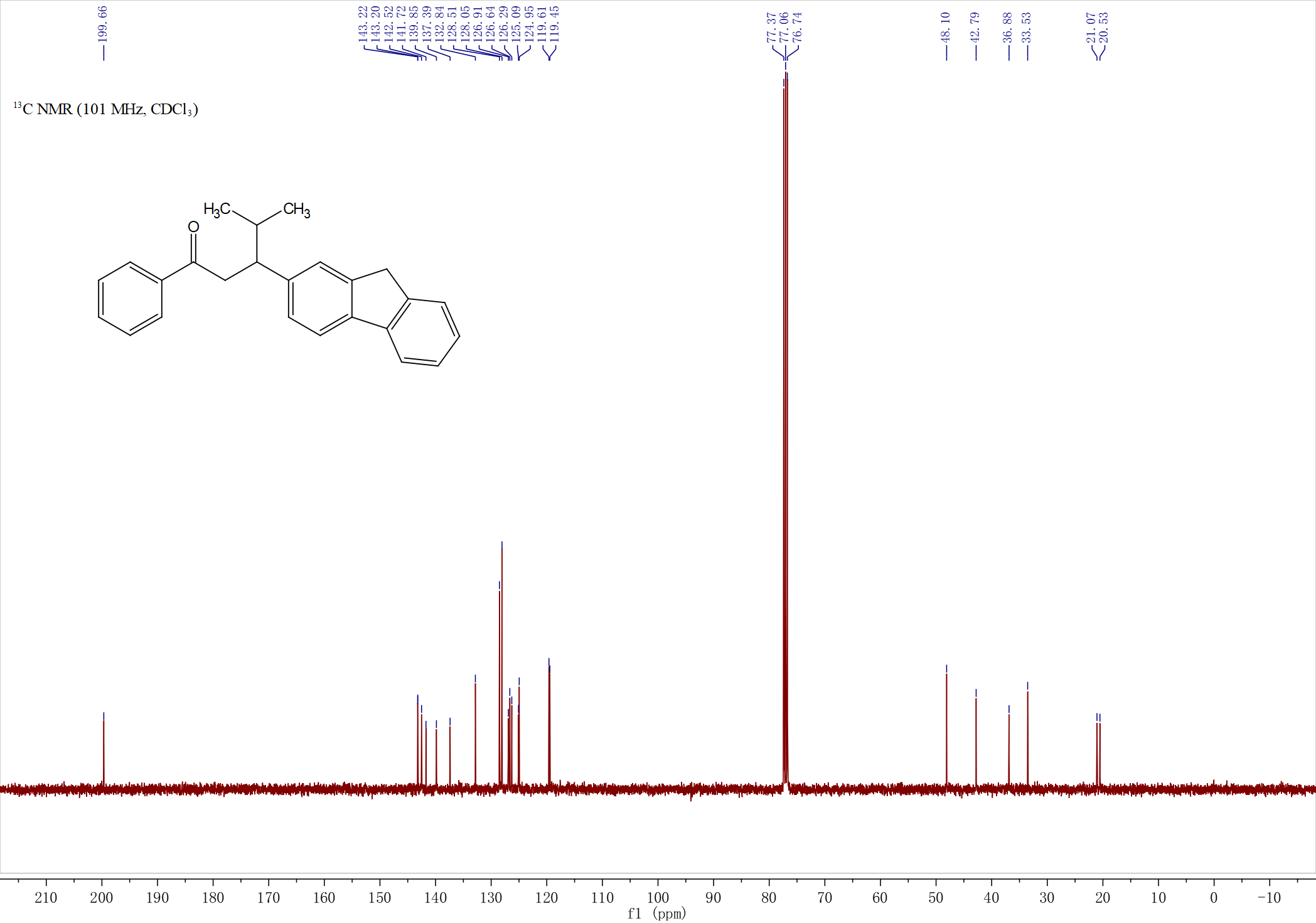


**4-methyl-3-(naphthalen-2-yl)-1-phenylpentan-1-one (3r)**


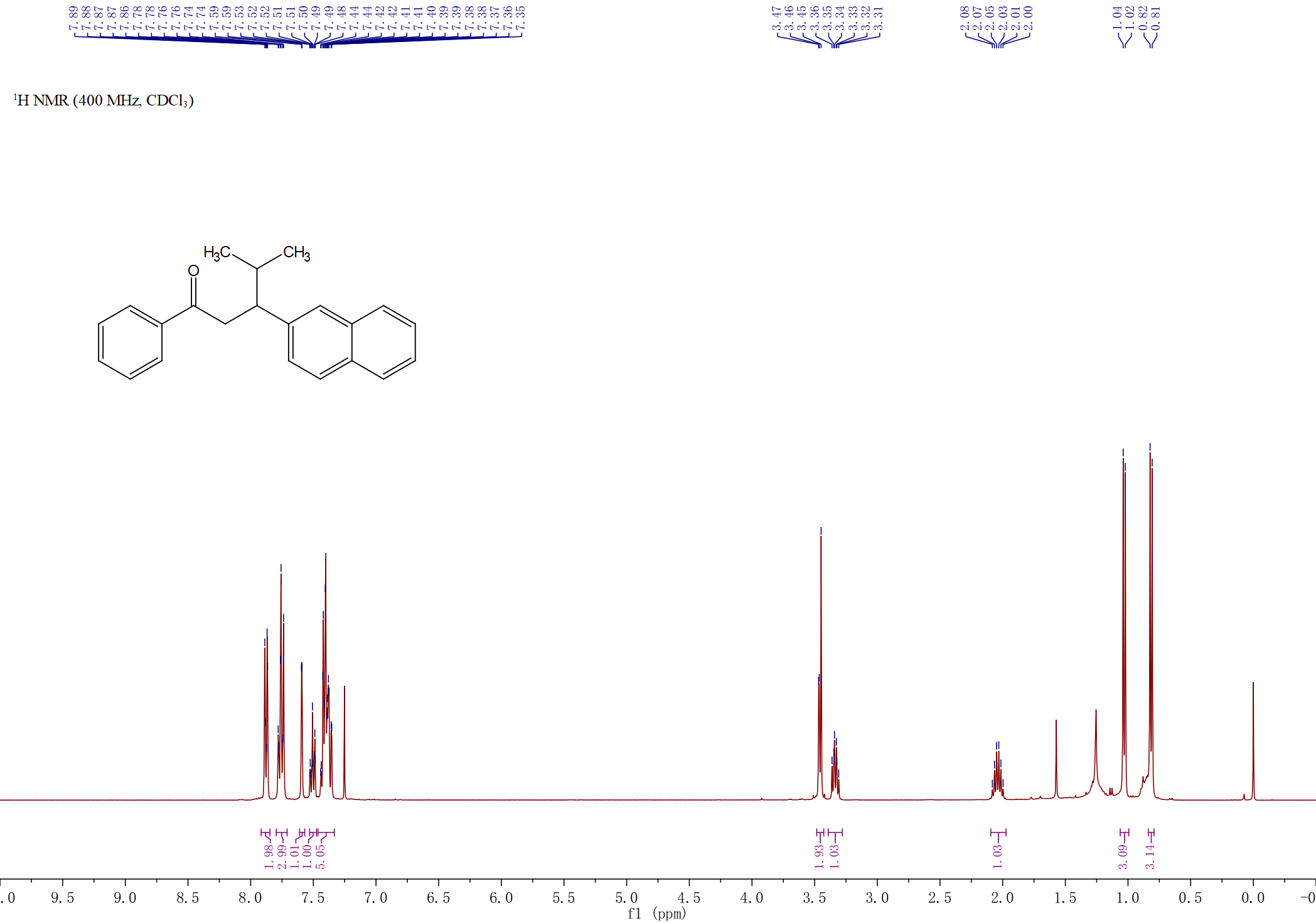


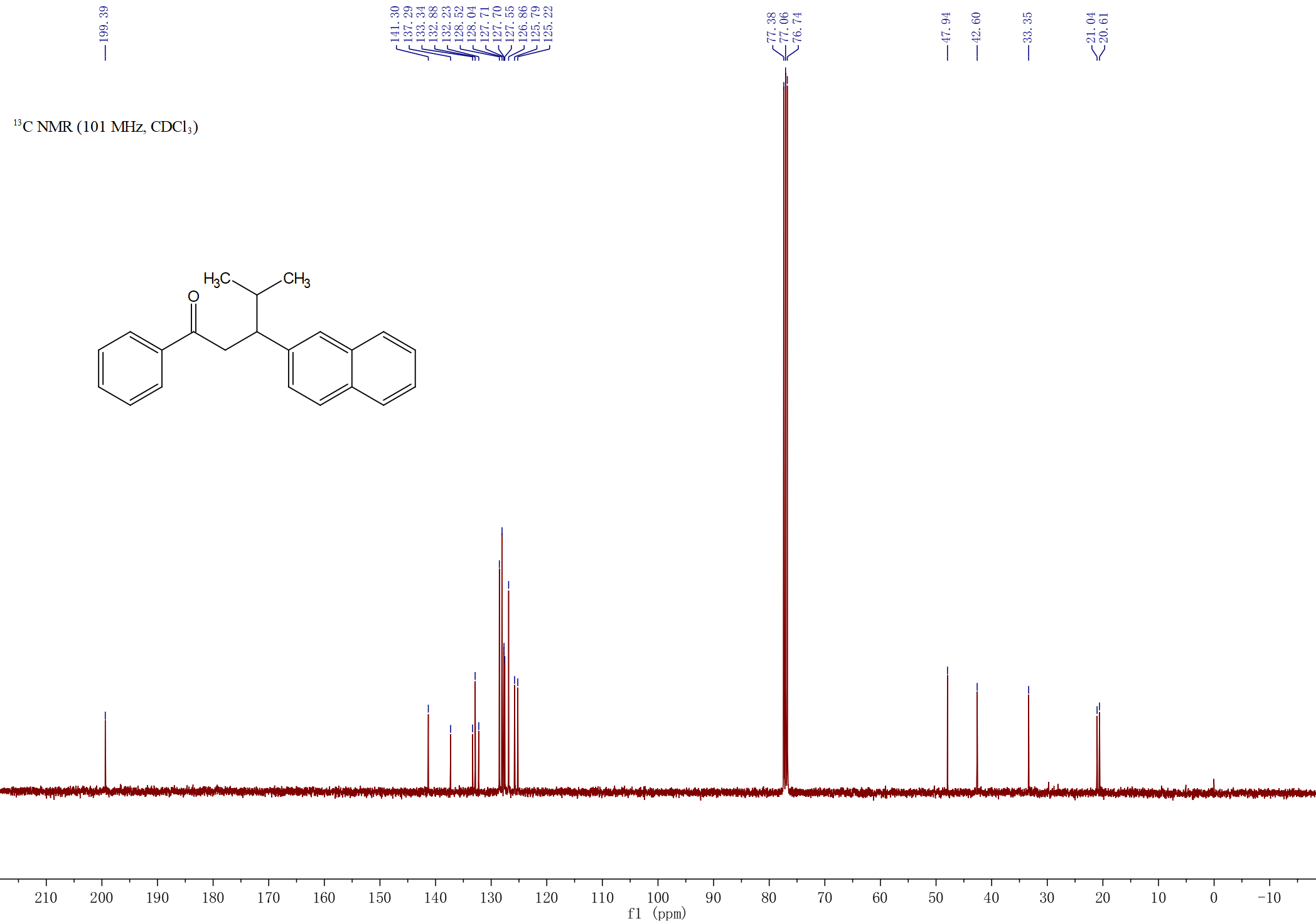


**3-(6-methoxynaphthalen-2-yl)-4-methyl-1-phenylpentan-1-one (3s)**


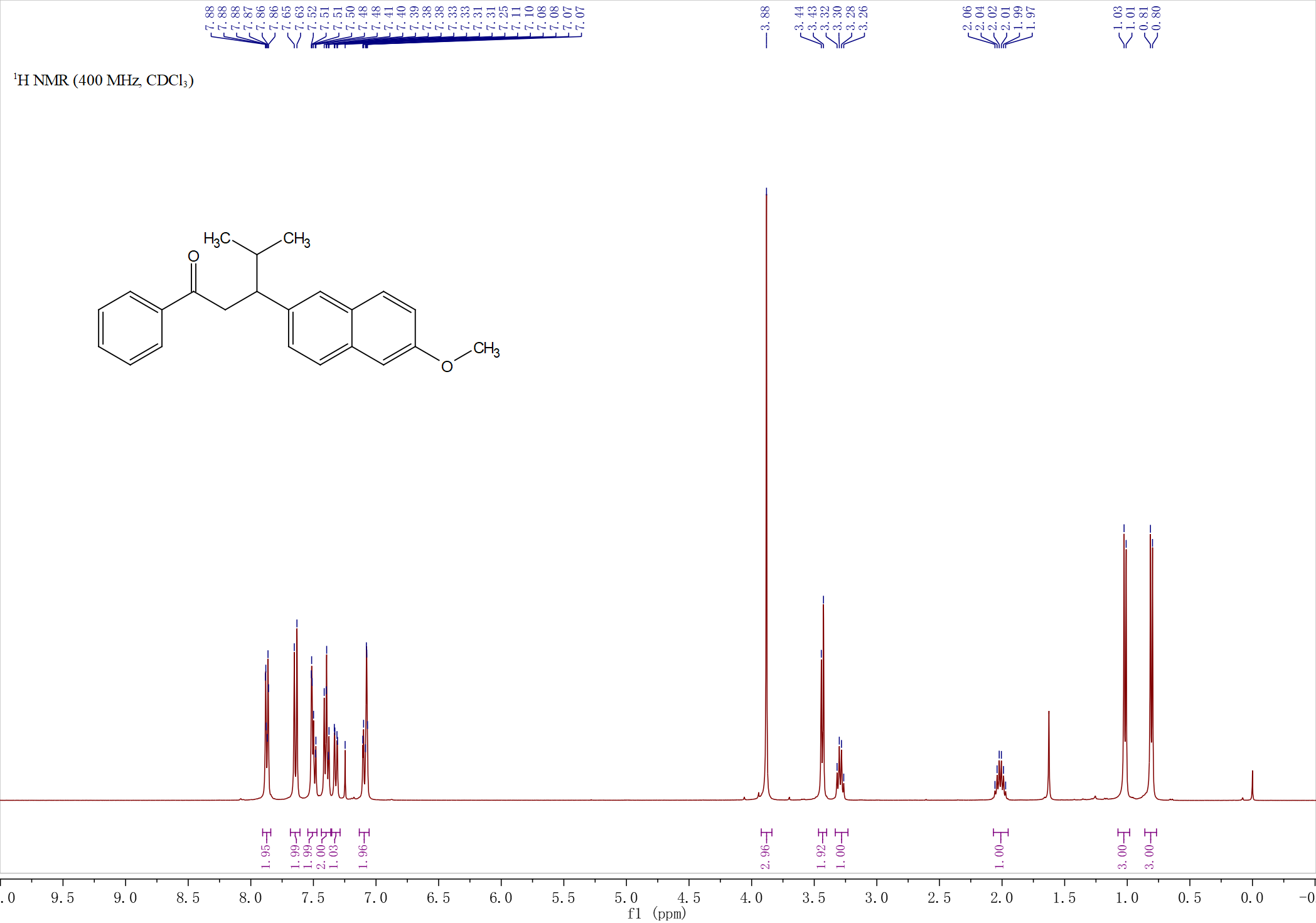


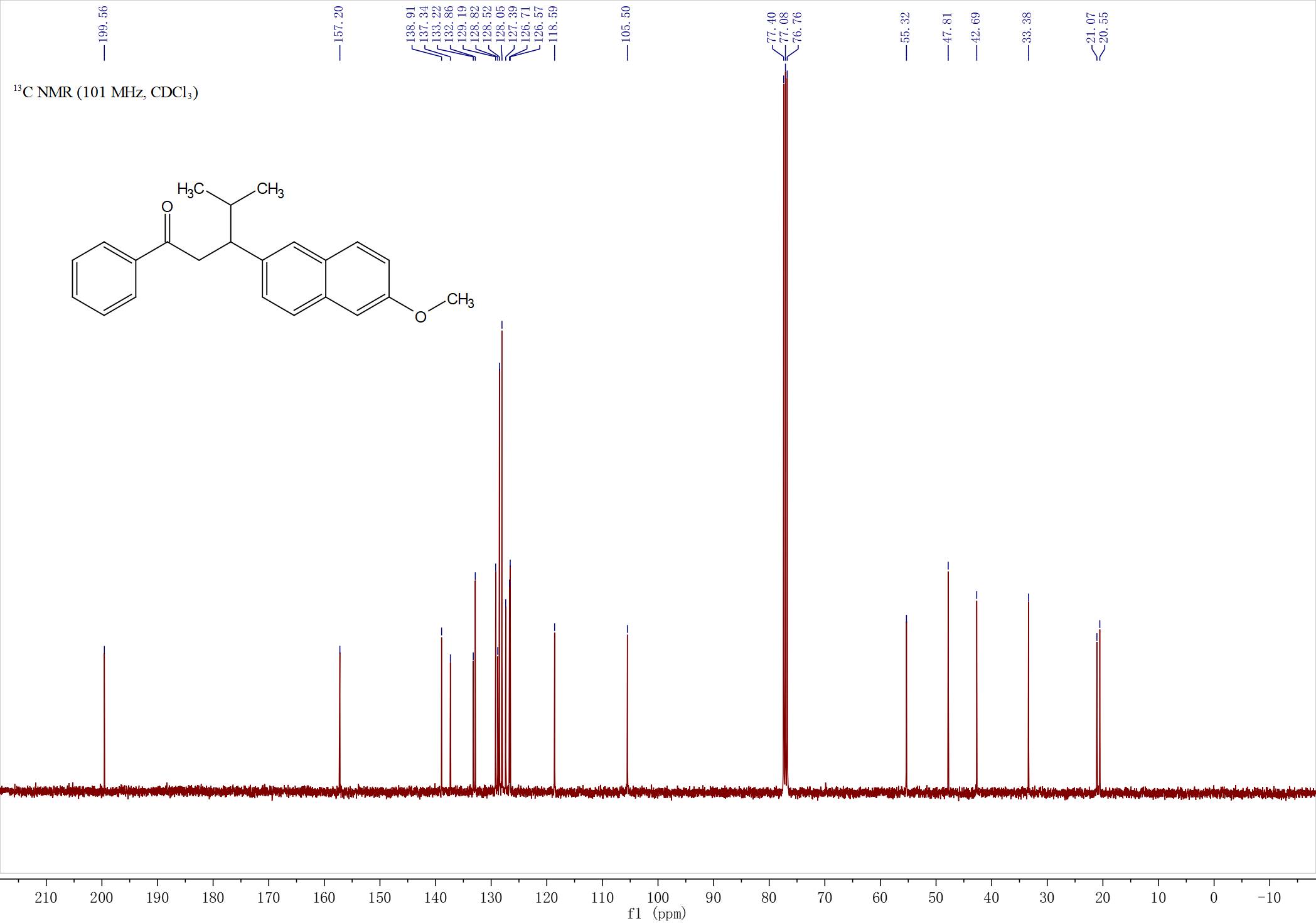


***tert*-butyl-6-(4-methyl-1-oxo-1-phenylpentan-3-yl)-1*H*-indazole-1-carboxylate (3t)**


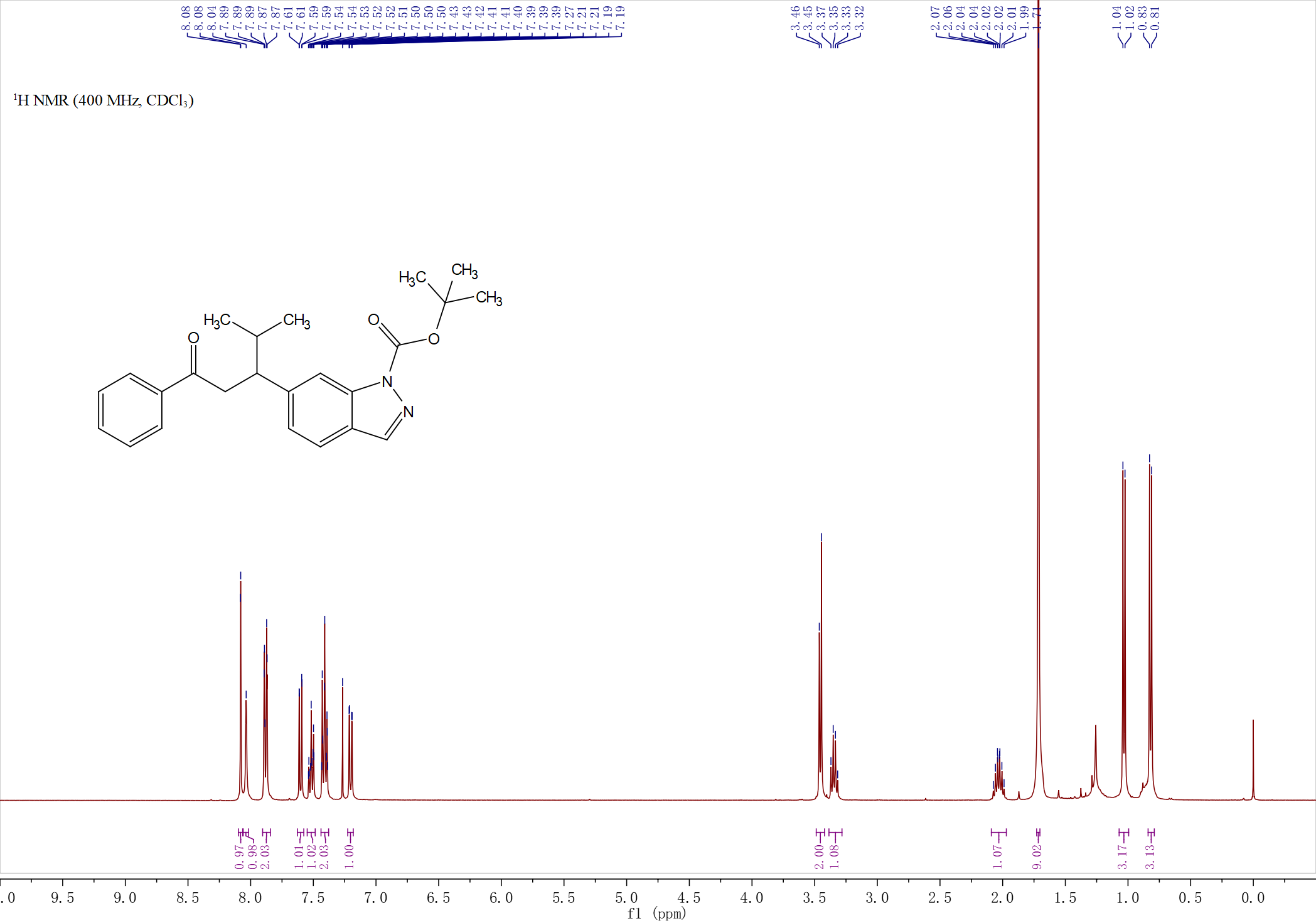


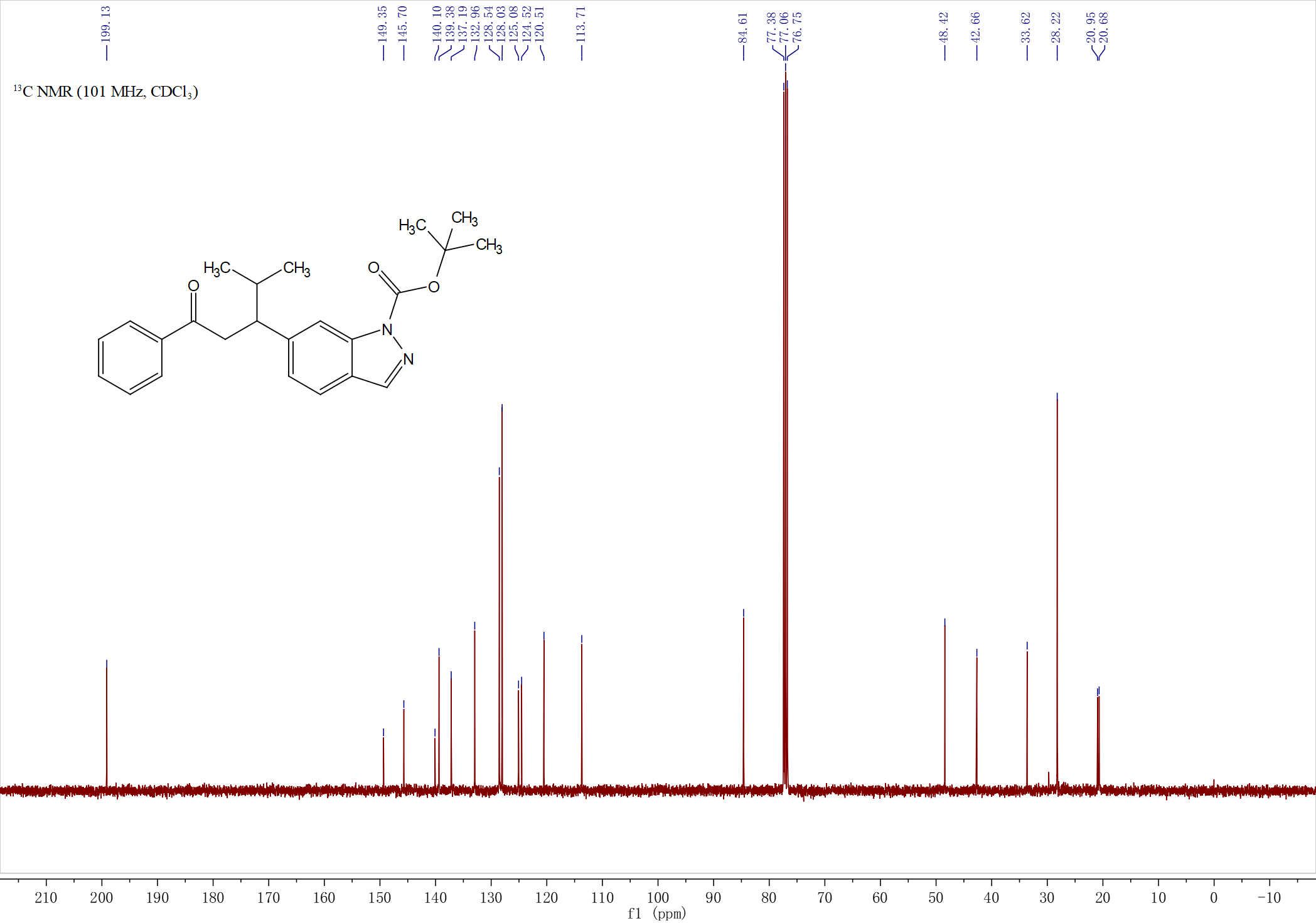


**3-(4-methoxybenzyl)-6-(4-methyl-1-oxo-1-phenylpentan-3-yl)quinazolin-4(3*H*)-one (3u)**


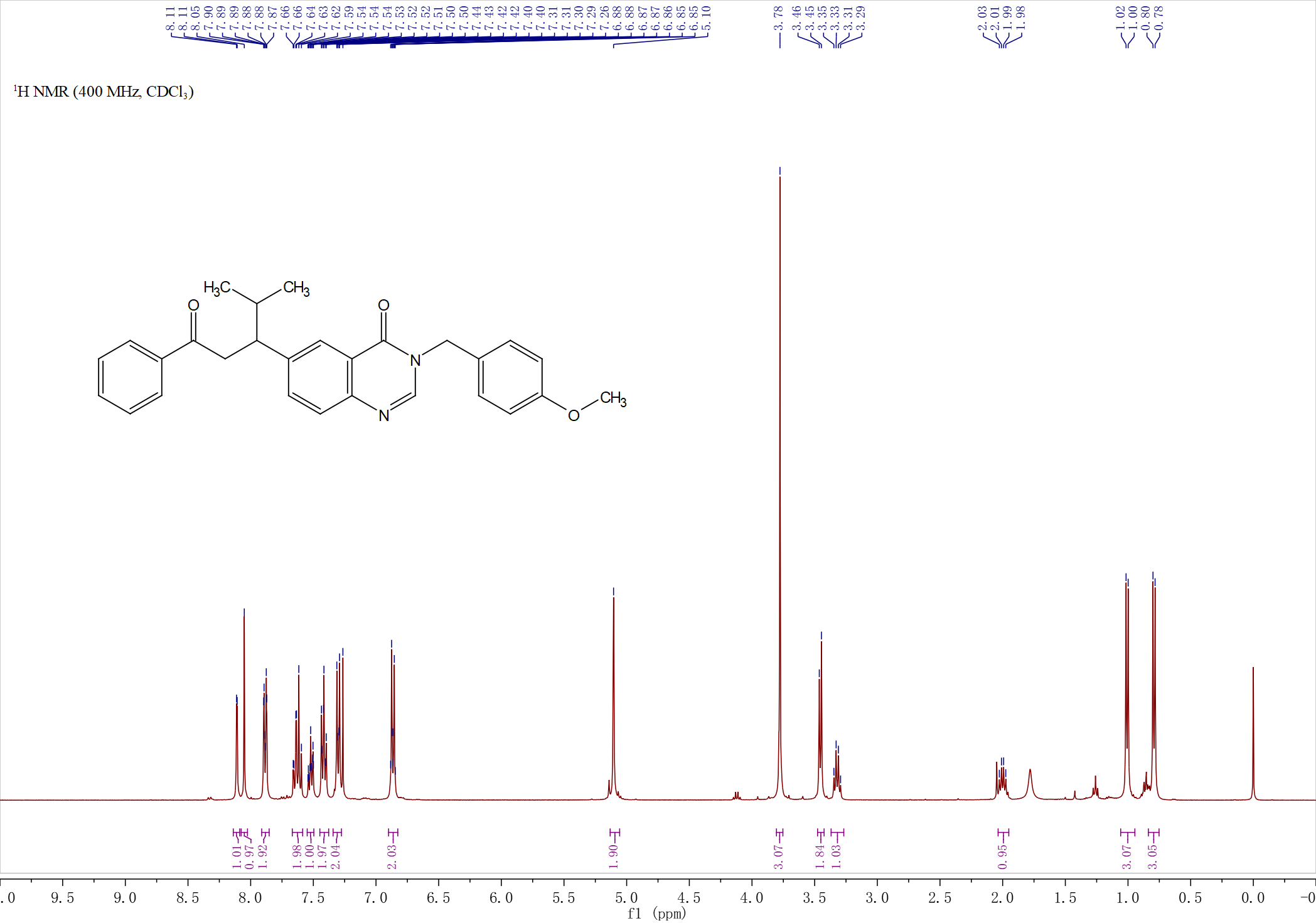


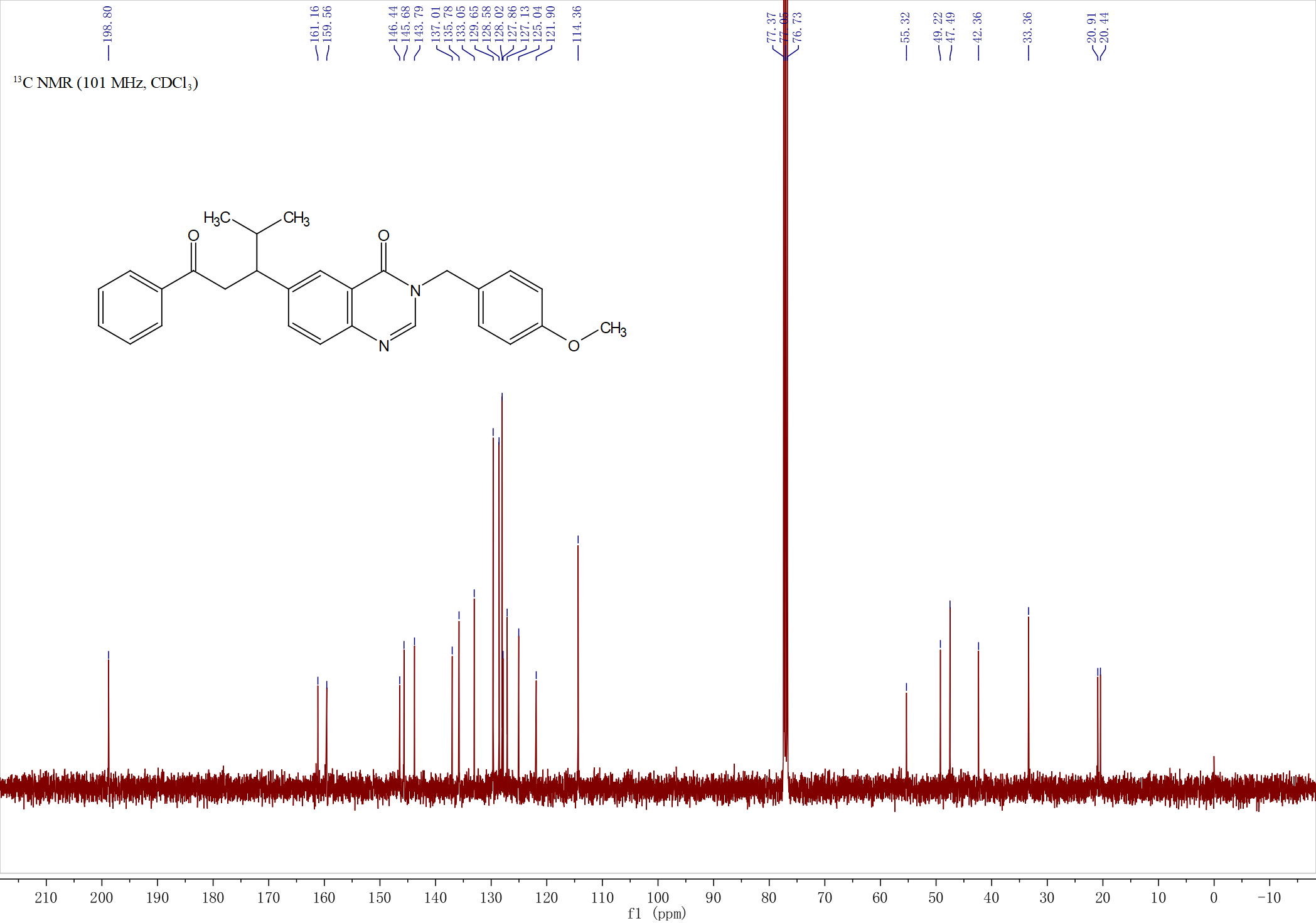


**(*E*)-1,3-diphenyl-5-(*p*-tolyl)pent-4-en-1-one (3v)**


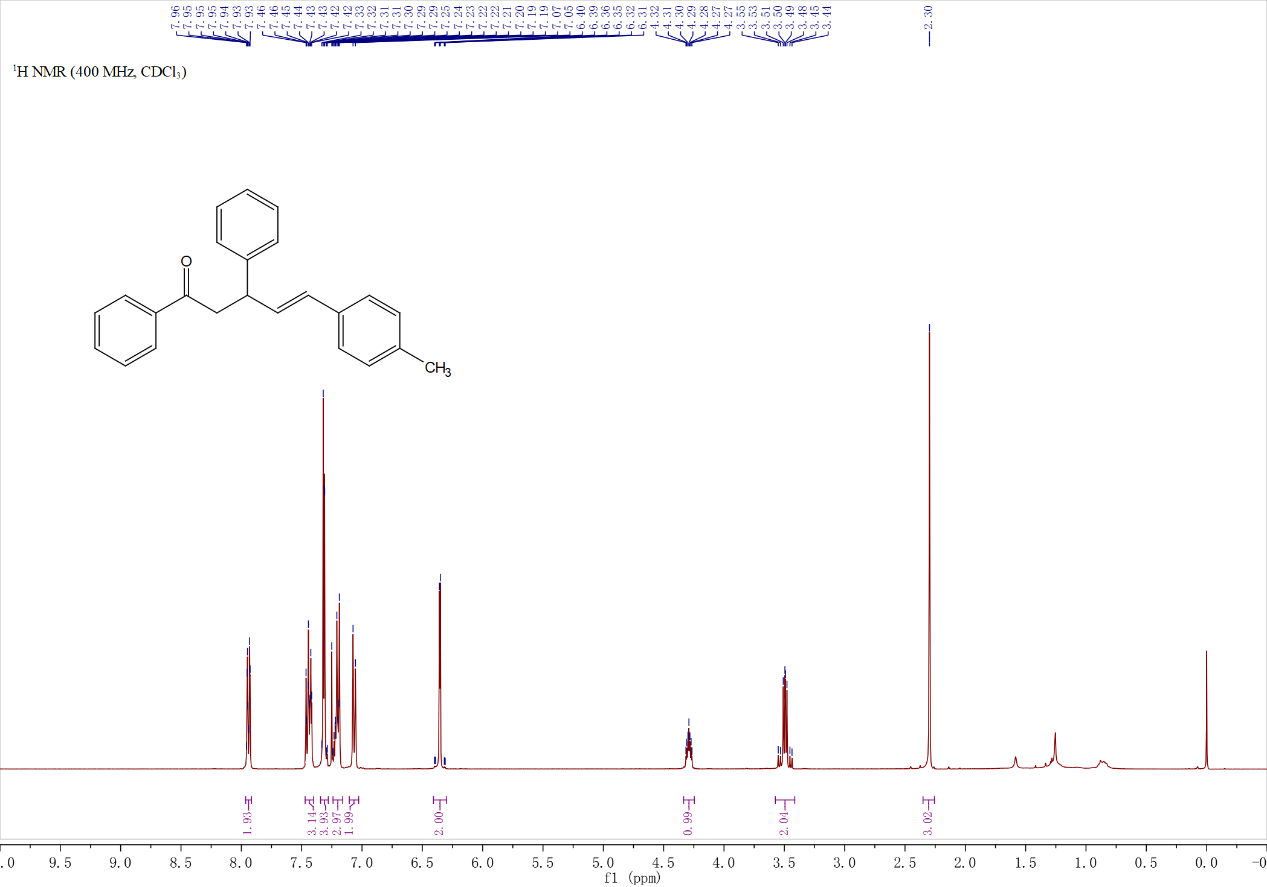


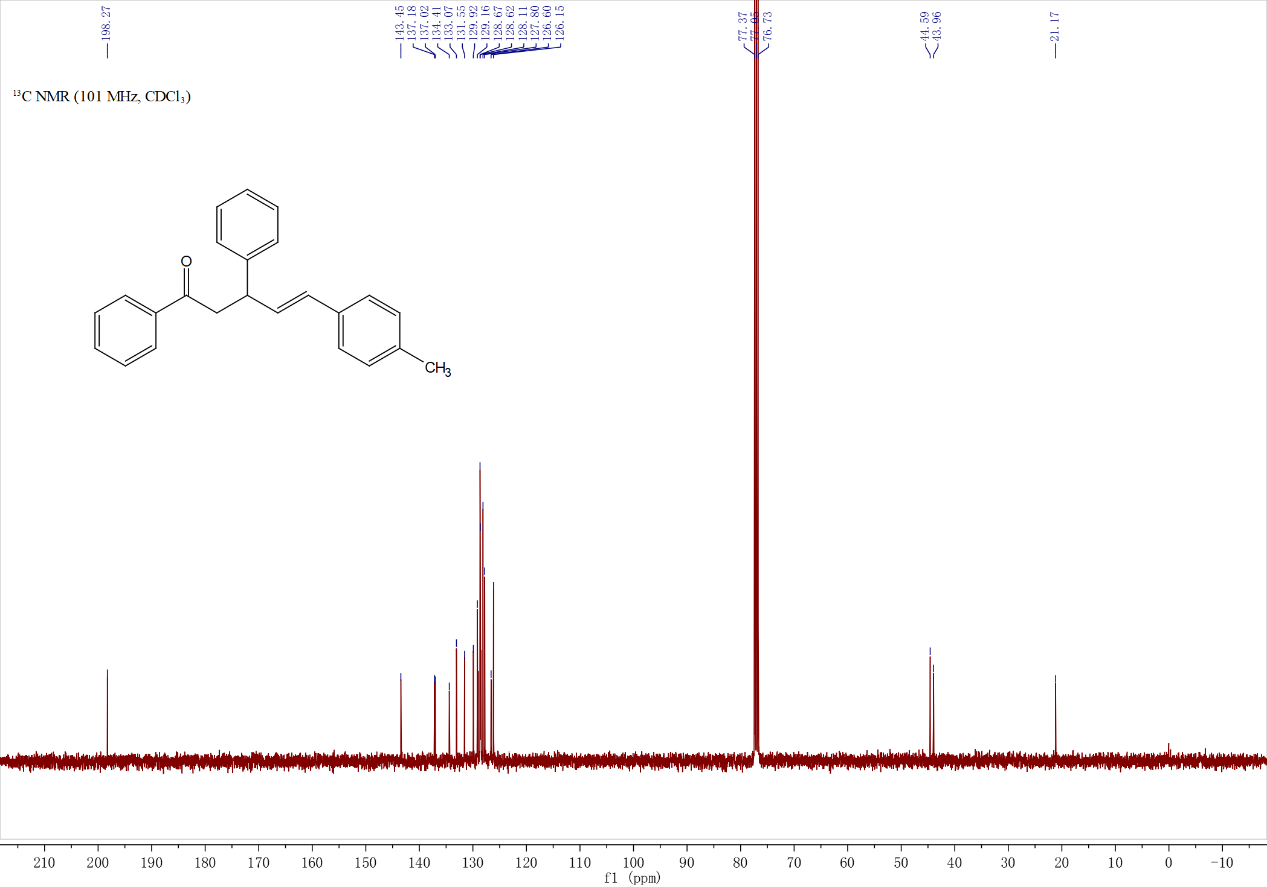


**(E)-3-isopropyl-5-(4-methoxyphenyl)-1-phenylpent-4-en-1-one (3w)**


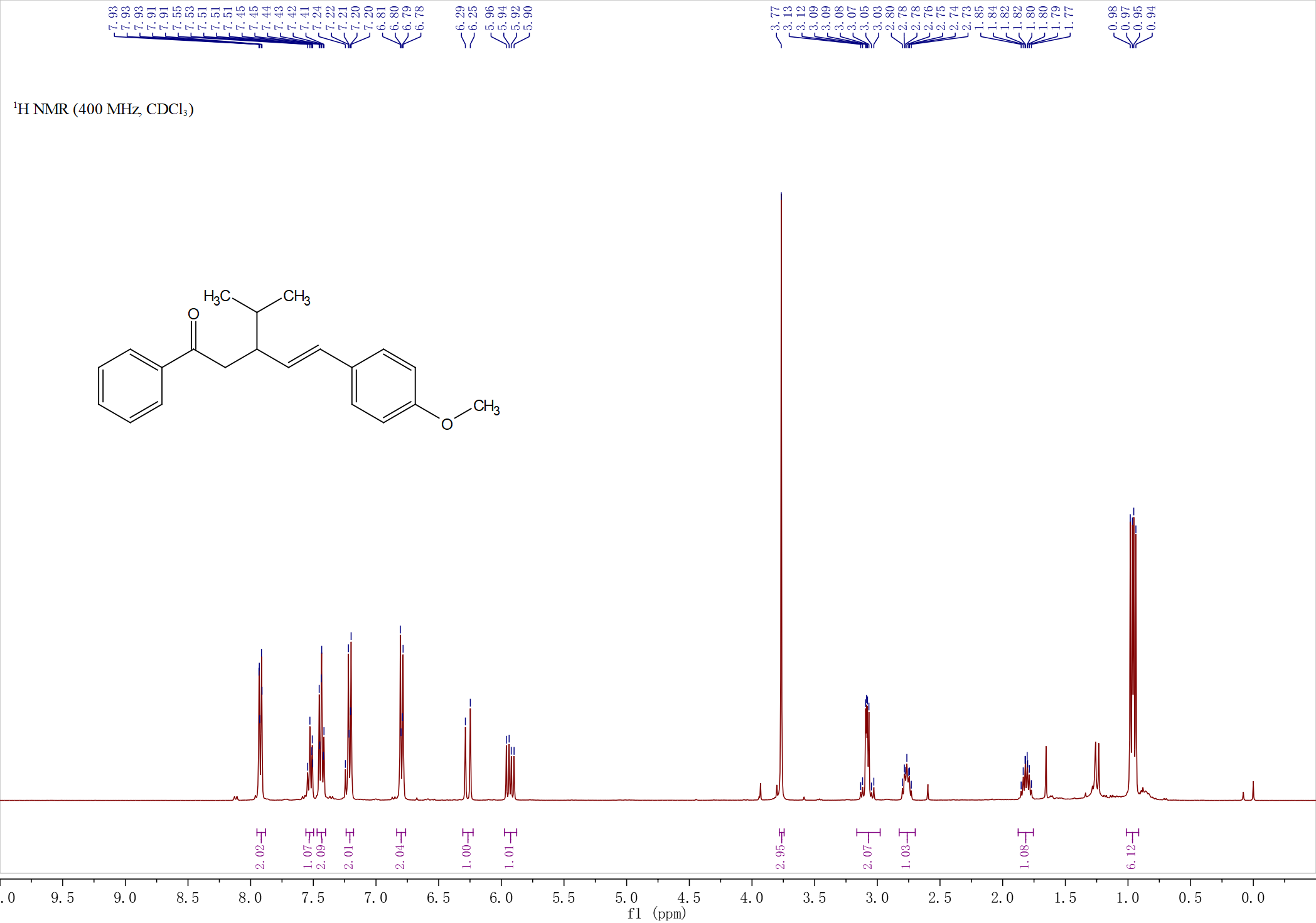


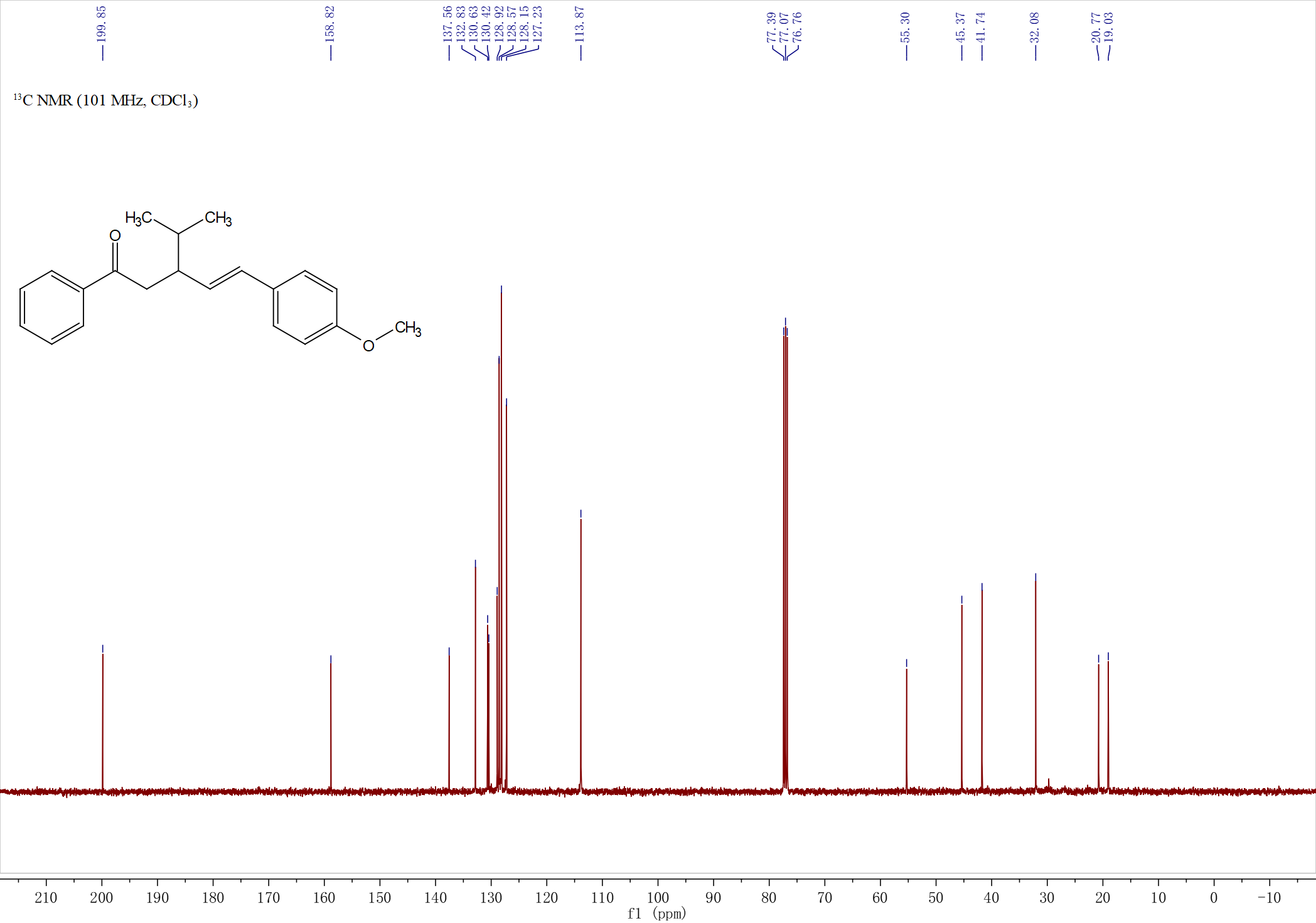


***tert*-butyl (R,E)-4-(1-(4-methoxyphenyl)-5-oxo-5-phenylpent-1-en-3-yl)piperidine-1-carboxylate (3x)**


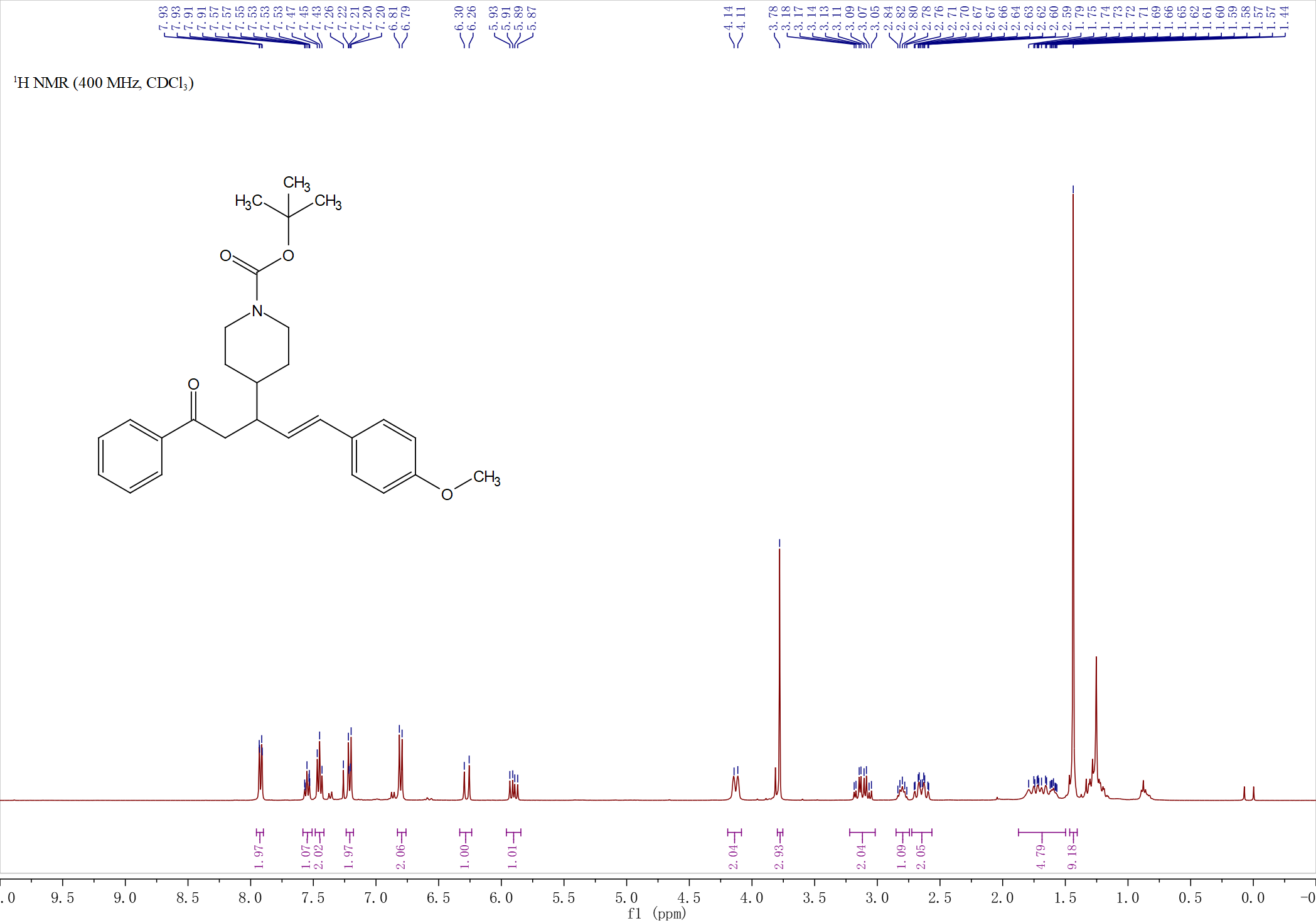


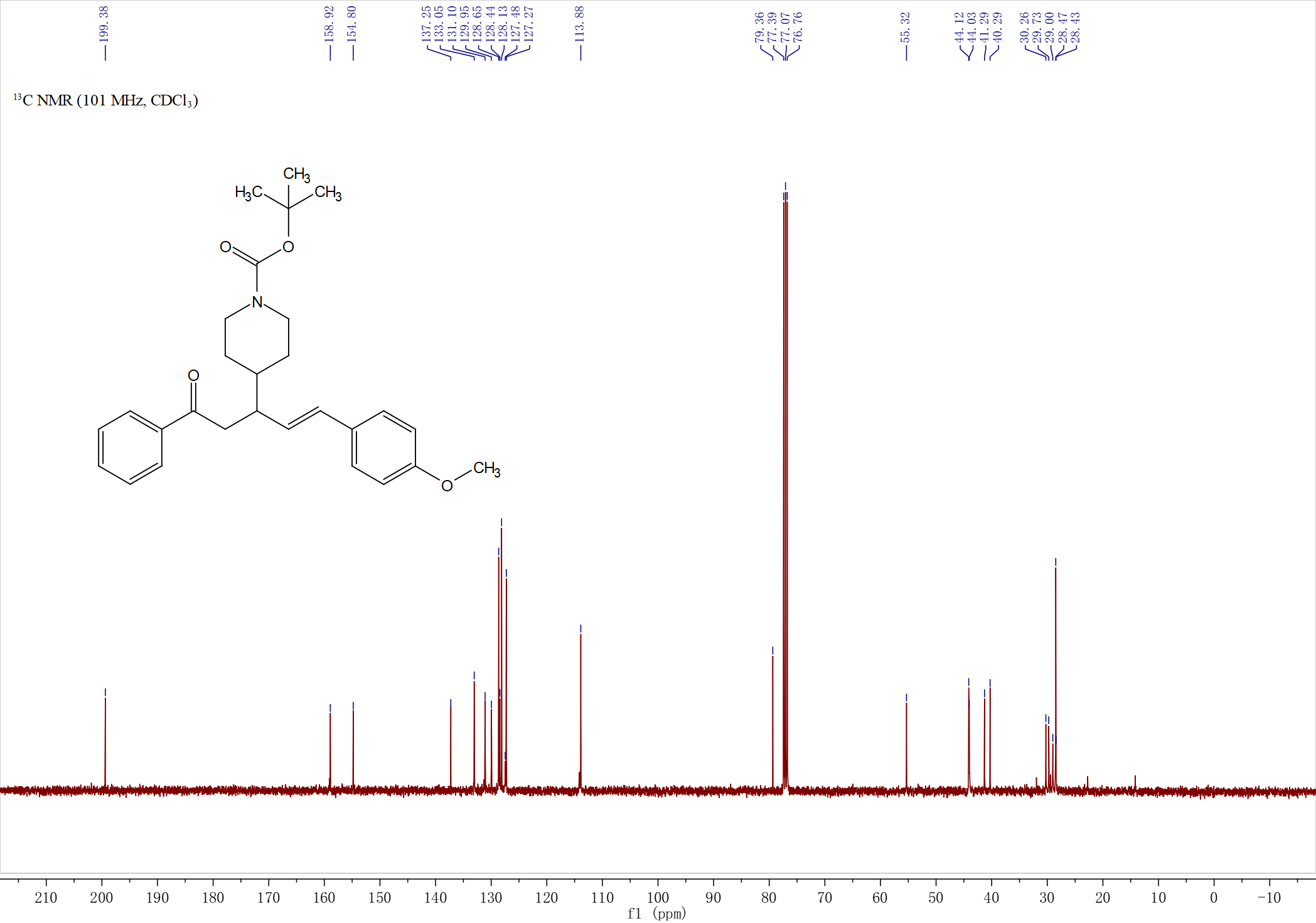


***tert*-butyl (E)-4-(1-(2,3-dihydrobenzofuran-5-yl)-5-oxo-5-phenylpent-1-en-3-yl)piperidine-1-carboxylate (3y):**


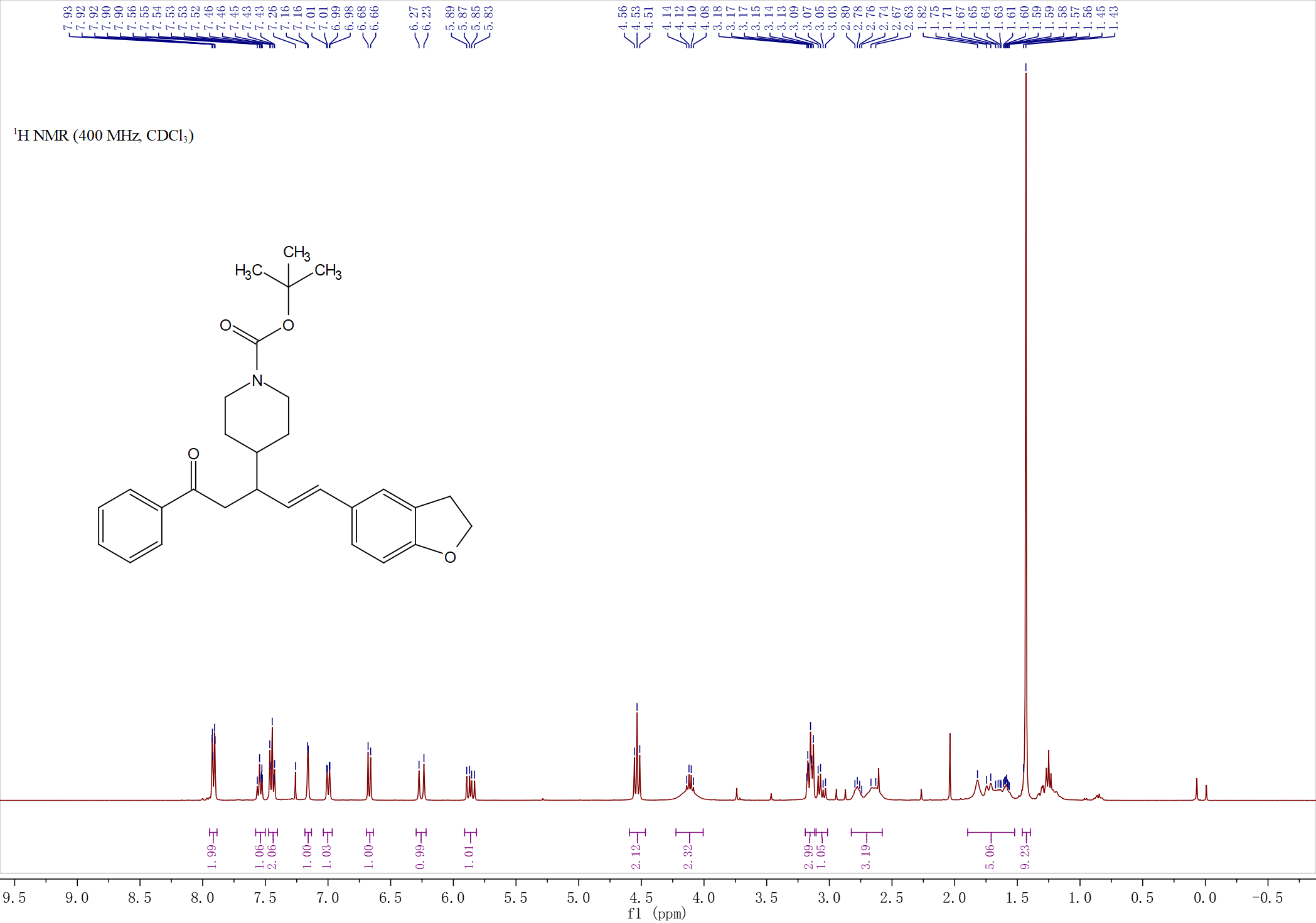


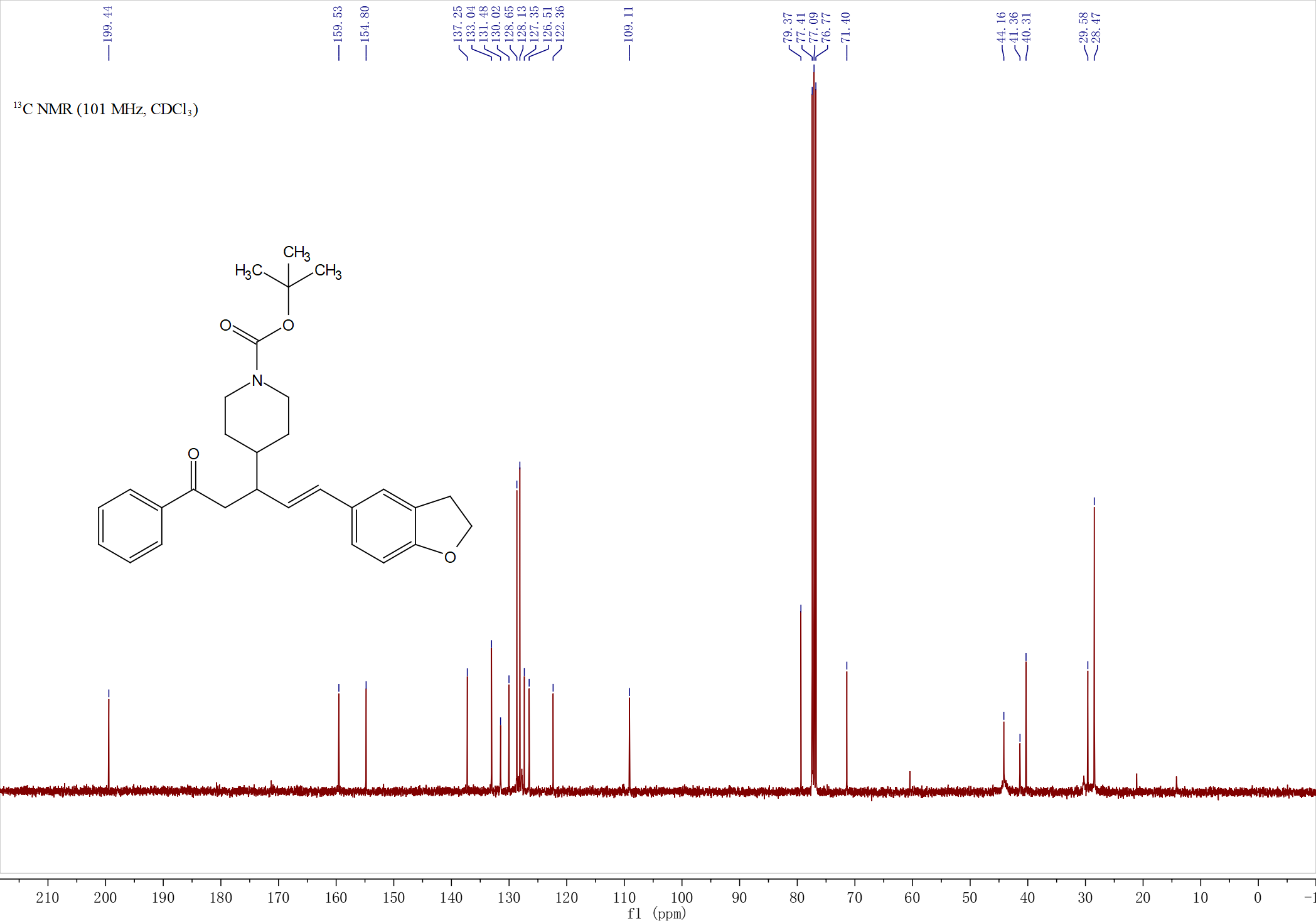


**4-methyl-1,3-diphenylpent-4-en-1-one (3z):**


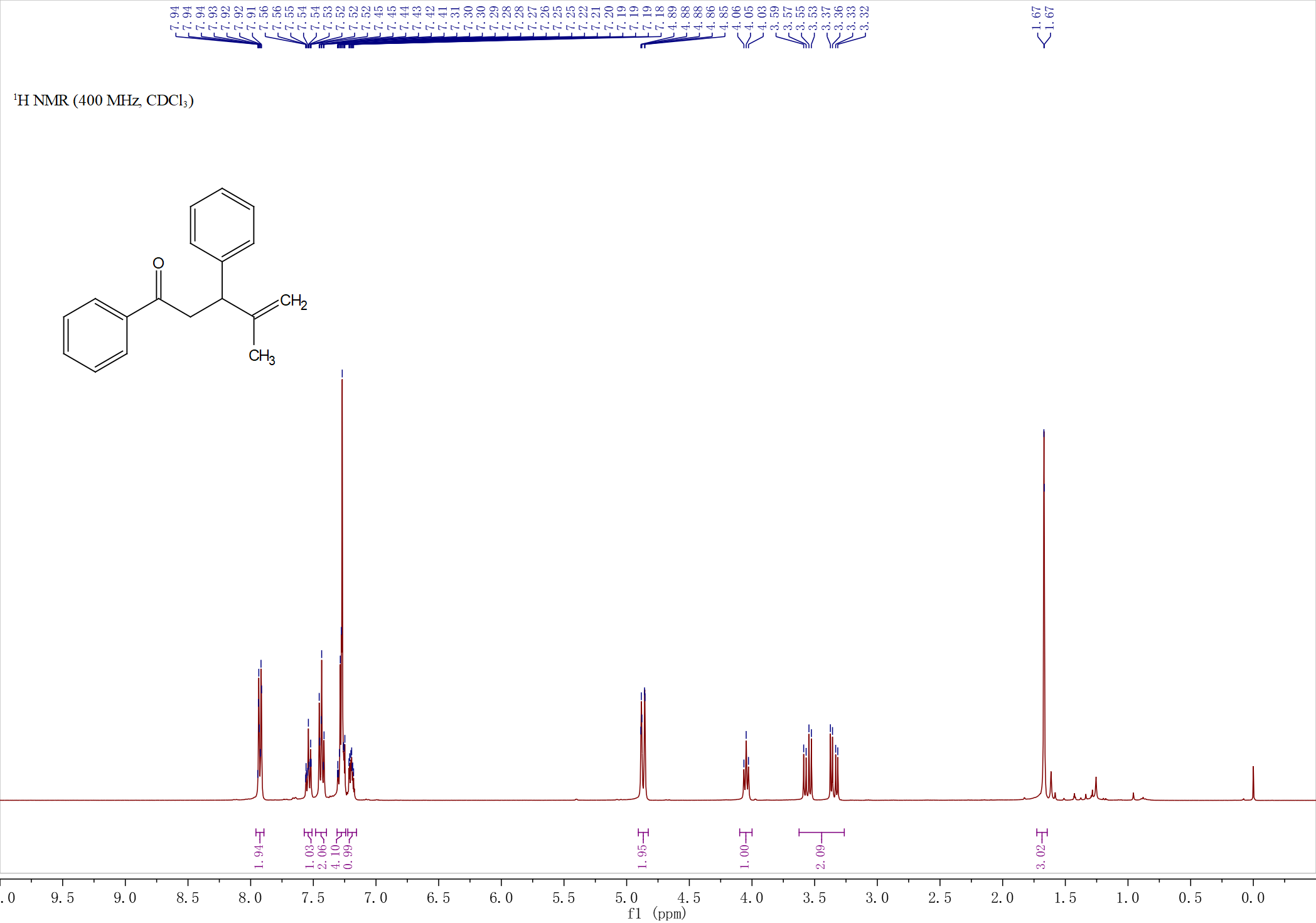


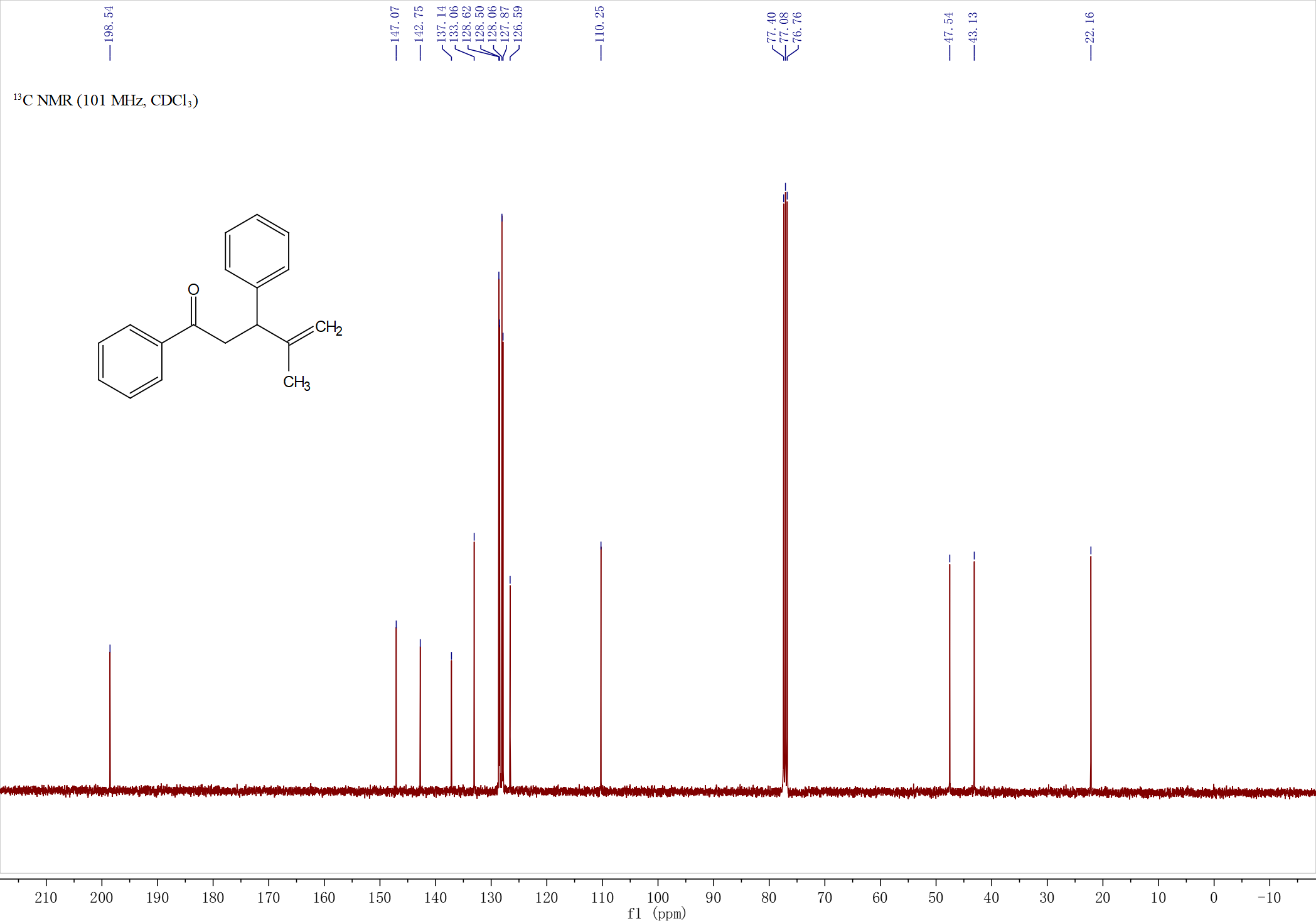


**3-(4-acetylphenyl)-1-(4-chlorophenyl)-3-phenylpropan-1-one (4a)**


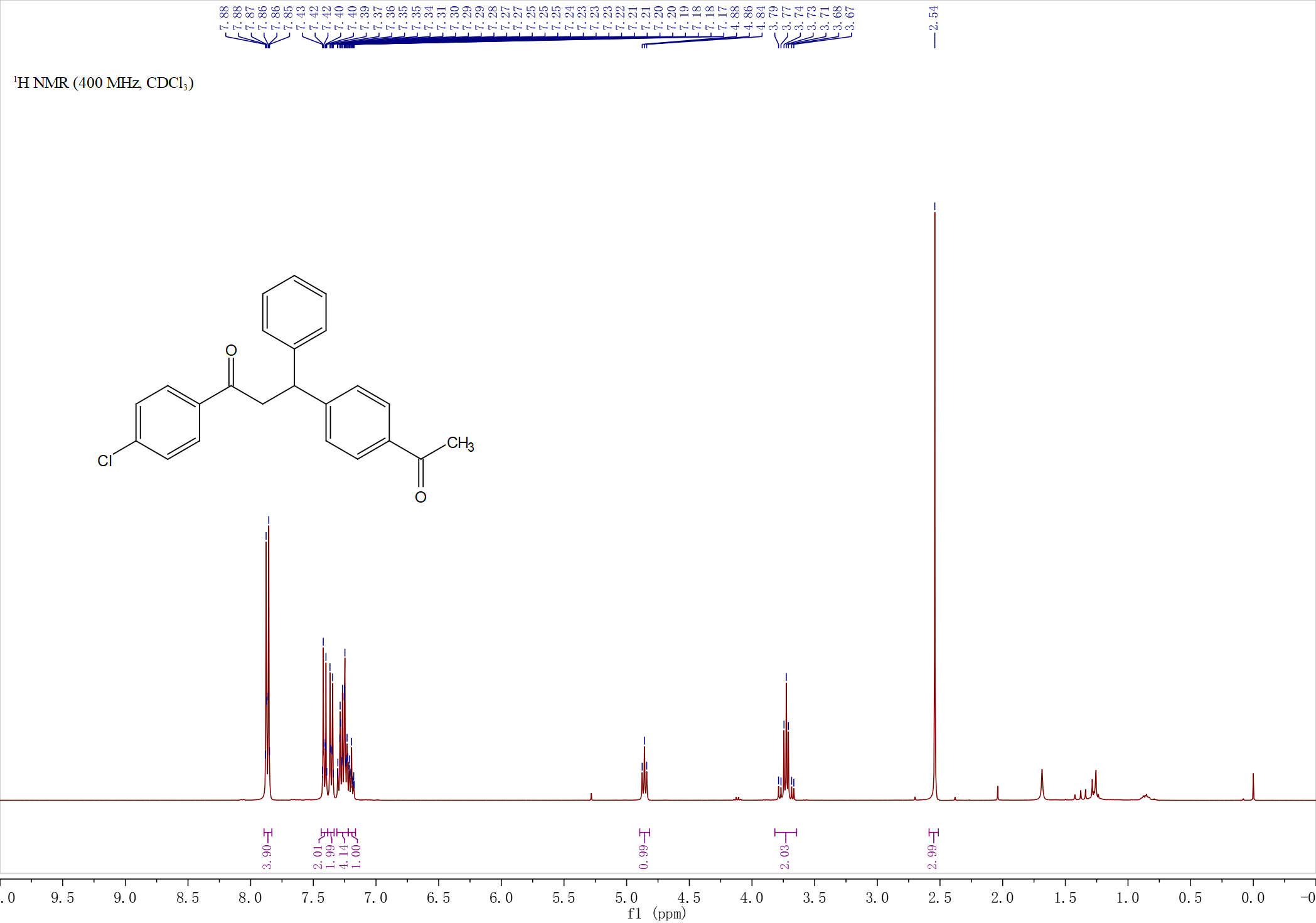


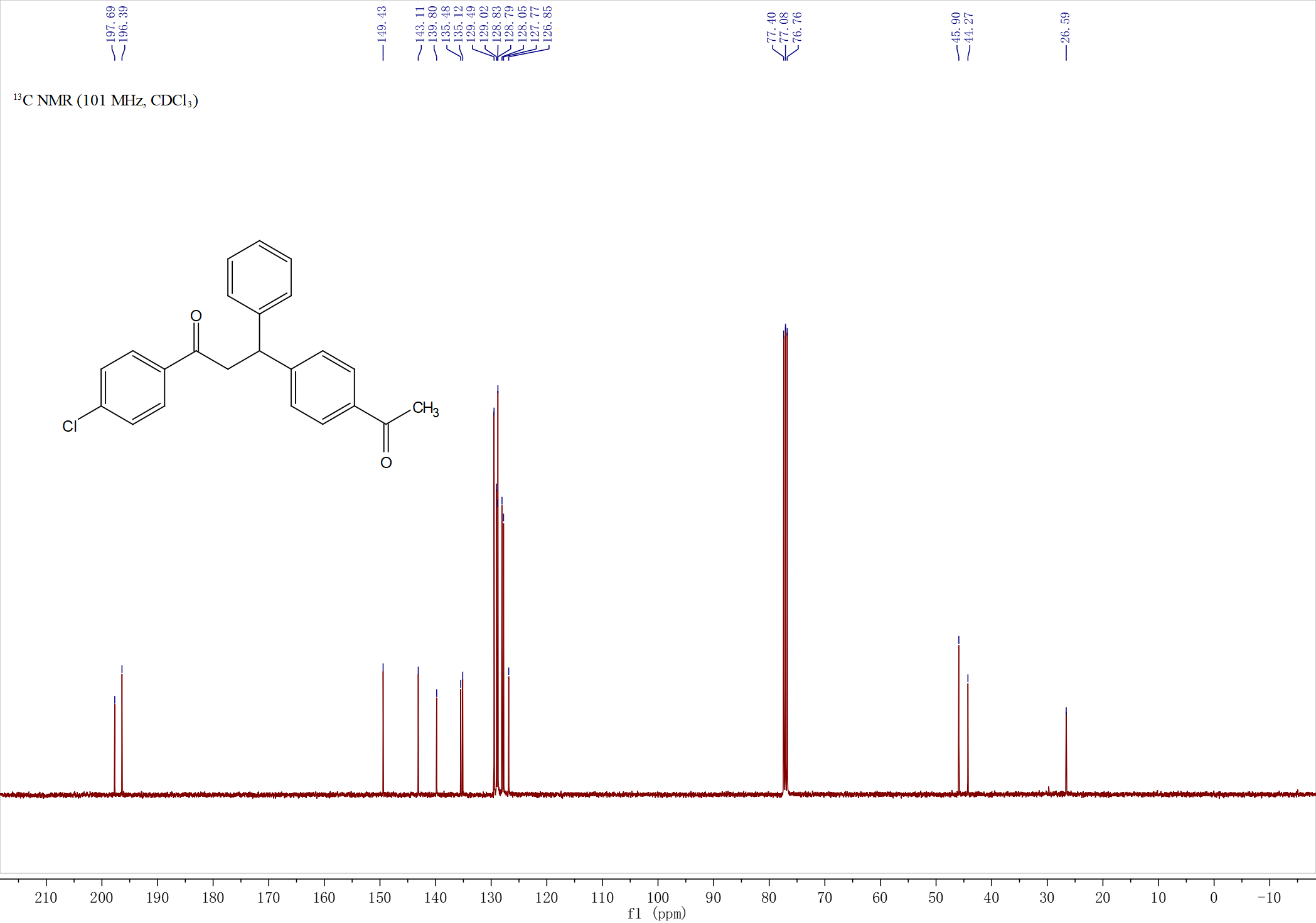


**3-(4-acetylphenyl)-1-(4-fluorophenyl)-3-phenylpropan-1-one (4b)**


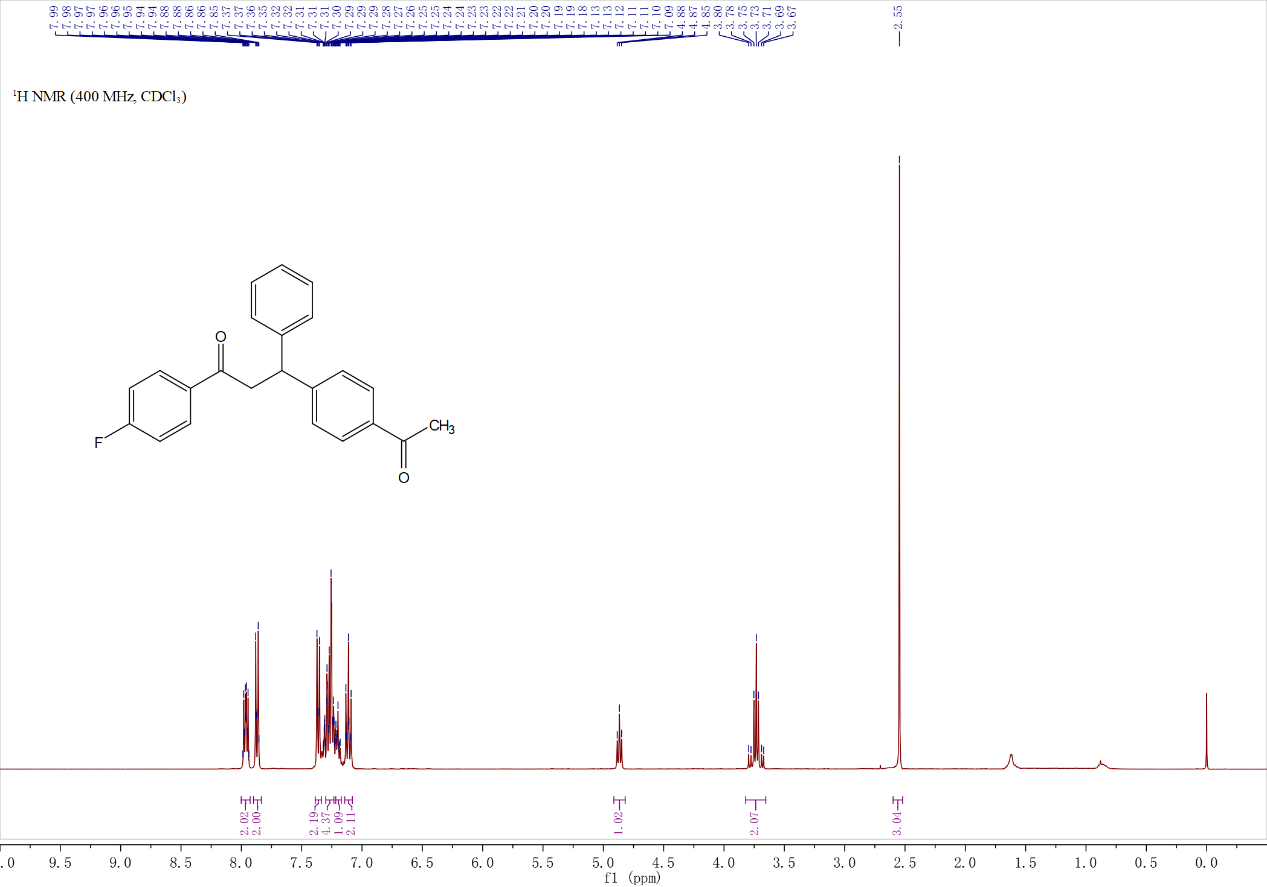


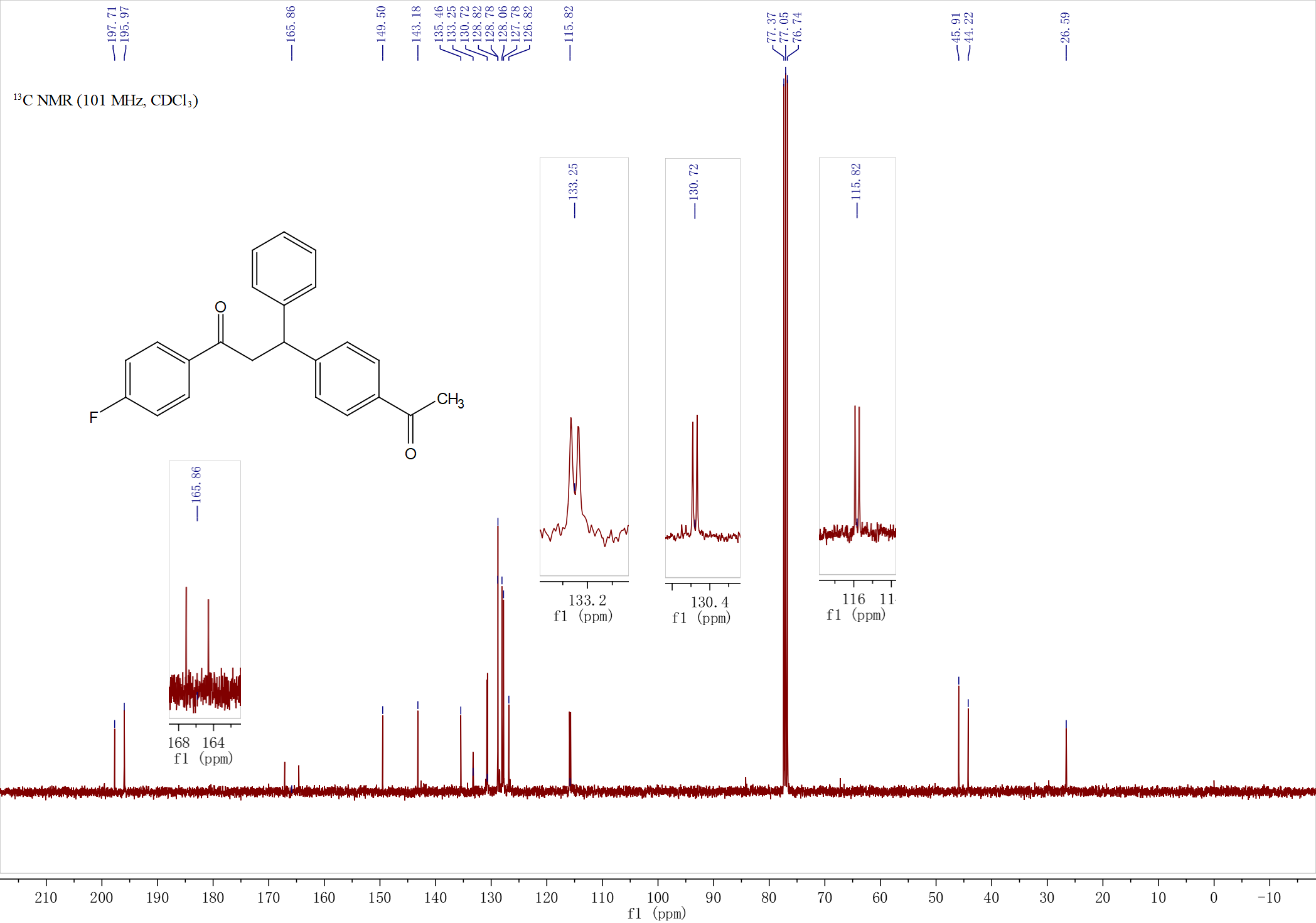


**3-(4-acetylphenyl)-1-(naphthalen-2-yl)-3-phenylpropan-1-one (4c)**


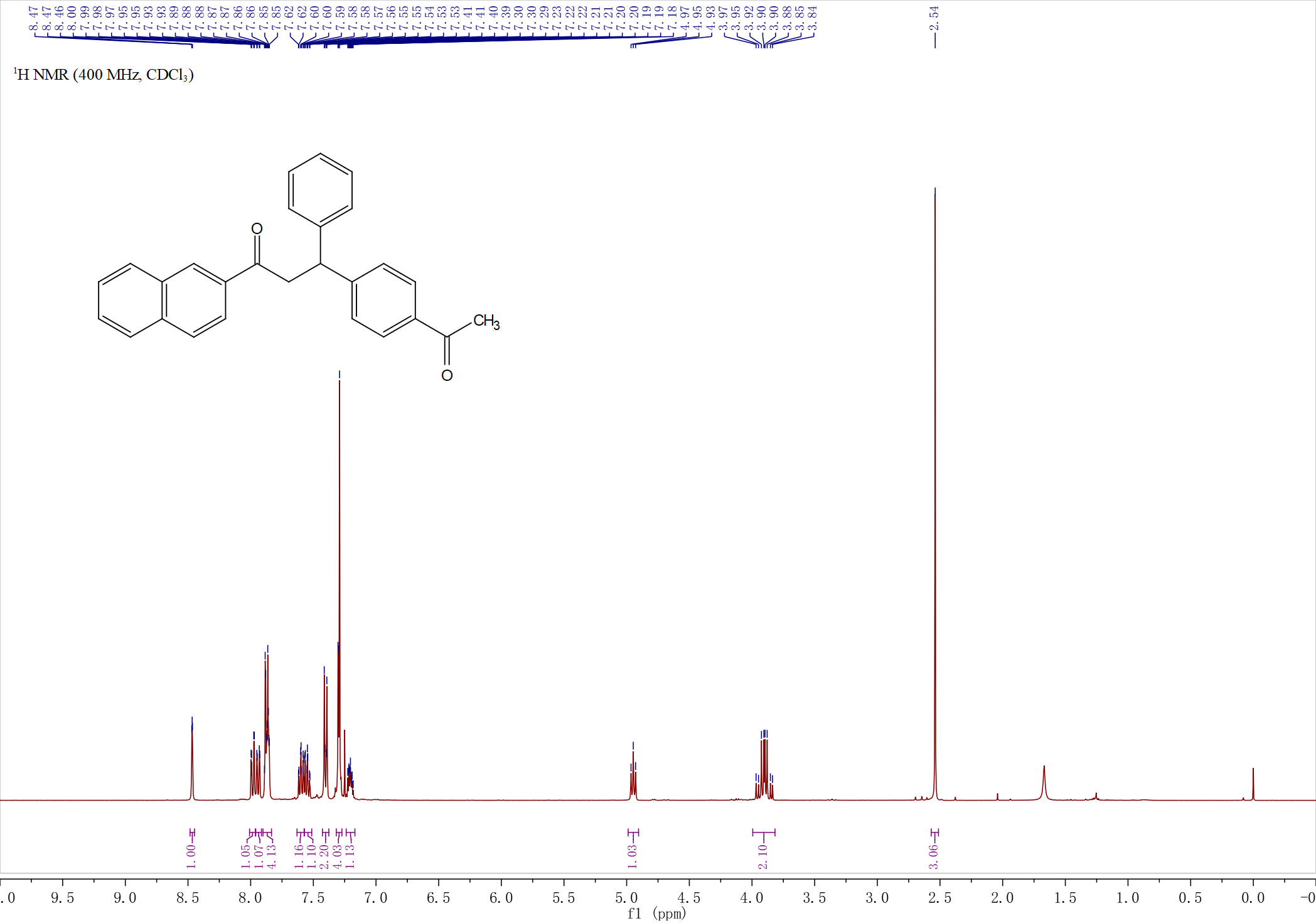


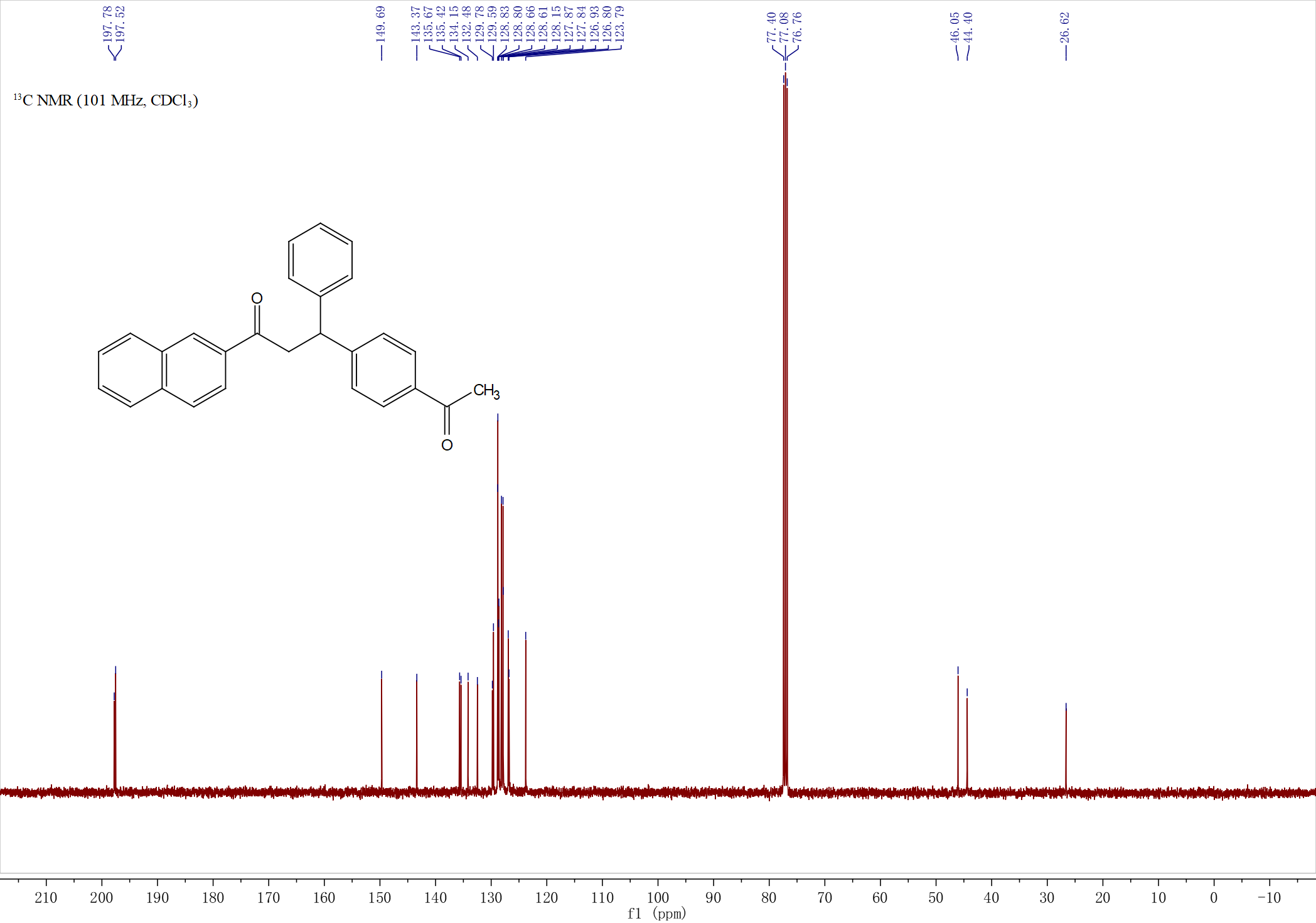


**3-(4-acetylphenyl)-3-phenyl-1-(pyridin-3-yl) propan-1-one (4d)**


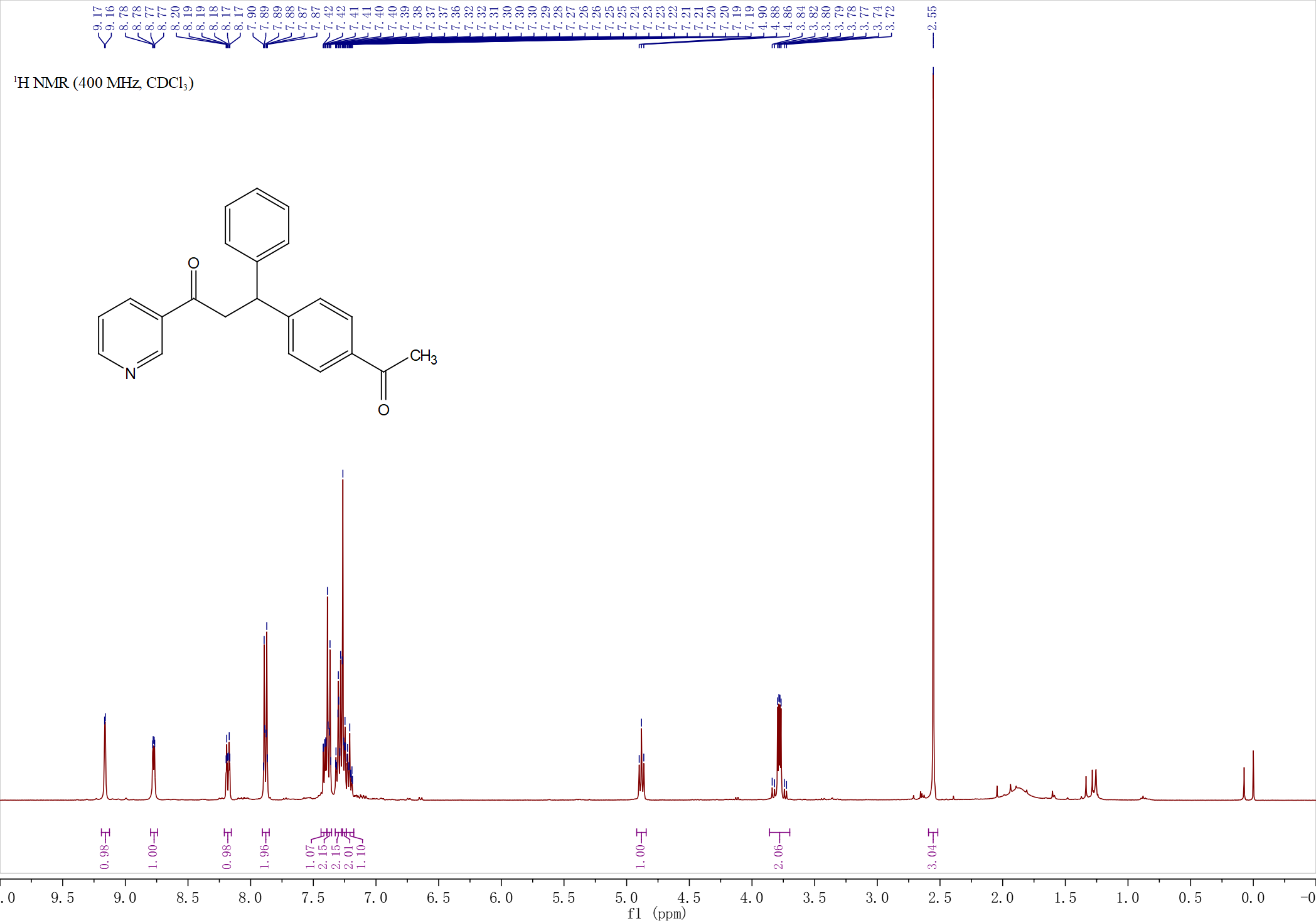


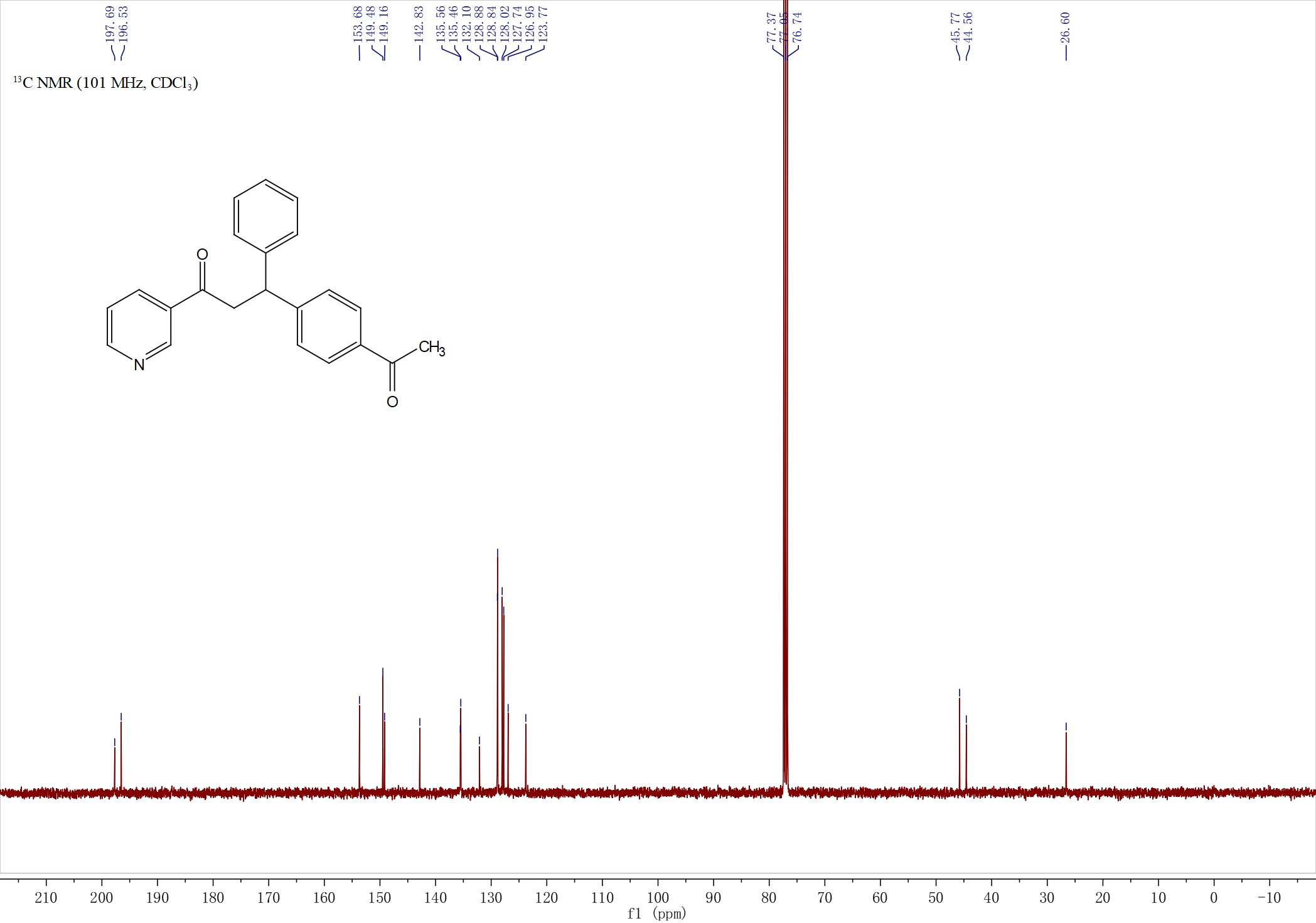


**3-(4-acetylphenyl)-3-(furan-2-yl)-1-phenylpropan-1-one (4e)**


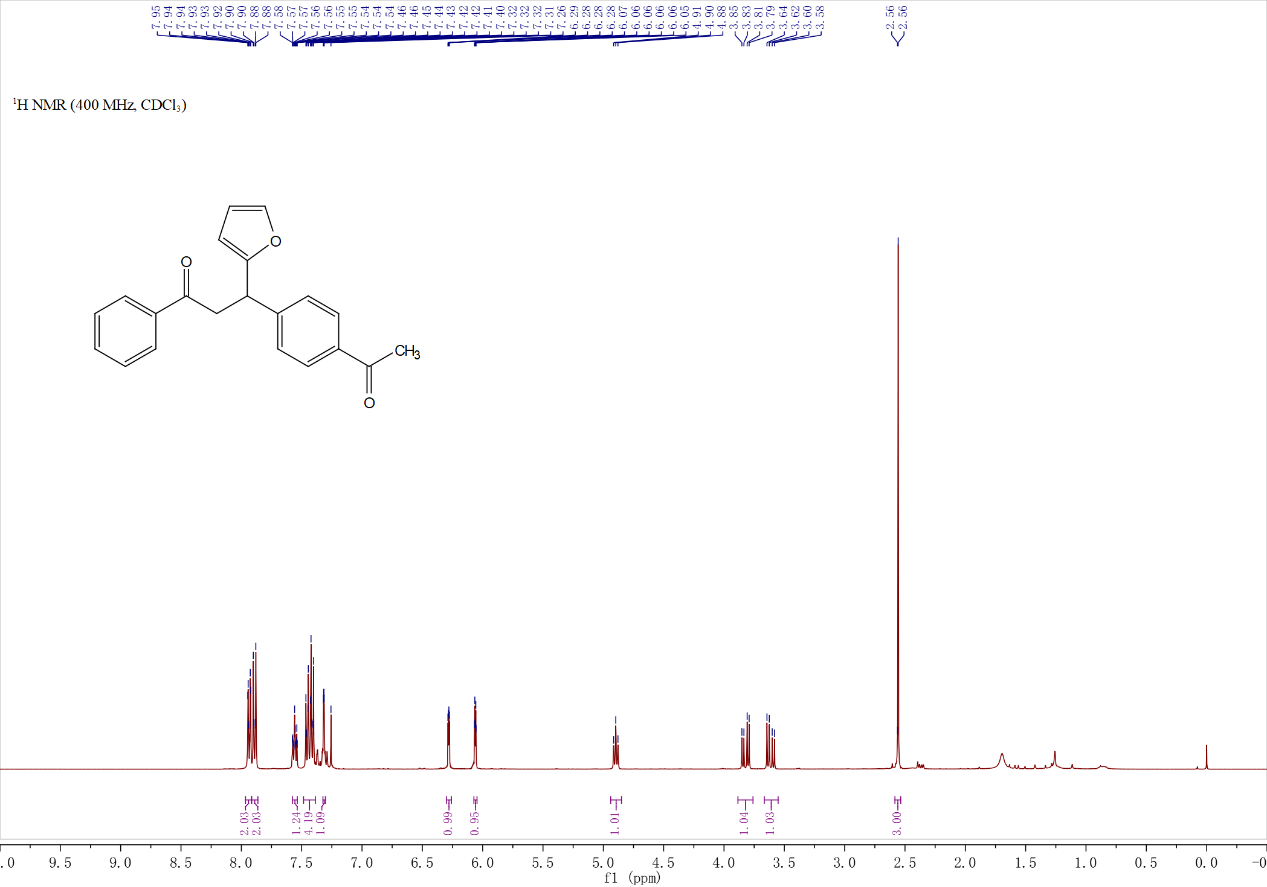


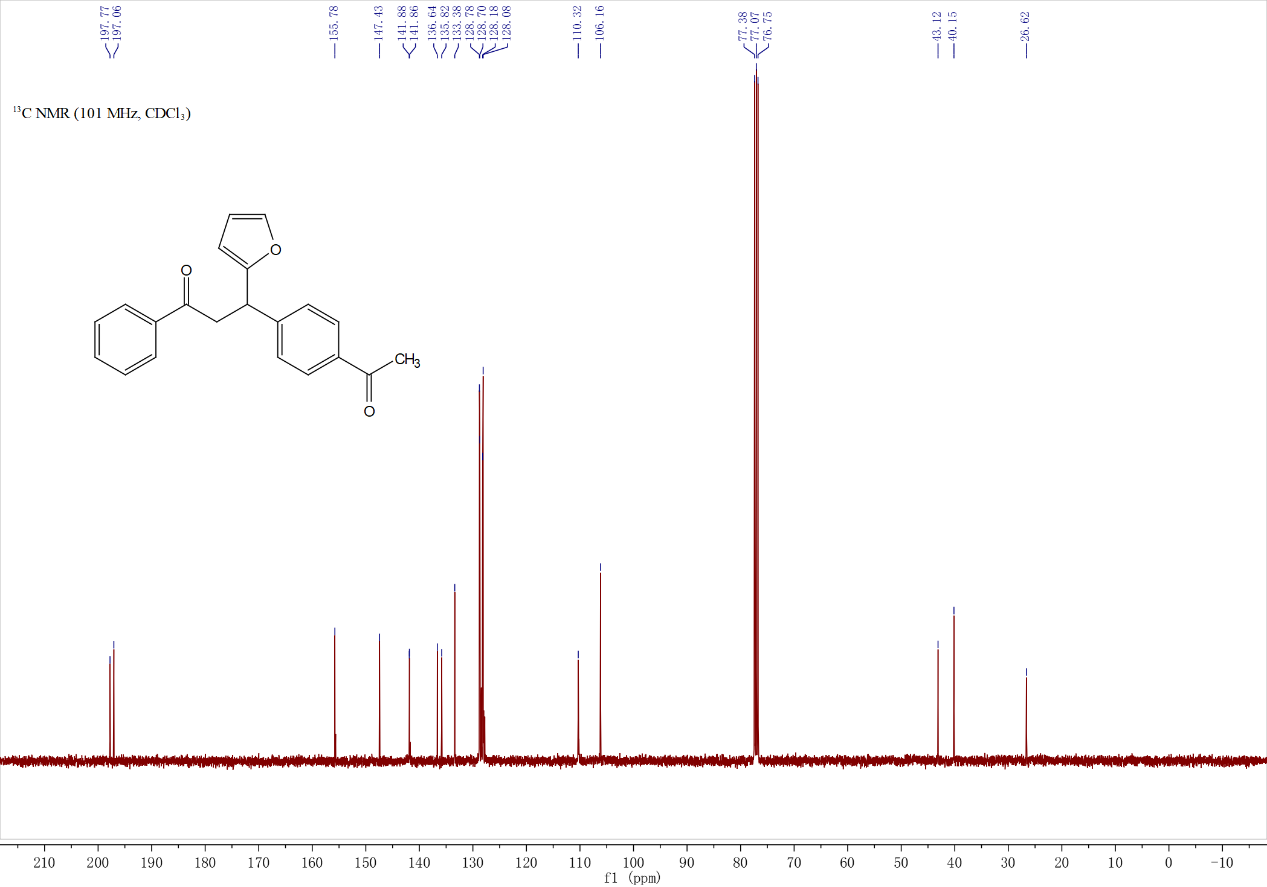


**3-(4-acetylphenyl)-1-phenyl-3-(thiophen-3-yl)propan-1-one (4f)**


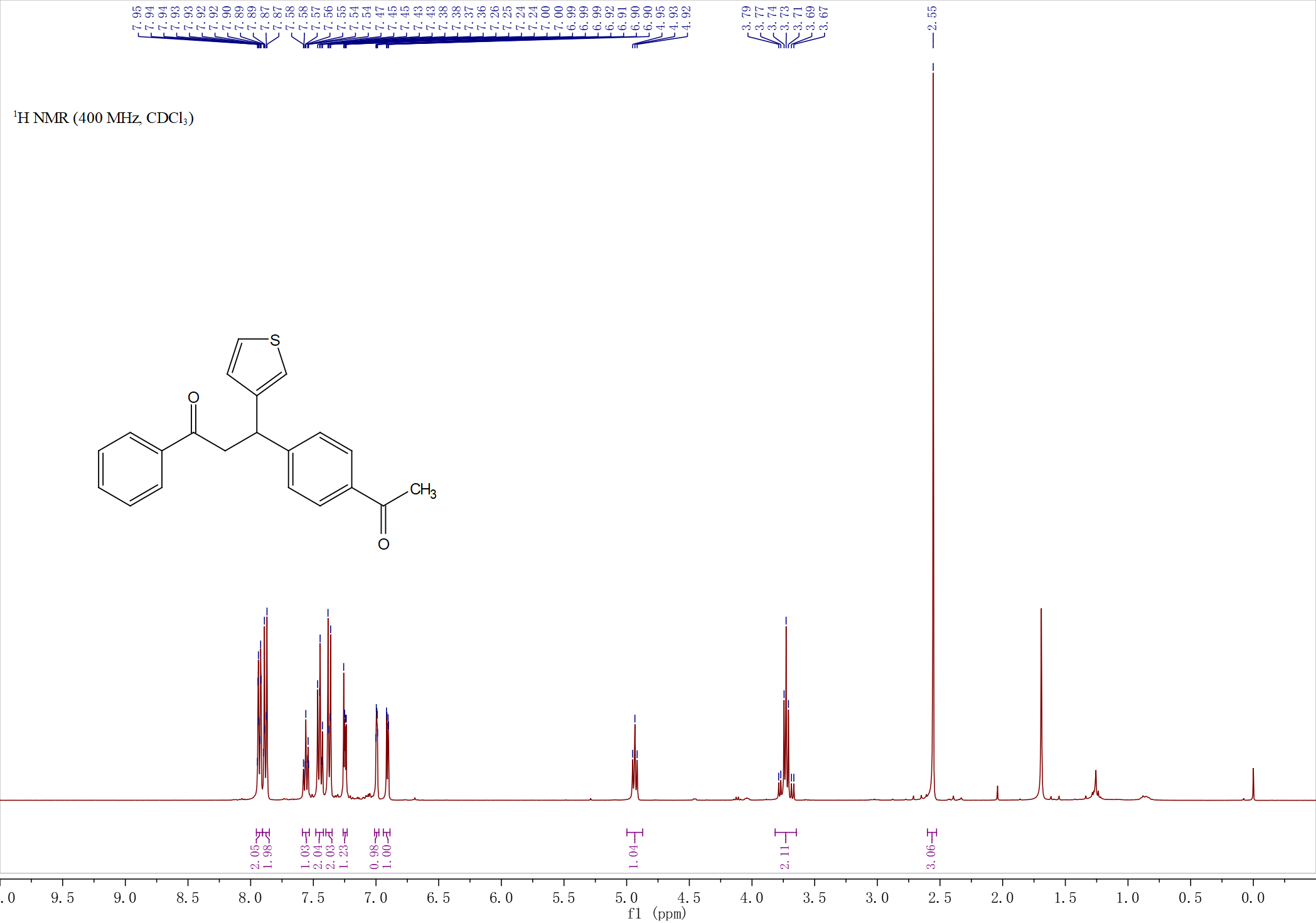


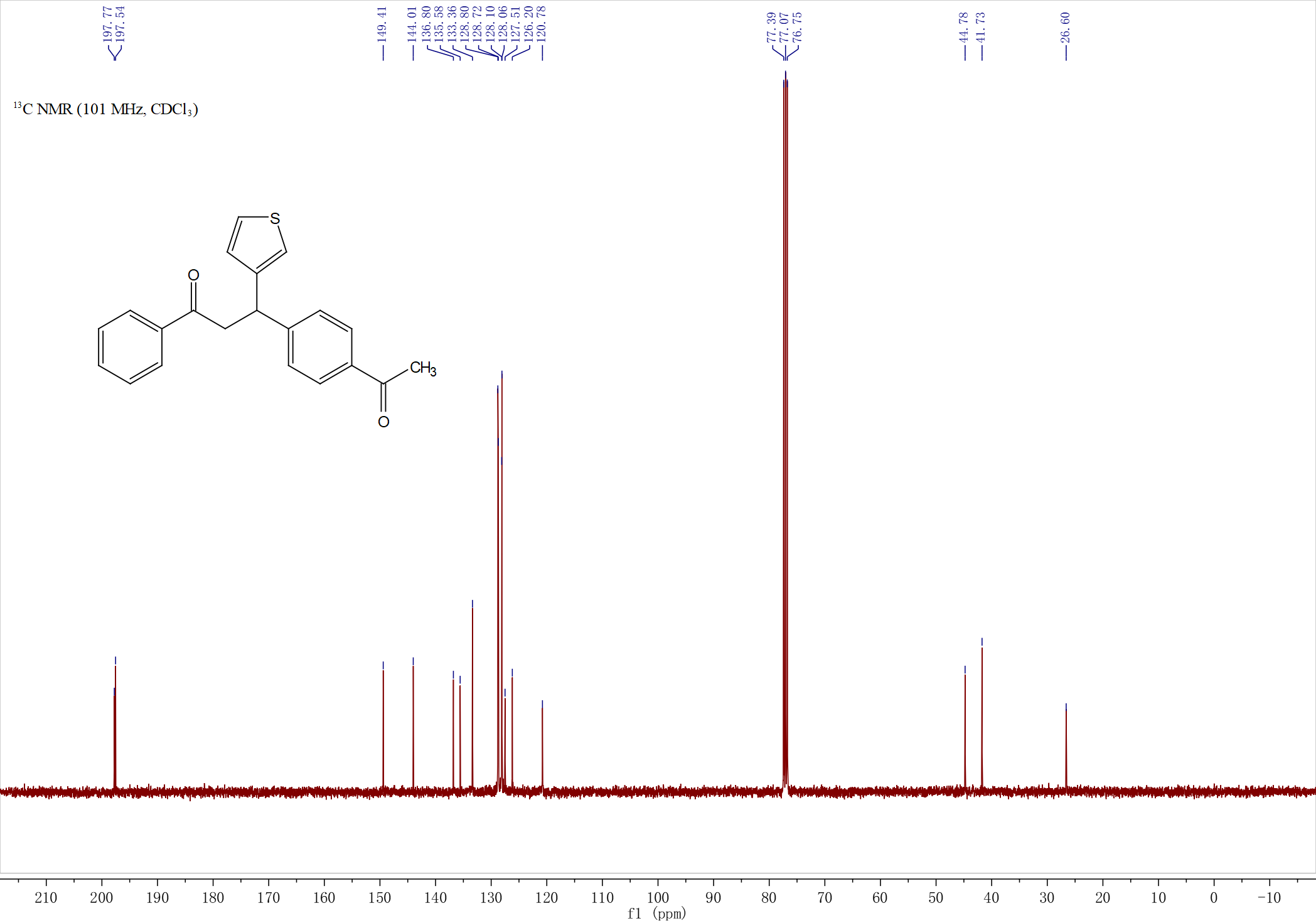


**3-(4-acetylphenyl)-3-(4-methoxyphenyl)-1-phenyl propan-1-one (4g)**


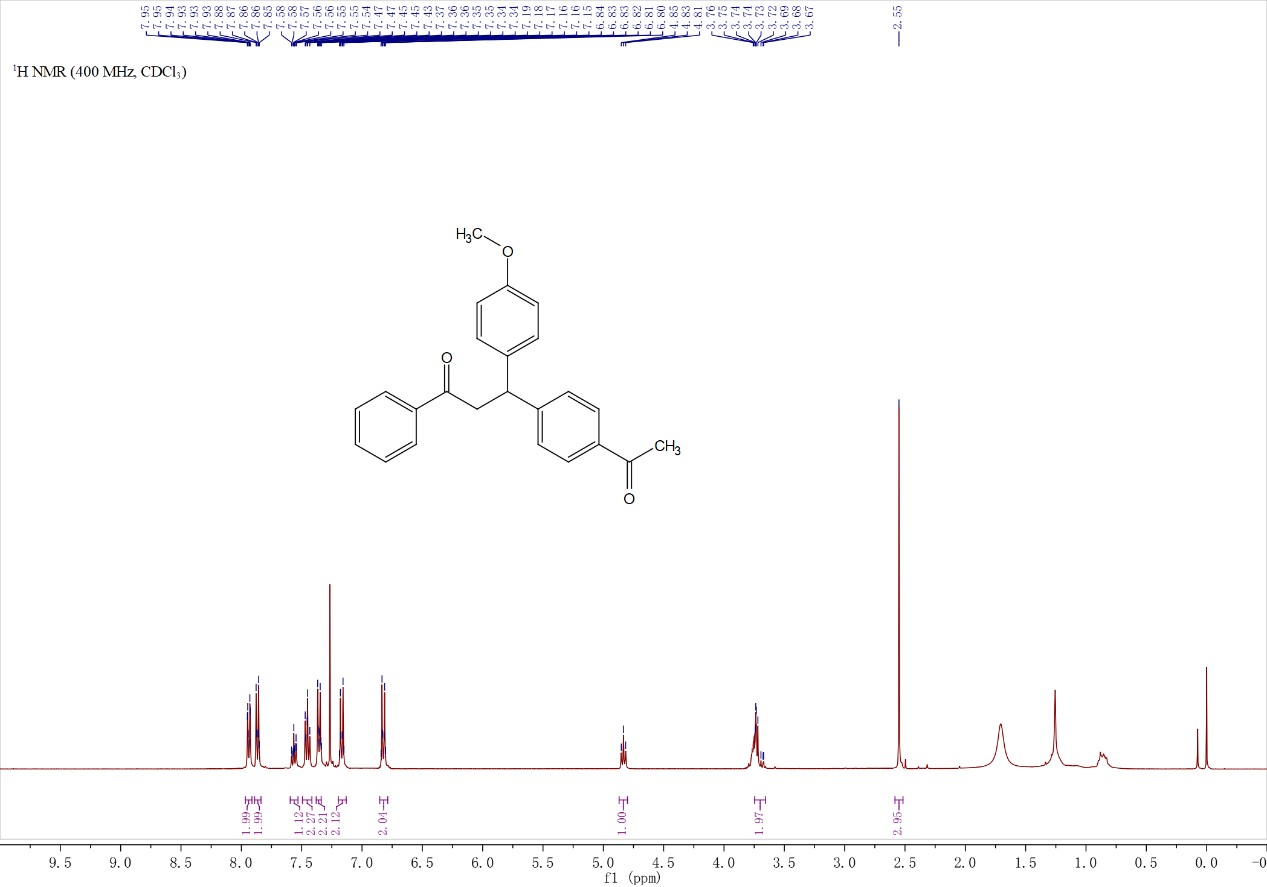


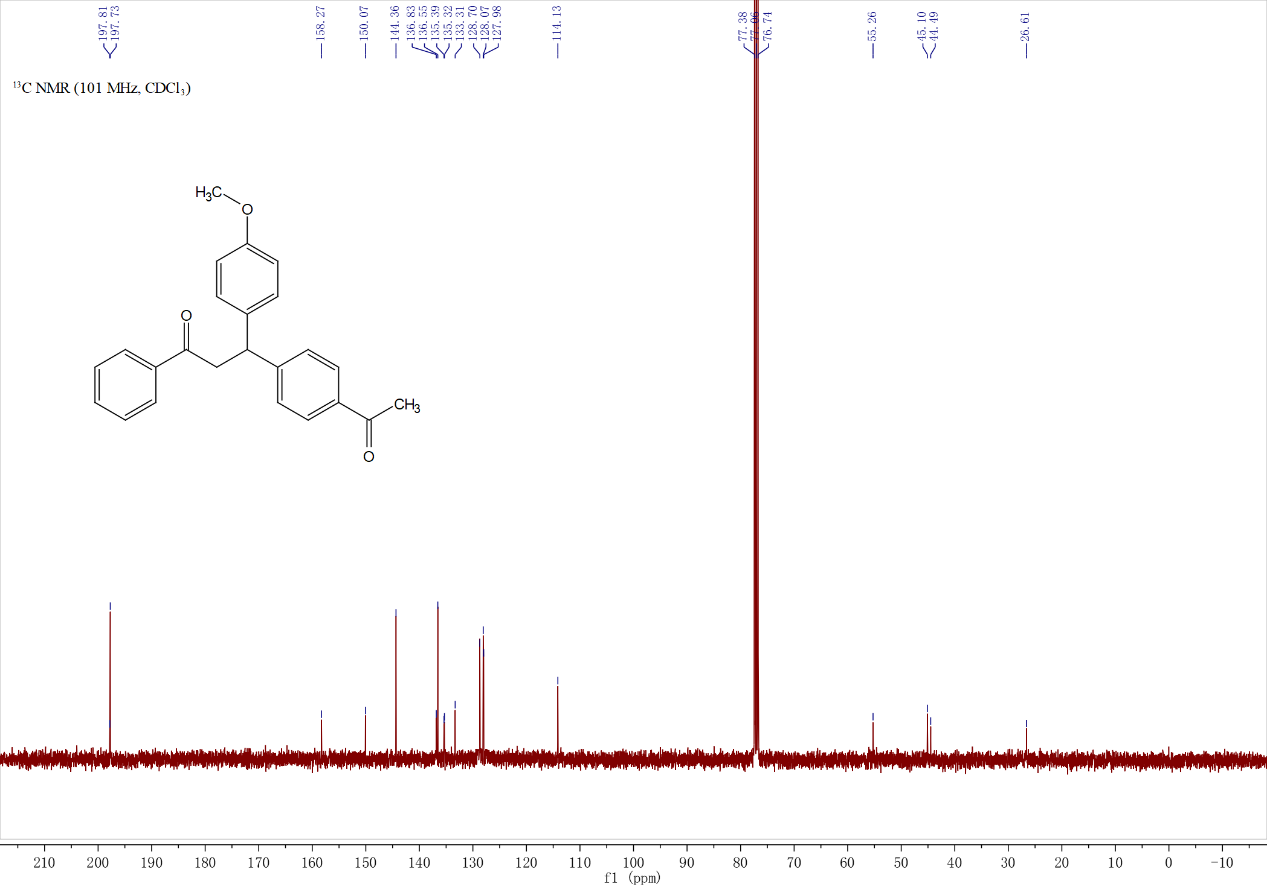


**3-(4-acetylphenyl)-1-phenylpentan-1-one (4h)**


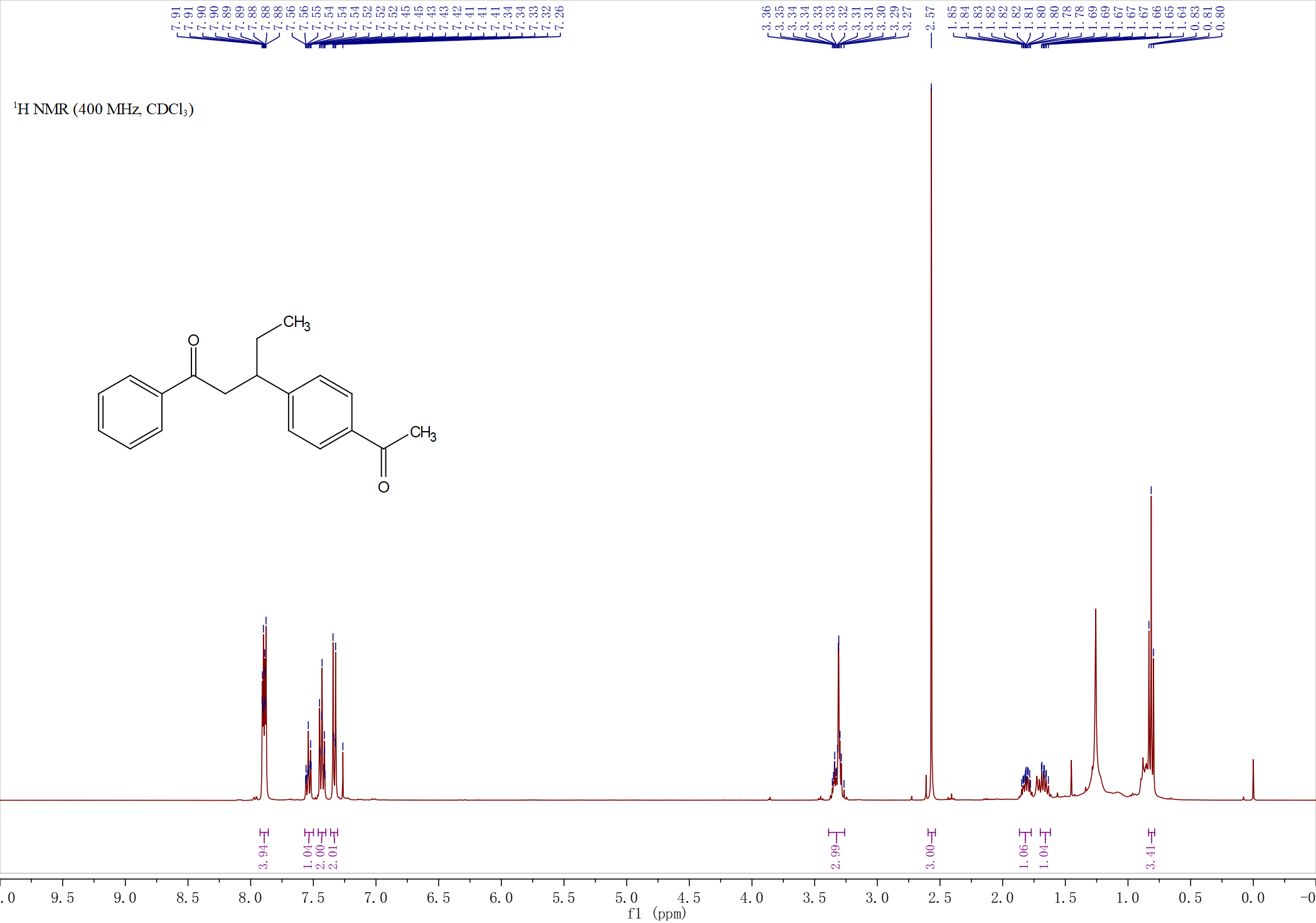


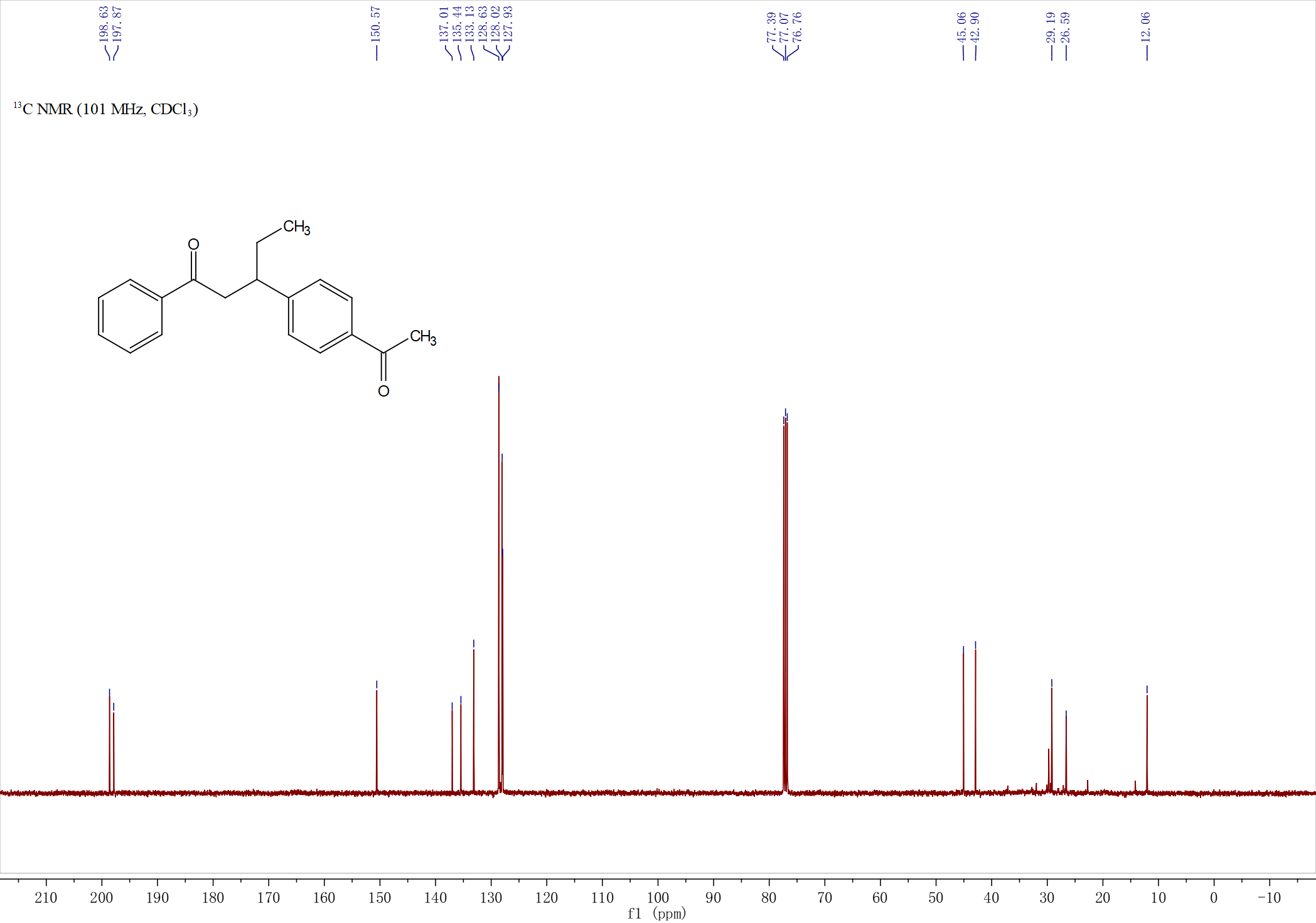


**3-(4-acetylphenyl)-1,5-diphenylpentan-1-one (4i)**


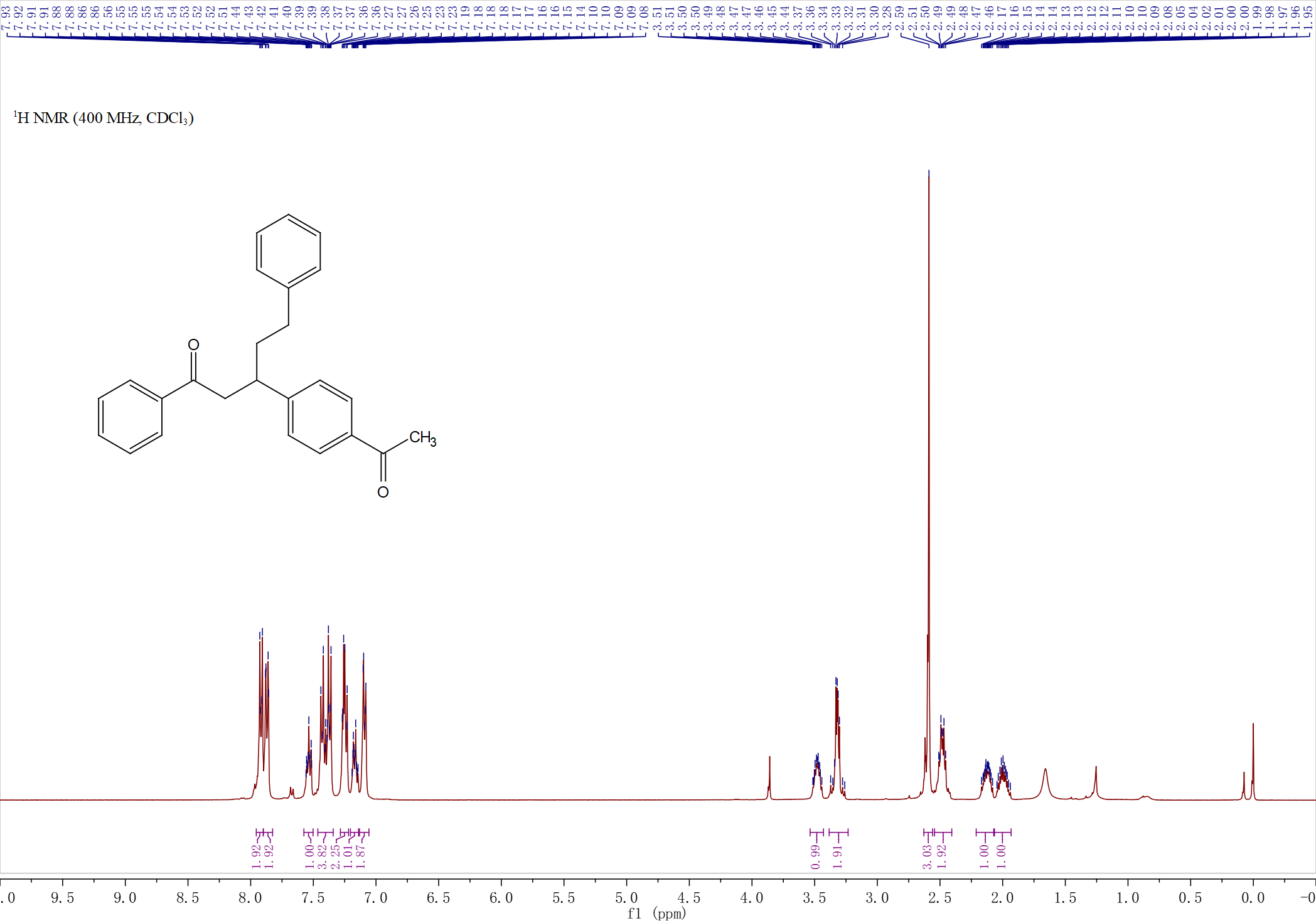


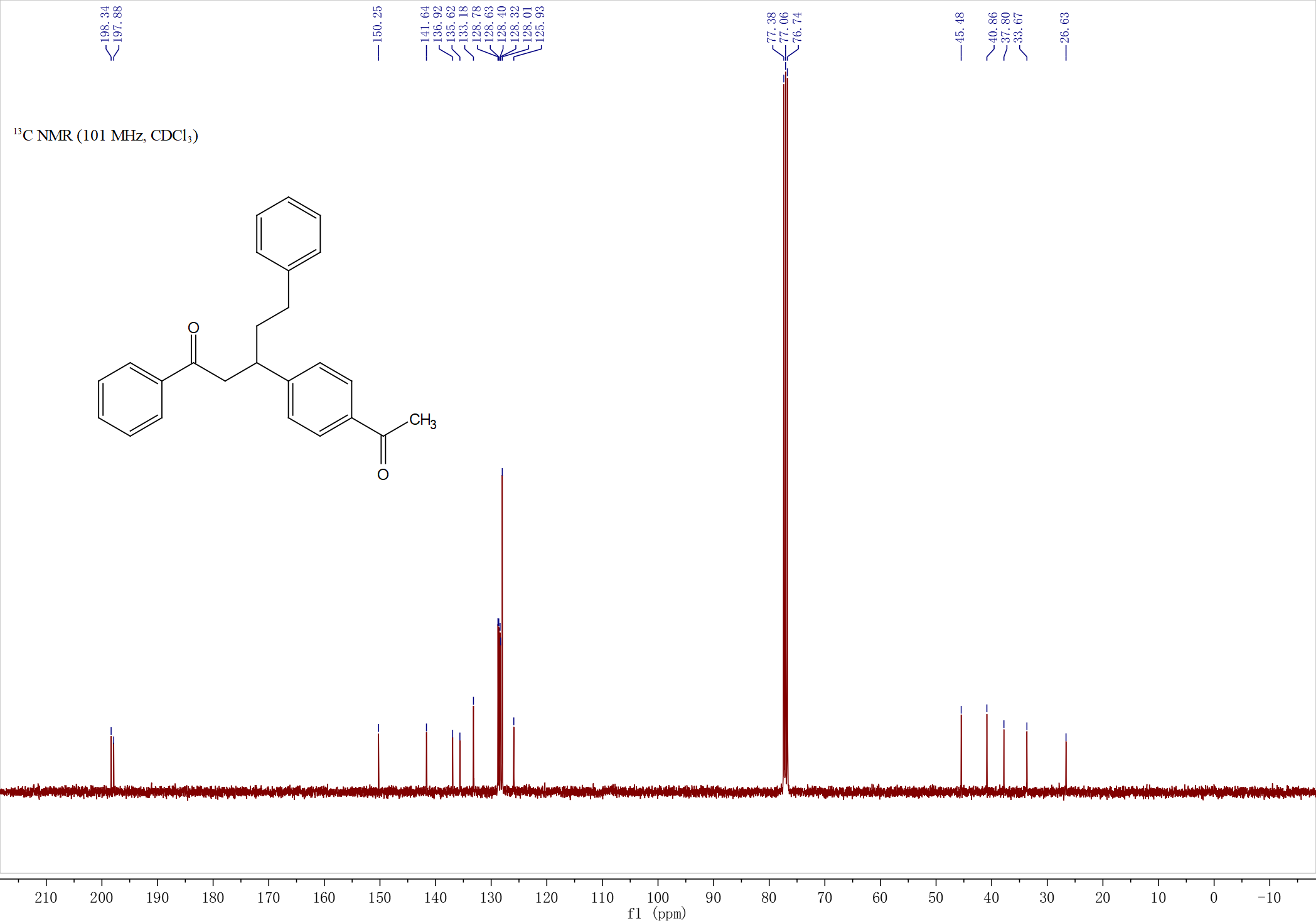


**2-(3-(4-acetylphenyl)-5-oxo-5-phenylpentyl)isoindoline-1,3-dione (4j)**


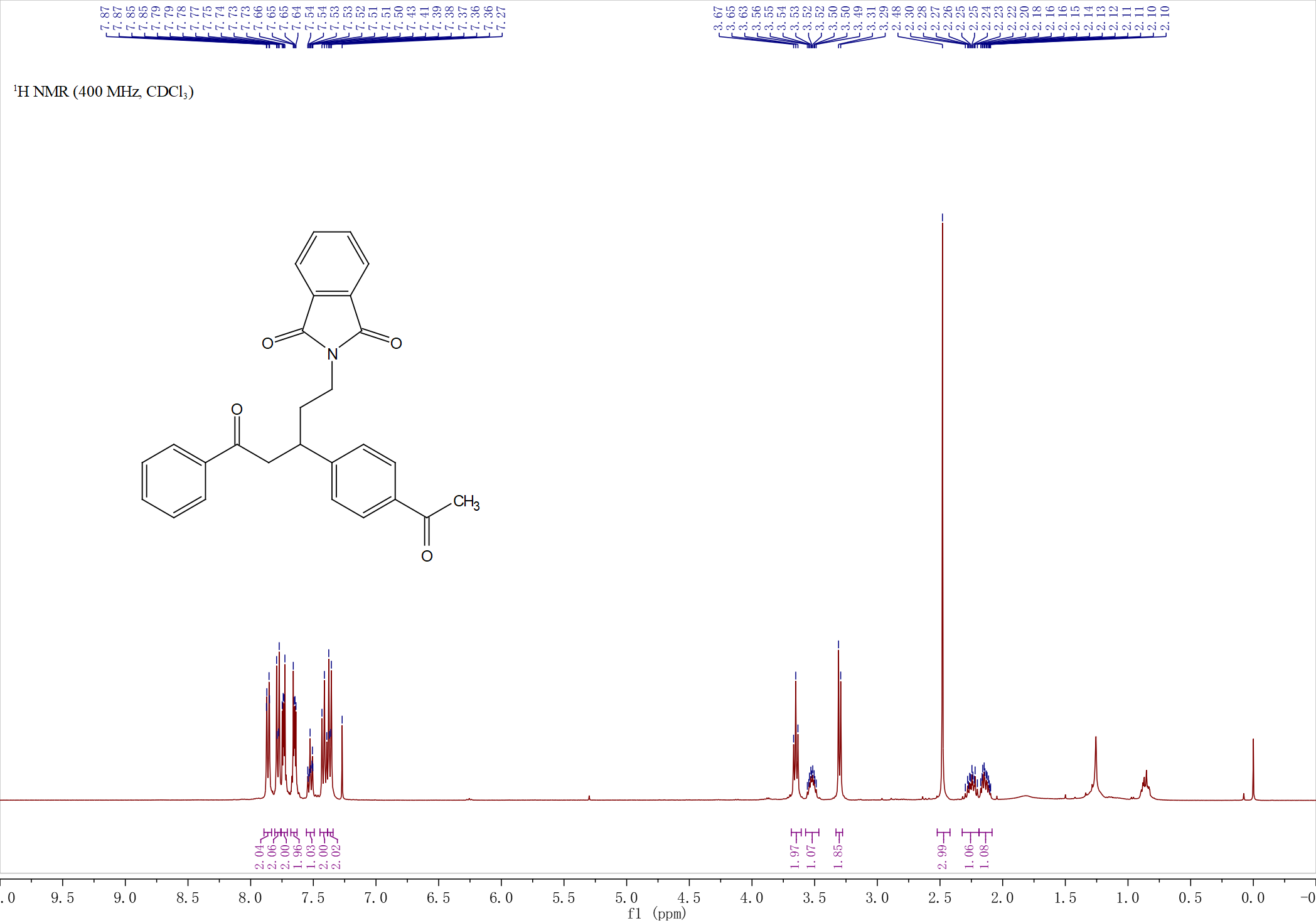


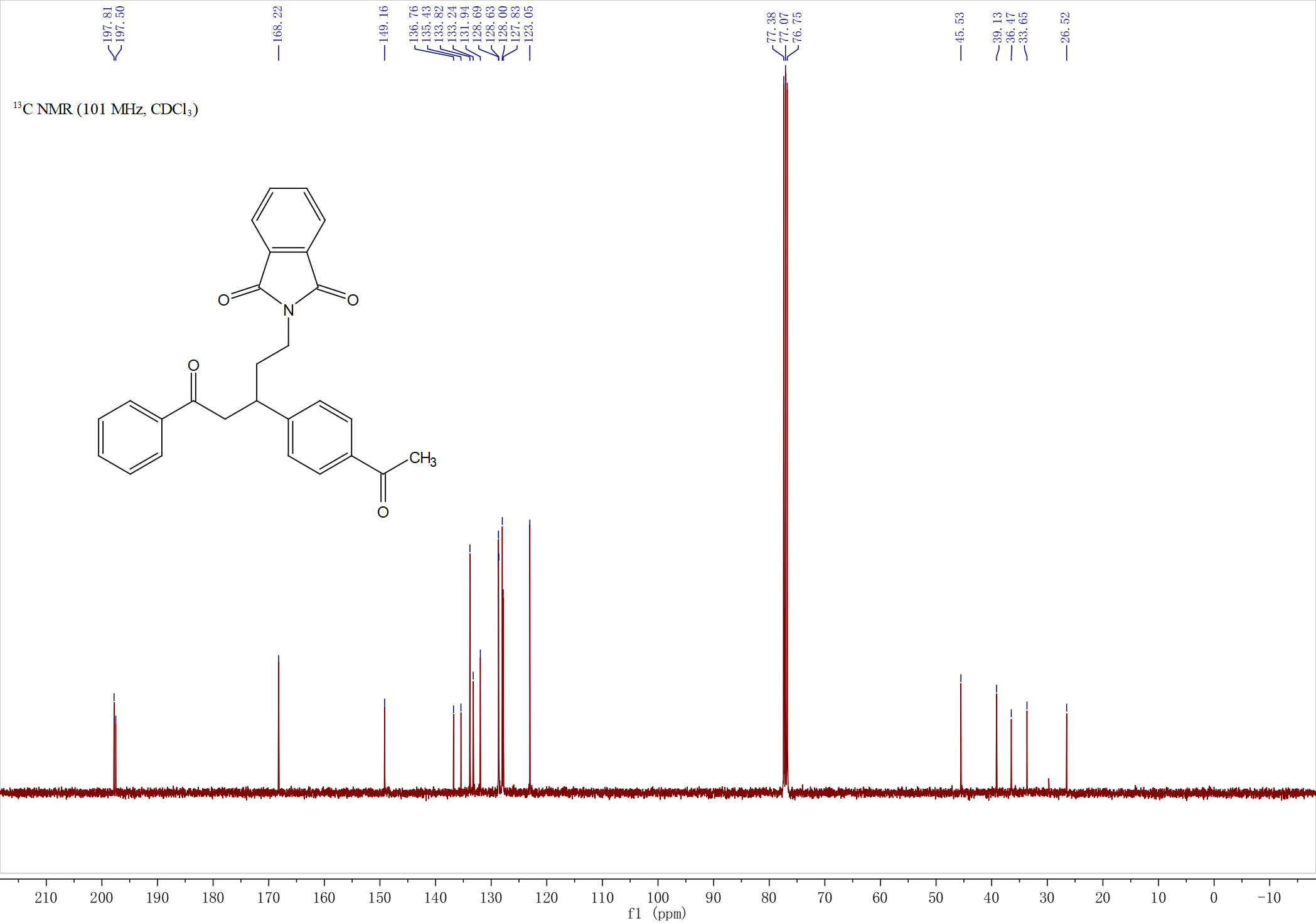


**3-(4-acetylphenyl)-1-phenyldodecan-1-one (4k)**


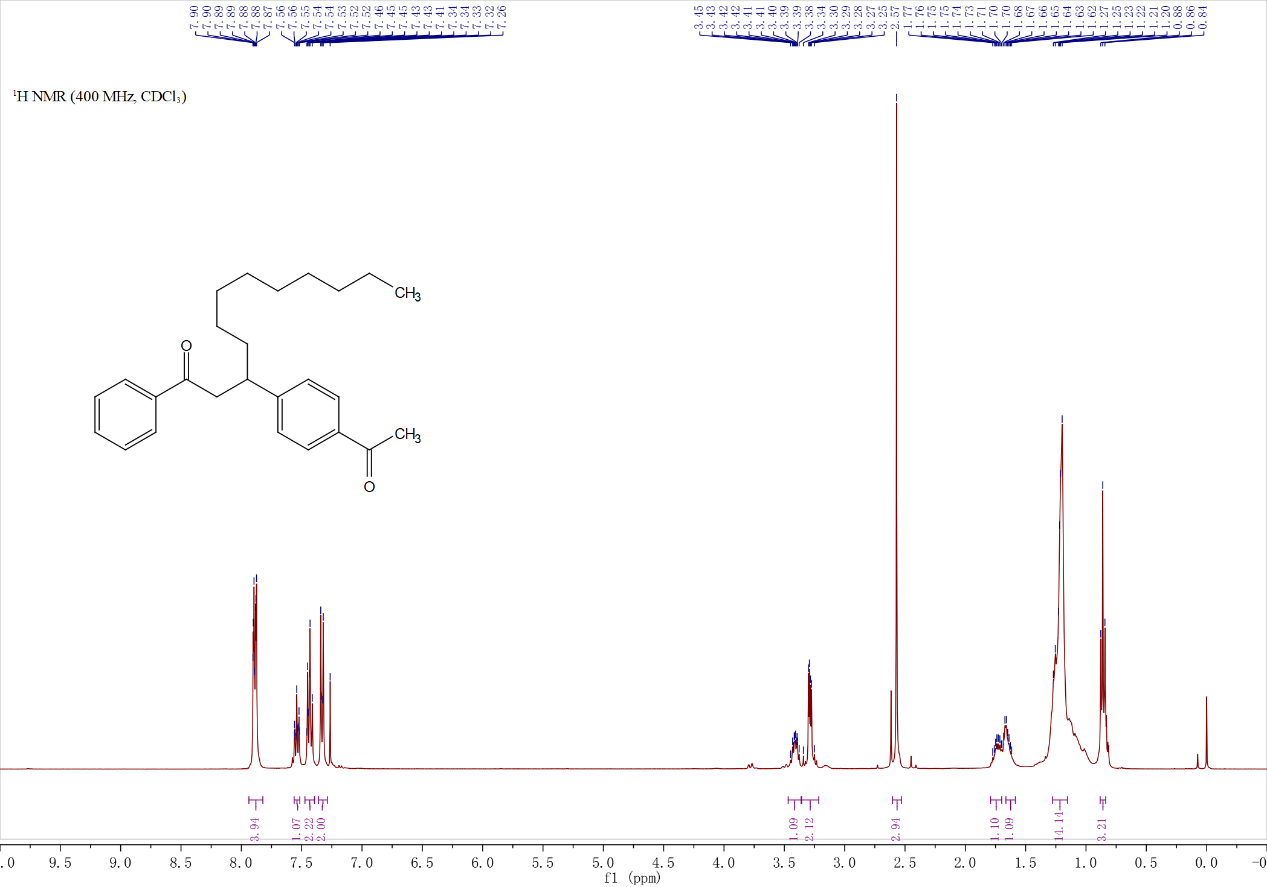


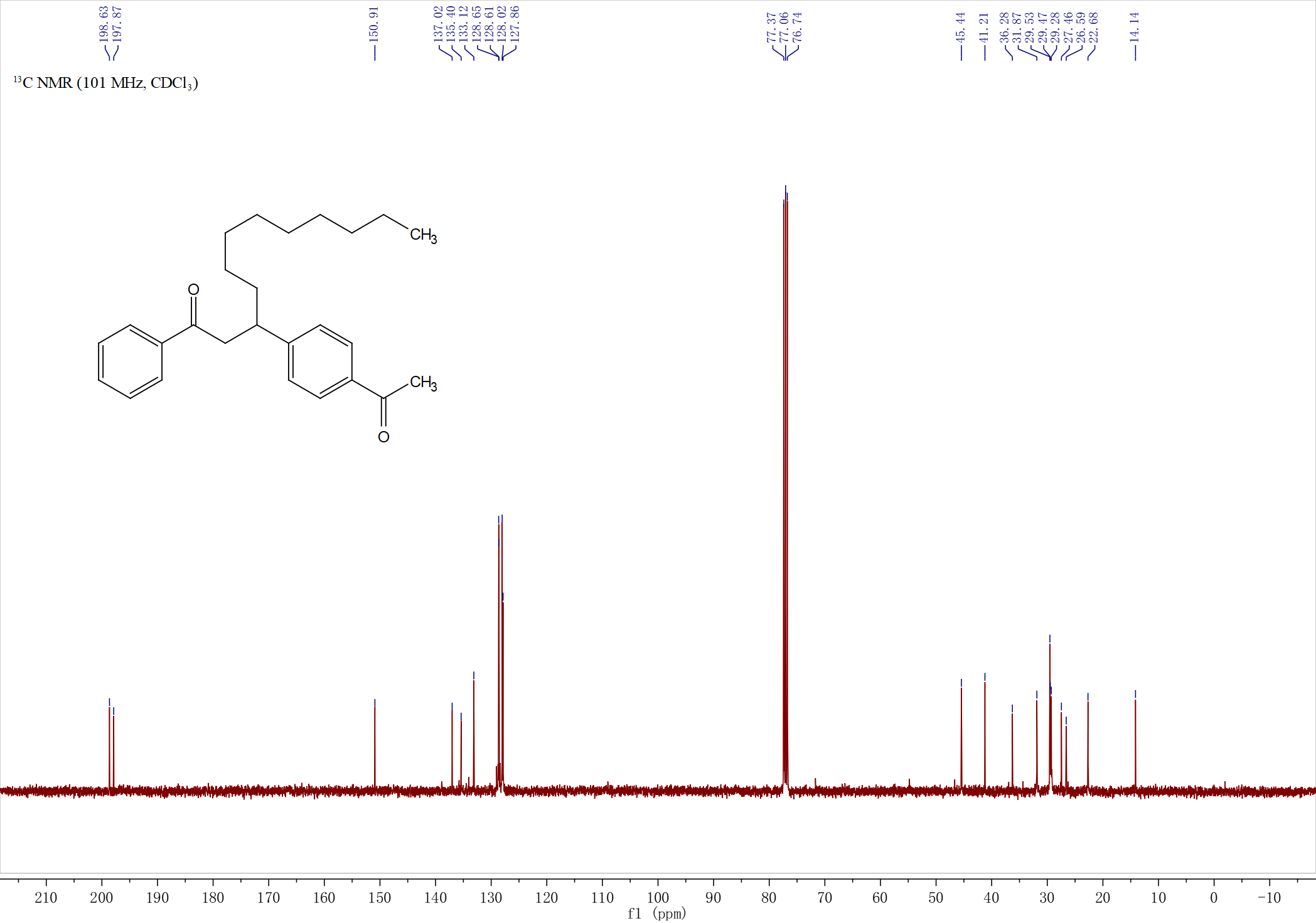


**3-(4-acetylphenyl)-5,5-dimethyl-1-phenylhexan-1-one (4l)**


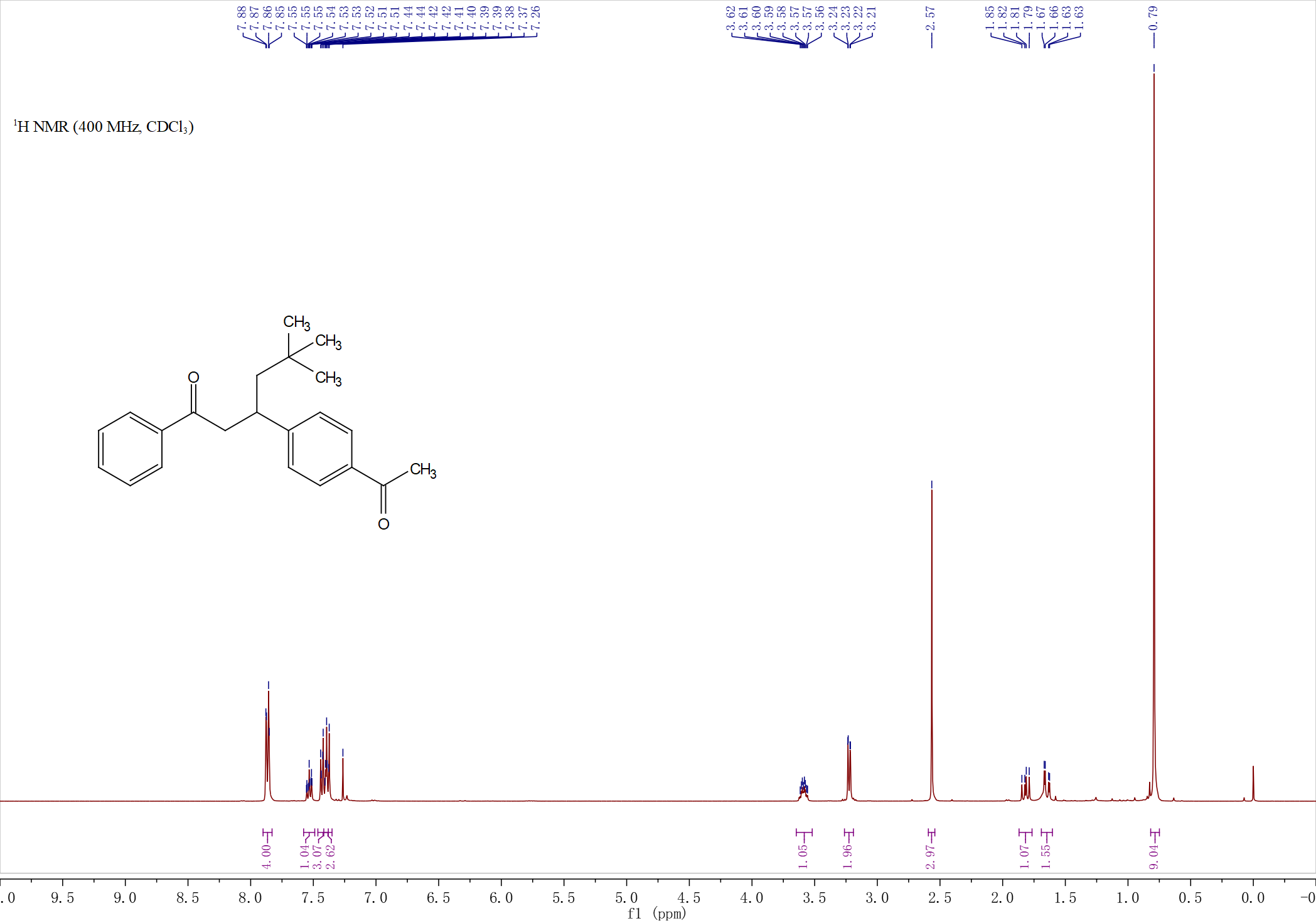


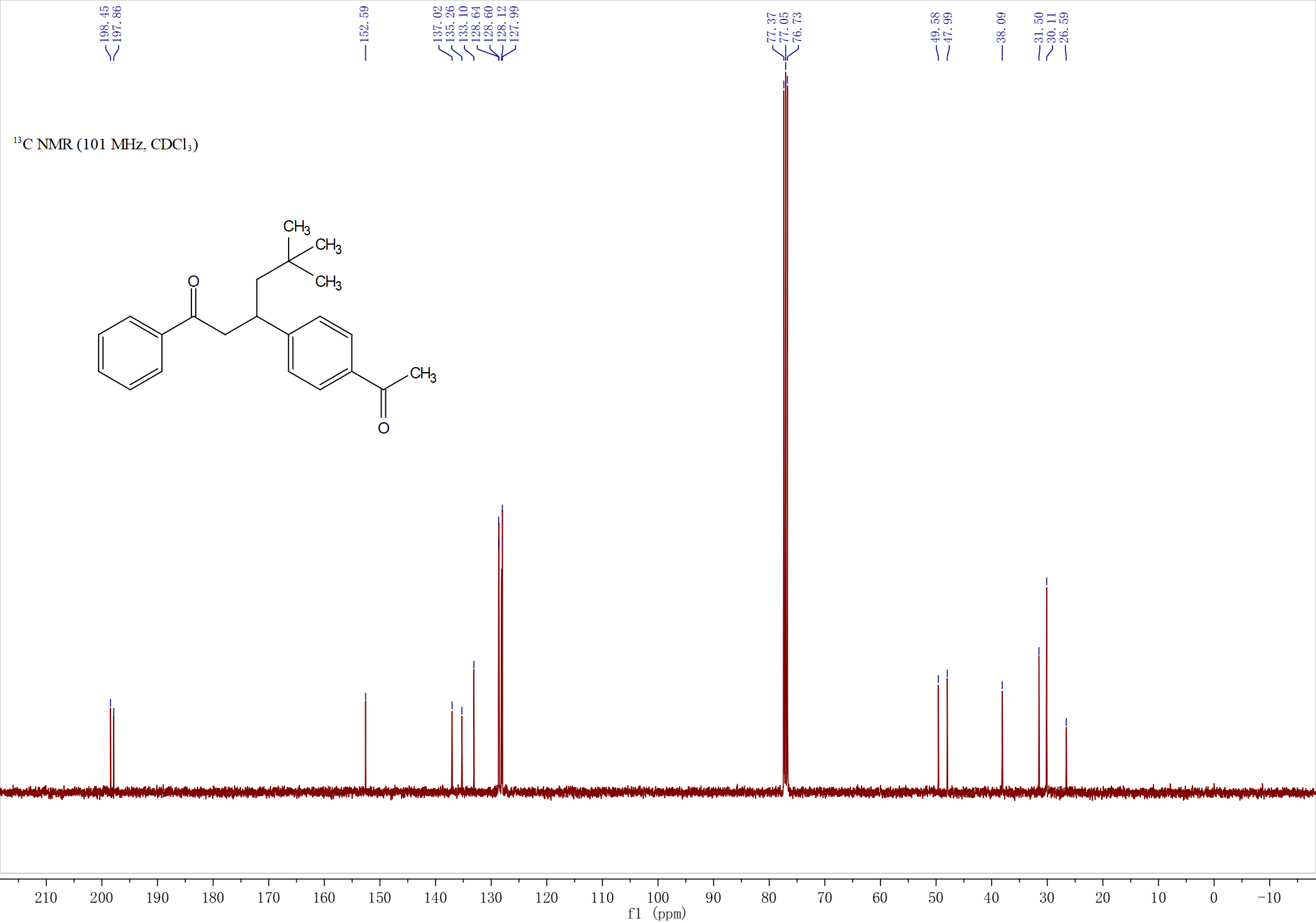


**3-(4-acetylphenyl)-3-cyclopropyl-1-phenylpropan-1-one (4m)**


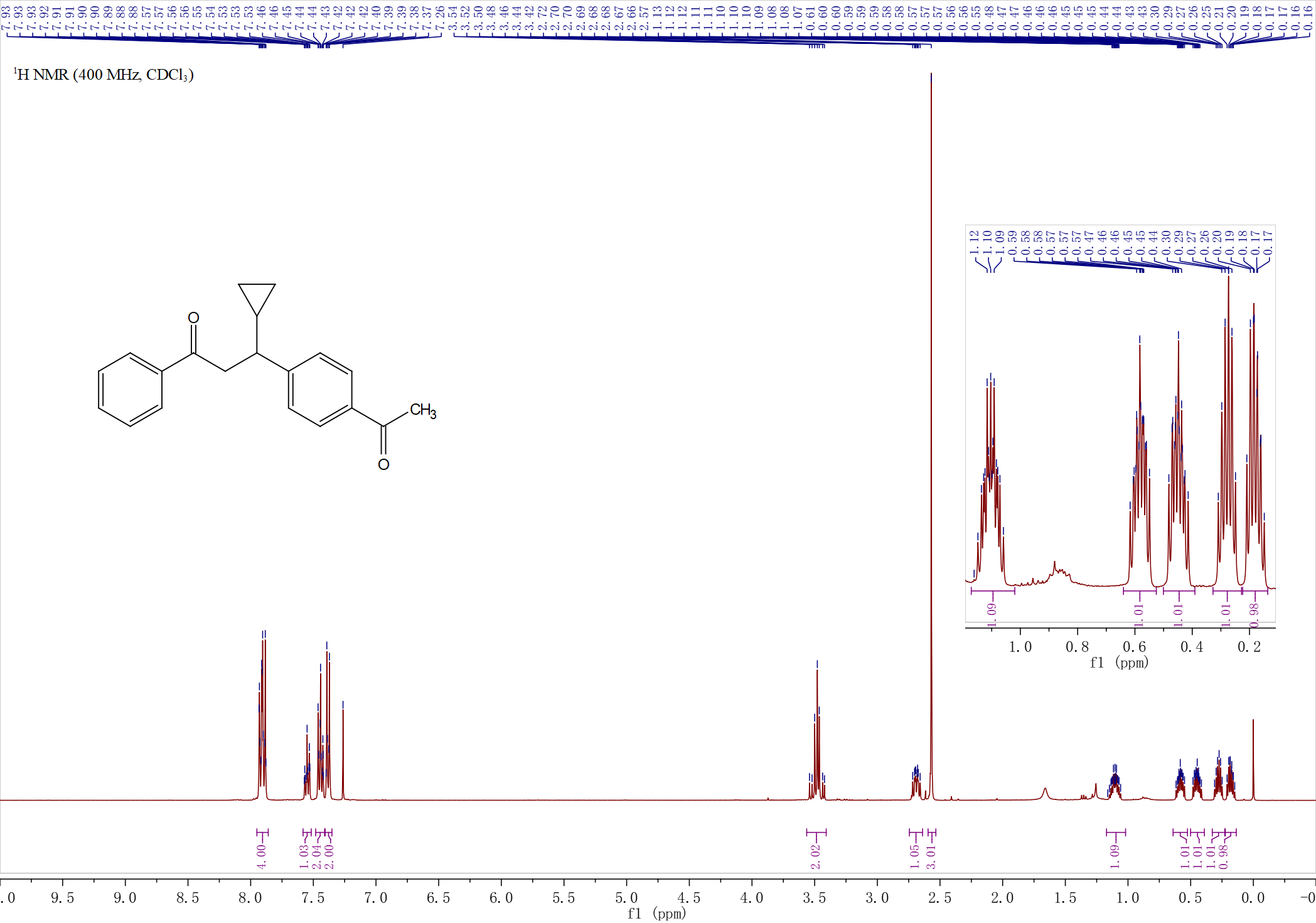


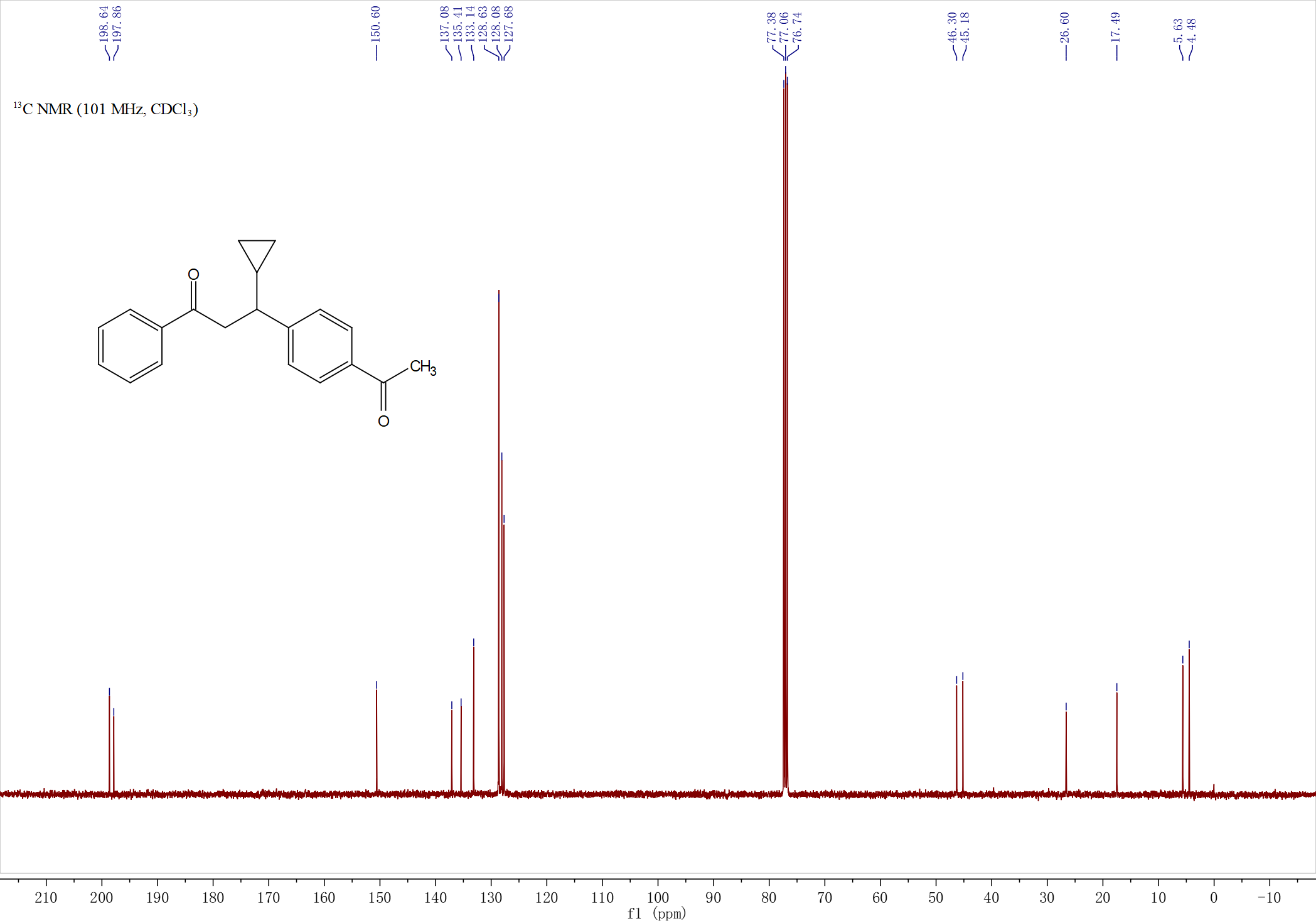


**3-(4-acetylphenyl)-3-cyclohexyl-1-phenylpropan-1-one (4n)**


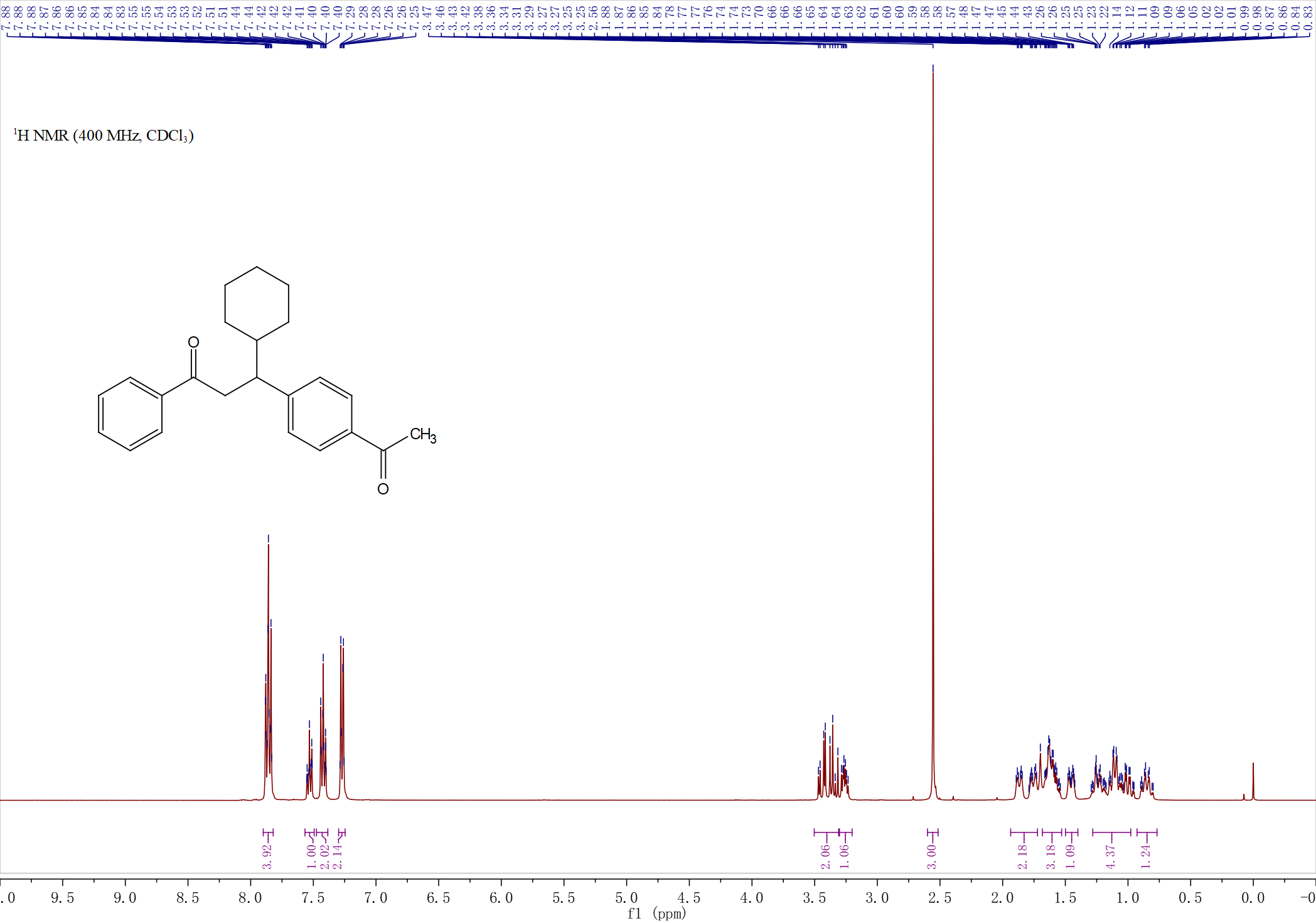


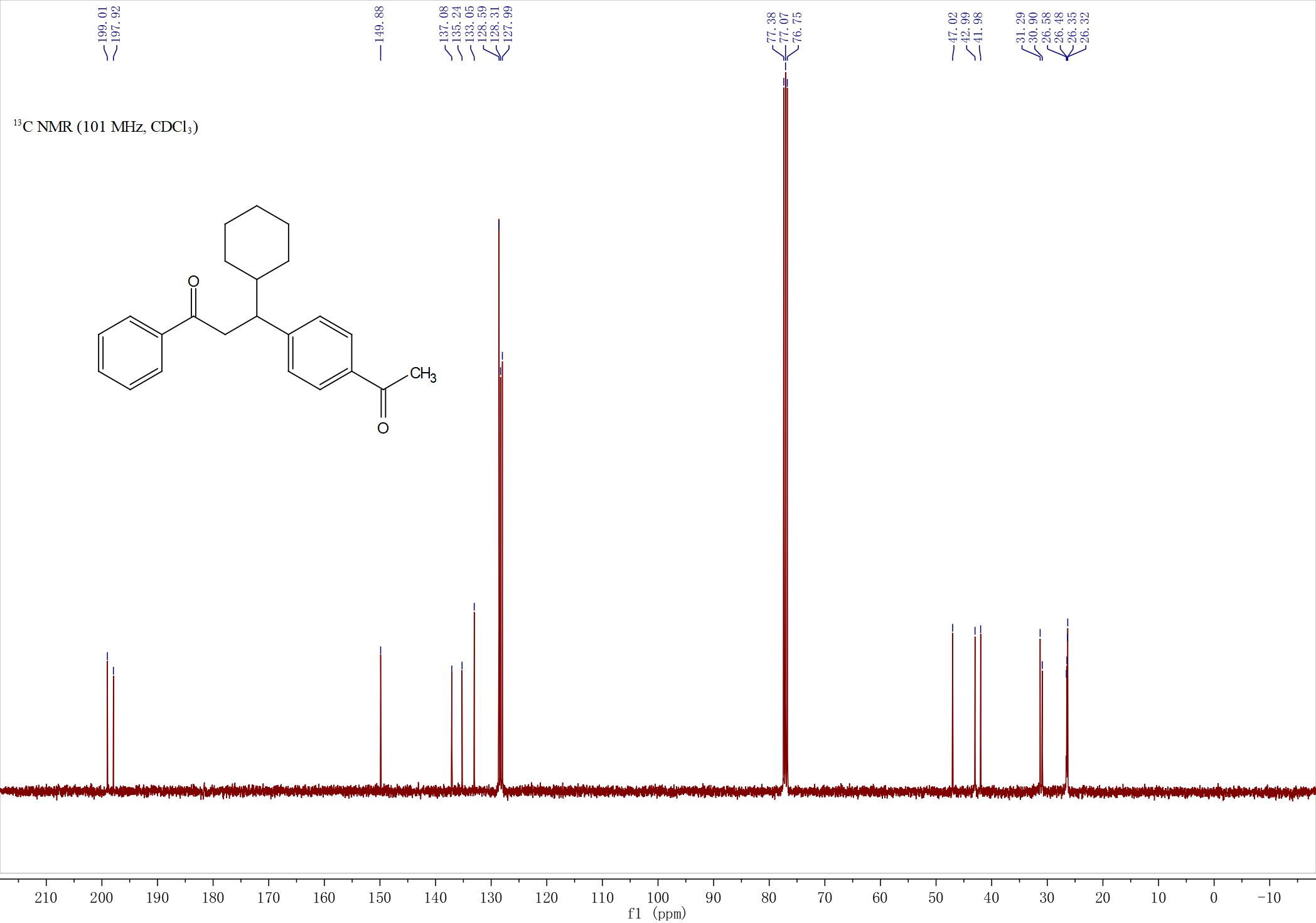


***tert*-butyl-4-(1-(4-acetylphenyl)-3-oxo-3-phenylpropyl) piperidine-1-carboxylate (4o)**


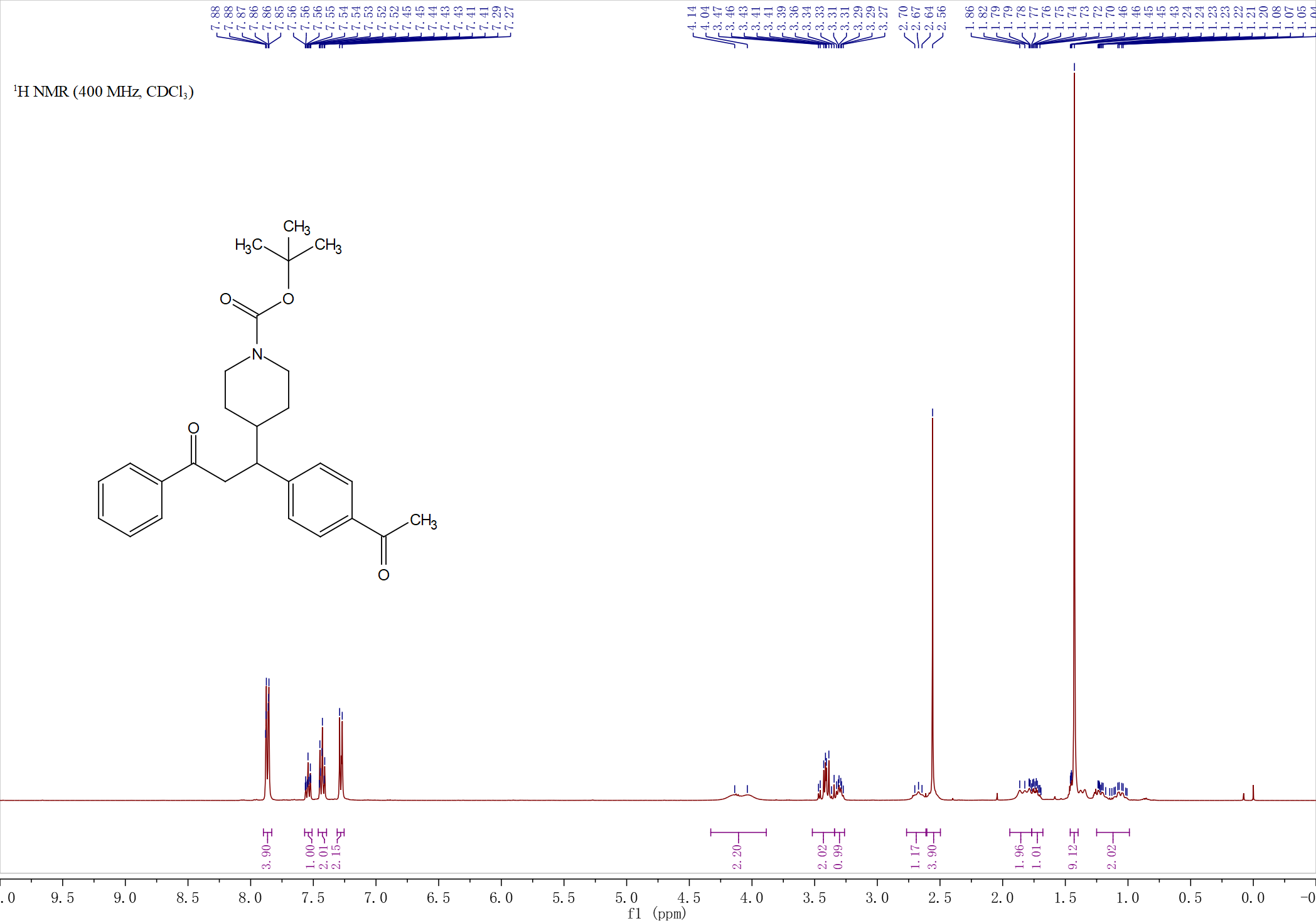


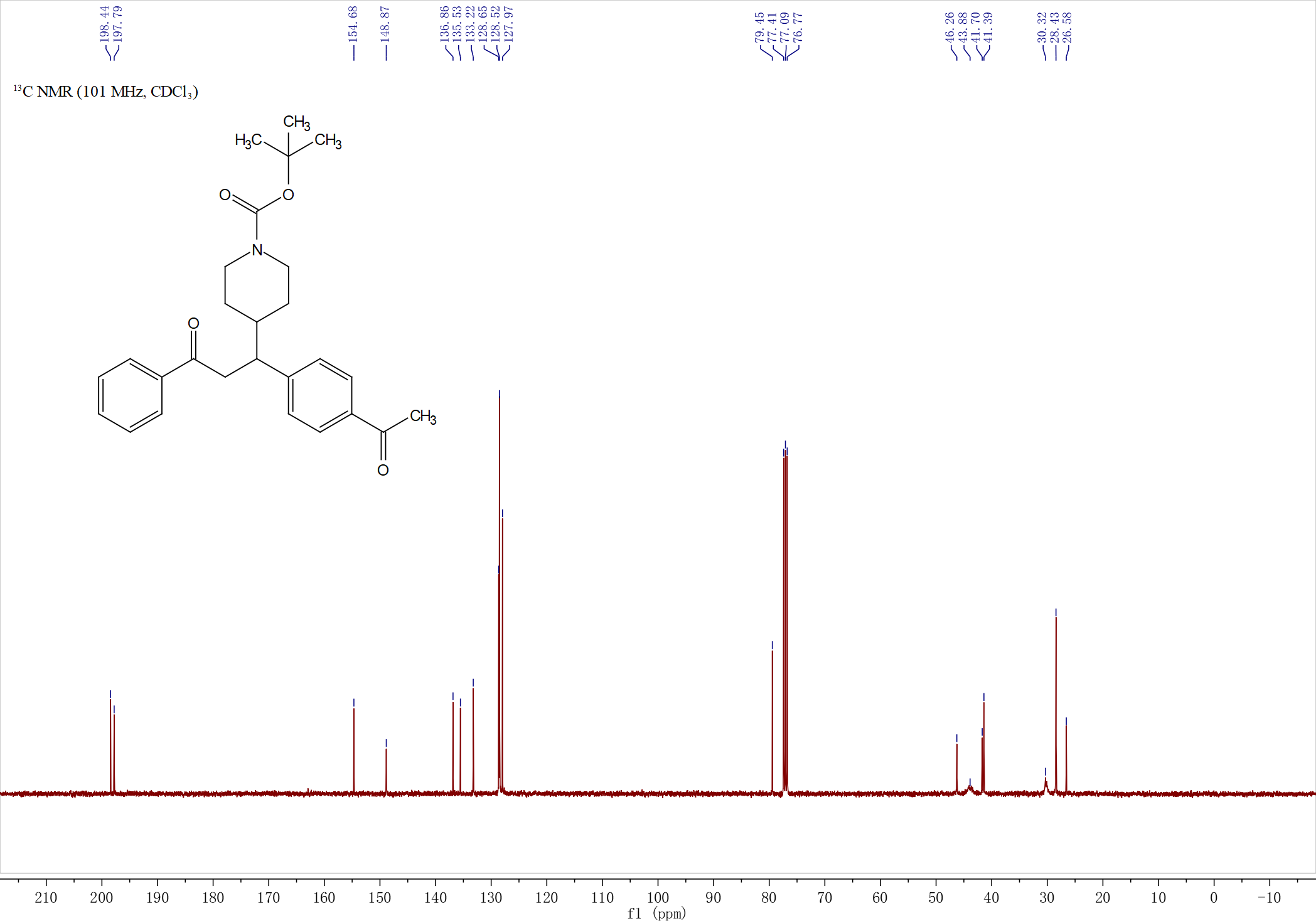


**3-(4-acetylphenyl)-3-(cyclohex-3-en-1-yl)-1-phenylpropan-1-one (4p)**


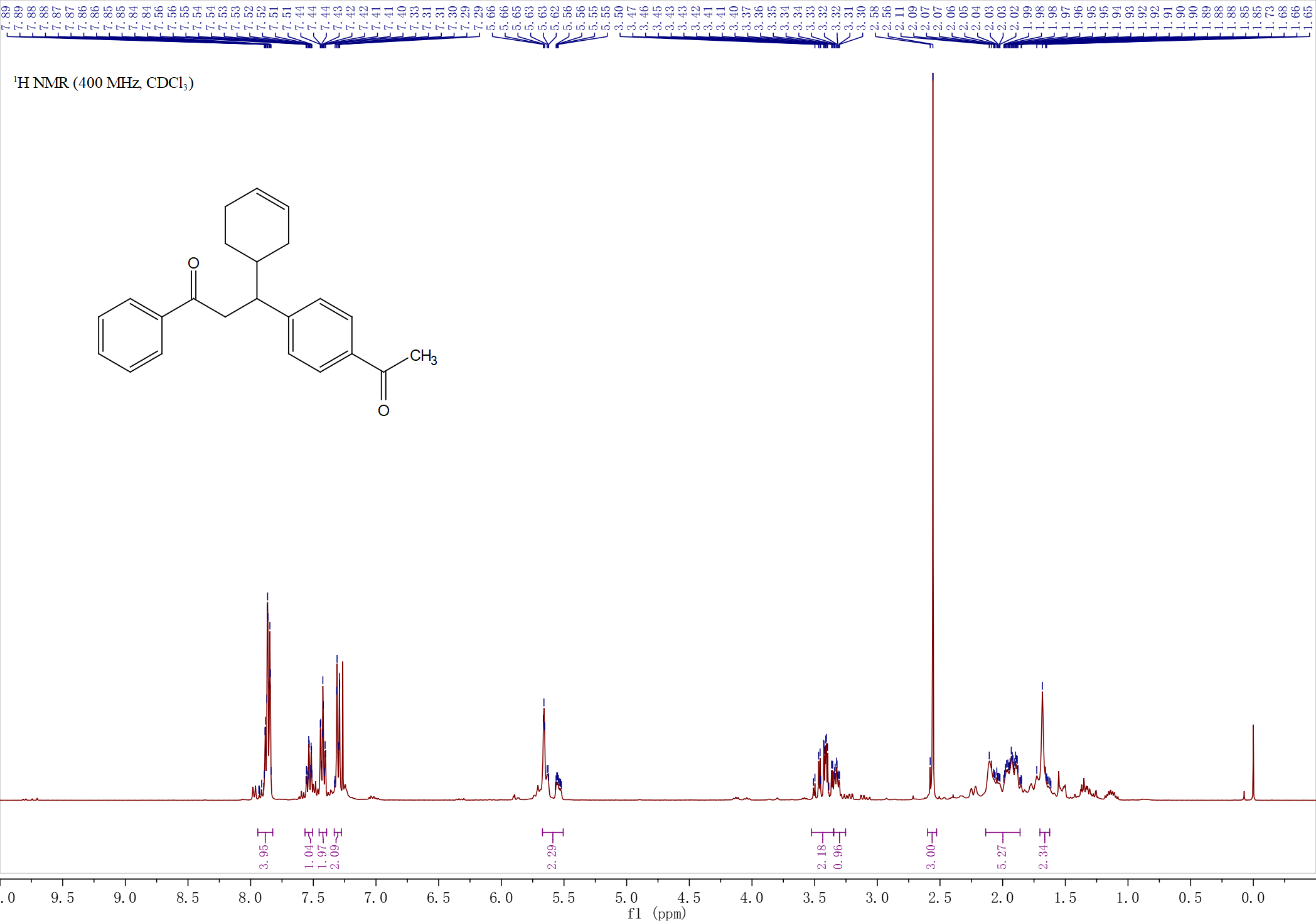


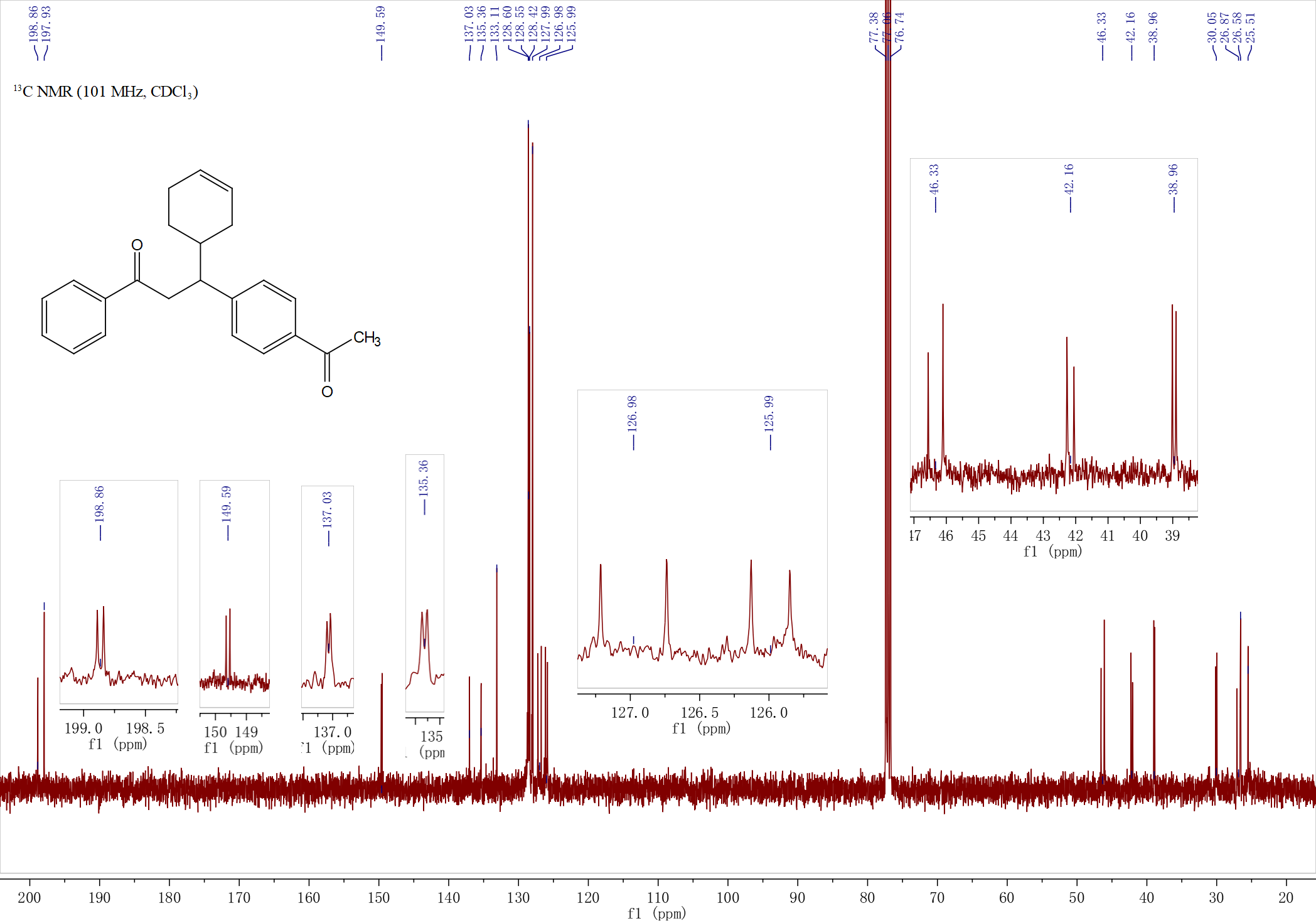


**3-(4-acetylphenyl)-5-(4-isopropylphenyl)-4-methyl-1-phenylpentan-1-one (4q)**


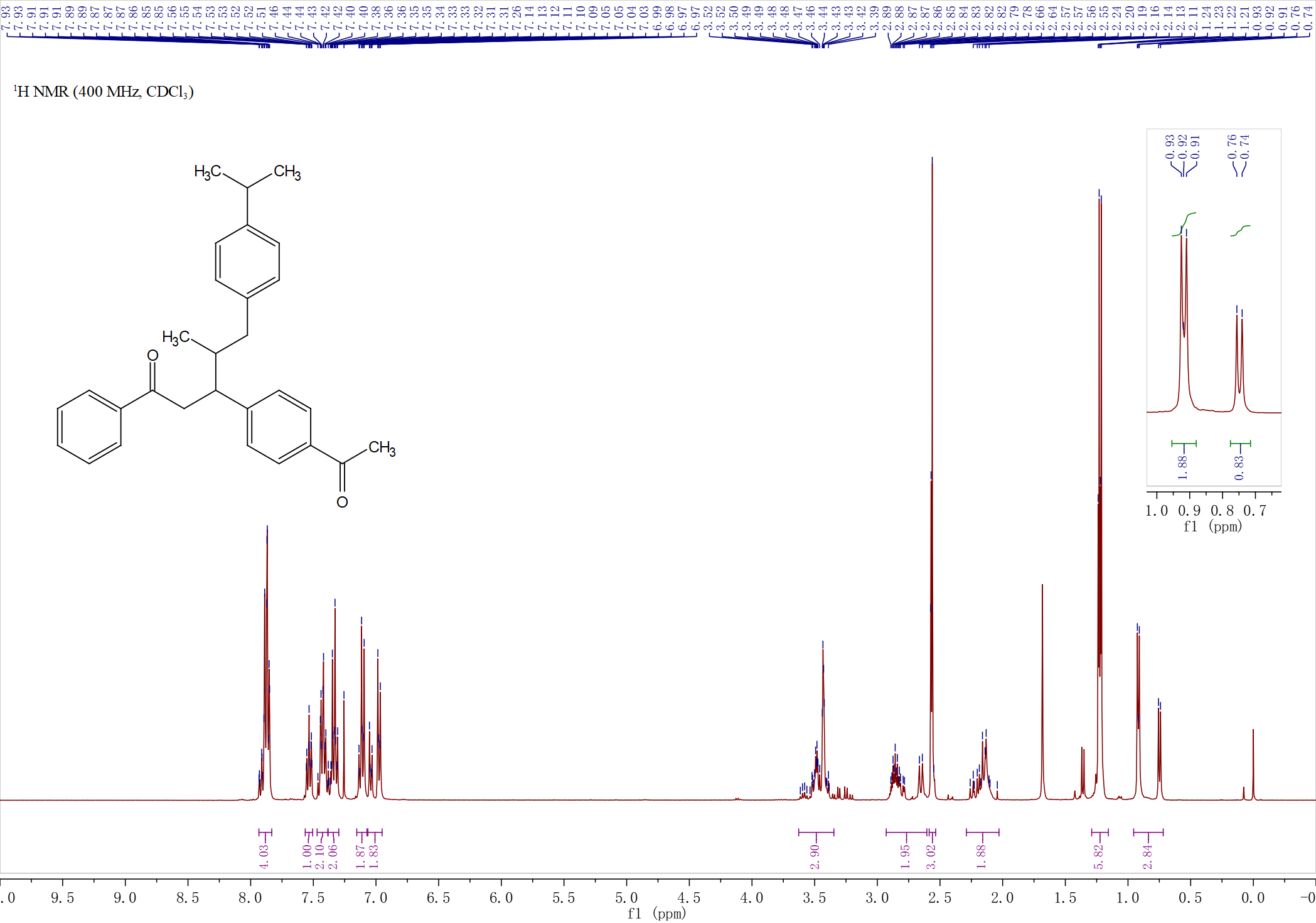


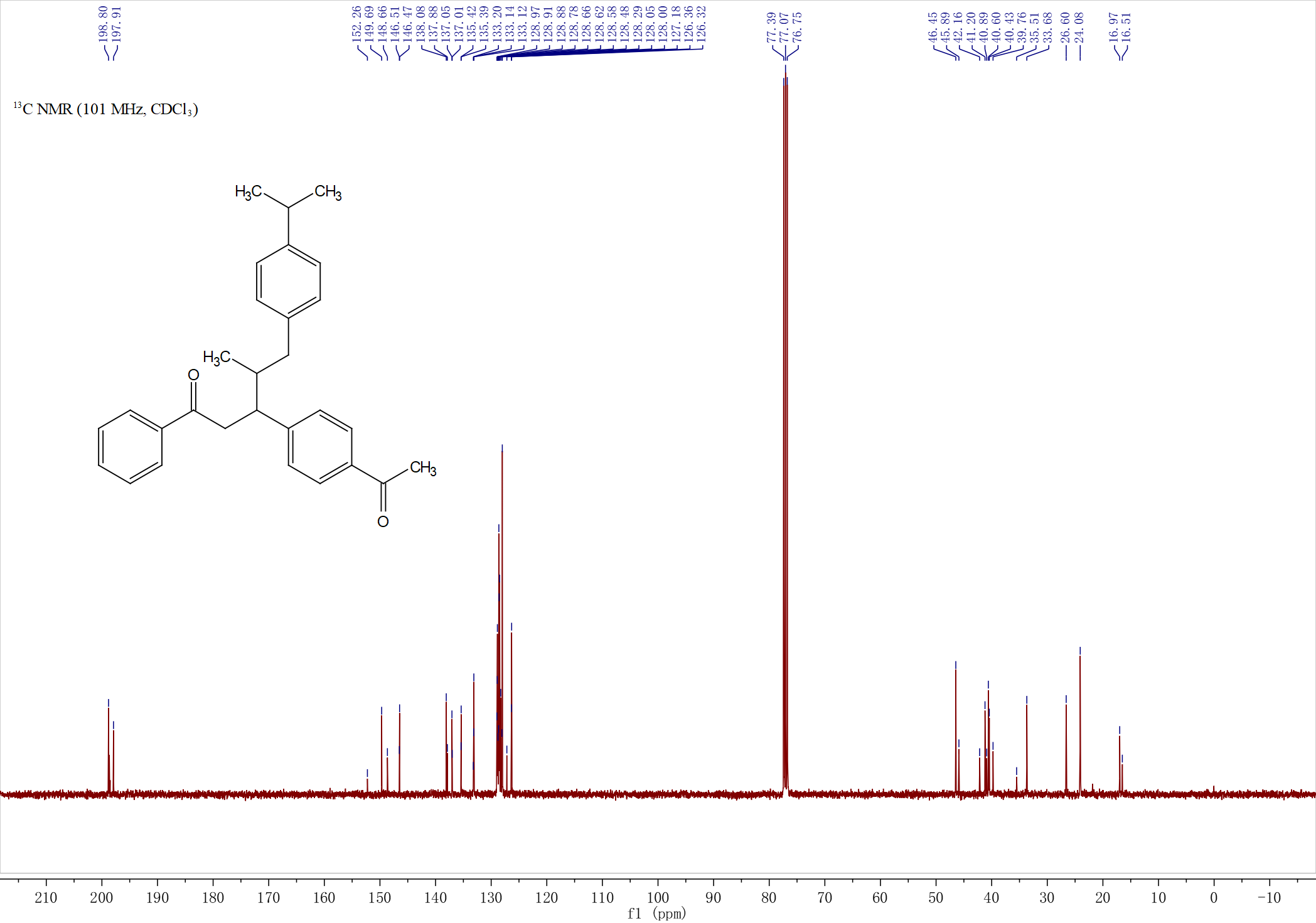


**3-(4-acetylphenyl)-1-(4-methoxyphenyl)-4-methylpentan-1-one (4r)**


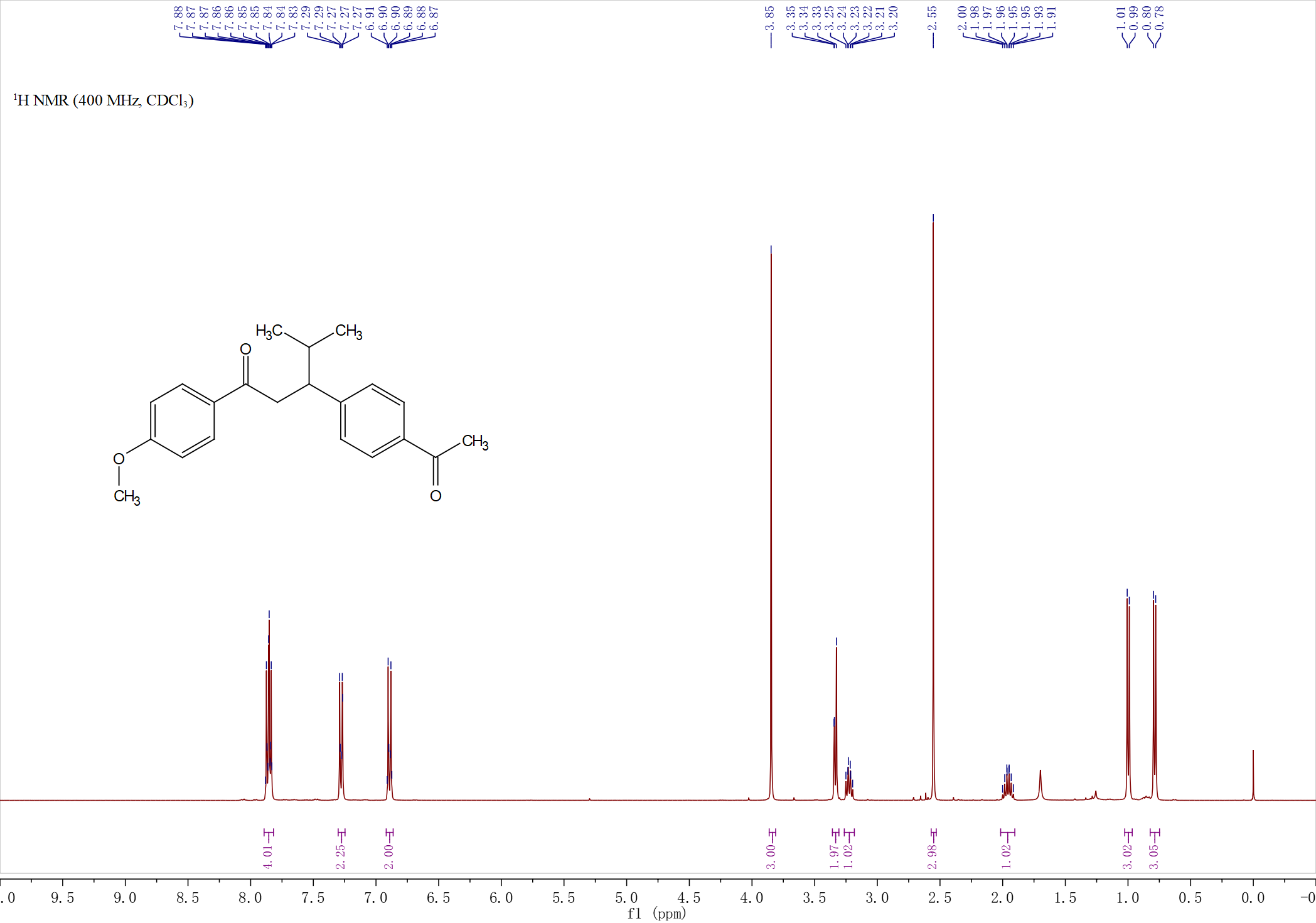


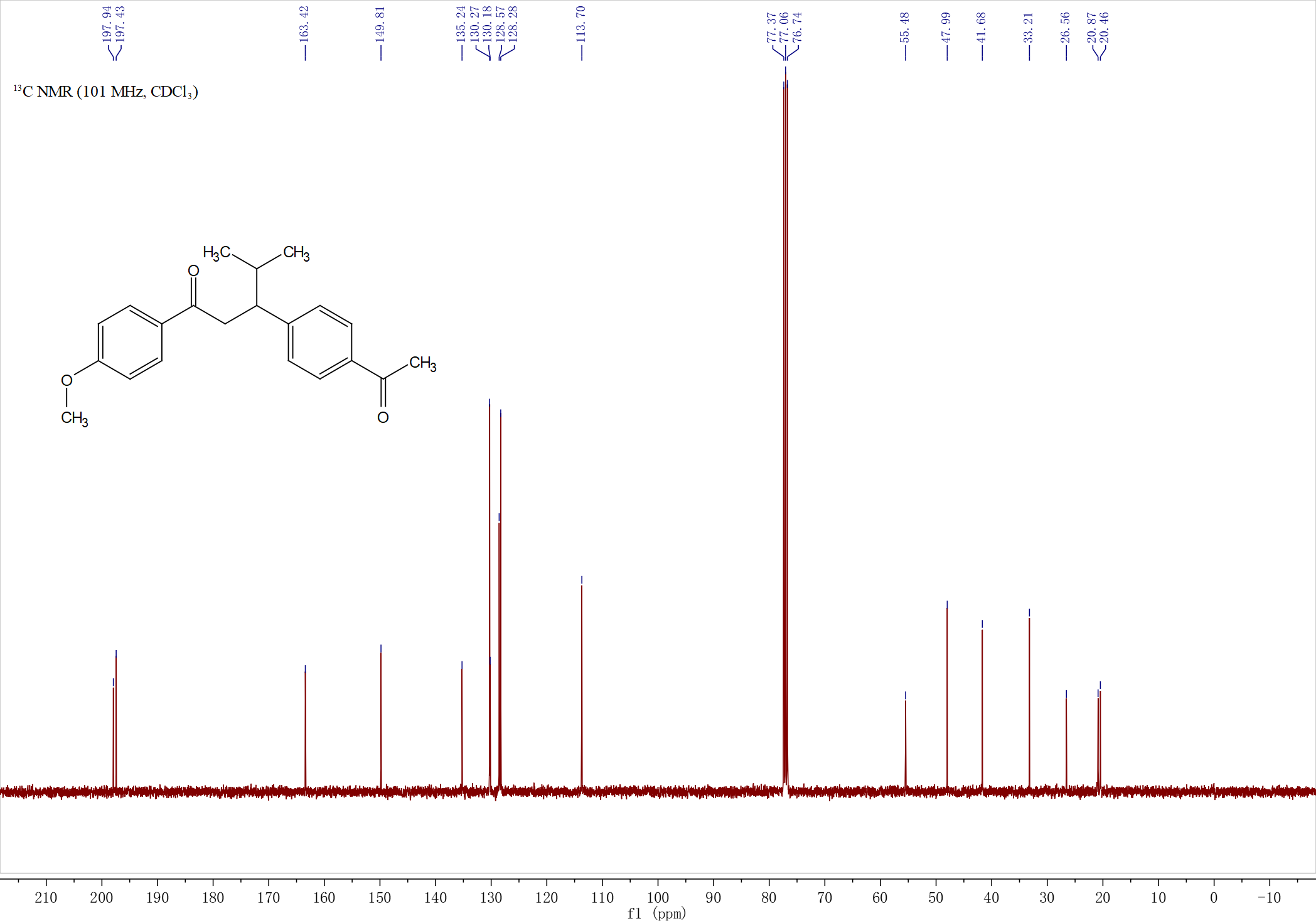


**1-(4-acetylphenyl)-4,4-dimethyl-1-phenylpentan-3-one (4s)**


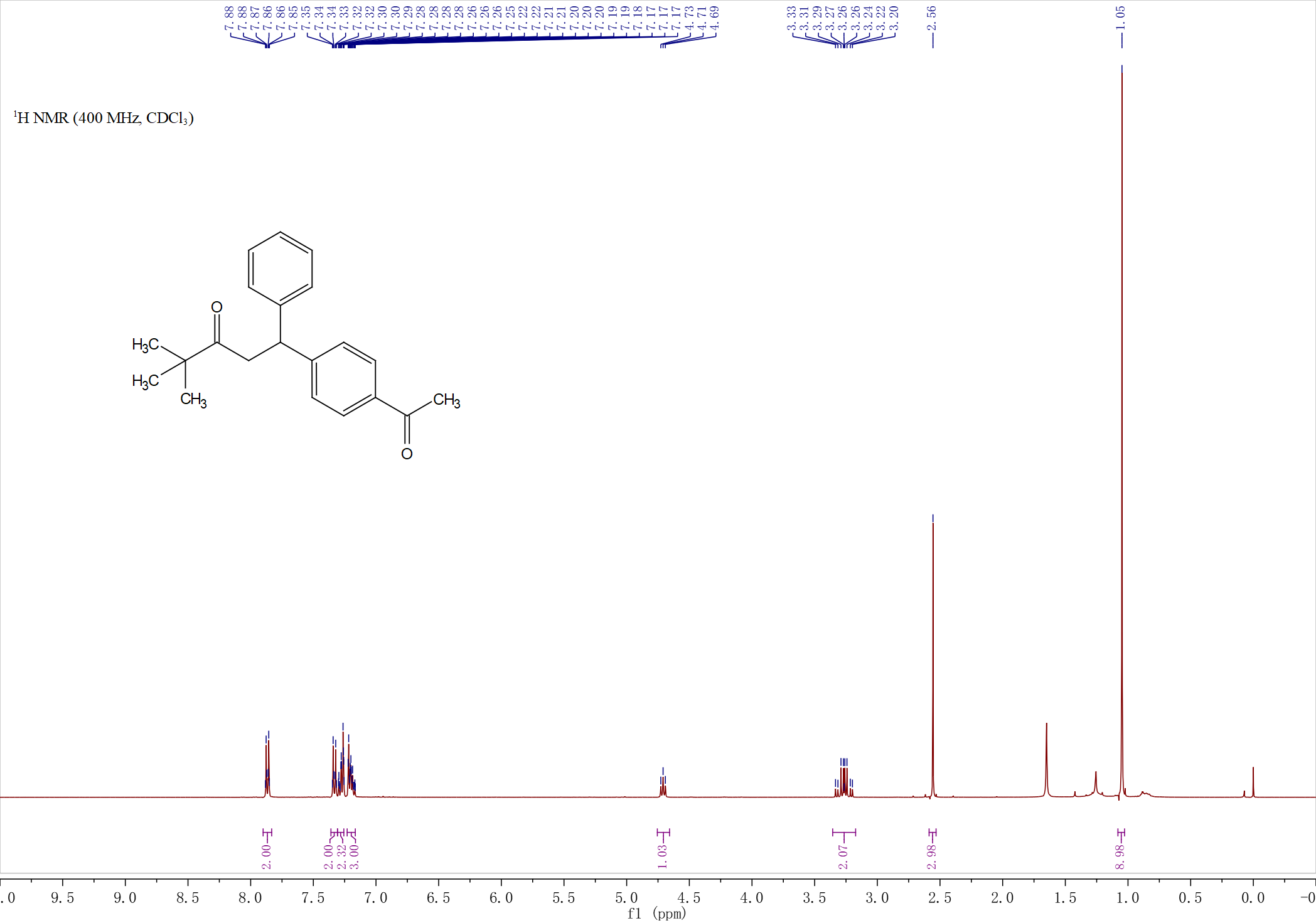


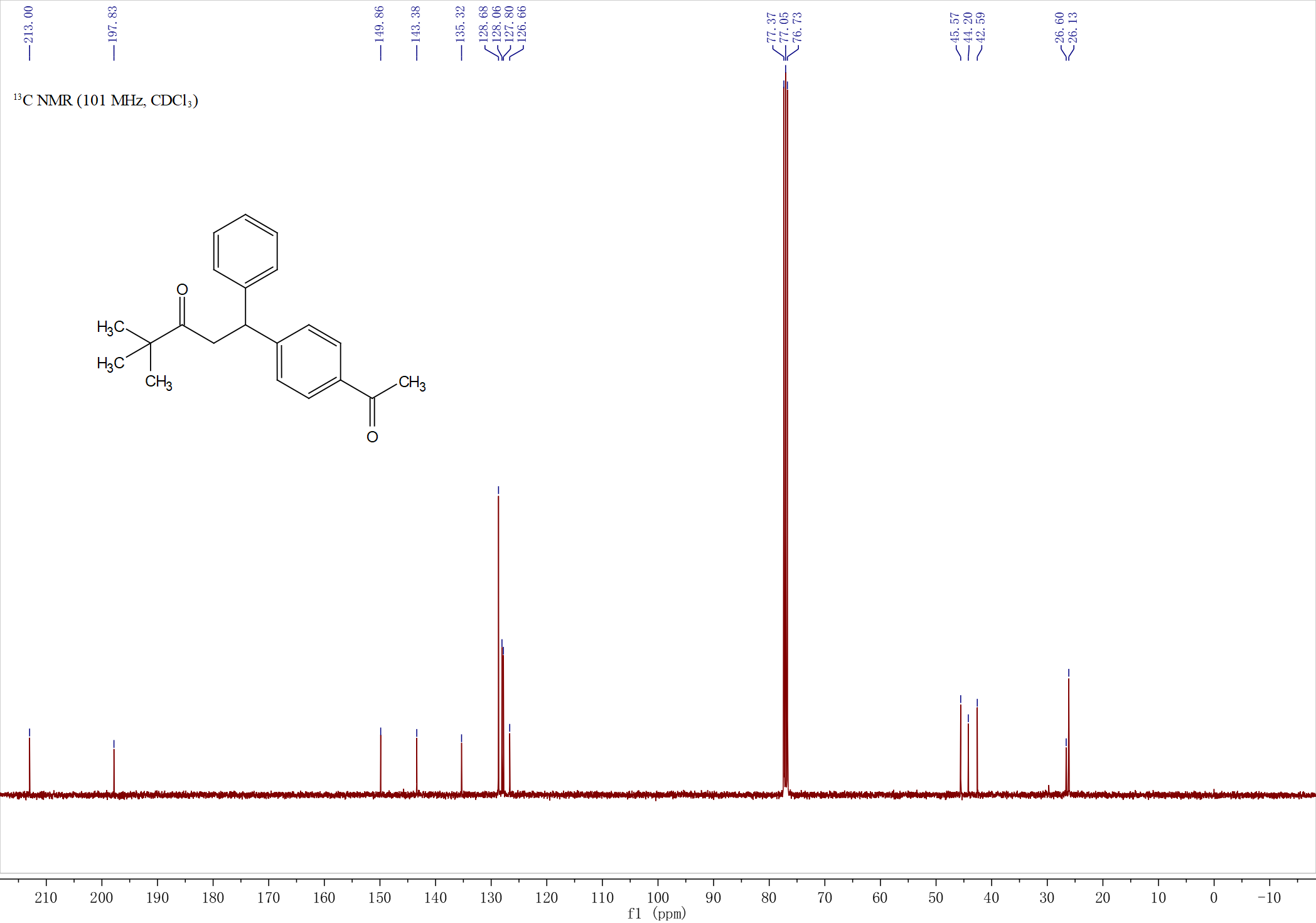


**3-(4-acetylphenyl)-1-((1s,3S)-adamantan-1-yl)-3-phenylpropan-1-one (4t)：**


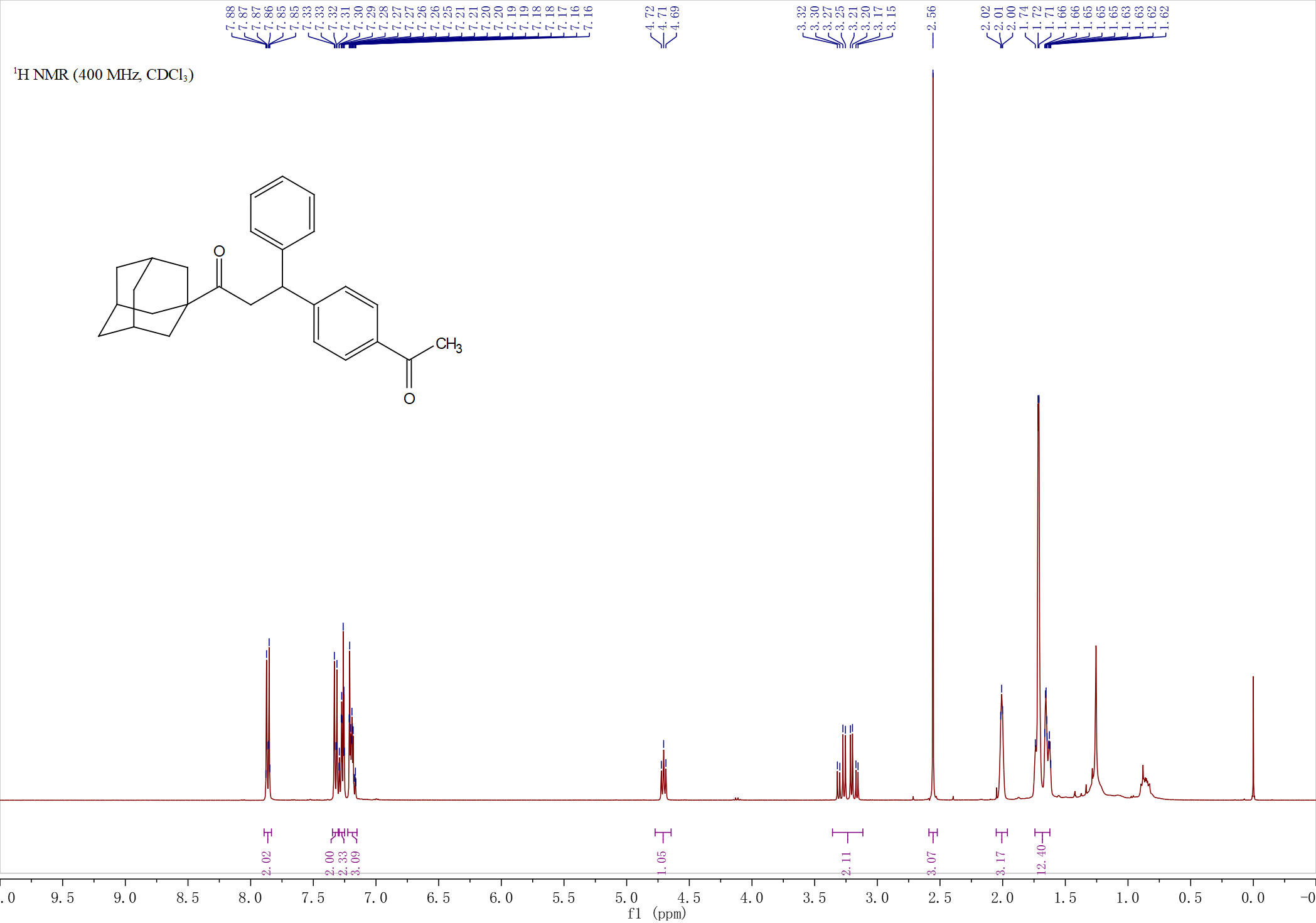


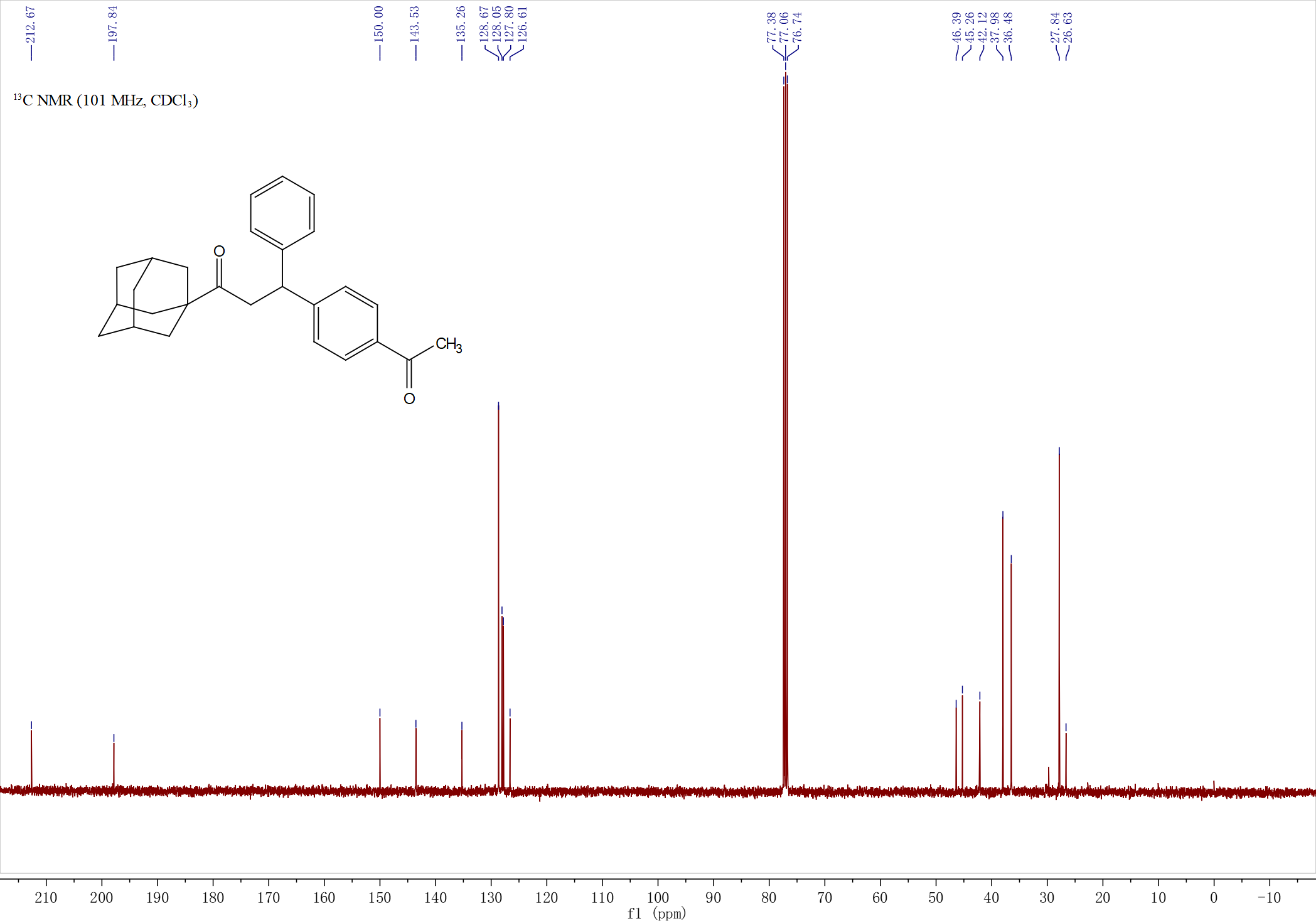


**1-(4-acetylphenyl)-4-methyl-1-phenylpentan-3-one (4u):**


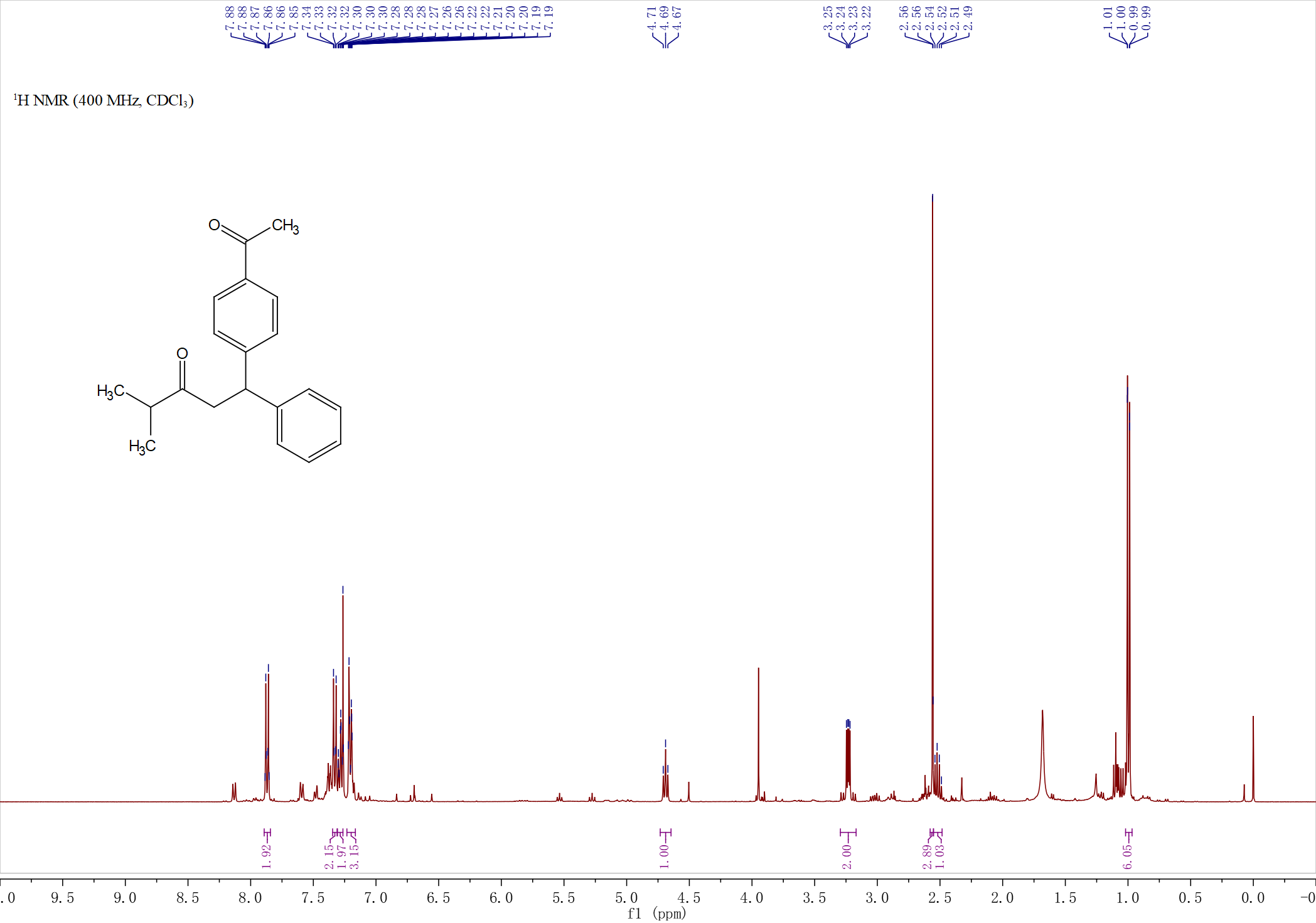


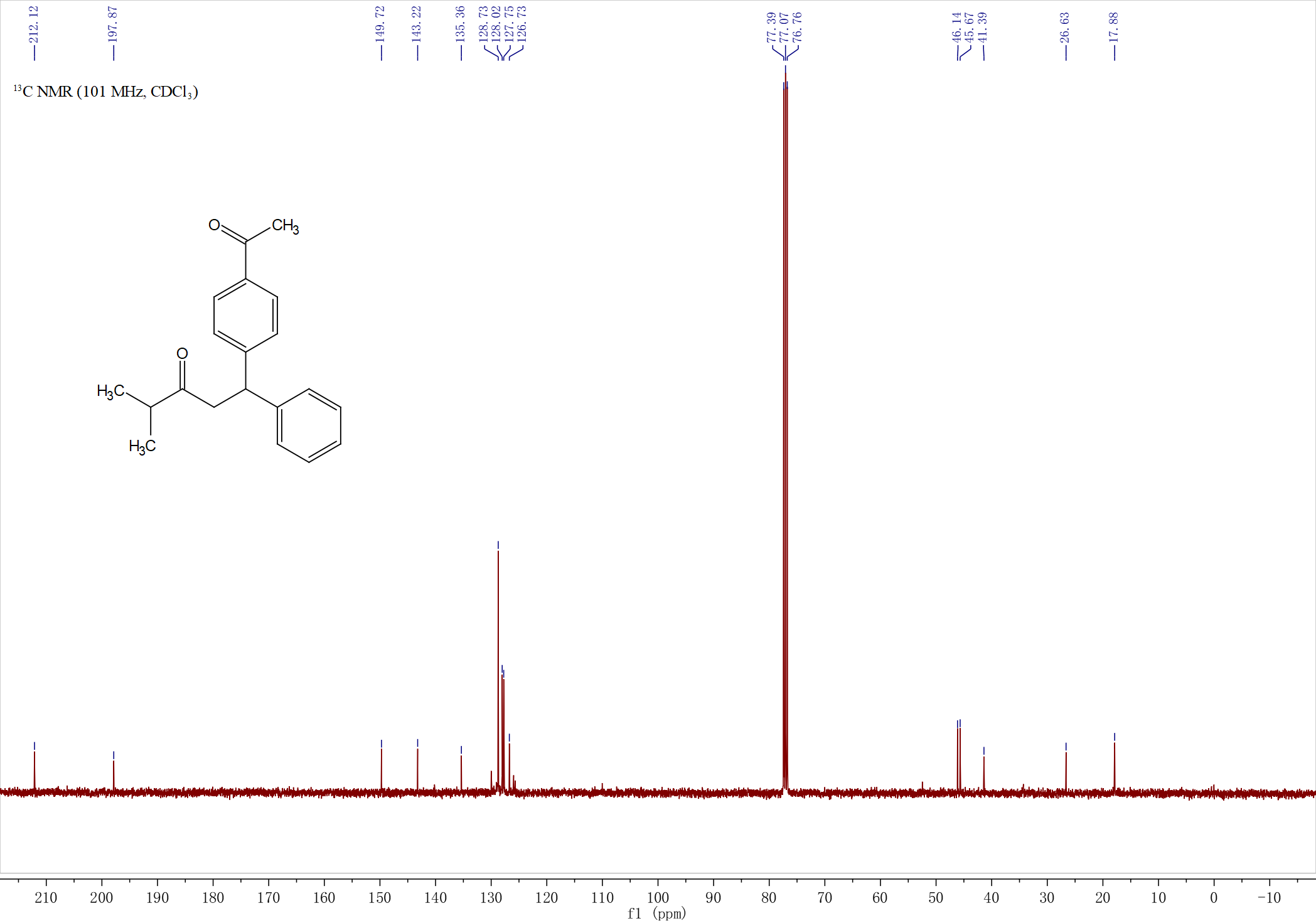


***tert*-butyl (*R*)-4-(3-(4-acetylphenyl)-3-phenylpropanoyl)piperidine-1-carboxylate (4v):**

***N*-(cyclohexylcarbamoyl)-4-(4-methyl-1-oxo-1-phenylpentan-3-yl)benzenesulfonamide (3m-urea)**

***tert*-butyl-4-(1-(3-(4-methoxybenzyl)-4-oxo-3,4-dihydroquinazolin-6-yl)-3-oxo-3-phenylpropyl)piperidine-1-carboxylate (4w)**

**(3a*R*,5*R*,6*S*,6a*R*)-5-((*S*)-2,2-dimethyl-1,3-dioxolan-4-yl)-2,2-dimethyltetrahydrofuro[2,3-*d*][1,3]dioxol-6-yl 4-((*R*)-4-methyl-1-oxo-1-phenylpentan-3-yl)benzoate (5a)**

**(*R*)-2,5,7,8-tetramethyl-2-((4*R*,8*R*)-4,8,12-trimethyltridecyl)chroman-6-yl 4-((*R*)-4-methyl-1-oxo-1-phenylpentan-3-yl)benzoate (5b)**

**(8*R*,9*S*,13*S*,14*S*)-13-methyl-17-oxo-7,8,9,11,12,13,14,15,16,17-decahydro-6*H*-cyclopenta[*a*]phenanthren-3-yl 4-((*R*)-4-methyl-1-oxo-1-phenylpentan-3-yl)benzoate (5c)**

**4-methyl-1-phenyl-3-(4-(4-(2,3,4-trimethoxybenzyl)piperazine-1-carbonyl)phenyl)pentan-1-one (5d)**

**4-methyl-1-phenyl-3-(4-(4-(4-phenylquinolin-2-yl)piperazine-1-carbonyl)phenyl)pentan-1-one (5e)**

**3-(3-(4-(cyclopropanecarbonyl)piperazine-1-carbonyl)-4-fluorophenyl)-4-methyl-1-phenylpentan-1-one (5f)**

***tert*-butyl-4-(1-(4-((3-chloro-4-fluorophenyl)amino)quinazolin-6-yl)-3-oxo-3-phenylpropyl)piperidine-1-carboxylate (5g)**

**11. HPLC Spectra**

***Rac*-(1,3-diphenyl-3-(*p*-tolyl)propan-1-one (3a)**

***(R*)-(1,3-diphenyl-3-(*p*-tolyl)propan-1-one (3a)**

***R*ac-3-(4-methoxyphenyl)-1,3-diphenylpropan-1-one (3b)**

**(*R*)-3-(4-methoxyphenyl)-1,3-diphenylpropan-1-one (3b)**

***R*ac-Methyl-4-(3-oxo-1,3-diphenylpropyl)benzoate (3c)**

**(*R*)-Methyl -4-(3-oxo-1,3-diphenylpropyl)benzoate (3c)**

***Rac*-3-(4-acetylphenyl)-1,3-diphenylpropan-1-one (3d)**

**(*R*)-3-(4-acetylphenyl)-1,3-diphenylpropan-1-one (3d)**

***R*ac-3-(naphthalen-2-yl)-1,3-diphenylpropan-1-one (3e)**

**(*R*)-3-(naphthalen-2-yl)-1,3-diphenylpropan-1-one (3e)**

***Rac*-4-methyl-1-phenyl-3-(*p*-tolyl)pentan-1-one (3f)**

**(*R*)-4-methyl-1-phenyl-3-(*p*-tolyl)pentan-1-one (3f)**

***Rac*-4-methyl-3-(4-(methylthio)phenyl)-1-phenylpentan-1-one (3g)**

**(*R*)-4-methyl-3-(4-(methylthio)phenyl)-1-phenylpentan-1-one (3g)**

**(*R*)-3-([1,1'-biphenyl]-4-yl)-4-methyl-1-phenylpentan-1-one (3h)**

***Rac*-methyl-4-(4-methyl-1-oxo-1-phenylpentan-3-yl)benzoate (3i)**

**(*R*)-methyl-4-(4-methyl-1-oxo-1-phenylpentan-3-yl)benzoate (3i)**

**(*Rac*)-3-(4-acetylphenyl)-4-methyl-1-phenylpentan-1-one (3j)**

**(*R*)-3-(4-acetylphenyl)-4-methyl-1-phenylpentan-1-one (3j)**

**(*Rac*)-4-methyl-1-phenyl-3-(4-(trifluoromethyl)phenyl) pentan-1-one (3k)**

**(*R*)-4-methyl-1-phenyl-3-(4-(trifluoromethyl)phenyl) pentan-1-one (3k)**

**(*Rac*)-4-(4-methyl-1-oxo-1-phenylpentan-3-yl)benzonitrile (3l)**

**(*R*)-4-(4-methyl-1-oxo-1-phenylpentan-3-yl)benzonitrile (3l)**

**(*Rac*)-4-(4-methyl-1-oxo-1-phenylpentan-3-yl)benzene sulfonamide (3m)**

**(*R*)-4-(4-methyl-1-oxo-1-phenylpentan-3-yl)benzene sulfonamide (3m)**

**(*Rac*)-*N*-(4-(4-methyl-1-oxo-1-phenylpentan-3-yl)phenyl) acetamide (3n)**

**(*R*)-*N*-(4-(4-methyl-1-oxo-1-phenylpentan-3-yl)phenyl) acetamide (3n)**

**(*Rac*)-3-(3-acetylphenyl)-4-methyl-1-phenylpentan-1-one (3o)**

**(*R*)-3-(3-acetylphenyl)-4-methyl-1-phenylpentan-1-one (3o)**

**(*Rac*)-5-(4-methyl-1-oxo-1-phenylpentan-3-yl)isobenzofuran-1(3*H*)-one (3p)**

**(*R*)-5-(4-methyl-1-oxo-1-phenylpentan-3-yl)isobenzofuran-1(3*H*)-one (3p)**

**(*Rac*)-3-(9*H*-fluoren-2-yl)-4-methyl-1-phenylpentan-1-one (3q)**

**(*R*)-3-(9*H*-fluoren-2-yl)-4-methyl-1-phenylpentan-1-one (3q)**

**(*Rac*)-4-methyl-3-(naphthalen-2-yl)-1-phenylpentan-1-one (3r)**

**(*R*)-4-methyl-3-(naphthalen-2-yl)-1-phenylpentan-1-one (3r)**

**(*Rac*)-3-(6-methoxynaphthalen-2-yl)-4-methyl-1-phenylpentan-1-one (3s)**

**(*R*)-3-(6-methoxynaphthalen-2-yl)-4-methyl-1-phenylpentan-1-one (3s)**

***tert*-butyl (*Rac*)-6-(4-methyl-1-oxo-1-phenylpentan-3-yl)-1*H*-indazole-1-carboxylate (3t)**

***tert*-butyl (*R*)-6-(4-methyl-1-oxo-1-phenylpentan-3-yl)-1*H*-indazole-1-carboxylate (3t)**

**(*Rac*)-3-(4-methoxybenzyl)-6-(4-methyl-1-oxo-1-phenylpentan-3-yl)quinazolin-4(3*H*)-one (3u)**

**(*R*)-3-(4-methoxybenzyl)-6-(4-methyl-1-oxo-1-phenylpentan-3-yl)quinazolin-4(3*H*)-one (3u)**

**(*Rac, E*)-1,3-diphenyl-5-(*p*-tolyl)pent-4-en-1-one (3v)**

**(*S, E*)-1,3-diphenyl-5-(*p*-tolyl)pent-4-en-1-one (3v)**

**(*Rac*, E)-3-isopropyl-5-(4-methoxyphenyl)-1-phenylpent-4-en-1-one (3w)**

**(*R*, E)-3-isopropyl-5-(4-methoxyphenyl)-1-phenylpent-4-en-1-one (3w)**

***tert*-butyl (*Rac, E*)-4-(1-(4-methoxyphenyl)-5-oxo-5-phenylpent-1-en-3-yl)piperidine-1-carboxylate (3x)**

***tert*-butyl (*R, E*)-4-(1-(4-methoxyphenyl)-5-oxo-5-phenylpent-1-en-3-yl)piperidine-1-carboxylate (3x)**

***tert*-butyl (*Rac, E*)-4-(1-(2,3-dihydrobenzofuran-5-yl)-5-oxo-5-phenylpent-1-en-3-yl)piperidine-1-carboxylate (3y):**

***tert*-butyl (*R, E*)-4-(1-(2,3-dihydrobenzofuran-5-yl)-5-oxo-5-phenylpent-1-en-3-yl)piperidine-1-carboxylate (3y):**

**(*Rac*)-4-methyl-1,3-diphenylpent-4-en-1-one (3z):**

**(*S*)-4-methyl-1,3-diphenylpent-4-en-1-one (3z):**

**(*Rac*)-3-(4-acetylphenyl)-1-(4-chlorophenyl)-3-phenyl propan-1-one (4a)**

**(*R*)-3-(4-acetylphenyl)-1-(4-chlorophenyl)-3-phenyl propan-1-one (4a)**

**(*Rac*)-3-(4-acetylphenyl)-1-(4-fluorophenyl)-3-phenyl propan-1-one (4b)**

**(*R*)-3-(4-acetylphenyl)-1-(4-fluorophenyl)-3-phenyl propan-1-one (4b)**

**(*Rac*)-3-(4-acetylphenyl)-1-(naphthalen-2-yl)-3-phenylpropan-1-one (4c)**

**(*R*)-3-(4-acetylphenyl)-1-(naphthalen-2-yl)-3-phenylpropan-1-one (4c)**

**(*Rac*)-3-(4-acetylphenyl)-3-phenyl-1-(pyridin-3-yl) propan-1-one (4d)**

**(*R*)-3-(4-acetylphenyl)-3-phenyl-1-(pyridin-3-yl) propan-1-one (4d)**

**(*Rac*)-3-(4-acetylphenyl)-3-(furan-2-yl)-1-phenylpropan-1-one (4e)**

**(*S*)-3-(4-acetylphenyl)-3-(furan-2-yl)-1-phenylpropan-1-one (4e)**

**(*Rac*)-3-(4-acetylphenyl)-1-phenyl-3-(thiophen-3-yl)propan-1-one (4f)**

**(*S*)-3-(4-acetylphenyl)-1-phenyl-3-(thiophen-3-yl)propan-1-one (4f)**

**(*rac*)-3-(4-acetylphenyl)-3-(4-methoxyphenyl)-1-phenyl propan-1-one (4g)**

**(*S*)-3-(4-acetylphenyl)-3-(4-methoxyphenyl)-1-phenyl propan-1-one (4g)**

**(*rac*)-3-(4-acetylphenyl)-1-phenylpentan-1-one (4h)**

**(*S*)-3-(4-acetylphenyl)-1-phenylpentan-1-one (4h)**

**(*rac*)-3-(4-acetylphenyl)-1,5-diphenylpentan-1-one (4i)**

**(*S*)-3-(4-acetylphenyl)-1,5-diphenylpentan-1-one (4i)**

**(*rac*)-2-(3-(4-acetylphenyl)-5-oxo-5-phenylpentyl)isoindoline -1,3-dione (4j)**

**(*S*)-2-(3-(4-acetylphenyl)-5-oxo-5-phenylpentyl)isoindoline -1,3-dione (4j)**

**(*rac*)-3-(4-acetylphenyl)-1-phenyldodecan-1-one (4k)**

**(*S*)-3-(4-acetylphenyl)-1-phenyldodecan-1-one (4k)**

**(*rac*)-3-(4-acetylphenyl)-5,5-dimethyl-1-phenylhexan-1-one (4l)**

**(*S*)-3-(4-acetylphenyl)-5,5-dimethyl-1-phenylhexan-1-one (4l)**

**(*Rac*)-3-(4-acetylphenyl)-3-cyclopropyl-1-phenylpropan-1-one (4m)**

**(*R*)-3-(4-acetylphenyl)-3-cyclopropyl-1-phenylpropan-1-one (4m)**

**(*Rac*)-3-(4-acetylphenyl)-3-cyclohexyl-1-phenylpropan-1-one (4n)**

**(*R*)-3-(4-acetylphenyl)-3-cyclohexyl-1-phenylpropan-1-one (4n)**

***tert*-butyl (*Rac*)-4-(1-(4-acetylphenyl)-3-oxo-3-phenylpropyl) piperidine-1-carboxylate (4o)**

***tert*-butyl (*R*)-4-(1-(4-acetylphenyl)-3-oxo-3-phenylpropyl) piperidine-1-carboxylate (4o)**

**(3*Rac*)-3-(4-acetylphenyl)-3-(cyclohex-3-en-1-yl)-1-phenylpropan-1-one (4p)**

**(3*R*)-3-(4-acetylphenyl)-3-(cyclohex-3-en-1-yl)-1-phenylpropan-1-one (4p)**

**(3*Rac*)-3-(4-acetylphenyl)-5-(4-isopropylphenyl)-4-methyl-1-phenylpentan-1-one (4q)**

**(3*R*)-3-(4-acetylphenyl)-5-(4-isopropylphenyl)-4-methyl-1-phenylpentan-1-one (4q)**

**(*Rac*)-3-(4-acetylphenyl)-1-(4-methoxyphenyl)-4-methylpentan-1-one (4r)**

**(*R*)-3-(4-acetylphenyl)-1-(4-methoxyphenyl)-4-methylpentan-1-one (4r)**

**(*Rac*)-1-(4-acetylphenyl)-4,4-dimethyl-1-phenylpentan-3-one (4s)**

**(*R*)-1-(4-acetylphenyl)-4,4-dimethyl-1-phenylpentan-3-one (4s)**

**(*rac*)-3-(4-acetylphenyl)-1-((1s,3S)-adamantan-1-yl)-3-phenylpropan-1-one (4t):**

**(3*R*)-3-(4-acetylphenyl)-1-((1s,3S)-adamantan-1-yl)-3-phenylpropan-1-one (4t):**

**(*Rac*)-1-(4-acetylphenyl)-4-methyl-1-phenylpentan-3-one (4u):**

**(*R*)-1-(4-acetylphenyl)-4-methyl-1-phenylpentan-3-one (4u):**

**(*R*)-*N*-(cyclohexylcarbamoyl)-4-(4-methyl-1-oxo-1-phenylpentan-3-yl)benzene sulfonamide (3m-urea)**

***tert*-butyl (*Rac*)-4-(1-(3-(4-methoxybenzyl)-4-oxo-3,4-dihydroquinazolin-6-yl)-3-oxo-3-phenylpropyl)piperidine-1-carboxylate (4w)**

***tert*-butyl (*R*)-4-(1-(3-(4-methoxybenzyl)-4-oxo-3,4-dihydroquinazolin-6-yl)-3-oxo-3-phenylpropyl)piperidine-1-carboxylate (4w)**

**(3a*R*,5*R*,6*S*,6a*R*)-5-((*rac*)-2,2-dimethyl-1,3-dioxolan-4-yl)-2,2-dimethyltetrahydrofuro[2,3-*d*][1,3]dioxol-6-yl 4-((*R*)-4-methyl-1-oxo-1-phenylpentan-3-yl)benzoate (5a)**

**(3a*R*,5*R*,6*S*,6a*R*)-5-((*S*)-2,2-dimethyl-1,3-dioxolan-4-yl)-2,2-dimethyltetrahydrofuro[2,3-*d*][1,3]dioxol-6-yl 4-((*R*)-4-methyl-1-oxo-1-phenylpentan-3-yl)benzoate (5a)**

**(*Rac*)-2,5,7,8-tetramethyl-2-((4*R*,8*R*)-4,8,12-trimethyltridecyl)chroman-6-yl 4-((*R*)-4-methyl-1-oxo-1-phenylpentan-3-yl)benzoate (5b)**

**(*R*)-2,5,7,8-tetramethyl-2-((4*R*,8*R*)-4,8,12-trimethyltridecyl)chroman-6-yl 4-((*R*)-4-methyl-1-oxo-1-phenylpentan-3-yl)benzoate (5b)**

**(8*R*,9*S*,13*S*,14*S*)-13-methyl-17-oxo-7,8,9,11,12,13,14,15,16,17-decahydro-6*H*-cyclopenta[*a*]phenanthren-3-yl 4-((*rac*)-4-methyl-1-oxo-1-phenylpentan-3-yl)benzoate (5c)**

**(8*R*,9*S*,13*S*,14*S*)-13-methyl-17-oxo-7,8,9,11,12,13,14,15,16,17-decahydro-6*H*-cyclopenta[*a*]phenanthren-3-yl 4-((*R*)-4-methyl-1-oxo-1-phenylpentan-3-yl)benzoate (5c)**

**(*Rac*)-4-methyl-1-phenyl-3-(4-(4-(2,3,4-trimethoxybenzyl)piperazine-1-carbonyl) phenyl)pentan-1-one (5d)**

**(*R*)-4-methyl-1-phenyl-3-(4-(4-(2,3,4-trimethoxybenzyl)piperazine-1-carbonyl) phenyl)pentan-1-one (5d)**

**(*Rac*)-4-methyl-1-phenyl-3-(4-(4-(4-phenylquinolin-2-yl)piperazine-1-carbonyl) phenyl)pentan-1-one (5e)**

**(*R*)-4-methyl-1-phenyl-3-(4-(4-(4-phenylquinolin-2-yl)piperazine-1-carbonyl) phenyl)pentan-1-one (5e)**

**(*Rac*)-3-(3-(4-(cyclopropanecarbonyl)piperazine-1-carbonyl)-4-fluorophenyl)-4-methyl-1-phenylpentan-1-one (5f)**

**(*R*)-3-(3-(4-(cyclopropanecarbonyl)piperazine-1-carbonyl)-4-fluorophenyl)-4-methyl-1-phenylpentan-1-one (5f)**

***tert*-butyl (*Rac*)-4-(1-(4-((3-chloro-4-fluorophenyl)amino)quinazolin-6-yl)-3-oxo-3-phenylpropyl)piperidine-1-carboxylate (5g)**

***tert*-butyl (*R*)-4-(1-(4-((3-chloro-4-fluorophenyl)amino)quinazolin-6-yl)-3-oxo-3-phenylpropyl)piperidine-1-carboxylate (5g)**
